# Abnormal intestinal microbial colonization in prenatally stressed offspring is related to lung and intestinal cytokine expression

**DOI:** 10.3389/fmicb.2026.1813467

**Published:** 2026-04-09

**Authors:** Audrey F. Duff, Michael T. Bailey

**Affiliations:** 1 Center for Microbe and Immunity Research, Abigail Wexner Research Institute at Nationwide Children’s Hospital, Columbus, OH, United States; 2 Oral and Gastrointestinal Microbiology Research Affinity Group, Abigail Wexner Research Institute at Nationwide Children’s Hospital, Columbus, OH, United States; 3 Department of Pediatrics, The Ohio State University, Wexner Medical Center, Columbus, OH, United States

**Keywords:** cytokine gene expression, intestinal microbiome, intestine, lung, MyD88, prenatal stress

## Abstract

**Introduction:**

Prenatal stress (PNS) is associated with deleterious effects on childhood health and wellbeing. Among these consequential health repercussions, PNS-exposed children are at increased risk for acquiring early-life infections, with respiratory infections frequently reported. Stress-induced perturbations in the maternal microbiome during pregnancy represent a key link between stress *in utero* and aberrant offspring development and can drive abnormal pioneer colonization of offspring microbiomes.

**Methods:**

Using a mouse model of PNS, we aimed to understand the extent to which these early-life intestinal microbial perturbations are related to intestinal and lung cytokine gene expression. The intestinal microbiome alongside intestinal and lung tissue gene expression were assessed over the first five weeks of life in PNS-exposed offspring to characterize basal cytokine differences in relation to intestinal microbial composition.

**Results:**

In addition to significant changes in microbiome diversity and differential abundance, PNS offspring exhibited significant differences in ileal and lung cytokines characterized by overall increased interferon and proinflammatory gene signatures. PNS-associated microbiome changes also correlated to gene expression in both the ileum and lung. Finally, PNS-associated cytokine differences were not observed in *MyD88^−/−^* offspring which lack the ability to initiate inflammatory responses through microbially-stimulated toll-like receptor signaling.

**Conclusion:**

These findings suggest that PNS-mediated changes in the early-life microbiome are linked to respiratory and ileal immune development and the microbe-immune interactions are MyD88 pathway-dependent.

## Introduction

The prenatal period is a sensitive stage of life wherein an abundance of maternally derived signals can influence the developing fetus. Among these signals, elevated levels of maternal stress during pregnancy, or prenatal stress (PNS), has become a notably recognized contributing factor to consequential psychopathologies and health adversities in exposed offspring (Stepanikova et al., 2019; den Van Bergh et al., 2020; Bush et al., 2021; Babineau et al., 2022). The influence of PNS on offspring development is partly mediated through intrauterine exposure to excessive levels of maternal glucocorticoids (Seckl and Holmes, 2007) and stress-associated alterations in maternal gut microbiome composition (Chen et al., 2022) that can exert lasting effects on fetal biological systems including the nervous, respiratory, and immune systems. Studies of child and adolescent cohorts have described associations with PNS exposure and increased risk of behavioral and mood disorders such as anxiety, depression, and autism spectrum disorder (Kim et al., 2015) which have been widely recapitulated in animal models (Weinstock, 2016; Zohar et al., 2016; Gur et al., 2017, 2019; Chen et al., 2020; Zoubovsky et al., 2022). Respiratory dysfunction has also been observed in epidemiological studies that describe relationships between PNS exposure and incidence of asthma and atopy in children (Rosa et al., 2016; Flanigan et al., 2018), a finding similarly supported by animal models (Nogueira et al., 1999; Pincus-Knackstedt et al., 2006; Lim et al., 2014). Moreover, longitudinal studies report positive associations between PNS exposure and risk of acquiring infectious diseases in childhood, which as of 2024, remain a major source of worldwide morbidity and mortality in children under the age of five (United Nations Inter-Agency Group for Child Mortality Estimation (UN IGME), 2025).

In a nationwide cohort of Danish children 0–14 years of age, Nielson et al. reported that PNS exposure led to a respective 25 and 31% increased risk for hospitalization with severe (sepsis, meningitis, pyelonephritis, osteomyelitis, ethmoiditis) or less severe infectious diseases (pneumonia, upper respiratory infections, gastroenteritis, cystitis, bronchitis, conjunctivitis, influenza), with the greatest susceptibility observed in the first year of life (Nielsen et al., 2011). Others have similarly reported statistically increased frequency of early-life throat, ear, gastric, and respiratory infections associated with stressful life events during pregnancy (Henriksen and Thuen, 2015; Robinson et al., 2024). Importantly, the increased risk associated with acquiring these infections, including respiratory infection, persist even when controlling for maternal health status and behaviors during pregnancy (Stepanikova et al., 2019). On a worldwide scale, lower respiratory tract infections account for approximately 20% of deaths among newborns and children under five (United Nations Inter-Agency Group for Child Mortality Estimation (UN IGME), 2025), and infection of the respiratory tract specifically is a commonly reported childhood morbidity associated with PNS (Lee et al., 2014; Phelan et al., 2015; Stepanikova et al., 2019; Orr et al., 2024).

While mechanisms driving disparities in early-life disease susceptibility are likely multifaceted, disruption of the gut microbiome is one pathway through which PNS could shape offspring behavioral and health outcomes (Chen et al., 2020, 2022). It has been established that PNS can significantly alter maternal and offspring intestinal microbiome composition (Gur et al., 2017, 2019; Galley et al., 2023) as well as levels of microbial metabolites (Galley et al., 2021), but the extent to which these microbial perturbations are related to disease risk is unclear. The importance of the microbiota is especially emphasized early in life. Commensal bacteria have pivotal roles in the maturation of appropriate tolerogenic and protective immune responses which can exert permanent programming effects on neonatal immunity and modify disease susceptibility (Gensollen et al., 2016). Furthermore, intricate intertwining of the gut microbiota and immune system can influence extraintestinal organs via complex, bidirectional networks such as the well-known gut-brain-axis, but also less discussed networks such as the gut-lung axis. With respect to the latter, perturbations to the gut microbiome early in life can indeed contribute to dysregulated respiratory immune responses (McAleer and Kolls, 2018), and have been implicated in the onset of childhood asthma and allergic disease (Yamamoto-Hanada et al., 2017; Fishman et al., 2019).

Given the cooccurrence of changes in microbiome composition and increased risk of neurobehavioral abnormalities, infection, and respiratory dysfunction in PNS offspring, it is reasonable to speculate that PNS-associated changes in the microbiome and associated immune dysregulation are important driving forces behind health outcomes in early childhood. Here, we hypothesized that aberrant early-life colonization of the intestinal microbiome and early-life cytokine expression are interconnected through bidirectional host–microbe interactions influenced by PNS exposure. The primary aim of this work was to first characterize baseline cytokines and microbiome composition in PNS-exposed offspring in the absence of any kind of additional immune-activating challenge. Using an established rodent model of prenatal restraint stress, we characterized differences in intestinal and lung gene expression and intestinal microbiome composition in offspring over the first 5 weeks of life. Our results demonstrate that even in the absence of immune challenge, PNS exposure is sufficient to induce significant, tissue-specific dysregulation of proinflammatory and interferon gene expression, which were related to compositional changes in the microbiome. Microbiome-mediated immune stimulation occurs largely through toll-like receptor (TLR) signaling that is initiated in response to both pathogenic and commensal bacterial signals (Akira et al., 2006). Our results further demonstrated that ablation of myeloid differentiation primary response protein 88 (MyD88), a critical adaptor protein that mediates TLR induction of inflammatory responses, significantly attenuated early-life immune dysregulation associated with PNS. These results suggest a potential role of intestinal microbial composition and inflammatory signaling pathways in the PNS offspring health paradigm.

## Results

A total of 5 control litters and 6 PNS-exposed litters were included in this study. All control dams remained undisturbed throughout parturition and weaning, and PNS dams were similarly left undisturbed following the PNS period (Gestational day (GD)10-GD16; see Materials and Methods). All subsequent analyses focused solely on offspring immune gene expression in the intestine and lung and intestinal microbiome composition. A subset of offspring were randomly sampled from each litter across four time points: birth (d0), day 7 (d7), day 21 (d21), and day 35 (d35). Characteristics of the study cohort are detailed in Table 1.

**Table 1:** Experiment summary table.

Dam (Litter) ID	Dam prenatal treatment	Total pups	Offspring analyzed per time point			
			d0	d7	d21	d35
			*n*	*n* (M/F)	*n* (M/F)	*n* (M/F)
A	Control	5	2	-	3 (0/3)	-
B	Control	9	3	2 (1/1)	2 (0/2)	2 (1/1)
C	Control	8	2	2 (0/2)	2 (0/2)	2 (2/0)
D	Control	6	1	1 (0/1)	2 (2/0)	2 (2/0)
E	Control	8	2	2 (0/2)	2 (2/0)	2 (1/1)
F	PNS	6	2	2 (1/1)	2 (2/0)	-
G	PNS	7	2	2 (1/1)	3 (2/1)	-
H	PNS	7	1	2 (2/0)	2 (0/2)	2 (0/2)
I	PNS	9	3	1 (1/0)	2 (1/1)	3 (1/2)
J	PNS	9	2	2 (0/2)	3 (2/1)	2 (0/2)
K	PNS	8	2	2 (2/0)	2 (2/0)	2 (1/1)
Control Dams *n* = 5	Control Offspring *n* =	36	10	7 (1/6)	11 (4/7)	8 (6/2)
PNS Dams *n* = 6	PNS Offspring *n* =	46	12	11 (7/4)	14 (9/5)	9 (2/7)

(M/F): Number of male/female mice analyzed from each litter at a specified time point.

Sex was not assessed on d0.

### Exposure to prenatal stress alters inflammatory cytokine expression in the lung and intestine

#### Offspring lung gene expression

**Figure 1:**
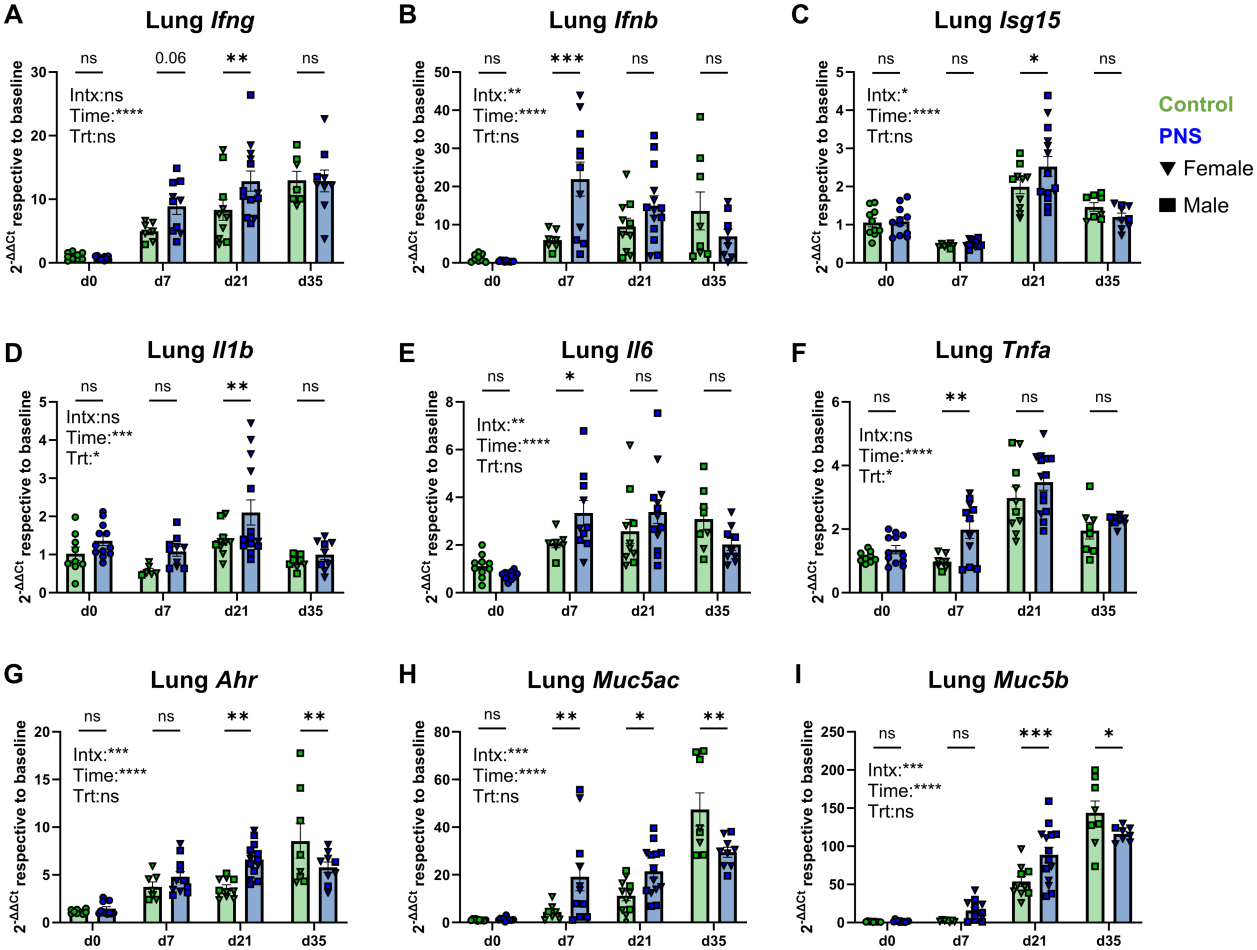
Lung tissue gene expression. Lung tissue interferon **(A–C)**, classical proinflammatory **(D–F)**, and aryl hydrocarbon receptor and mucin gene expression **(G–I)** was assessed in control and PNS offspring at d0, d7, d21, and d35. Values are expressed using the 2^-ΔΔCt^ method relative to control values at baseline to highlight how exposure to chronic prenatal stress altered gene expression over time. Control values are denoted by green bars and PNS values are denoted by blue bars; females are denoted by triangles and males are denoted by squares. d0 (n_control_ = 10; n_PNS_ = 12), d7 (n_control♀_ = 6, n_control♂_ = 1; n_PNS♀_ = 4, n_PNS♂_ = 7), d21 (n_control♀_ = 7, n_control♂_ = 4; n_PNS♀_ = 5, n_PNS♂_ = 9), d35 (n_control♀_ = 2, n_control♂_ = 6; n_PNS♀_ = 7, n_PNS♂_ = 2). Missing values are the result of outlier removal or due to samples with expression that was below qPCR detection limits. Bar graphs denote mean ± SEM. Fixed effects and their interaction are denoted on each graph, and post-hoc comparisons between control and PNS mice are denoted above each respective time point. Data were analyzed by linear mixed effects models examining effects of treatment, time point, and their interaction with litter included as a random intercept. *p ≤ 0.05, **p ≤ 0.01, ***p ≤ 0.001, ****p ≤ 0.0001. Intx, interaction; Time, ain effect of time point; Trt, ain effect of treatment.

Immune gene expression was assessed in offspring at four independent time points spanning birth (d0) through adulthood (d35). Sex differences in gene expression were investigated in all tissues for each gene and time point and are discussed where significant treatment by time by sex interactions were observed. Offspring from dams exposed to 2 h of restraint stress on gestational days 10–16 had an overall increase in proinflammatory gene signatures characterized by interferon (IFN), classical proinflammatory cytokine, and mucin gene expression compared to offspring from control pregnancies (Figure 1). An independent effect of time (*p <* 0.001) supported the observation that as offspring aged, lung *Ifng* expression naturally increased regardless of prenatal conditions. However, PNS exposure led to increased lung *Ifng* gene expression at d21 (*p* = 0.01) relative to controls (Figure 1A). Lung *Ifnb* had a significant treatment by time interaction (*p* < 0.01) suggesting age-dependent effects of PNS exposure with higher expression observed in PNS offspring at d7 relative to controls (*p* < 0.001; Figure 1B). Interferon-stimulated gene (*Isg*)*15*—which plays a role in a variety of cellular processes and response to viral infection upon Type I IFN stimulation (e.g., *Ifnb*)—also had a significant interaction of treatment and time (*p* < 0.05). Expression of *Isg15* peaked at d21 in both groups, but levels were higher in offspring exposed to PNS (*p* = 0.05; Figure 1C). In general, lung interferon-related gene expression tended to be elevated in PNS offspring, particularly early in life.

Independent main effects of time (*p* < 0.001) and treatment (*p* < 0.05) were observed for lung *Il1b*, with significantly higher levels of expression observed in PNS offspring at d21 (*p* = 0.01; Figure 1D). A significant interaction of sex, treatment, and time point was also noted for d21 lung *Il1b* (*p* < 0.01; Supplementary Figure 1A), with PNS females expressing significantly higher *Il1b* relative to PNS males (*p* < 0.001) and relative to control females (*p* < 0.001). Lung tissue *Il6* had a significant treatment by time interaction (*p* < 0.001) with higher levels of expression in PNS offspring relative to controls at d7 (*p* = 0.05; Figure 1E). Lung *Tnfa* peaked at d21 across offspring (main effect of time *p* < 0.001), but main effects of treatment (*p* < 0.05) were most evident at d7 where expression was significantly higher in PNS lungs compared to controls (*p* = 0.01; Figure 1F).

Additional immunomodulatory genes including aryl hydrocarbon receptor (Ahr)—a key transcription factor that responds to both microbial- and host-derived ligands—and key lung mucins, *Muc5ac* and *Muc5b*, were assessed. All three genes possessed significant interaction effects (*p* < 0.001) and increased in expression over time relative to baseline, but PNS-mediated changes were dependent upon age (Figures 1G–I). Exposure to PNS was associated with greater expression of lung *Ahr* at d21 (*p* = 0.002) but lower expression at d35, post-weaning (*p* = 0.01; Figure 1G) relative to controls. Between the two lung mucins, similar trends in expression were observed. PNS offspring had increased levels of expression at d7 (*Muc5ac*, *p* = 0.008) and d21 (*Muc5ac* *p* = 0.03 and *Muc5b* and *p* = 0.001), and reduced expression of *Muc5ac* and *Muc5b* at d35 (*p* = 0.002 and *p* = 0.02, respectively; Figures 1H,I) when compared to controls. Changes in anti-inflammatory and reparative responses were assessed by *Il10* and *Tgfb1* expression, but no differences were observed between control and PNS offspring lungs (Supplementary Figures 2A,B). Expression of *Foxp3*, an important regulatory transcription factor associated with immune tolerance, had a significant treatment by time interaction (*p* < 0.001), and was higher in the lungs of PNS offspring at d21 (*p* < 0.001) but lower at d35 (*p* = 0.02; Supplementary Figure 2C) relative to controls. Exposure to PNS has been associated with a skewing of T helper (T_h_)1/T_h_2 responses toward a T_h_2 bias. However, quantification of key T_h_1 and T_h_2 cytokine expression showed no differences between PNS and control offspring *Il18* (T_h_1 cytokine; Supplementary Figure 2D) nor *Il4* (T_h_2 cytokine) expression in the lungs (Supplementary Figure 2E). Finally, expression of key allergic response cytokines, *Il5* and *Il13*, was assessed given increased likelihood of early-life asthma and atopy in children exposed PNS. In line with these reports, both *Il5* (*p* = 0.02) and *Il13* (*p* = 0.003) expression was significantly higher in PNS offspring lungs early in life at d7 (Supplementary Figures 2F,G). Linear mixed effects modeling for lung gene expression is available in Supplementary File 1.

#### Offspring ileal gene expression

**Figure 2:**
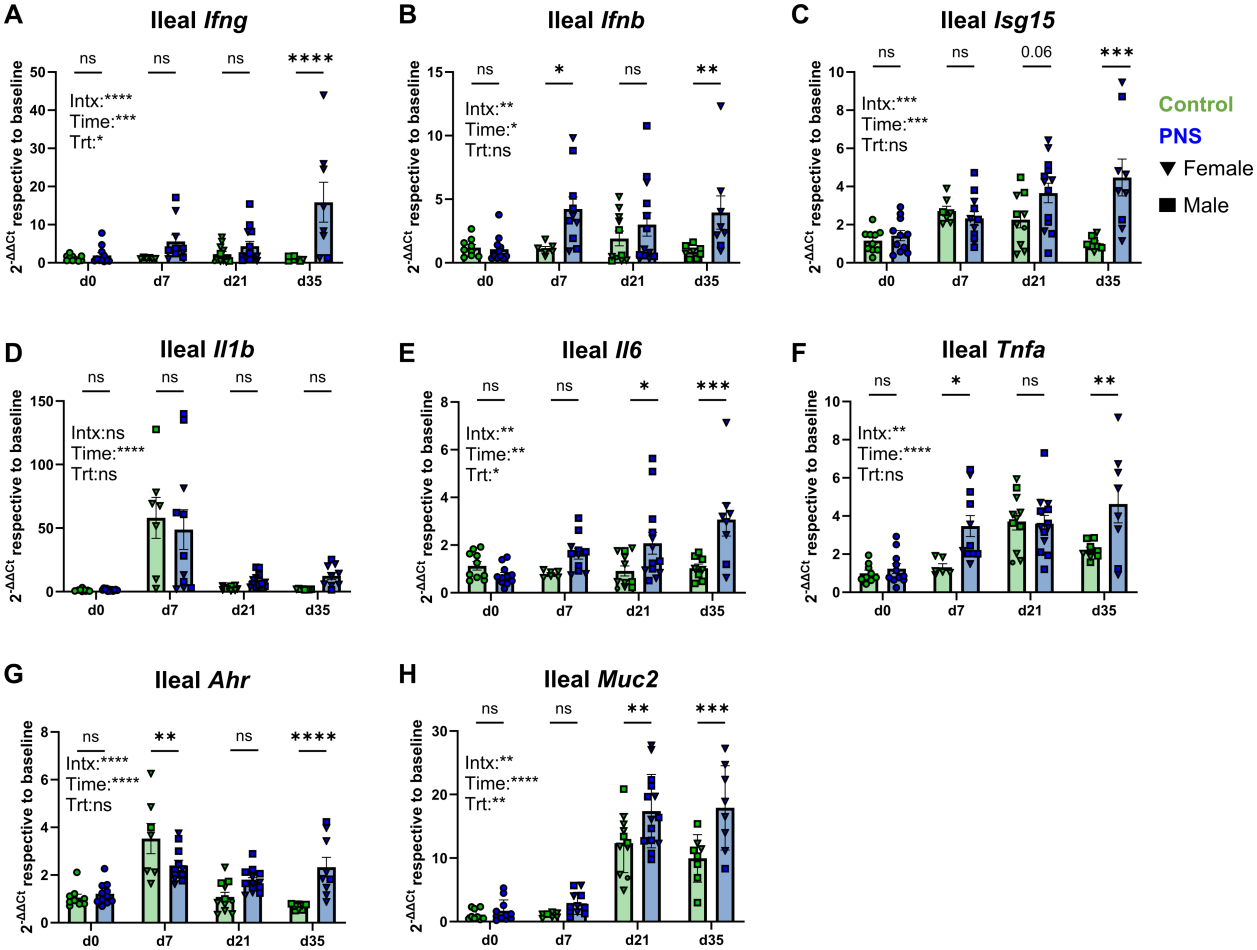
Ileum tissue gene expression. Ileum tissue interferon **(A–C)**, classical proinflammatory **(D–F)**, and aryl hydrocarbon receptor and mucin gene expression **(G,H)** was assessed in control and PNS offspring at d0, d7, d21, and d35. Values are expressed using the 2^-ΔΔCt^ method relative to control values at baseline to highlight how exposure to chronic prenatal stress altered gene expression over time. Control values are denoted by green bars and PNS values are denoted by blue bars; females are denoted by triangles and males are denoted by squares. d0 (*nco_n_*trol = 10; n_PNS_ = 12), d7 (n_control♀_ = 6, n_control♂_ = 1; n_PNS♀_ = 4, n_PNS♂_ = 7), d21 (n_control♀_ = 7, n_control♂_ = 4; n_PNS♀_ = 5, n_PNS♂_ = 9), d35 (n_control♀_ = 2, n_control♂_ = 6; n_PNS♀_ = 7, n_PNS♂_ = 2). Missing values are the result of outlier removal or due to samples with expression that was below qPCR detection limits. Bar graphs denote mean ± SEM. Fixed effects and their interaction are denoted on each graph, and post-hoc comparisons between control and PNS mice are denoted above each respective time point. Data were analyzed by linear mixed effects models examining effects of treatment, time point, and their interaction with litter included as a random intercept. *p ≤ 0.05, **p ≤ 0.01, ***p ≤ 0.001, ****p ≤ 0.0001. Intx, interaction; Time, main effect of time point; Trt, main effect of treatment.

Interferon, proinflammatory, and immunoregulatory gene expression tended to be similarly elevated in PNS offspring ileums. A significant interaction effect (*p* < 0.001) for ileal *Ifng* indicated age-dependent effects of PNS exposure, with significantly higher expression in PNS offspring relative to controls at d35 (*p* < 0.001; Figure 2A). An interaction of time, treatment, and sex was also detected for d35 ileal *Ifng* showing the same pattern noted in the lung of elevated expression in PNS females relative to PNS males (*p* < 0.05) and control females (*p* < 0.001; Supplementary Figure 1B). An interaction of treatment and time was observed for *Ifnb* expression which was significantly higher in PNS ileums than controls at d7 (*p* = 0.04) and d35 (*p* = 0.01; Figure 2B). Ileal *Isg15* similarly exhibited an interaction effect (*p* < 0.001) that aligned with heightened expression in PNS offspring at d35 (*p* < 0.001) compared to controls, but not earlier in life (Figure 2C).

Proinflammatory gene signatures in the ileum were most notably elevated in PNS offspring later in life, with the exception of *Il1b* which peaked at d7 (*p* > 0.05; Figure 2D). Both ileal *Il6* and *Tnfa* showed significant interaction effects (*p* < 0.01) that highlighted an age-dependent effect of PNS-mediated changes, particularly post-weaning. Offspring exposed to PNS showed a general increase in *Il6* expression with time, significantly higher than controls at d21 (*p* = 0.02) and d35 (*p* < 0.001; Figure 2E), whereas expression of *Tnfa* was higher in PNS offspring at d7 (*p* = 0.02) and d35 (*p* = 0.003; Figure 2F).

An interaction effect for *Ahr* (*p* < 0.001) suggests PNS-mediated effects on expression were age-dependent in agreement with lower expression at d7 (*p* = 0.01), but higher expression at d35 (*p* < 0.001) relative to controls (Figure 2G). An interaction effect (*p* < 0.05) for ileal *Muc2*, a key intestinal mucin, corresponded with higher expression in PNS offspring at d21 (*p* = 0.008) and d35 (*p* < 0.001) but not at earlier time points. Alternative immunomodulatory genes *Il10*, *Foxp3*, and *Il18* also possessed significant treatment and time interaction effects (*p* < 0.001). Consistent with heightened inflammatory gene expression at the same time point, PNS offspring had higher *Il10* expression at d35 (*p* < 0.001; Supplementary Figure 3A). In PNS offspring, ileal *Foxp3* was lower at d21 (*p* = 0.01; Supplementary Figure 3C), and ileal *Il18* was higher at d35 relative to controls (*p* < 0.001; Supplementary Figure 3D). Ileal expression of *Il5* and *Il13* was also impacted by PNS exposure, with greater *Il5* expression at d0 (*p* = 0.03; Supplementary Figure 3F) and greater *Il13* expression at d7 (*p* = 0.02) and d21 (*p* = 0.03) in PNS offspring relative to controls (Supplementary Figure 3G). Overall ileal gene expression differences between PNS and control offspring were most pronounced at d35, two weeks post-weaning. Linear mixed effects modeling for ileal gene expression is available in Supplementary File 2.

#### Offspring colonic gene expression

**Figure 3:**
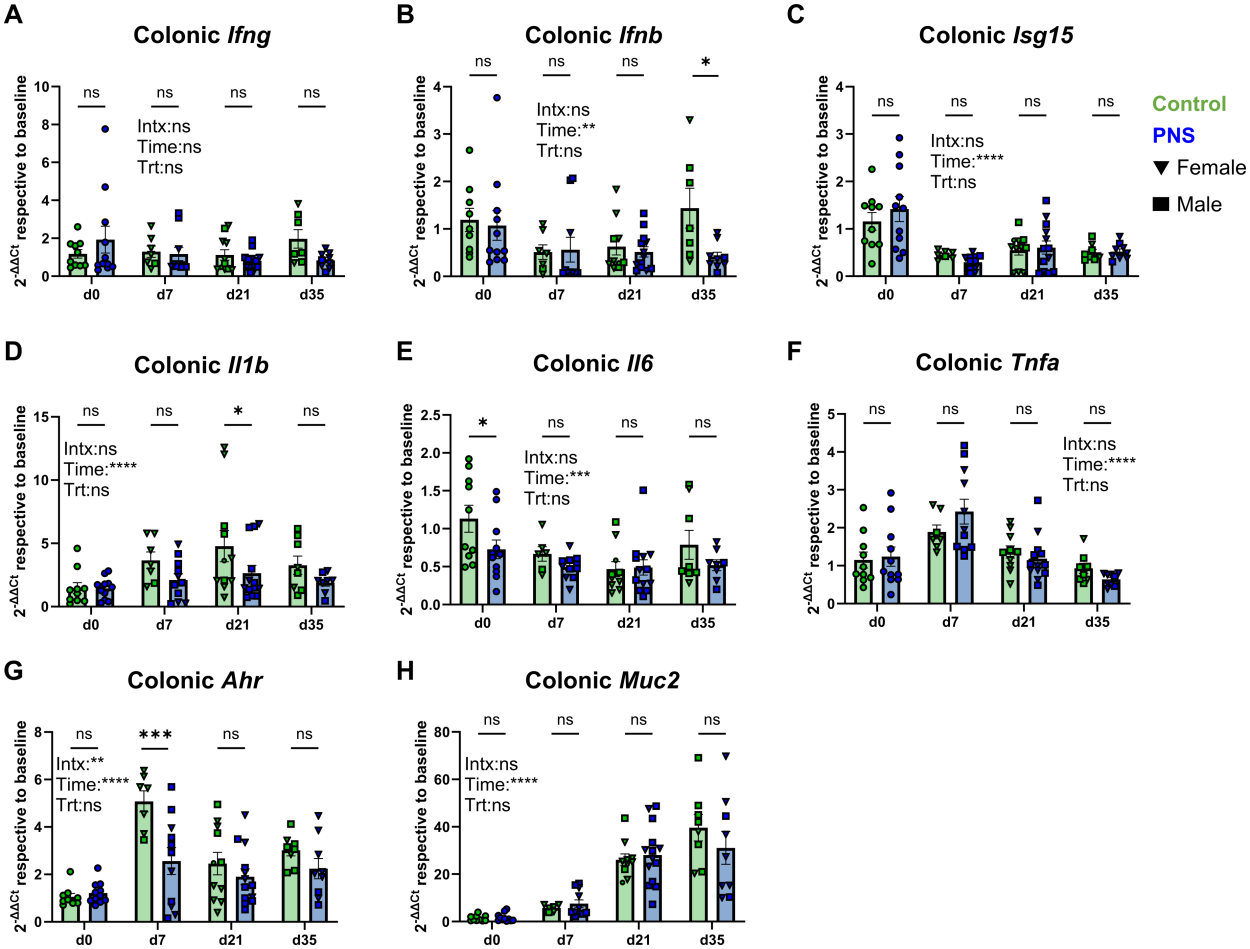
Colon tissue gene expression. Colon tissue interferon **(A–C)**, classical proinflammatory **(D–F)**, and aryl hydrocarbon receptor and mucin gene expression **(G,H)** was assessed in control and PNS offspring at d0, d7, d21, and d35. Values are expressed using the 2^-ΔΔCt^ method relative to control values at baseline to highlight how exposure to chronic prenatal stress altered gene expression over time. Control values are denoted by green bars and PNS values are denoted by blue bars; females are denoted by triangles and males are denoted by squares. d0 (*n*_control_ = 10; *n*_PNS_ = 12), d7 (*n*_control♀_ = 6, *n*_control♂_ = 1; *n*_PNS♀_ = 4, *n*_PNS♂_ = 7), d21 (*n*_control♀_ = 7, *n*_control♂_ = 4; *n*_PNS♀_ = 5, *n*_PNS♂_ = 9), d35 (*n*_control♀_ = 2, *n*_control♂_ = 6; *n*_PNS♀_ = 7, *n*_PNS♂_ = 2). Missing values are the result of outlier removal or due to samples with expression that was below qPCR detection limits. Bar graphs denote mean ± SEM. Fixed effects and their interaction are denoted on each graph, and post-hoc comparisons between control and PNS mice are denoted above each respective time point. Data were analyzed by linear mixed effects models examining effects of treatment, time point, and their interaction with litter included as a random intercept. *p ≤ 0.05, **p ≤ 0.01, ***p ≤ 0.001, ****p ≤ 0.0001. Intx, interaction; Time, main effect of time point; Trt, main effect of treatment.

Unexpectedly, minimal differences in offspring gene expression were observed in colonic tissue. With the exception of decreased *Ifnb* in d35 PNS colons (*p* = 0.05), no differences were detected in colonic interferon genes (Figures 3A–C). There was, however, a main effect of time (*p* < 0.001) that corresponded with consistently lower *Isg15* expression at all time points after d0 implying a degree of reduced colonic interferon-related immune stimulation with age. PNS offspring exhibited lower expression levels of colonic *Il1b* at d21 (*p* = 0.03) and *Il6* at d0 (*p* = 0.02) relative to controls, with no differences in *Tnfa* expression between offspring (Figures 3D–F). An interaction effect was observed for colonic *Ahr* (*p* < 0.01), and PNS exposure was associated with lower expression at d7 (*p* < 0.001) relative to controls (Figure 3G). Though a steady increase in colonic *Muc2* was noted with increased age (main effect of time *p* < 0.001), no differences were observed between PNS and control offspring (Figure 3H). Relative to controls, PNS exposure was also associated with lower levels of *Foxp3* at d35 (*p* = 0.02), *Il4* at d35 (*p* = 0.01), and an increase in *Il5* at d0 (*p* = 0.02; Supplementary Figure 4). Linear mixed effects modeling for colonic gene expression is available in Supplementary File 3.

### Exposure to prenatal stress alters offspring intestinal microbiome composition

#### Offspring intestinal diversity

**Figure 4:**
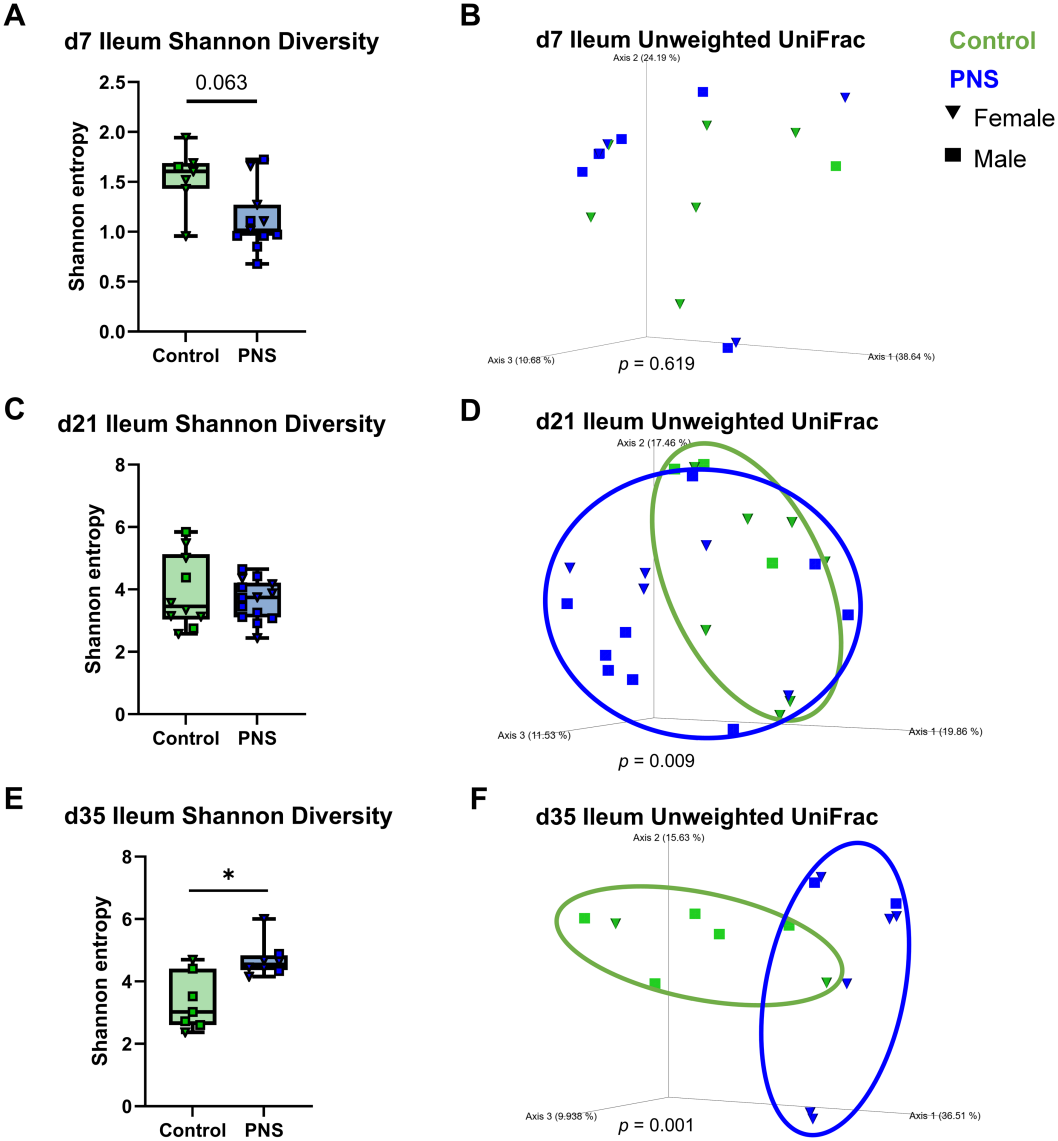
Ileum microbiome diversity. Shannon alpha diversity and principal coordinate analysis plots of unweighted UniFrac beta diversity in ileum contents at d7 **(A,B)**, d21 **(C,D)**, and d35 **(E,F)**. Differences in alpha diversity were analyzed in QIIME 2.0 by Kruskal-Wallis tests; *p ≤ 0.05, **p ≤ 0.01. Alpha diversity values are represented as box and whisker plots where boxes denote interquartile range and lower and upper whiskers denote minimum and maximum, respectively. Differences in beta diversity distances were analyzed in QIIME 2.0 by PERMANOVA with 999 randomizations of the data; ellipses have been drawn around groups where beta diversity distances are significantly different (*p* ≤ 0.05). Control offspring are denoted in green and PNS offspring are denoted in blue; females are denoted by triangles and males are denoted by squares. d7 (n_control♀_ = 6, n_control♂_ = 1; n_PNS♀_ = 4, n_PNS♂_ = 7), d21 (n_control♀_ = 7, n_control♂_ = 4; n_PNS♀_ = 5, n_PNS♂_ = 9), d35 (n_control♀_ = 2, n_control♂_ = 6; n_PNS♀_ = 7, n_PNS♂_ = 2). Missing values were dropped due to rarefaction.

Microbiome analyses were conducted at d7, d21, and d35 in both ileal and colonic contents. Differences in intestinal microbial composition were assessed by measures of alpha and beta diversity, as well as a compositionally aware measure of differential abundance (DA). Alpha diversity was quantified using Shannon’s diversity, and beta diversity was measured using unweighted UniFrac. Prior to time point specific investigation, alpha and beta diversity were analyzed using multi-factorial analyses to control for litter effects in order to validate that litter-to-litter variation did not influence observed treatment effects. Indeed, treatment by time interactions were noted for all metrics suggesting that while treatment effects varied over time, litter was not a confounding factor where significant treatment effects were noted (Supplementary File 4). No differences in alpha or beta diversity were noted in the ileum at d7 (Figures 4A,B), however alpha diversity trended lower in PNS offspring (*p* = 0.06). Overall, ileal diversity appeared to be low at d7 as noted by alpha diversity values 2- to 4-times lower than noted at subsequent time points. At d21, no differences between PNS and controls were detected in ileal alpha diversity (Figure 4C), but a significant shift in beta diversity became apparent (*p* = 0.009; Figure 4D). By d35, differences in ileal diversity were even more clear and showed increased alpha diversity in PNS offspring (*p* = 0.02; Figure 4E) accompanied by a larger separation of PNS versus control offspring beta diversity (*p* = 0.001; Figure 4F). No sex differences were noted within treatment groups across time points or metrics for ileal alpha or beta diversity (Supplementary Table 1).

**Figure 5:**
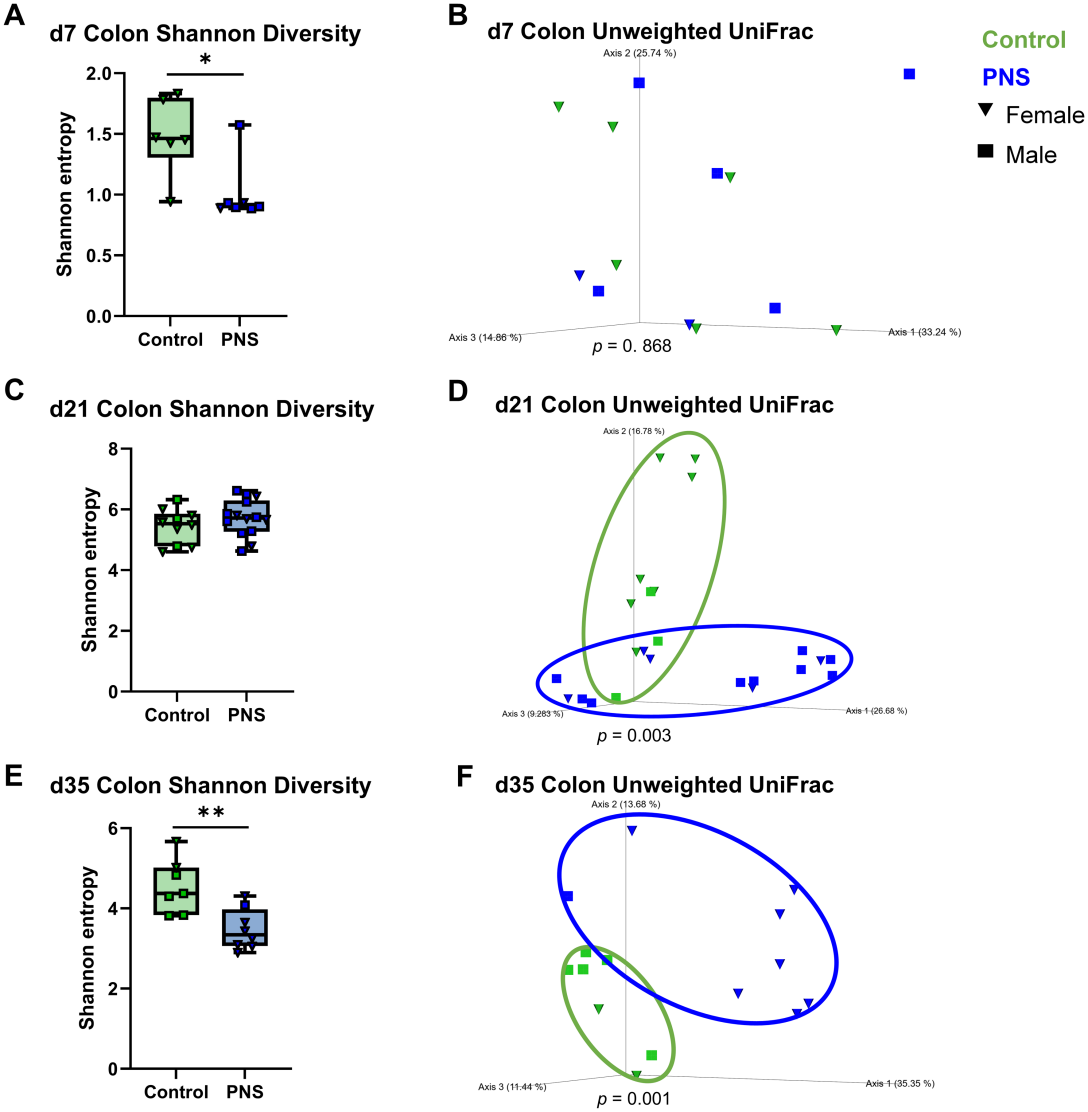
Colon microbiome diversity. Shannon alpha diversity and principal coordinate analysis plots of unweighted UniFrac beta diversity in colon contents at d7 **(A,B)**, d21 **(C,D)**, and d35 **(E,F)**. Differences in alpha diversity were analyzed in QIIME 2.0 by Kruskal-Wallis tests; *p ≤ 0.05, **p ≤ 0.01. Alpha diversity values are represented as box and whisker plots where boxes denote interquartile range and lower and upper whiskers denote minimum and maximum, respectively. Differences in beta diversity distances were analyzed in QIIME 2.0 by PERMANOVA with 999 randomizations of the data; ellipses have been drawn around groups where beta diversity distances are significantly different (*p* ≤ 0.05). Control offspring are denoted in green and PNS offspring are denoted in blue; females are denoted by triangles and males are denoted by squares. d7 (*n*_control♀_ = 6, *n*_control♂_ = 1; *n*_PNS♀_ = 4, *n*_PNS♂_ = 7), d21 (*n*_control♀_ = 7, *n*_control♂_ = 4; *n*_PNS♀_ = 5, *n*_PNS♂_ = 9), d35 (*n*_control♀_ = 2, *n*_control♂_ = 6; *n*_PNS♀_ = 7, *n*_PNS♂_ = 2). Missing values were dropped due to rarefaction.

Despite overall low colonic alpha diversity at d7, a significant reduction was detected in PNS offspring with a uniform treatment effect across pups (*p* = 0.02; Figure 5A). As in the ileum, no differences in d7 beta diversity were detected between PNS and controls (Figure 5B). No differences in d21 colonic alpha diversity were detected (Figure 5C), though very clear differences in beta diversity were observed (*p* = 0.003; Figure 5D). Treatment differences were evident in both diversity metrics in d35 colonic contents, with a significant reduction in PNS alpha diversity (*p* = 0.008) and distinct separation of PNS and control offspring beta diversity (*p* = 0.001) (Figures 5E, F). With the exception of control offspring unweighted UniFrac distances at d21 (*p* = 0.04; Supplementary Table 1), no sex differences were observed for colonic diversity across time points or metrics.

#### Offspring intestinal differential abundance

To determine taxa that may be driving compositional differences, analysis of compositions of microbiomes with bias correction (ANCOM-BC) was used to probe the dataset at the genus level. ANCOM-BC bar plots denote microbes that were significantly (*q* ≤ 0.05) differentially enriched (blue) or depleted (orange) in PNS offspring relative to controls. No DA microbes were detected at d7 in either the ileum or the colon. Plots that visualize relative abundance of DA microbes only can be found in Supplementary Figure 5.

**Figure 6:**
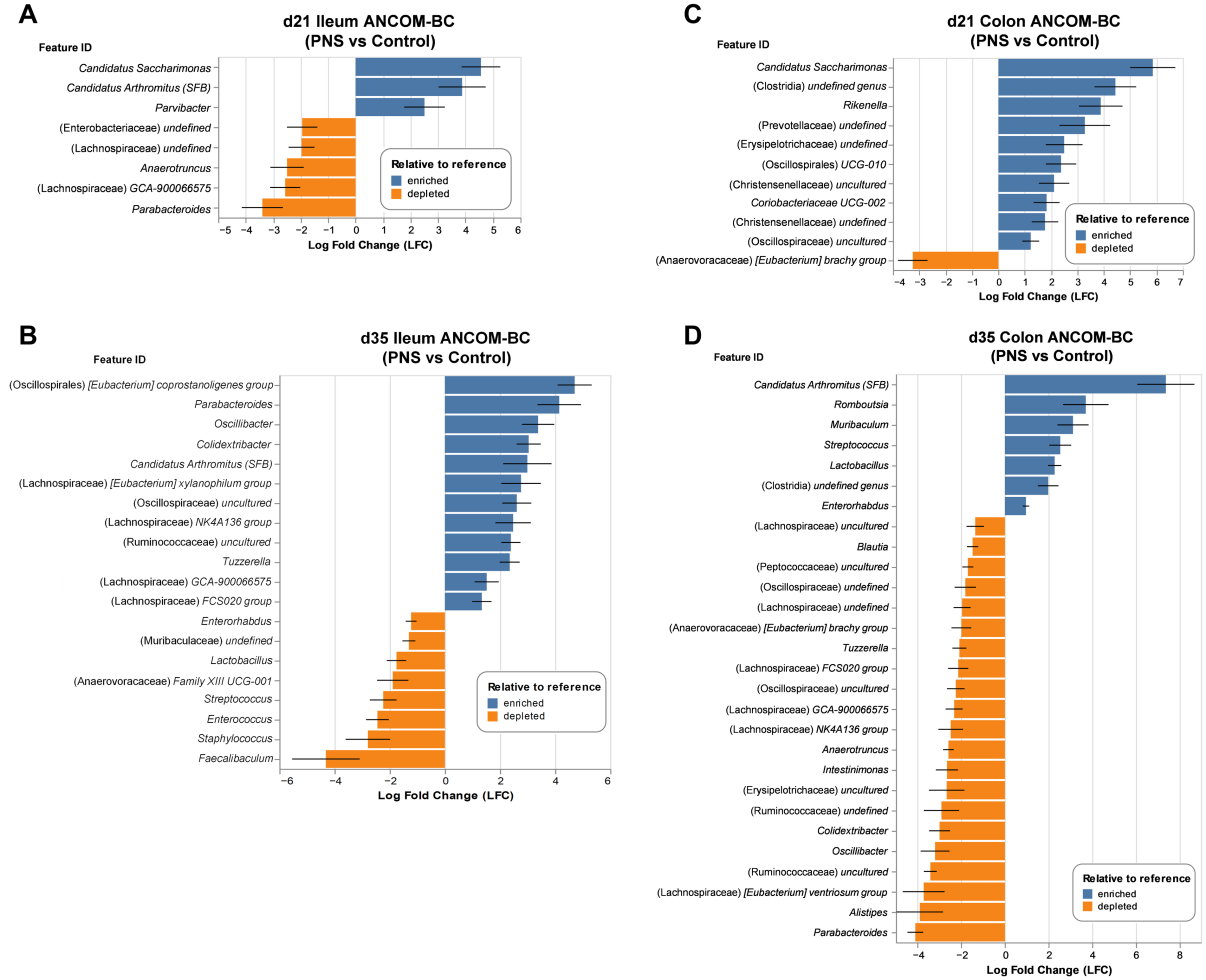
Intestinal differential abundance. Differences in genus level taxa (feature) abundance between control and PNS offspring ileum **(A,B)** and colon contents **(C,D)** were assessed with ANCOM-BC at d21 (*n*cont_rol♀_ = 7, *n*cont_rol♂_ = 4; *n*PNS♀ = 5, *n*PNS♂ = 9) and d35 (*n*cont_rol♀_ = 2, *n*cont_rol♂_ = 6; *n*PNS♀ = 7, *n*PNS♂ = 2). Analyses were conducted in QIIME 2.0 with Holm’s FDR correction. Taxa denoted in bar plots were differentially enriched (blue) or depleted (orange) in PNS offspring relative to control offspring (*q* ≤ 0.05). No differences were detected at d7.

There was a significant enrichment of *Candidatus Saccharimonas, Candidatus Arthromitus*—also known as segmented filamentous bacteria (SFB)—and *Parvibacter* in PNS offspring ileal contents at d21, in addition to a depletion of *Anaerotruncus*, *Parabacteroides*, and incompletely defined genera in Lachnospiraceae and Enterobacteriaceae (Figure 6A). This enrichment of SFB also persisted in d35 PNS ileums. In contrast, several of the genera that were depleted at d21 were found to be enriched in d35 PNS ileums including *Parabacteroides* and genera within Lachnospiraceae. Other enriched taxa included several incompletely defined genera that belonged within Oscillospirales and Ruminococcaceae. Multiple genera were depleted in d35 PNS ileums, including *Lactobacillus*, *Enterorhabdus*, *Muribaculaceae*, *Streptococcus*, and *Faecalibaculum*, as well several other low-level abundance genera (Figure 6B; Supplementary Figure 5). The consistent enrichment of SFB in PNS ileums was of particular interest given this genera’s notable roles in stimulating *Il17a* gene expression which was significantly upregulated in d35 PNS ileums (*p* < 0.001) and correlated with d35 SFB abundance (*p* = 0.003; Supplementary Figure 6).

At d21, PNS offspring colonic contents were enriched with *Candidatus Saccharimonas*, *Rikenella*, *Coriobacteriaceae UCG-002*, and several incompletely defined genera within Clostridia, Prevotellaceae, Erysipelotrichaceae, Oscillospirales, and Christensenellaceae, while *Eubacterium brachy group* was found to be depleted (Figure 6C). At d35, 28 genera were found to be differentially abundant. As in the ileum, a significant enrichment of SFB was observed in d35 PNS colonic contents, in addition to enrichment of *Romboutsia*, *Streptococcus*, and *Lactobacillus* among others. A depletion of *Parabacteroides*, *Alistipes*, and various genera within Lachnospiraceae, Oscillospirales, and Ruminococcaceae was also observed in d35 PNS colons in addition to several other taxa with low overall abundance (Figure 6D; Supplementary Figure 5).

### Differentially abundant bacteria correlate with lung and intestinal gene expression

#### Microbiome DA abundance and gene expression correlations

Spearman correlations were calculated to assess relationships between significant DA microbes and gene expression at corresponding time points. Ileal DA microbes were correlated to both ileal and lung gene expression, whereas colonic DA microbes were correlated with lung gene expression. No correlations were performed for d7 or within the colon due to a lack of DA microbes and gene expression differences, respectively. These analyses were intended to be exploratory, and only significant correlations (*p* ≤ 0.05) with linear trends are discussed (Spearman rho, *p*-values, and correlation matrices are listed in Supplementary Tables 2–7 and Supplementary Figures 7–12).

**Figure 7:**
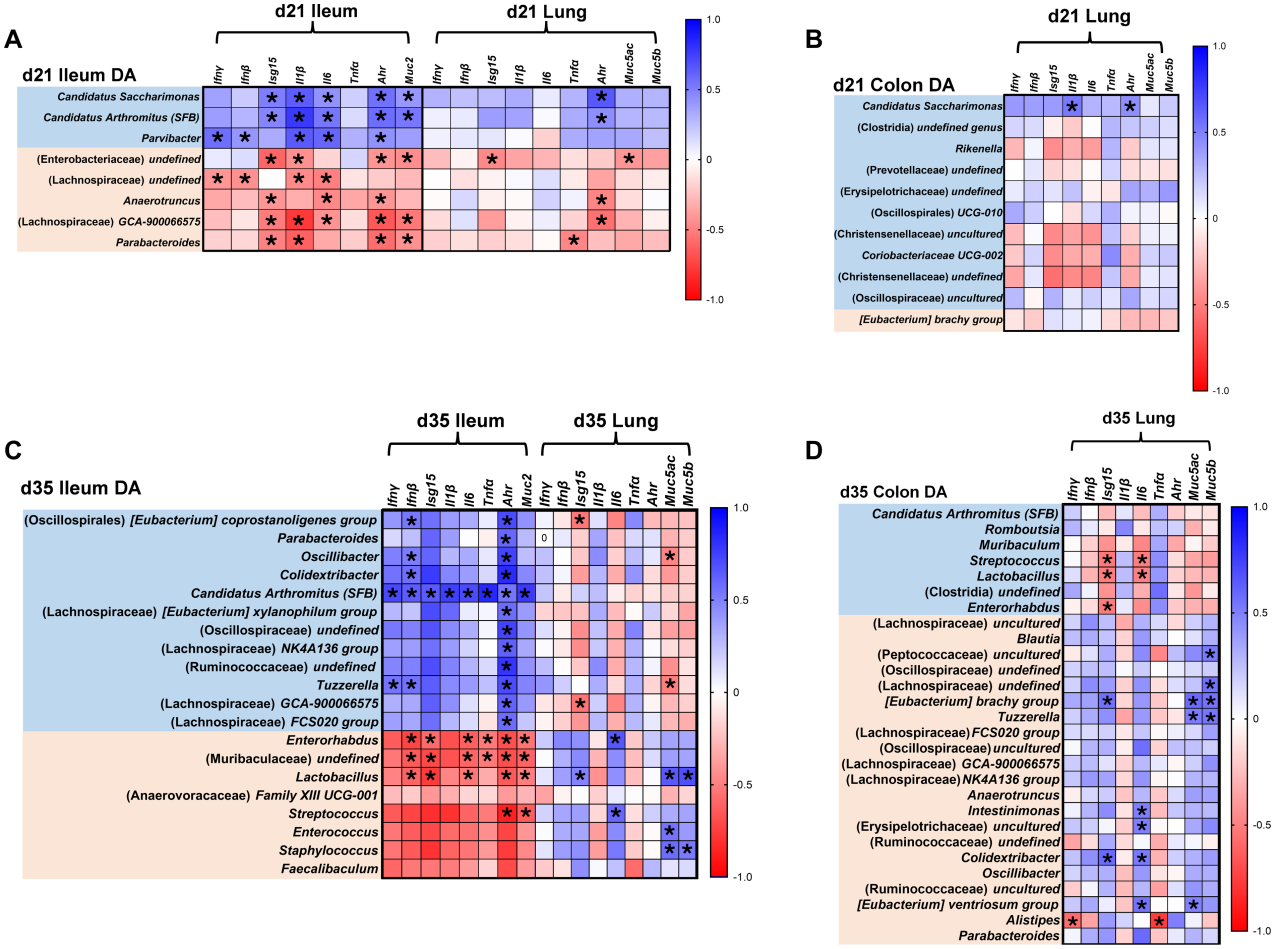
Differentially abundant microbes and gene expression. Spearman correlations were used to assess the association between relative abundance of significant DA microbes and gene expression. Ileal DA microbes at d21 (*n*_control♀_ = 7, *n*_control♂_ = 4; *n*_PNS♀_ = 5, *n*_PNS♂_ = 9) and d35 (*n*_control♀_ = 2, *n*_control♂_ = 6; *n*_PNS♀_ = 7, *n*_PNS♂_ = 2) were correlated to ileal and lung gene expression at respective time points **(A,C)**, and colonic DA microbes at d21 (*n*_control♀_ = 7, *n*_control♂_ = 4; *n*_PNS♀_ = 5, *n*_PNS♂_ = 9) and d35 (*n*_control♀_ = 2, *n*_control♂_ = 6; *n*_PNS♀_ = 7, *n*_PNS♂_ = 2) were correlated to lung gene expression at respective time points **(B,D)**. Center log ratio transformations were performed on bacterial abundances prior to computing correlations to account for the compositional nature of the data. Along the *y*-axis of the correlation matrix, enriched genera are represented in blue and depleted genera are represented in orange. Within the correlation matrix, blue indicates positive associations while red indicates negative associations. *p ≤ 0.05.

At d21, PNS offspring ileums had greater expression of *Isg15*, *Il6*, *Ahr*, and *Muc2* than controls (*p* < 0.05; Figure 2). *Candidatus Saccharimonas* and SFB abundance, enriched in d21 PNS ileums, was positively correlated with expression of all significantly different genes (Figure 7A). *Parvibacter*, enriched in PNS offspring, also had significant correlative trends with d21 ileal *Ifng*, *Ifnb*, *Il6* and *Ahr* expression. All three enriched DA microbes exhibited significant positive correlations with d21 *Il1b* expression, which had a mean relative fold change of 8.52 in PNS offspring versus 2.66 in controls (*p* > 0.05; Figure 2D). Among depleted DA microbes, abundance of Lachnospiraceae *GCA-900066575* and *Parabacteroides* was negatively correlated with d21 ileal *Il1b*, *Ahr*, and *Muc2*, in addition to negative correlations between *Anaerotruncus* and *Ahr*, and Lachnospiraceae (*undefined*) and *Il6* (Figure 7A). This would imply that harboring a reduced proportion of these genera was associated with increased, or inability to regulate, gene expression relative to control offspring.

Correlations were also performed between DA microbes and lung gene expression to examine potential gut-lung-axis interactions. At d21, PNS offspring lungs had heightened expression of *Ifng*, *Isg15*, *Il1b*, *Ahr*, *Muc5ac*, and *Muc5b* (*p* < 0.05; Figure 1). Similar to the ileum, *Ahr* gene expression was found to be positively correlated with *Candidatus Saccharimonas* and *SFB* abundance and negatively correlated with the depletion of *Anaerotruncus* and Lachnospiraceae *GCA-900066575* (Figure 7A). Correlations between colonic d21 DA microbes and lung gene expression revealed a similar positive correlation between enrichment of *Candidatus Saccharimonas* and lung *Ahr*, as well as lung *Il1b* (Figure 7B).

At d35 gene expression was dramatically increased in PNS ileums with heightened expression of all interferon genes, *Il6*, *Tnfa*, *Ahr* and *Muc2* (*p* < 0.05; Figure 1). Enrichment of SFB abundance had strikingly positive, significant correlations to all genes assessed in the ileum. Almost all d35 DA microbes were significantly correlated to ileal *Ahr* expression, with notable positive correlations associated with enrichment of SFB and genera within Lachnospiraceae and Ruminococcaceae and negative correlations with depletion of *Enterorhabdus*, *Muribaculaceae*, and *Lactobacillus* (Figure 7C). Depletion of *Enterorhabdus*, *Muribaculaceae*, and *Lactobacillus* at d35 negatively correlated with expression of d35 ileal *Ifnb*, *Il6*, and *Muc2*. Depletion of ileal *Streptococcus* also negatively correlated with d35 *Ahr* and *Muc2*.

In d35 lungs, PNS offspring exhibited reduced expression of *Ahr*, *Muc5ac*, and *Muc5b* (*p* < 0.05; Figure 1). Positive correlations were detected between depletion of ileal *Lactobacillus* and *Muc5ac*, *Muc5b*, and *Isg15* expression, as well as between depletion of ileal *Enterorhabdus* and *Streptococcus* and d35 lung *Il6* expression (Figure 7C). In contrast to the ileum, *Streptococcus*, *Lactobacillus* and *Enterorhabdus* were enriched in PNS colons at d35 where abundance negatively correlated with lung *Isg15* (all three) and *Il6* (former two), thereby corroborating an association between these microbes and genes in the lung. Lastly, d35 lung *Muc5b* was found to positively correlate with Peptococcaceae and Lachnospiraceae, while *Tnfa* negatively correlated with *Alistipes*, all of which were depleted in PNS colons (Figure 7D). Collectively, correlative analyses provide evidence of a relationship between intestinal microbial composition and the immune dysregulation observed in intestinal and lung tissue of PNS offspring.

#### Microbiome diversity and gene expression correlations

While informative, testing associations between DA microbes and host gene expression narrowly defines the scope of microbial contributions to a subset of total sequencing output. Therefore, to broaden analyses to intestinal microbial composition as a whole, alpha diversity values were correlated to tissue gene expression on d35 (the day at which alpha diversity was significantly different in PNS offspring). Correlations were tested between ileal alpha diversity and gene expression and, to explore possible gut-lung associations, between both ileal and colonic alpha diversity and lung gene expression. In addition to Shannon diversity, Faith’s phylogenetic alpha diversity (Faith PD) was calculated and used for comparisons (see Supplementary Figure 13 for offspring Faith PD across time points). Ileum microbial composition was correlated with the majority of ileal tissue gene expression. Shannon diversity, which was increased in d35 PNS ileums (Figure 4E), had significant positive associations with ileal *Isg15* and *Ahr* expression (*p* < 0.05; Supplementary Figure 14A). Ileal Faith PD, also increased in d35 PNS ileums (Supplementary Figure 13E), significantly correlated with all ileal genes except *Tnfa* (*p* = 0.06) and *Muc2* (*p* = 0.06; Supplementary Figure 14A). Ileal Shannon diversity was negatively associated with lung *Isg15* and positively association with lung *Tnfa* (*p* < 0.05) at d35, but no significant associations were noted for ileal Faith PD. In contrast, no correlations were observed between d35 colonic Shannon diversity and lung gene expression, but colonic Faith PD was positively correlated with lung *Isg15* and *Il6* and negatively correlated with lung *Tnfa* (*p* < 0.05; Supplementary Figure 14B). Together, these findings suggest that PNS-induced changes in the microbiome are linked to early life abnormalities in intestinal and lung immune responses in exposed offspring.

### *MyD88^−/−^* offspring gene expression

**Figure 8:**
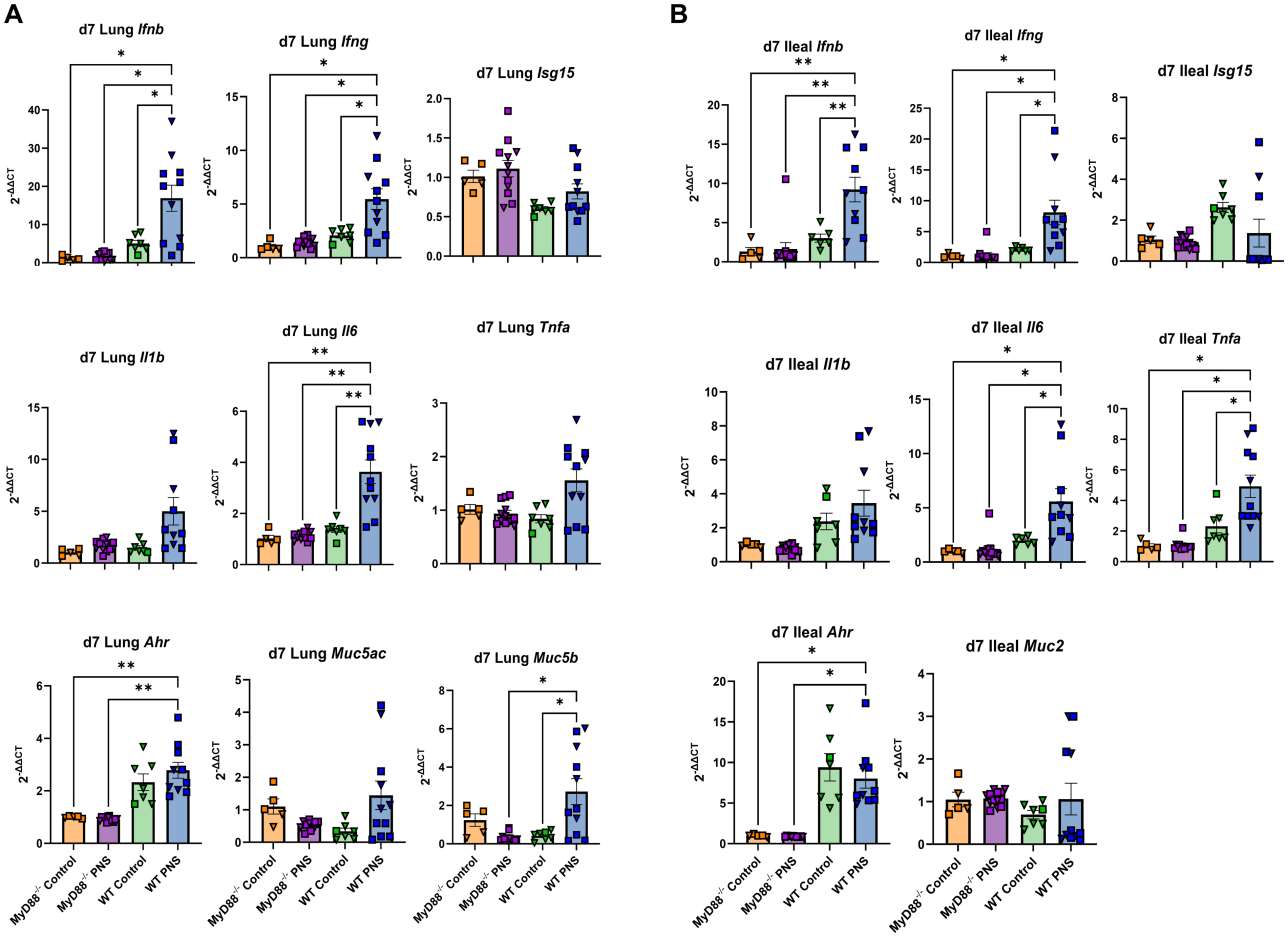
*MyD88*^−/−^ vs. C57BL/6 WT d7 tissue gene expression. Interferon, classical proinflammatory, aryl hydrocarbon receptor, and mucin gene expression in lung tissue **(A)** and ileum tissue **(B)** was assessed in control and PNS *MyD88*^−/−^ and C57BL/6 offspring at d7. Values are expressed using the 2^-ΔΔCt^ method relative to *MyD88*^−/−^ control values. *Myd88*^−/−^ control values are denoted by orange bars (*n♀* = 2, *n♂* = 3), *Myd88*^−/−^ PNS values are denoted by purple bars (*n♀* = 6, *n♂* = 7), WT control values are denoted by green bars (*n♀* = 6, *n♂* = 1), and WT PNS values are denoted by purple bars (*n♀* = 4, *n♂* = 7); females are denoted by triangles and males are denoted by squares. Missing values are the result of outlier removal or due to samples with expression that was below qPCR detection limits. Bar graphs denote mean ± SEM. Planned contrasts were implemented on linear mixed models, and significant comparisons relative to PNS WT offspring are denoted on each respective graph. *p ≤ 0.05, **p ≤ 0.01, ***p ≤ 0.001, ****p ≤ 0.0001.

To investigate the role of microbial signaling on PNS-mediated cytokine expression, homozygous MyD88 knockout (*MyD88^−/−^*) breeders were used to generate control and PNS *MyD88^−/−^* offspring for comparative gene expression quantification relative to C57BL/6 (wild type; WT) offspring. Analyses in *MyD88*^−/−^ offspring were conducted on d7 at a time aligned with increased immune gene expression in WT PNS offspring and in alignment with report of early-life respiratory infection in PNS-exposed children. Strikingly, there were no differences in expression of any of the interferon, proinflammatory, *Ahr*, or mucin genes in the lungs or ileums of *MyD88^−/−^* control versus *MyD88^−/−^* PNS offspring (Figure 8; Supplementary File 5). Comparatively, expression of lung and ileal *Ifnb*, *Ifng*, *Il6*, and *Ahr* was significantly higher in WT PNS offspring relative to both groups of *MyD88^−/−^* offspring (*p* < 0.05; Figures 8A,B). No differences in lung or ileal tissue gene expression were observed between WT controls and either group of *MyD88^−/−^* offspring with the exception of *Ahr* (Supplementary File 5) showing that the knockout genotype did not have significant differences in baseline gene expression of the targets assessed. No differences due to PNS exposure or between genotypes were observed for lung or ileal *Isg15* and *Il1b* (Figures 8A,B) in agreement with findings in WT mice at d7 (Figures 1–3). Lung *Muc5b* expression was greater in WT PNS relative to *MyD88^−/−^* PNS offspring (*p* < 0.001), but no differences were detected for lung *Muc5ac* (Figure 8A) or ileal *Muc2* (Figure 8B). Interestingly, ileal *Tnfa* was increased in PNS WT relative to *MyD88^−/−^* offspring (Figure 8B), but the same trend did not reach significance in the lung (Figure 8A). However, relative expression of *Tnfa* was approximately 2-fold higher in the ileums compared to the lungs of WT mice at d7 (Figures 1F, 2F), and microbial density is far greater in the intestine than the respiratory tract. Therefore, it is possible that the role of MyD88 signaling was better captured in the ileum. Collectively, our knockout study provides evidence that MyD88 signaling, a key intermediary of microbial-mediated TLR stimulation and downstream immune responses, could play an important role in PNS-mediated skewing of proinflammatory and interferon gene signatures in the ileum, and excitingly, in the lung.

## Discussion

Fetal exposure to chronic maternal anxiety, depression, or adversity during pregnancy is recognized as a prominent driver of deleterious postnatal mental and physical health outcomes in children (Kim et al., 2015; Stepanikova et al., 2019; Bush et al., 2021). During this susceptible stage of development, placental transfer of increased levels of maternally-derived glucocorticoids, inflammatory mediators, neurotransmitters, and microbes or microbial products can shape the developing fetus through fetal programming (Rakers et al., 2020). While the link between PNS exposure and childhood disease risk is not completely defined, a significant body of literature (reviewed elsewhere (Jašarević et al., 2014; O’Mahony et al., 2017; Collins et al., 2024; Kimmel et al., 2024)) has investigated potential mechanisms through which PNS contributes to childhood disorders such as anxiety, depression, schizophrenia, ADHD, and autism. In addition to mechanisms such as enhanced HPA responsiveness (McGowan and Matthews, 2018) and fetal neuroinflammation (Chen et al., 2020), PNS-mediated changes in the maternal and offspring microbiome are recurrently emphasized as a key influencer of these pathologies in the postnatal period (Jašarević et al., 2017, 2018; Chen et al., 2022; Ahrens et al., 2024). While neurodevelopment and mental health outcomes have been the focus of multiple studies, fewer studies have focused on the importance of PNS-driven shifts in offspring microbiomes in the context of immune development and infectious disease susceptibility. This gap is particularly pronounced for respiratory infections, where PNS offspring frequently exhibit heightened vulnerability (Nielsen et al., 2011; Lee et al., 2014; Phelan et al., 2015; Stepanikova et al., 2019; Lau et al., 2022; Orr et al., 2024), but whether the microbiome is associated with this heightened vulnerability has not been systematically studied. Here, we used 16S rRNA sequencing and tissue gene expression in offspring exposed to PNS as a preliminary characterization of differences in baseline cytokine profiles and microbiome composition that may be related to disease susceptibility in this population. Our results show that PNS-mediated changes in the offspring intestinal microbiome correlate to long-lasting lung and intestinal cytokine expression, and that MyD88 signaling contributes to increased cytokine expression in PNS-exposed offspring.

In our study, PNS offspring had significant increases in interferon (*Ifng*, *Ifnb*, *Isg15*) and proinflammatory (*Il1b*, *Il6*, and *Tnfa*) gene expression primarily in the lung and ileum, with less effect in the colon. These effects were most pronounced in the lung at d7 and d21 and in the ileum at d21 and d35, in agreement with reports in mice of windows of time where microbial exposure is particularly critical for immune development in these tissues (Gollwitzer et al., 2014; Al Nabhani et al., 2019). Furthermore, these observations agree with the timing of heighted disease risk in PNS-exposed children, where susceptibility to respiratory diseases is highest earlier in development (Nielsen et al., 2011; Henriksen and Thuen, 2015; Stepanikova et al., 2019; Robinson et al., 2021). Offspring in the present study underwent weaning at d21, a notoriously stressful period that induces a vigorous immune response largely driven by microbiome expansion in response to significant dietary changes. Interestingly, we observed a 2- to 4-fold increase in d21 (weaning age) vs. d0 ileal interferon and *Tnfa* gene expression across offspring but found that PNS ileums maintained heightened levels of proinflammatory and interferon gene expression through d35 (*p* < 0.05). This could suggest an exaggerated or dysregulated prolonged response to weaning stress as pups were not exposed to any postanal stressors or immune challenges. The dysregulation in the intestine post-weaning could also lend to reports of increased severity of experimentally induced colitis in adult mice exposed to prenatal stress (Sun et al., 2021). In line with these findings, Al Nabhani et al. (2019) reported a transient increase in ileal *Tnfa* and *Ifng* expression around weaning age in healthy WT—but not germ-free, antibiotic-treated, or MyD88- and Trif-deficient mice. Al Nabhani et al. (2019) also found that lack of or disruption of the microbiome during the weaning period was associated with increased susceptibility to inflammatory pathology during colitis, emphasizing microbial composition and immunomodulatory microbial metabolites during weaning as a critical determinant in this phenotype. Of note, PNS offspring in the present study exhibited shifts in abundance of genera within Lachnospiraceae, Ruminococcaceae, and Faecalibaculum—bacterial families comprised of prolific short-chain fatty acid (SCFA) producers (Singh et al., 2023)—as well as prolonged enrichment of immunomodulatory bacteria such as SFB and others that correlated with inflammatory gene expression at d21 and d35. It is possible that these changes in commensal composition led to an imbalance between beneficial microbial metabolites and immune cell function that exaggerated immune responses in PNS ileums at d35. In addition to warranting investigation into effects of PNS exposure on regulatory immune cells, these data support the hypothesis that imbalances in early-life commensal composition observed in PNS offspring may be acting as important drivers of dysregulation of immune homeostasis.

It is well-known that pioneer colonization, or initial seeding, of the intestinal microbiome at birth has significant ramifications on early-life immune development (Gensollen et al., 2016; Reynolds and Bettini, 2023). Although we observed the most prominent changes in PNS offspring microbiomes at d21 and d35, modest changes in d7 alpha diversity were noted. We hypothesize that we were unable to fully capture appreciable microbiome differences at d7 due to overall low biomass and low diversity in the first week of life when all offspring showed an overwhelming predominance of *Lactobacillus* (Supplementary Figure 16), compounded by limited taxonomic resolution associated with sequencing 16S rRNA hypervariable region V4 as opposed to full-length or multiple region sequencing (Katiraei et al., 2022; Zhang et al., 2024) where such higher powered techniques have been more successful in detecting early-life differences (Zijlmans et al., 2015; Galley et al., 2023). However, not only do our data indicate that PNS significantly impacts early-life intestinal microbial composition as early as d7 (alpha diversity) and through d35 (both alpha and beta diversity), but results from our *MyD88*^−/−^ model are consistent with the hypothesis that signaling via pattern recognition receptors may play a role in the development of abnormal immune gene signatures in PNS offspring. Microbial stimulation of the immune system is largely coordinated through TLR detection of microbial ligands, downstream signaling via MyD88, and activation of NF-κB and MAPK signaling cascades that elicit production of proinflammatory cytokines and other immune effectors (Akira et al., 2006). Consequently, MyD88 plays a pivotal role in translating microbially-derived signals into appropriate immune responses relevant to immune maturation and homeostasis and has been targeted as a means to investigate microbial contributions in a variety of contexts (Watanabe et al., 2020; Hsiao et al., 2024; Knezović et al., 2024; Li J. et al., 2024). Here we showed that offspring lacking a functional MyD88 signaling pathway (*MyD88^−/−^*), and therefore unable to appropriately respond to the microbiome, did not exhibit changes in early-life lung or ileal proinflammatory and interferon gene expression as a result of PNS exposure. We hypothesize that differences in offspring microbial composition, and subsequent immunomodulatory microbial signaling via MyD88, could contribute to the divergence in baseline immune gene expression observed in WT prenatally stressed offspring. Indeed, Jašarević et al. (2018) previously implicated a role of the maternal microbiome in the transcriptomic enrichment of MyD88, *Tnfa*, and interferon-response gene sets in the fetal intestine after PNS exposure, which could in turn contribute to exaggerated postnatal immune responses and altered reactivity toward abnormalities in PNS offspring microbiomes. While our knockout model does not consider the impact of alternative pathways affected by the *MyD88*^−/−^ genotype or later offspring time points, similarities between knockout offspring and WT controls at d7 suggest minor confounding effects.

A variety of bacterial taxa were DA in PNS offspring, including SFB, Lachnospiraceae, and *Lactobacillus*, which have known immunomodulatory roles (Wells, 2011; Flannigan and Denning, 2018; Vacca et al., 2020). Enrichment of SFB was detected in d21 PNS ileums with persistent enrichment throughout the intestine at d35. Unlike most commensals, SFB can directly adhere to intestinal epithelial cells and are most known for their roles in T helper (T_h_)_17_ cell differentiation, *Il17a* induction, and stimulation of mucosal immunity (Lécuyer et al., 2014; Flannigan and Denning, 2018). Accordingly, we found that SFB abundance was correlated with increased *Il17a* expression in the ileum but not the colon, perhaps due to differences in gut-associated lymphoid tissue localization (Jung et al., 2010). The importance of this increased *Il17a* expression is not yet known, but dysregulation of IL-17a production has been reported to promote inflammatory pathologies (Mills, 2023) and has been linked to neurodevelopmental abnormalities in offspring exposed to prenatal maternal immune activation, another form of PNS (Kim et al., 2017).

The Lachnospiraceae family and *Lactobacillus* genus have also been found to be dysregulated in human and rodent offspring exposed to PNS (Jašarević et al., 2017; Chen et al., 2022; Galley et al., 2023; Rojas et al., 2023). At d35 in the present study, Lachnospiraceae genera were enriched in the ileum and depleted in the colon, whereas *Lactobacillus* was depleted in the ileum and enriched in the colon. These bacteria are obligate and facultative anaerobes, respectively, and normally would accordingly reside in the small intestine or colon. Redistribution along the gut could potentially have resulted in a loss or change of beneficial functions specific to these bacteria in their typical resident sites (e.g., inflammatory signaling, SCFA production, influences on gut barrier integrity (Wells, 2011; Vacca et al., 2020)) and contributed to an imbalance in intestinal immune homeostasis in line with correlations between these bacteria and inflammatory gene expression reported here.

Interestingly, select bacteria that were DA in PNS offspring ileums and colons correlated with immune gene expression in the lungs. At d21, for example, SFB, Lachnospiraceae (*GCA-*900066575), and *Candidatus Saccharimonas* correlated with lung *Ahr* expression which increased in PNS offspring (*p* < 0.05). The aryl hydrocarbon receptor is a transcription factor that possesses a diverse array of immunomodulatory roles and ligands, including microbially-produced tryptophan derivatives (Lamas et al., 2018). However, none of the correlated microbes reportedly metabolize tryptophan, thus it is unlikely that the bacteria-*Ahr* correlations are due to bacterially produced tryptophan metabolites. However, *Ahr* expression is known to be upregulated during inflammation (Qiu et al., 2013). Therefore, bacterial correlations with lung *Ahr* expression may be secondary to inflammatory feedback across the gut-lung-axis. At d35, depletion of *Lactobacillus* and Lachnospiraceae (*undefined*) were correlated with expression of lung mucins which were reduced in PNS offspring (*p* < 0.05). Accordingly, both bacteria have reported roles in maintenance of the mucus barrier within the gut (Ouwerkerk et al., 2013; Zha et al., 2023).

Correlations between DA microbes and lung gene expression may not be surprising, since intestinal bacteria are known to influence immunity in the intestine itself, but also in the lungs. For example, reductions in SCFA-producing genera such as *Bifidobacterium*, *Lactobacillus*, and *Faecalibacterium*—which were dysregulated in PNS offspring here and in other reports (Galley et al., 2023; Kimmel et al., 2024)— have been associated with increased risk of childhood asthma (Fujimura et al., 2016), which also develops with increased prevalence in children exposed to PNS (Flanigan et al., 2018). Intestinal microbe-derived metabolites, including the SCFA, can also enter systemic circulation and facilitate gut-lung cross talk by modulating immune responses in the lung (Dang and Marsland, 2019), as well as confer protection against viral respiratory infections including rhinovirus (Antunes et al., 2023), RSV (Antunes et al., 2019), and influenza (Trompette et al., 2018). Clinical trials further substantiate gut-lung axis modulation, with oral probiotic administration (e.g., *Lactobacillus* spp. and *Bifidobacterium* spp.) reducing respiratory infection rates by up to 27% (Hatakka et al., 2001; Hojsak et al., 2010; Taipale et al., 2011; Maldonado et al., 2012) and improving asthma-related outcomes in children (Ciprandi and Tosca, 2022). Future studies should assess metabolites in the intestine and lungs of PNS offspring, rather than DA microbe abundance alone, to provide additional insights into PNS microbiome contributions to respiratory health. Future studies should also assess the resident respiratory microbiome, which can locally modulate immunity in the lung (Li R. et al., 2024) and may impact the effects of PNS on lung immunity. Nevertheless, the absence of PNS-mediated changes in lung gene expression in *MyD88^−/−^* pups is consistent with a potential MyD88-dependent pathway and plausible role of bacterial signaling, whether derived from intestinal or respiratory bacteria, in this phenotype.

On a global scale, lower respiratory infections are one of the most common contributors of mortality in children under five (United Nations Inter-Agency Group for Child Mortality Estimation (UN IGME), 2025). Due to immaturity of adaptive immunity, children are particularly prone to recurrent infections early in life (El-Azami-El-Idrissi et al., 2016). Moreover, there is substantial variability in the manifestation and severity of these respiratory infections (Mejias et al., 2013; Mettelman and Thomas, 2021), but factors contributing to this variability are poorly understood. We hypothesize that PNS is one such factor modulating respiratory infection outcomes due to reports of increased susceptibility in exposed offspring. Among other infections, PNS has been found to increase the risk of acquiring bronchiolitis (Lee et al., 2014; Phelan et al., 2015; Orr et al., 2024), one of the most common causes of respiratory hospitalizations in children under the age of 2 and a common sequela of RSV infection (Koehoorn et al., 2008). Interestingly, heightened levels of interferon and innate proinflammatory mediators, as noted here in PNS offspring, have been associated with increased severity of respiratory diseases, including asthma (Rowe et al., 2004; Naumov et al., 2019) and RSV (Mejias et al., 2013). Moreover, longitudinal reports in PNS-exposed children have reported sex differences associated with infection risk and inflammatory responses (Rosa et al., 2016; Brunwasser et al., 2019; Robinson et al., 2021). Although statistical power to detect sex differences was limited in the present study, we did observe higher lung *Il1b*, *Il17a* and ileal *Ifng* in female relative to male PNS offspring (Supplementary Figure 1; Supplementary Files 1, 2). Further investigation is needed to determine the extent to which these observations translate to sex-specific protective or deleterious immunity during infectious challenge. Nevertheless, our findings present evidence that PNS exposure leads to changes in baseline offspring immune-related gene expression and microbiome composition in the absence of postnatal immune-activating challenge. Given the combination of our findings and published evidence of PNS exposure on respiratory disease risk, future iterations of this work are expected to incorporate a disease challenge (e.g., asthma or influenza) for continued investigation of the early-life health implications of PNS exposure in addition to characterization of immune cell populations and quantification of cytokine protein levels to further support gene expression findings. These future studies will also need to consider several limitations left unaddressed in the current work, including characterization of the respiratory microbiome, as well as characterization of the microbiome and gene expression at later time points in *MyD88*^−/−^ mice exposed to PNS.

In summary, we have demonstrated that offspring exposed to PNS exhibit dysregulated immune responses in the ileum and lung that are perpetuated over the first five weeks of life. In line with longitudinal observations in PNS-exposed children and subsequent disease risk, age appears to be an important factor in our model as peak differences in the lung were most prominent earlier in life in contrast to post-weaning in the ileum, possibly due to age-dependent maturation of the microbiome and immune system in these tissues. Further mechanistic investigation into the tissue-specific age-dependent influences of PNS and associated health risks in early versus later life is warranted in our future work and could help define optimal time points of treatment intervention. Intestinal microbiome composition and diversity, which are altered in PNS offspring, are correlated with these immunologic abnormalities, and MyD88 signaling appears to play an important role in the observed outcomes at baseline. The *MyD88*^−/−^ model is widely used as a proxy for microbial-driven signaling and subsequent immune responses throughout the literature and provides a compelling rationale for future studies to focus on microbiota-derived antigen stimulation of the immune system as a mechanistic underpinning for the observed PNS offspring phenotype.

## Materials and methods

### Animal housing and handling

Adult (6- to 8-week-old) nulliparous female and male C57BL/6 mice were obtained from Charles River (Wilmington, MA) and were left undisturbed for 1 week to permit acclimation. All mice were provided *ad libitum* access to a standard chow diet and autoclaved water. Females were paired with males (1:1) for overnight for mating and separated 24 h later to ensure uniform time of conception across all pregnancies. At the time of separation, designated as gestational day (GD)0, females were weighed and singly housed to control for coprophagic effects on subsequent microbiome analyses. Pregnancy was later confirmed by weight gain of approximately 2.5 g between GD0 and GD7. On GD10 dams were randomly assigned to either PNS or control groups. From GD10-GD16 PNS dams were restrained in perforated, autoclaved, 50 mL conical tubes for 2 h from 0900 and 1,100, while control dams were left undisturbed. This model of restraint stress has been previously shown to increase maternal serum corticosterone, disrupt the maternal microbiota and result in altered microbiota composition of prenatally stressed offspring relative to control counterparts (Gur et al., 2017, 2019; Galley et al., 2021; Chen et al., 2022). Body weights and fecal samples were collected from the dams at GD10 (pre-PNS) and GD16 (post-PNS) to assess changes in weight gain and the maternal microbiota, respectively (Supplementary Figure 15). Following the PNS period, cages were checked twice daily for pups, but dams remained otherwise undisturbed until parturition. Pups remained with dams until weaning at d21 at which time they were cohoused with same-sex littermates for the remainder of the study. Two replicate experiments were performed that, combined, consisted of 11 dams (n_control dams_ = 5 control and n_PNS dams_ = 6) resulting in a total of 36 control offspring and 46 PNS offspring. Experiments were conducted in accordance and with approval of the Institutional Animal Care and Use Committee at Nationwide Children’s Hospital (Protocol #AR16-00050).

### Sample collection

At d0 (parturition), d7, d21, and d35, a subset of offspring from each litter were euthanized by CO_2_ inhalation for sample collection. All samples were aseptically collected in a biosafety cabinet and snap frozen. Once the peritoneal cavity was exposed, a new set of sterile tools was used to collect intestinal samples for each mouse to avoid contamination of microbiome samples. At d0, whole gut and lung tissue were collected. At d7, d21, and d35, lung, ileal, and colonic tissues and intestinal contents were collected. All tissues were used for quantitative real-time (q)PCR analysis and intestinal contents were used for 16S rRNA sequencing. No 16S rRNA sequencing was performed at d0 due to poor quality library amplification attributable to the relative sterility of the gut immediately after birth. Offspring sample sizes for each time point are as follows: d0 (n_control_ = 10; n_PNS_ = 12), d7 (n_control_ = 7; n_PNS_ = 11), d21 (n_control_ = 11; n_PNS_ = 14), d35 (n_control_ = 8; n_PNS_ = 9).

### MyD88 knockout experiment

In a follow-up experiment, adult (6- to 8-week-old) nulliparous female and male *MyD88^−/−^* mice were obtained from The Jackson Laboratory (Bar Harbor, ME). Mice underwent the same breeding, PNS exposure, and husbandry practices described above and resulted in 2 litters of *MyD88^−/−^* control pups (*n* = 5) and 2 litters of *MyD88^−/−^* PNS pups (*n* = 13). It is standard practice to supplement MyD88^−/−^ colonies with antibiotics, but due to our interest in the role of the microbiome, no antibiotics were supplemented to any mice over the course of these experiments. All pups were sacrificed at d7 for collection of lung and ileum tissues for subsequent qPCR analyses as described above.

### RNA extraction and quantitative real-time PCR

Total RNA was extracted from lung, ileum, and colon tissues using Trizol Reagent (Invitrogen, Carlsbad, CA). After extraction, RNA quantity (ng/μL) and quality (A_260_/A_280_) were verified using the Take3 application “Nucleic Acid Quantification” within the Gen5 software on the Synergy HTX Multi-mode Microplate Reader (Agilent BioTek, Santa Clara, CA), and cDNA was synthesized using the High-Capacity cDNA Reverse Transcription Kit (Applied Biosystems, Foster City, CA) according to manufacturer’s instructions. All qPCR reactions were performed using the Power SYBR^®^ Green PCR Master Mix protocol (Applied Biosystems, Foster City, CA) and QuantStudio™ 5 Real-Time PCR Systems machine (Applied Biosystems) and included a non-template control well for all targets tested. Primers are listed in Supplementary Table 8. All C57BL/6 (WT) offspring qPCR data are expressed using the 2^-ΔΔCt^ method relative to control values at baseline to highlight how exposure to chronic prenatal stress altered gene expression over time. For ileum and colon gene expression, d0 gene expression corresponds with whole gut tissue due to challenges in sectioning and fragility of the d0 gut. For d7 *MyD88^−/−^* gene expression analyses, data are expressed using the 2^-ΔΔCt^ method relative to *MyD88^−/−^* control values, where d7 WT ΔΔCT and subsequent 2^-ΔΔCt^ values were recalculated accordingly. Gene expression was assessed using single-well measurements per target with high biological replication within treatment.

### DNA extraction

Aseptically collected dam fecal samples and offspring ileal and colonic contents were subjected to DNA extraction with the QIAamp DNA Mini Kit (Cat. No. 51306; Qiagen, Hilden, Germany) per manufacturer’s instructions with slight modifications to improve bacterial cell lysis. Samples were incubated for 45 min at 37 °C in lysozyme-mutanolysin buffer (pH 8.0) containing 22 mg/mL lysozyme, 0.1 U/mL mutanolysin, 20 mM TrisHCL, 1.2% Triton-x (Sigma Aldrich, St. Louis, MO), and 2 mM EDTA (Thermo Fisher Scientific, Waltham, MA), followed by homogenization for 150 s with 0.1 mm zirconia beads on the Mini-Beadbeater-16 (BioSpec Products Inc., Bartlesville, OK). After bead beating, samples were incubated at 95 °C for 5 min with InhibitEx Buffer (Qiagen), then incubated at 70 °C for 10 min with Proteinase K and Buffer AL. The QIAamp DNA Mini Kit isolation protocol was then followed beginning with the ethanol step. DNA was quantified with the Qubit 2.0 Fluorometer (Life Technologies, Carlsbad, CA) using the dsDNA Broad Range Assay Kit (Life Technologies). A non-template control (water blank) was also extracted alongside all samples to serve as a negative control. All DNA extracts were stored at −80 °C prior to 16S rRNA library preparation and sequencing.

### 16S rRNA library preparation and gene sequencing

For initial library preparation, DNA underwent PCR amplification of the 16S rRNA V4 hypervariable region using primers 515F (Parada et al., 2016) and 806R (Apprill et al., 2015) and the following reaction mixture: 12.5 μL Q5 High Fidelity 2X Master Mix (New England Biolabs, Ipswich, MA), 1.25 μL forward primer, 1.25 μL reverse primer, 9 μL of H_2_O, and 2 μL of DNA template. Reaction templates included intestinal content DNA, the extracted non-template DNA control, as well as PCR negative and positive controls. Amplification conditions consisted of 98 °C for 2 m for initial denaturation, 20 cycles of 98 °C for 20 s, 50 °C for 30 s, and 72 °C for 1 m, and a final extension at 72 °C for 2 m. Products were verified on a 0.8% TAE gel and cleaned using the Mag-Bind^®^ TotalPure NGS Kit (Omega Bio-Tek, Norcross, GA) according to manufacturer’s instructions. A second PCR amplification was performed on the bead-cleaned amplicons using the Nextera XT Index v2 Kit (Illumina, San Diego, CA) for tagmentation and generation of uniquely indexed samples according to manufacturer’s recommendations. Products from the second PCR were verified on a 0.8% TAE gel and quantified with the Quanti-iT PicoGreen dsDNA assay kit (Invitrogen, Waltham, MA). A finalized library was created by pooling 300 pg. of each Nextera PCR amplicon into a sterile 1.5 mL tube to facilitate similar coverage of all samples, and the pooled library was cleaned using the Mag-Bind^®^ TotalPure NGS Kit described above. The initial PCR reaction revealed that d7 ileum content DNA samples had low bacterial biomass, therefore, an independent pooled library was created for these samples. This was accomplished with a minor modification to the library preparation protocol in which 10 μL of DNA template was used (rather than 2 μL) in the initial 515F-806R PCR reaction. Subsequent library preparation steps were conducted as described above. The finalized pooled libraries were submitted to the Genomic Services Core at the Institute for Genomic Medicine at Nationwide Children’s Hospital (Columbus, OH) for high-throughput Illumina MiSeq (300 bp paired end) sequencing.

### 16S rRNA sequencing data analysis

All sequences were analyzed with Quantitative Insights Into Microbial Ecology (QIIME) 2.0 v2023.2 (Bolyen et al., 2019), and DADA2 (Callahan et al., 2016) was used for downstream amplicon processing, denoising, and quality control via the QIIME 2.0 dada2 plugin. To achieve an average quality score of at least 20, sequences were truncated from the 3′ end to 222 nt (forward reads) and 140 nt (reverse reads), and the first 20 nt were trimmed from the 5′ end of both forward and reverse reads. Sequences that did not meet quality control criteria were discarded. Taxonomy was assigned using a trained classifier constructed from the SILVAv138.99 ribosomal RNA database (Quast et al., 2013; Bokulich et al., 2018) via the classify-sklearn plugin, and features (DADA2-classified ASVs) denoted as “Eukaryota,” “Unassigned,” “Chloroplast,” “Mitochondria,” not annotated beyond the phylum level, and not present in at least 2 samples were filtered from the dataset. Beyond this point of processing, ileal, colonic, and dam datasets were independently analyzed. For diversity metrics, sequencing depth was determined respective to the dataset based on rarefaction curves (dams: 77,149; offspring ileum contents: 76,962; offspring colon contents: 71,115), and sequences with fewer reads were omitted from diversity analyses. Compositional diversity was assessed by following the QIIME 2.0 core-metrics-phylogenetic plugin. Shannon’s diversity (Shannon, 1948) was calculated to quantify alpha diversity, and unweighted UniFrac (Lozupone and Knight, 2005) distance matrices were used to assess beta diversity in offspring. As different individuals were collected at each time point, no longitudinal testing was performed. For differential abundance analyses, datasets were conditionally filtered to retain features with an abundance of at least (1/rarefaction depth) in at least 10% of samples to minimize noise and discard sequences that would likely be underpowered. Individual pup relative abundances for all characterized genera are presented in Supplementary Figures 16–21.

### Statistical analysis

Gene expression data were analyzed in R (v4.5.0) via linear mixed-effects models using the lme4 package (Bates et al., 2015). For each gene assessed in WT mice, expression was modeled as a function of treatment (control or PNS), time point (d0, d7, d21, d35), and their interaction, with litter included as a random intercept to account for non-independence of littermates. The significance of fixed effects was analyzed by type III ANOVA with Kenward-Roger-adjusted degrees of freedom via the lmerTest package (Kuznetsova et al., 2017). Where interactions were significant, model-based estimated marginal means were computed for the treatment × time combination and post-hoc contrasts were performed to assess treatment effects at individual time points via the emmeans package (Lenth and Piaskowski, 2025). In cases where treatment and time point interactions were not significant, the same full model was retained to maintain consistent variance estimation across analyses, and estimated marginal means were specified for treatment conditional on time point, with resulting contrasts interpreted as conditional model-based estimates rather than evidence of time-specific treatment effects. Sex effects were similarly modeled using fixed effects of treatment, time point, sex, and their interaction and are reported where significant 3-way interactions were detected. For analysis of WT versus MyD88^−/−^ d7 gene expression, linear mixed-effects models including treatment, genotype, and their interaction were fit for each gene with litter as a random intercept. Planned contrasts, specified *a priori*, were used to (1) compare treatment response between WT and knockout mice and compare gene expression relative to PNS WT (Figures 2, 8) assess baseline genotype differences under control conditions. All instances of multiple testing in gene expression analyses were controlled using Benjamini-Hochberg FDR correction. For all qPCR gene expression analyses, values that were outside the range of treatment mean ± 2 standard deviations on a per gene, per time point, per tissue basis, were treated as outliers and excluded from analysis. Outliers, if detected, were ≤ 1 sample/treatment group per gene per time point per tissue. Non-detect values were excluded from analysis. Microbiome diversity statistics were computed in QIIME 2.0 via respective plugins. Beta diversity was analyzed by permutational multivariate analysis of variance (PERMANOVA) with 999 randomizations of the data via the beta-group-significance plugin at the respective time points, and alpha diversity was analyzed by Kruskal-Wallis tests via the alpha-group-significance plugin at the respective time points. Effects of litter-to-litter variation on microbiome diversity were also investigated in R (v4.5.0). For alpha diversity, the treatment by time interaction was assessed with litter coded as a random effect via a linear mixed-effects model, while for beta diversity (distance matrix data) adonis2 via the vegan package (Oksanen et al., 2026), which allows for multi-factor analysis when running PERMANOVA, was used to assess the treatment by time interaction while also using the “permute how()” function of the permute package (Simpson, 2026) to control for litter (Supplementary File 4). Differential abundance was assessed by ANCOM-BC (Lin and Peddada, 2020) using the QIIME 2.0 composition plugin. For 16S analyses, differences in diversity and DA analyses were considered significant where Holm’s FDR-adjusted *p*-values (*q*-values) ≤ 0.05. Prior to correlation analyses between DA microbes and gene expression, DA microbe feature counts were center log ratio (CLR) transformed in R (v4.5.0) using the compositions package(v2.0–5) (van den Boogaart et al., 2023) to account for the compositional nature of the data. Spearman correlations between CLR transformed DA genera abundance and gene expression were performed in JMP17 (SAS Institute Inc., Cary, NC).

## Data Availability

The datasets generated and/or analyzed during the current study are available in the SRA repository under BioProject accession number PRJNA1289216, https://www.ncbi.nlm.nih.gov/bioproject/1289216.
