# Supplementary material for: Abnormal intestinal microbial colonization in prenatally stressed offspring is related to lung and intestinal cytokine expression
Source: Front Microbiol. 2026 Apr 9;17:1813467. doi: 10.3389/fmicb.2026.1813467 (PMC13102847; doi:10.3389/fmicb.2026.1813467)
Supplement: Supplementary file 1 [file Data_Sheet_1.PDF]

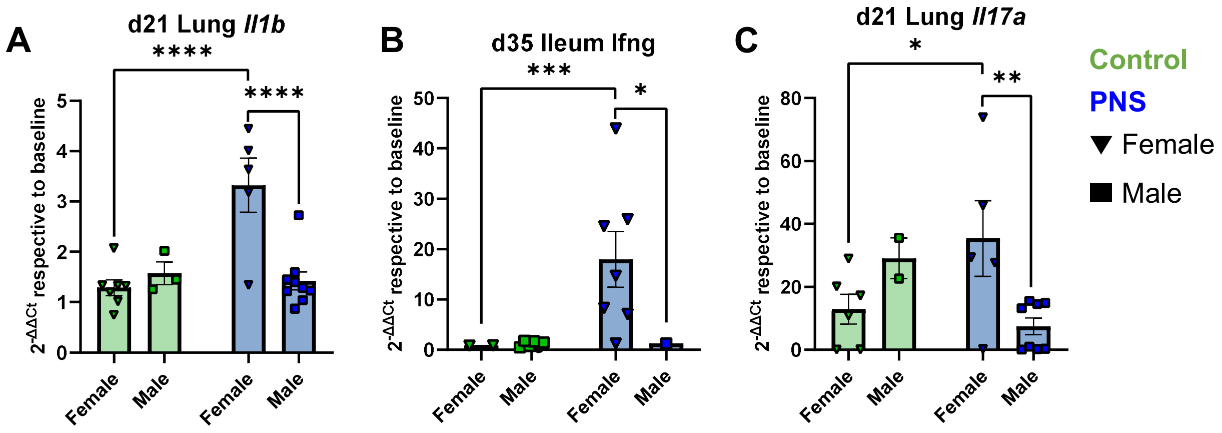

Supplementary Figure 1. Treatment by sex interactions. Gene expression found to have significant treatment by time point by sex interactions after fitting a linear mixed model. Post-hoc comparisons were made within treatment and between treatment respective to sex. Control values are denoted by green bars and PNS values are denoted by blue bars; females are denoted by triangles and males are denoted by squares. d21 ($n_{\text{control}♀}$ = 7, $n_{\text{control}♂}$ = 4; $n_{\text{PNS}♀}$ = 5, $n_{\text{PNS}♂}$ =9), d35 ($n_{\text{control}♀}$ = 2, $n_{\text{control}♂}$ = 6; $n_{\text{PNS}♀}$ = 7, $n_{\text{PNS}♂}$ = 2). Missing values are the result of outlier removal or due to samples with expression that was below qPCR detection limits. Bar graphs denote mean ± SEM. Sex effects were modeled via linear mixed effects models and reported where a significant 3-way interaction of treatment, time point, and sex were observed with litter included as a random intercept. **p* ≤ 0.05, ***p* ≤ 0.01, ****p* ≤ 0.001, *****p* ≤ 0.0001.

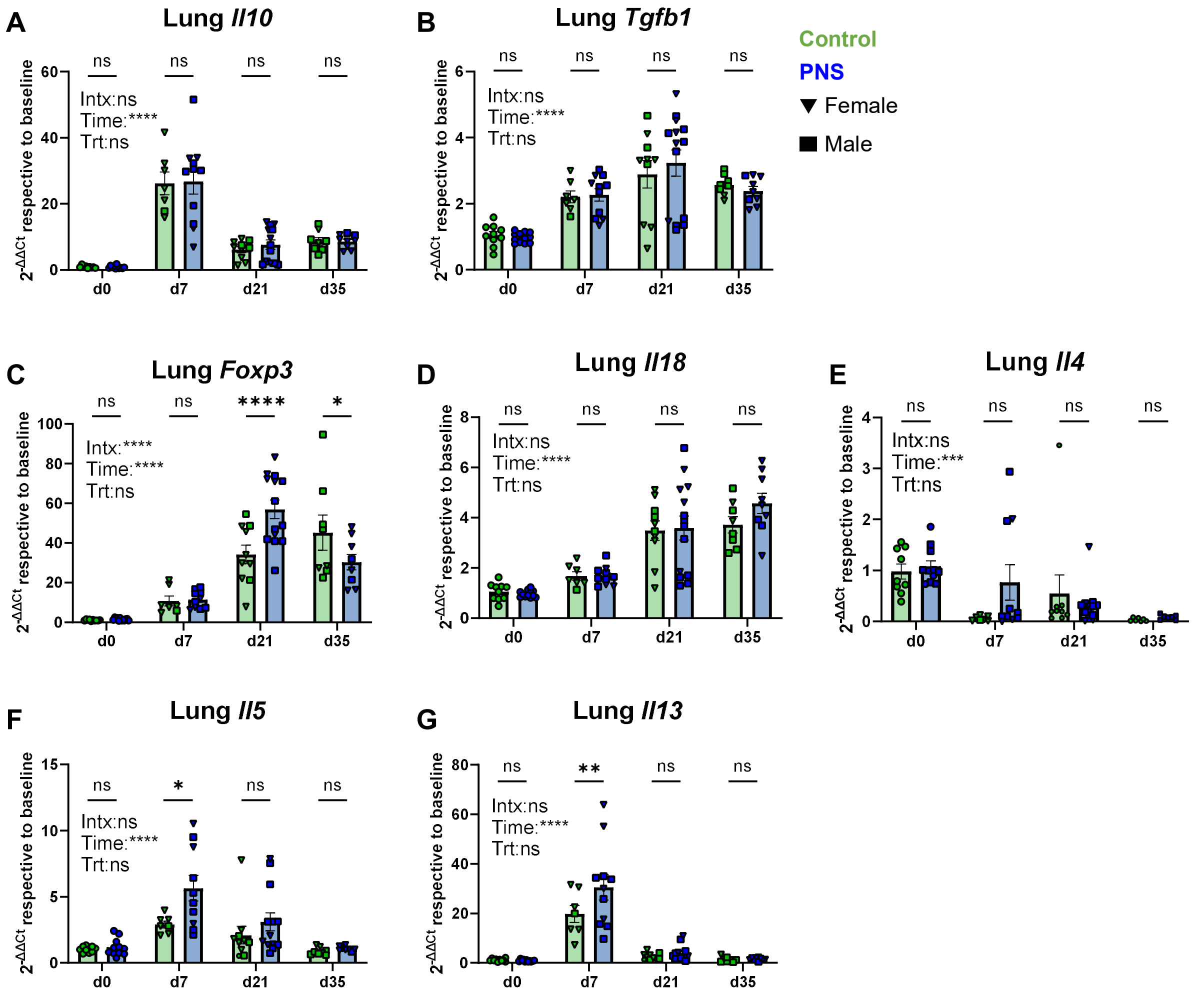

Supplementary Figure 2. Lung tissue alternative immunomodulatory gene expression. Expression of key anti-inflammatory **(A)**, reparative **(B)**, immunoregulatory **(C)**, Th1 **(D)**, Th2 **(E)**, and allergic response genes **(F-G)** were assessed in lung tissue of control and PNS offspring at d0, d7, d21, and d35. Values are expressed using the $2^{-\Delta\Delta Ct}$ method relative to control values at baseline to highlight how exposure to chronic prenatal stress altered gene expression over time. Control values are denoted by green bars and PNS values are denoted by blue bars; females are denoted by triangles and males are denoted by squares. d0 ($n_{control}$ = 10; n_{PNS} = 12), d7 ($n_{control♀}$ = 6, $n_{control♂}$ = 1; $n_{PNS♀}$ = 4, $n_{PNS♂}$ = 7), d21 ($n_{control♀}$ = 7, $n_{control♂}$ = 4; $n_{PNS♀}$ = 5, $n_{PNS♂}$ =9), d35 ($n_{control♀}$ = 2, $n_{control♂}$ = 6; $n_{PNS♀}$ = 7, $n_{PNS♂}$ = 2). Missing values are the result of outlier removal or due to samples with expression that was below qPCR detection limits. Bar graphs denote mean ± SEM. Fixed effects and their interaction are denoted on each graph, and post-hoc comparisons between control and PNS mice are denoted above each respective timepoint. Data were analyzed by linear mixed effects models examining effects of treatment, time point, and their interaction with litter included as a random intercept. $*p \leq 0.05$, $**p \leq 0.01$, $***p \leq 0.001$, $****p \leq 0.0001$. Intx: interaction, Time: main effect of time point, Trt: main effect of treatment.

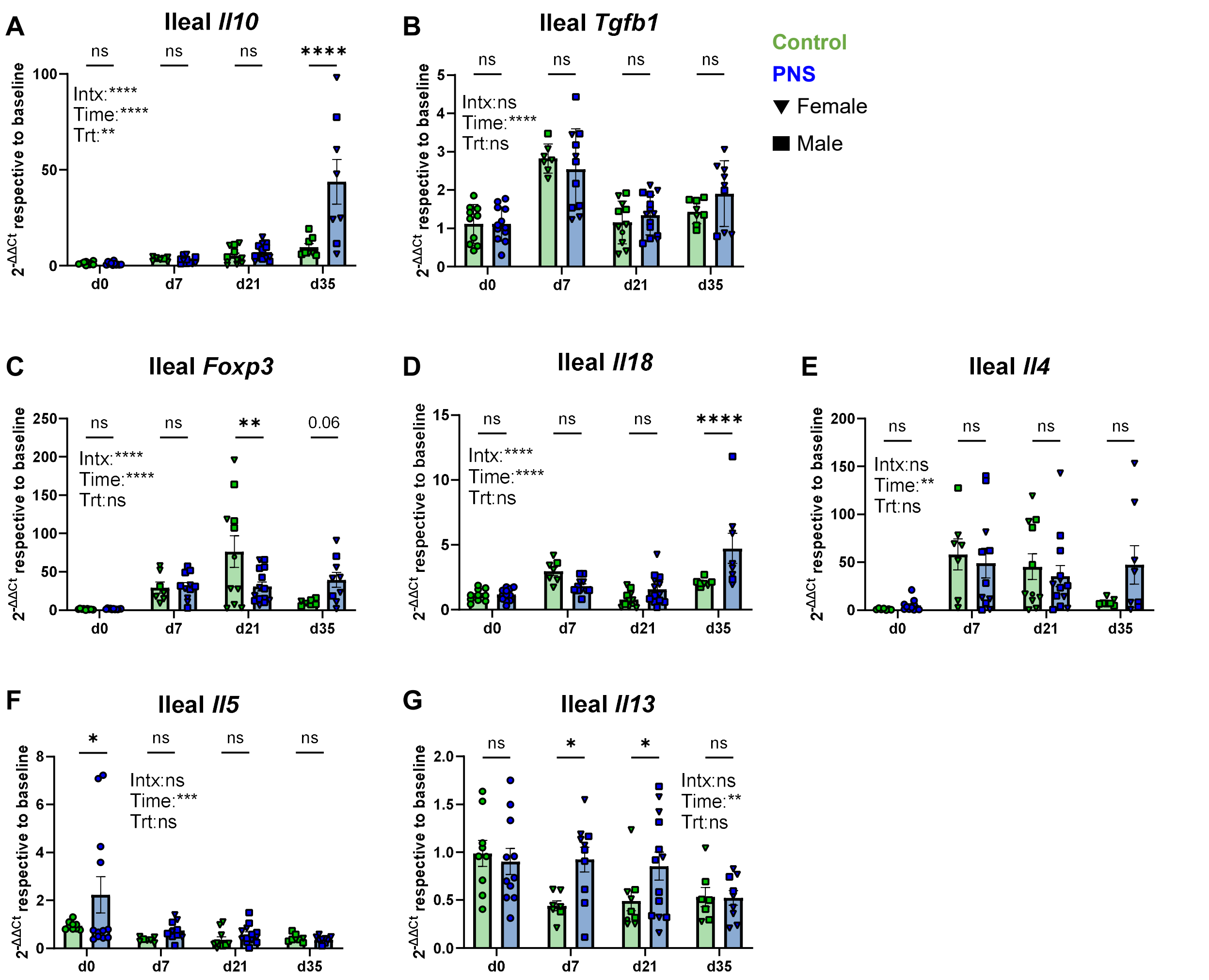

Supplementary Figure 3. Ileal tissue alternative immunomodulatory gene expression. Expression of key anti-inflammatory **(A)**, reparative **(B)**, immunoregulatory **(C)**, Th1 **(D)**, Th2 **(E)**, and allergic response genes **(F-G)** were assessed in ileal tissue of control and PNS offspring at d0, d7, d21, and d35. Values are expressed using the $2^{-\Delta\Delta Ct}$ method relative to control values at baseline to highlight how exposure to chronic prenatal stress altered gene expression over time. Control values are denoted by green bars and PNS values are denoted by blue bars; females are denoted by triangles and males are denoted by squares. d0 ($n_{control}$ = 10; n_{PNS} = 12), d7 ($n_{control♀}$ = 6, $n_{control♂}$ = 1; $n_{PNS♀}$ = 4, $n_{PNS♂}$ = 7), d21 ($n_{control♀}$ = 7, $n_{control♂}$ = 4; $n_{PNS♀}$ = 5, $n_{PNS♂}$ =9), d35 ($n_{control♀}$ = 2, $n_{control♂}$ = 6; $n_{PNS♀}$ = 7, $n_{PNS♂}$ = 2). Missing values are the result of outlier removal or due to samples with expression that was below qPCR detection limits. Bar graphs denote mean ± SEM. Fixed effects and their interaction are denoted on each graph, and post-hoc comparisons between control and PNS mice are denoted above each respective timepoint. Data were analyzed by linear mixed effects models examining effects of treatment, time point, and their interaction with litter included as a random intercept. *$p \leq 0.05$, **$p \leq 0.01$, ***$p \leq 0.001$, ****$p \leq 0.0001$. Intx: interaction, Time: main effect of time point, Trt: main effect of treatment.

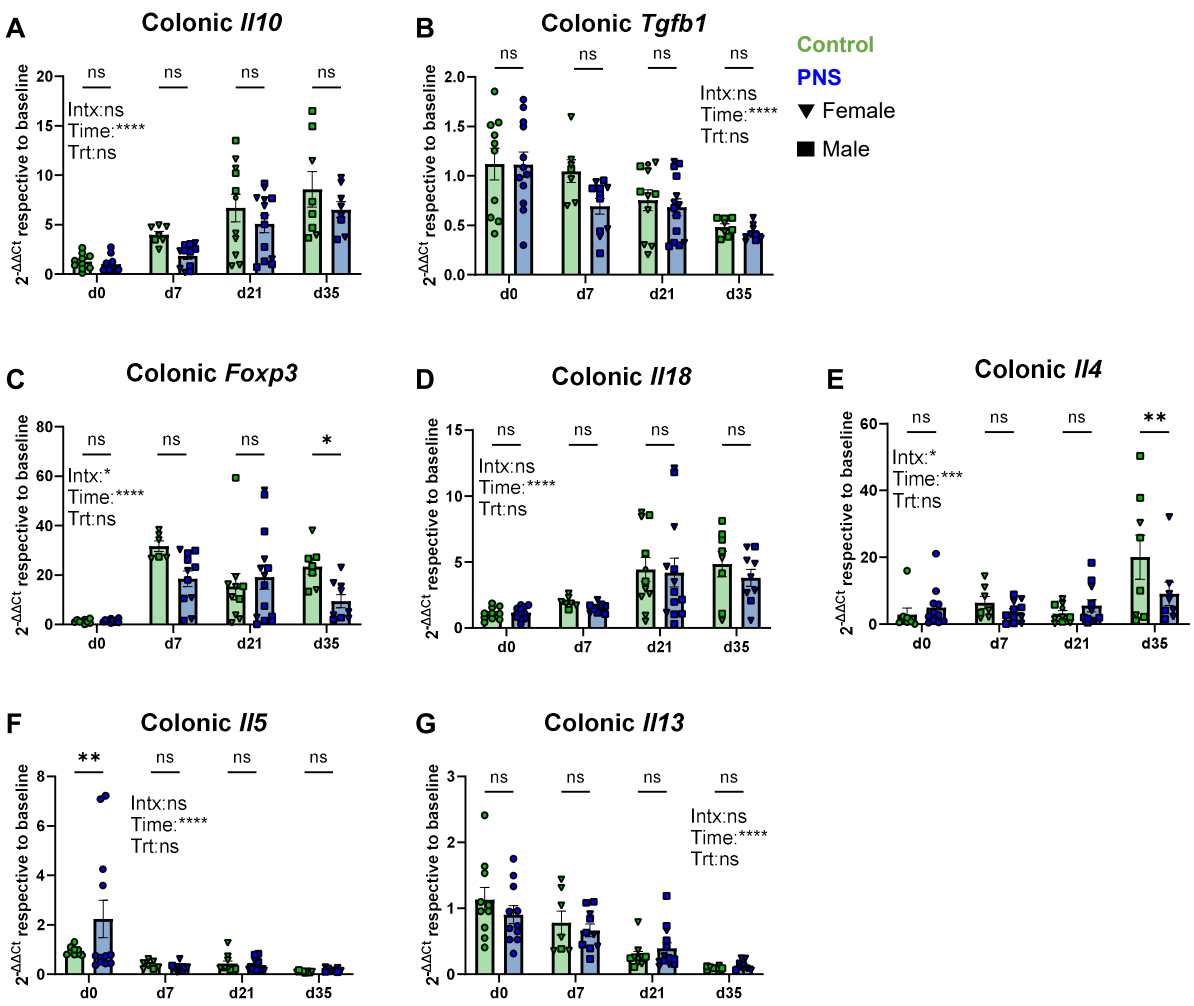

Supplementary Figure 4. Colonic tissue alternative immunomodulatory gene expression. Expression of key anti-inflammatory **(A)**, reparative **(B)**, immunoregulatory **(C)**, Th1 **(D)**, Th2 **(E)**, and allergic response genes **(F-G)** were assessed in colon tissue of control and PNS offspring at d0, d7, d21, and d35. Values are expressed using the $2^{-\Delta\Delta Ct}$ method relative to control values at baseline to highlight how exposure to chronic prenatal stress altered gene expression over time. Control values are denoted by green bars and PNS values are denoted by blue bars; females are denoted by triangles and males are denoted by squares. d0 ($n_{control}$ = 10; n_{PNS} = 12), d7 ($n_{control♀}$ = 6, $n_{control♂}$ = 1; $n_{PNS♀}$ = 4, $n_{PNS♂}$ = 7), d21 ($n_{control♀}$ = 7, $n_{control♂}$ = 4; $n_{PNS♀}$ = 5, $n_{PNS♂}$ =9), d35 ($n_{control♀}$ = 2, $n_{control♂}$ = 6; $n_{PNS♀}$ = 7, $n_{PNS♂}$ = 2). Missing values are the result of outlier removal or due to samples with expression that was below qPCR detection limits. Bar graphs denote mean ± SEM. Fixed effects and their interaction are denoted on each graph, and post-hoc comparisons between control and PNS mice are denoted above each respective timepoint. Data were analyzed by linear mixed effects models examining effects of treatment, time point, and their interaction with litter included as a random intercept. *$p \leq 0.05$, **$p \leq 0.01$, ***$p \leq 0.001$, ****$p \leq 0.0001$. Intx: interaction, Time: main effect of time point, Trt: main effect of treatment.

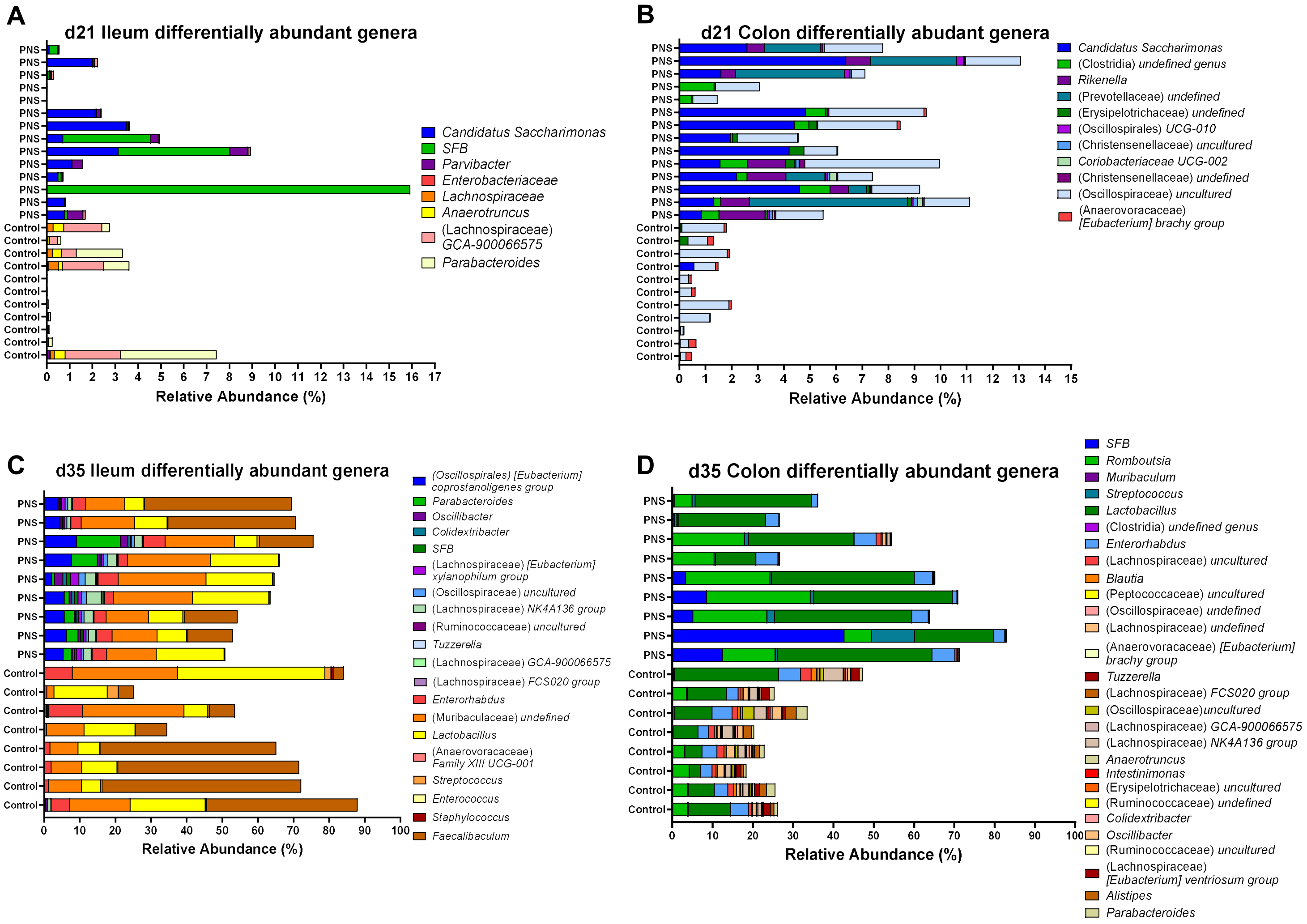

Supplementary Figure 5. Differentially abundant microbe relative abundance. Bar plots display relative abundance of significant differentially abundant genera in the ileum and colon at d21 ($n_{control♀} = 7$, $n_{control♂} = 4$; $n_{PNS♀} = 5$, $n_{PNS♂} = 9$)**(A-B)** and d35 ($n_{control♀} = 2$, $n_{control♂} = 6$; $n_{PNS♀} = 7$, $n_{PNS♂} = 2$) **(C-D)**. Blue, green, purple bars correspond to enriched features, and red, orange, and yellow bars correspond to depleted features. As only significantly different features are displayed, relative abundance graphs will not total 100% for each sample.

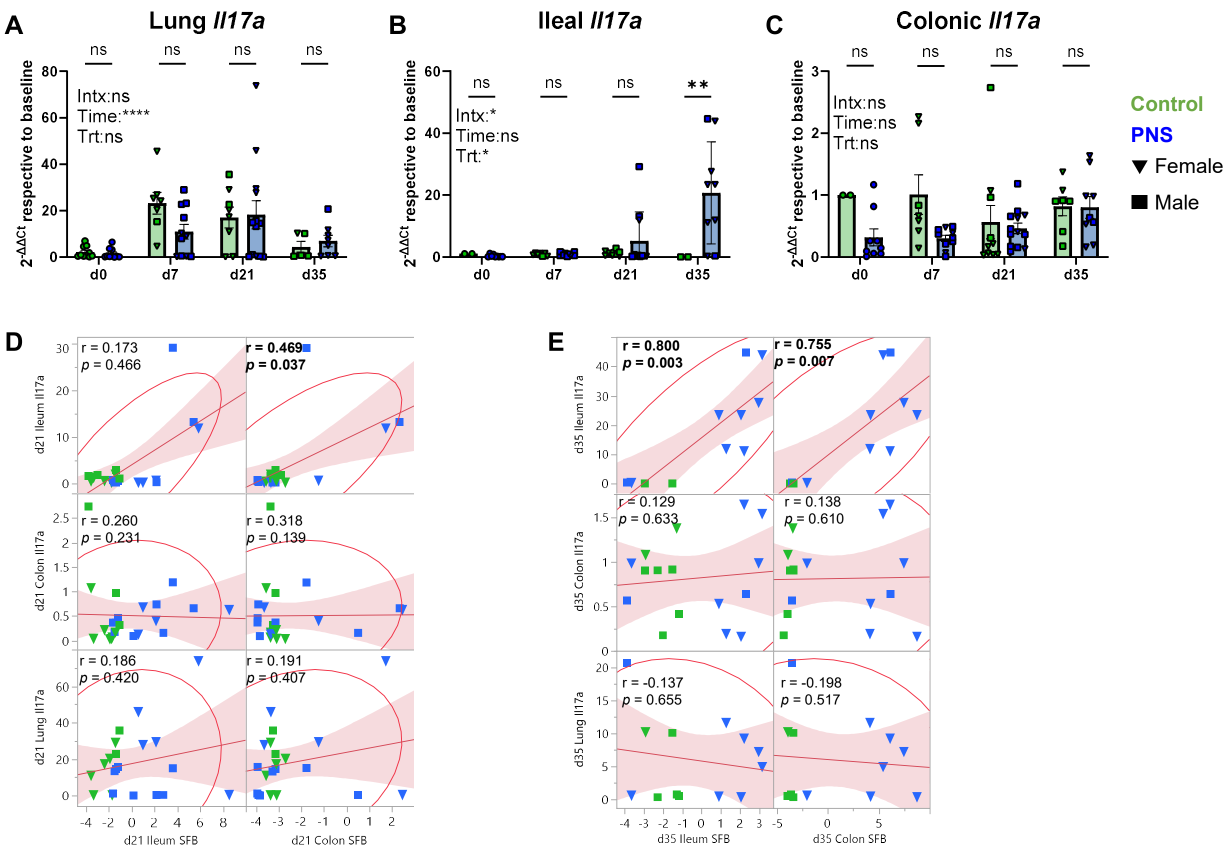

Supplementary Figure 6. IL-17a gene expression. Lung **(A)**, ileal **(B)**, and colonic **(C)** *Il17a* gene expression was assessed in control and PNS offspring at d0, d7, d21, and d35. Values are expressed using the $2^{-\Delta\Delta Ct}$ method relative to control values at baseline to highlight how exposure to chronic prenatal stress altered gene expression over time. Control values are denoted by green bars and PNS values are denoted by blue bars; females are denoted by triangles and males are denoted by squares. d0 ($n_{control}$ = 10; n_{PNS} = 12), d7 ($n_{control♀}$ = 6, $n_{control♂}$ = 1; $n_{PNS♀}$ = 4, $n_{PNS♂}$ = 7), d21 ($n_{control♀}$ = 7, $n_{control♂}$ = 4; $n_{PNS♀}$ = 5, $n_{PNS♂}$ =9), d35 ($n_{control♀}$ = 2, $n_{control♂}$ = 6; $n_{PNS♀}$ = 7, $n_{PNS♂}$ = 2). Missing values are the result of outlier removal or due to samples with expression that was below qPCR detection limits. Bar graphs denote mean ± SEM. Fixed effects and their interaction are denoted on each graph, and post-hoc comparisons between control and PNS mice are denoted above each respective timepoint. *Il17a* expression was analyzed by linear mixed effects models examining effects of treatment, time point, and their interaction with litter included as a random intercept. *$p \leq 0.05$, **$p \leq 0.01$, ***$p \leq 0.001$, ****$p \leq 0.0001$. Intx: interaction, Time: main effect of time point, Trt: main effect of treatment. Spearman correlations were used to assess the association between d21 ileum and colon SFB abundance and *Il17a* gene expression **(D)** and d35 ileum and colon SFB abundance and *Il17a* gene expression **(E)**. Center log ratio transformations were performed on bacterial abundances prior to computing correlations to account for the compositional nature of the data. All Spearman correlation r-values and *p*-values are displayed on respective scatterplots and are in bolded where $p \leq 0.05$. Within the scatterplot matrices, control data points are coded in green and PNS data points are coded in blue; females are denoted by triangles and males are denoted by squares. IC: ileum contents; CC: colon contents.

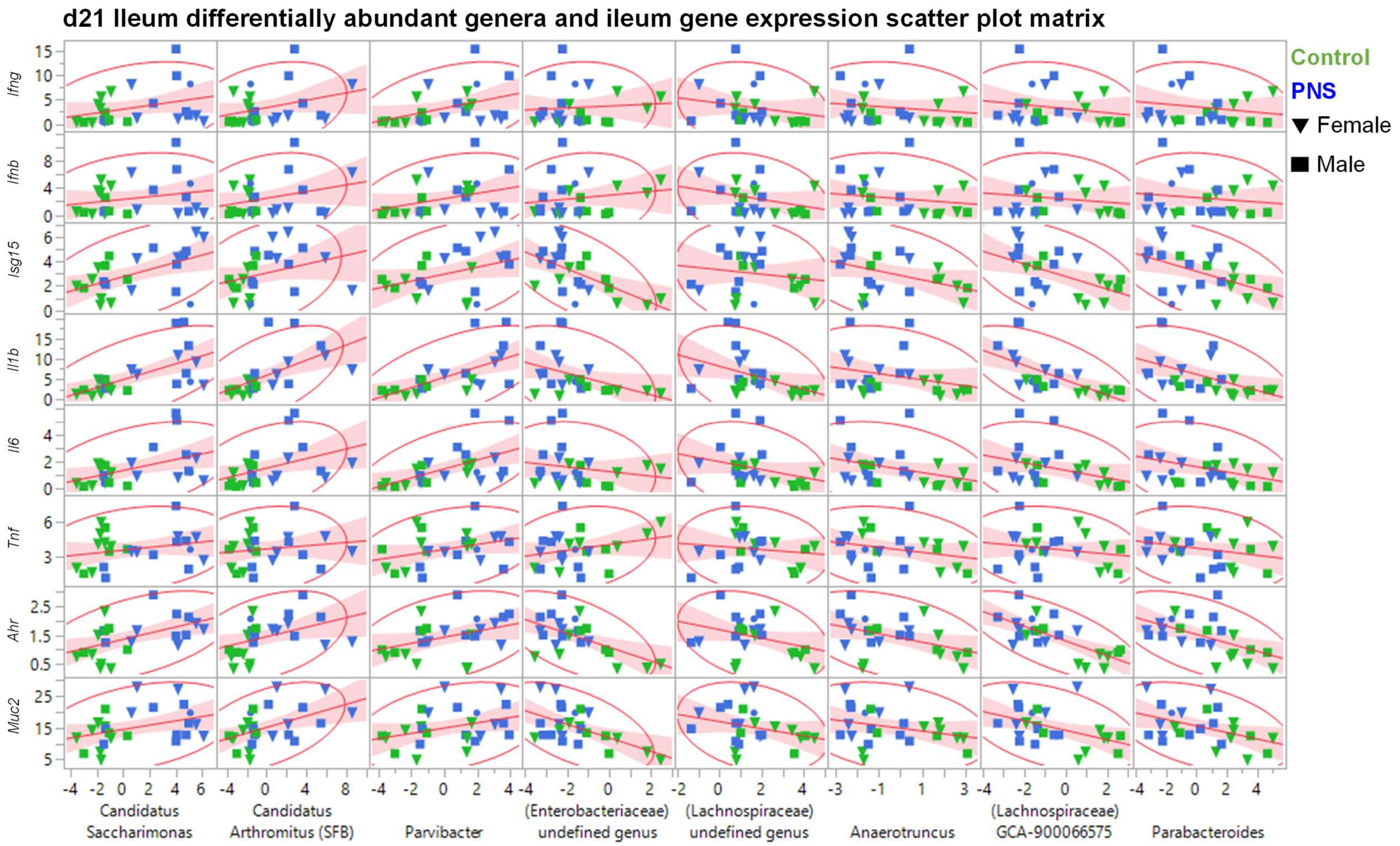

Supplementary Figure 7. Scatter plot matrix for d21 ileum DA and ileum gene expression correlations. Spearman correlations were used to assess the association between genera that were found to be differentially abundant in the ileum at d21 and d21 ileal gene expression. Center log ratio transformations were performed on bacterial abundances prior to computing correlations to account for the compositional nature of the data. Within the scatterplot matrices, control data points are coded in green and PNS data points are coded in blue; females are denoted by triangles and males are denoted by squares ($n_{control♀}$ = 7, $n_{control♂}$ = 4; $n_{PNS♀}$ = 5, $n_{PNS♂}$ =9). All corresponding Spearman correlation r-values and *p*-values can be found in Supplementary Table 2.

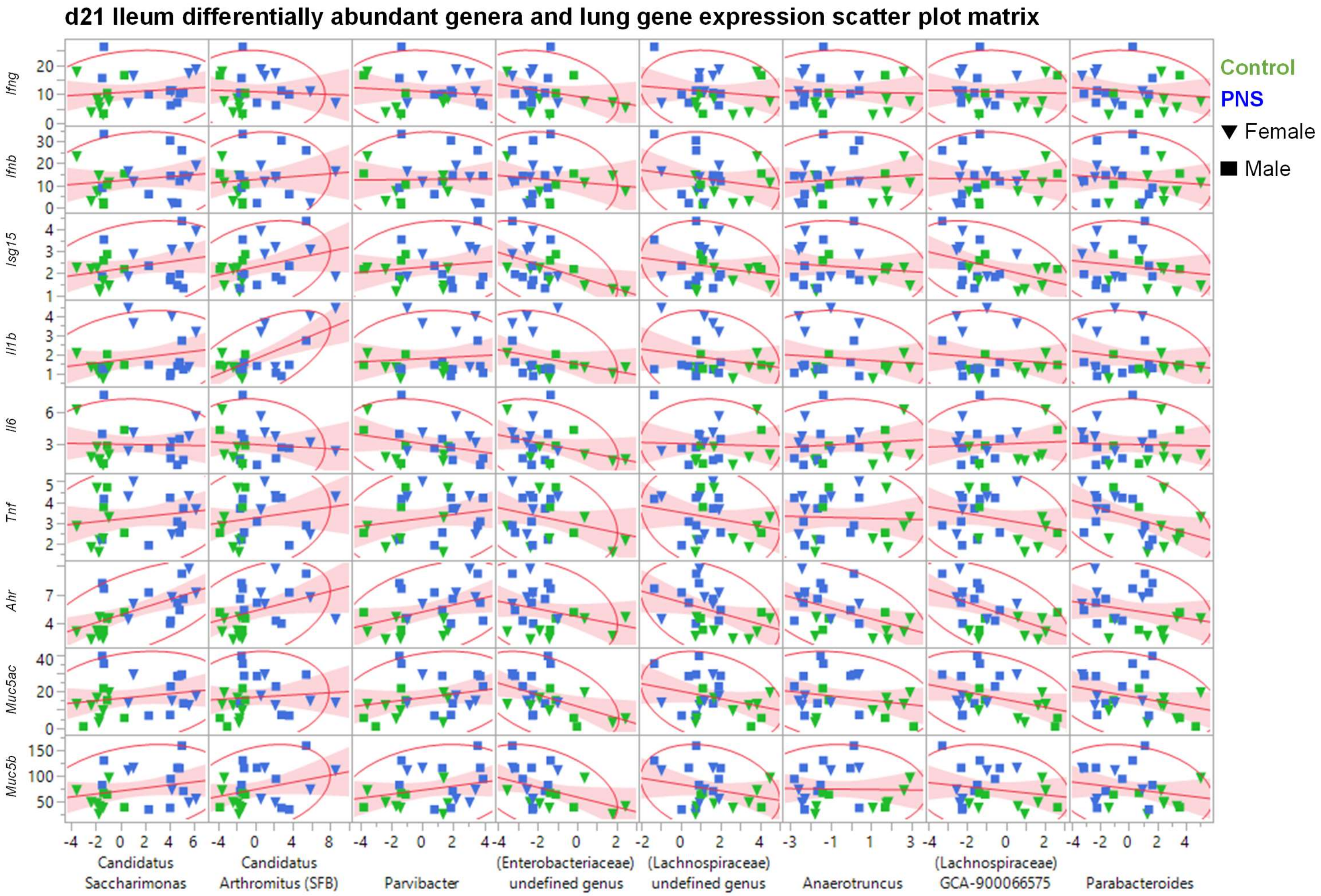

Supplementary Figure 8. Scatter plot matrix for d21 ileum DA and lung gene expression correlations. Spearman correlations were used to assess the association between genera that were found to be differentially abundant in the ileum at d21 and d21 lung gene expression. Center log ratio transformations were performed on bacterial abundances prior to computing correlations to account for the compositional nature of the data. Within the scatterplot matrices, control data points are coded in green and PNS data points are coded in blue; females are denoted by triangles and males are denoted by squares ($n_{control♀}$ = 7, $n_{control♂}$ = 4; $n_{PNS♀}$ = 5, $n_{PNS♂}$ =9). All corresponding Spearman correlation r-values and *p*-values can be found in Supplementary Table 3.

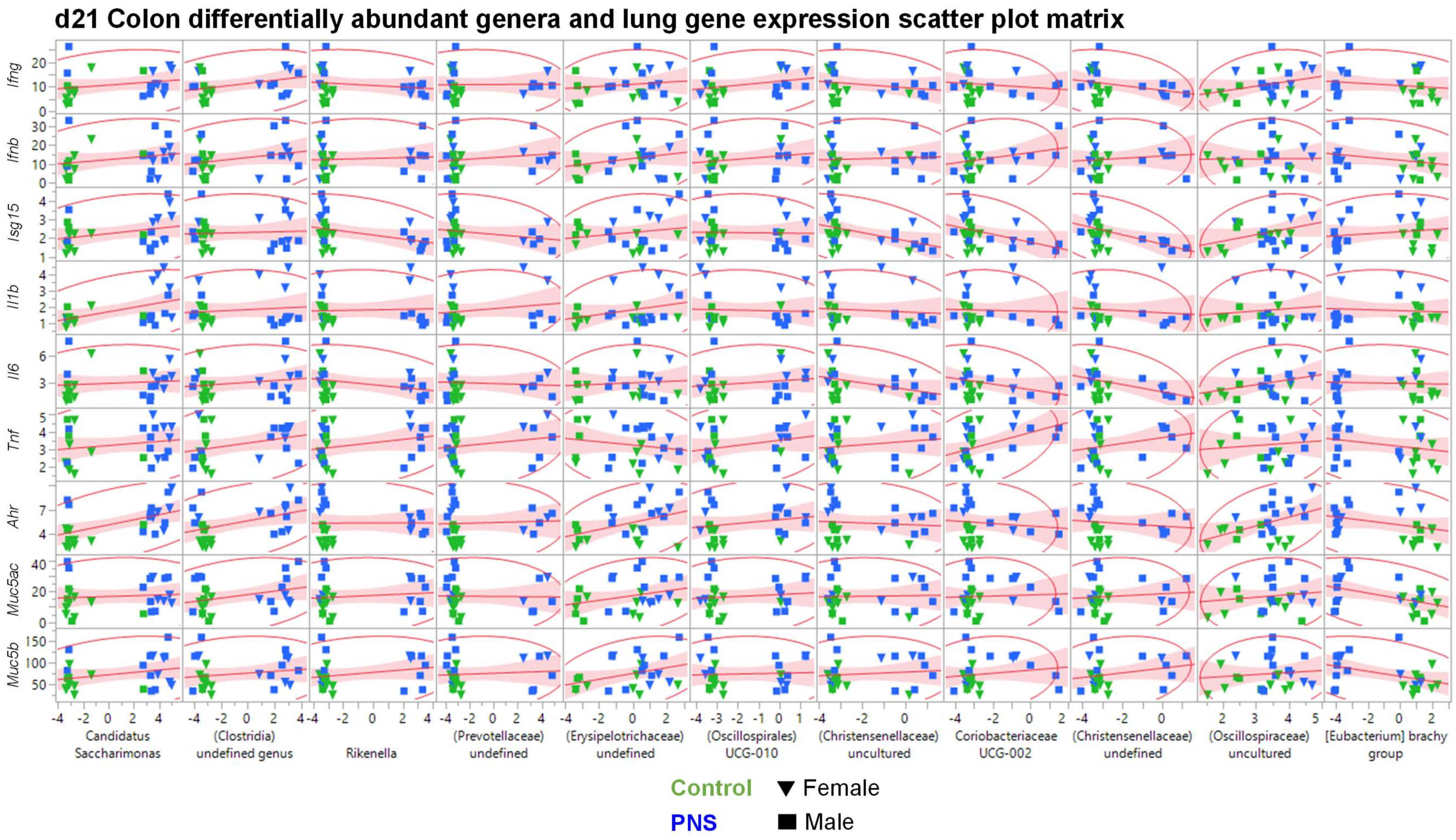

Supplementary Figure 9. Scatter plot matrix for d21 colon DA and lung gene expression correlations. Spearman correlations were used to assess the association between genera that were found to be differentially abundant in the colon at d35 and d35 lung gene expression. Center log ratio transformations were performed on bacterial abundances prior to computing correlations to account for the compositional nature of the data. Within the scatterplot matrices, control data points are coded in green and PNS data points are coded in blue; females are denoted by triangles and males are denoted by squares ($n_{control♀} = 7$, $n_{control♂} = 4$; $n_{PNS♀} = 5$, $n_{PNS♂} = 9$). All corresponding Spearman correlation r-values and *p*-values can be found in Supplementary Table 4.

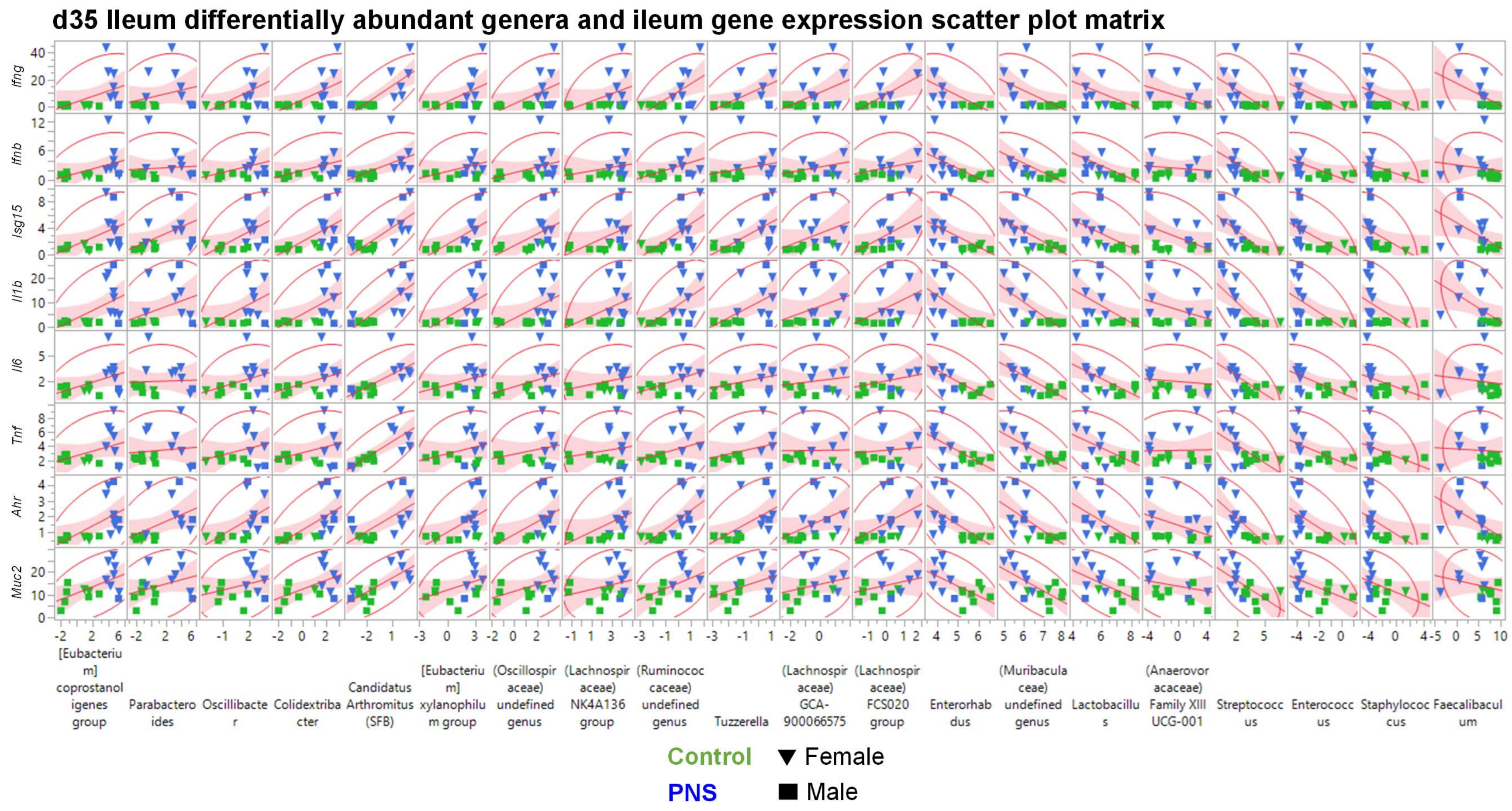

Supplementary Figure 10. Scatter plot matrix for d35 ileum DA and ileum gene expression correlations. Spearman correlations were used to assess the association between genera that were found to be differentially abundant in the ileum at d35 and d35 ileal gene expression. Center log ratio transformations were performed on bacterial abundances prior to computing correlations to account for the compositional nature of the data. Within the scatterplot matrices, control data points are coded in green and PNS data points are coded in blue; females are denoted by triangles and males are denoted by squares ($n_{control♀} = 2$, $n_{control♂} = 6$; $n_{PNS♀} = 7$, $n_{PNS♂} = 2$). All corresponding Spearman correlation r-values and *p*-values can be found in Supplementary Table 5.

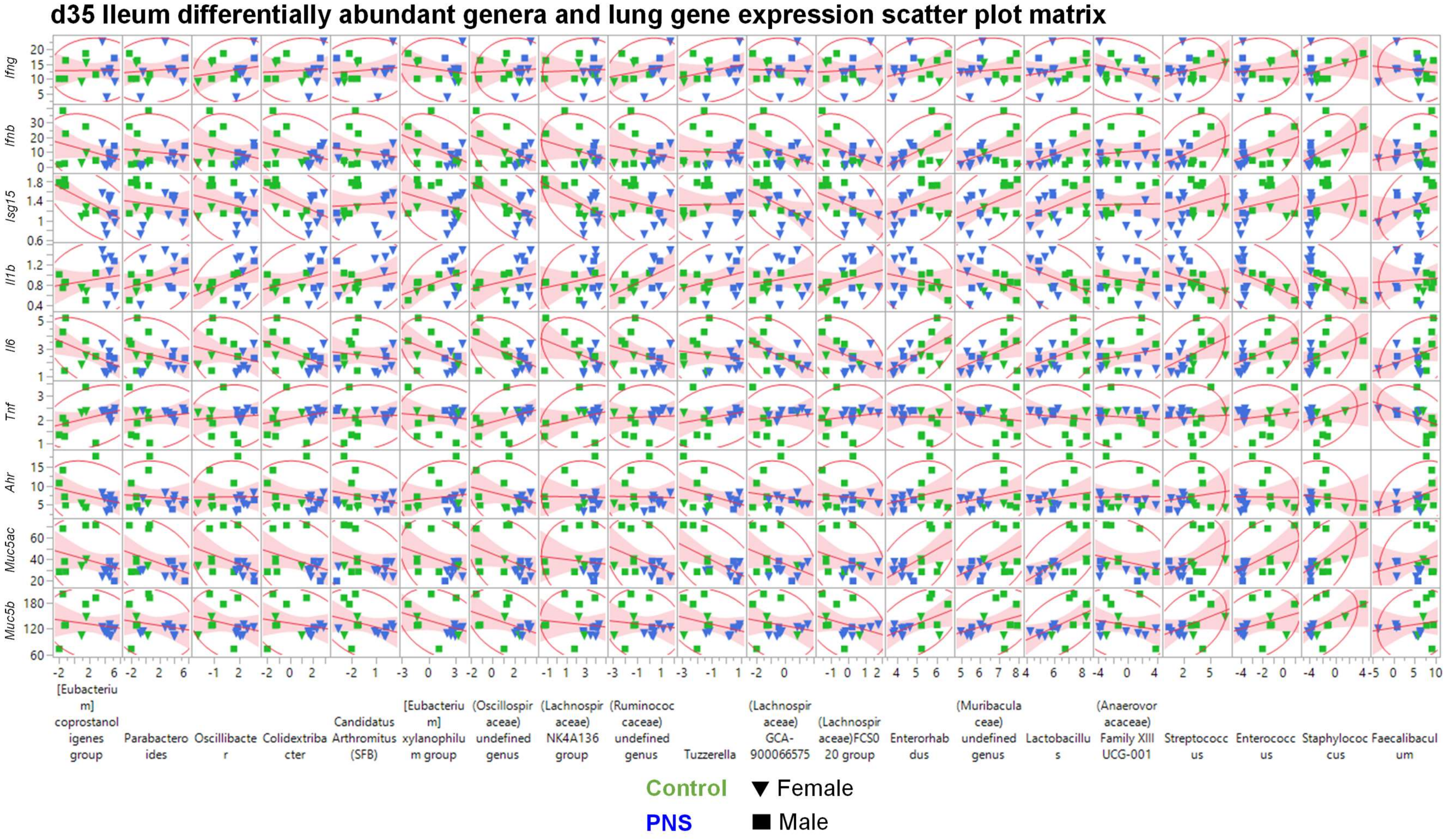

Supplementary Figure 11. Scatter plot matrix for d35 ileum DA and lung gene expression correlations. Spearman correlations were used to assess the association between genera that were found to be differentially abundant in the ileum at d35 and d35 lung gene expression. Center log ratio transformations were performed on bacterial abundances prior to computing correlations to account for the compositional nature of the data. Within the scatterplot matrices, control data points are coded in green and PNS data points are coded in blue; females are denoted by triangles and males are denoted by squares ($n_{control♀}$ = 2, $n_{control♂}$ = 6; $n_{PNS♀}$ = 7, $n_{PNS♂}$ = 2). All corresponding Spearman correlation r-values and *p*-values can be found in Supplementary Table 6.

d35 Colon differentially abundant genera and lung gene expression scatter plot matrix

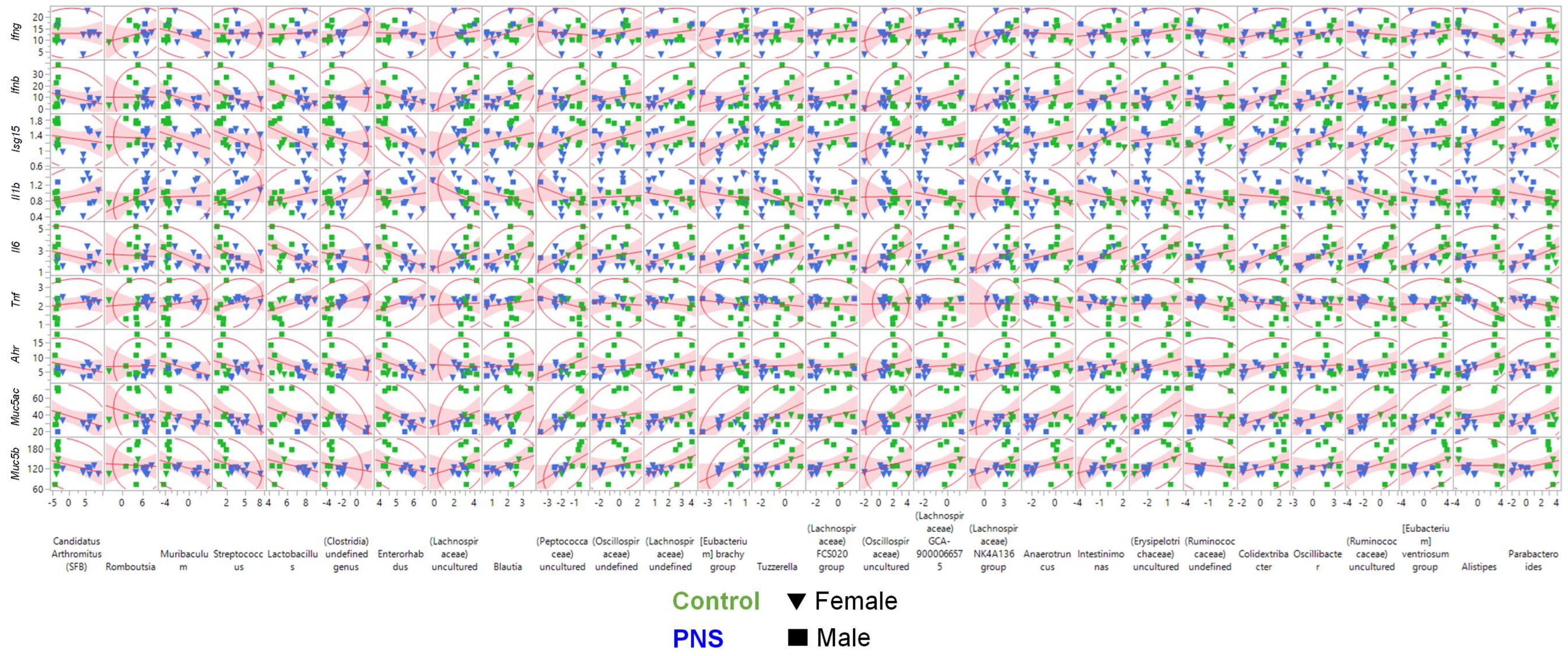

Supplementary Figure 12. Scatter plot matrix for d35 colon DA and lung gene expression correlations. Spearman correlations were used to assess the association between genera that were found to be differentially abundant in the colon at d35 and d35 lung gene expression. Center log ratio transformations were performed on bacterial abundances prior to computing correlations to account for the compositional nature of the data. Within the scatterplot matrices, control data points are coded in green and PNS data points are coded in blue; females are denoted by triangles and males are denoted by squares ($n_{control♀}$ = 2, $n_{control♂}$ = 6; $n_{PNS♀}$ = 7, $n_{PNS♂}$ = 2). All corresponding Spearman correlation r-values and *p*-values can be found in Supplementary Table 7.

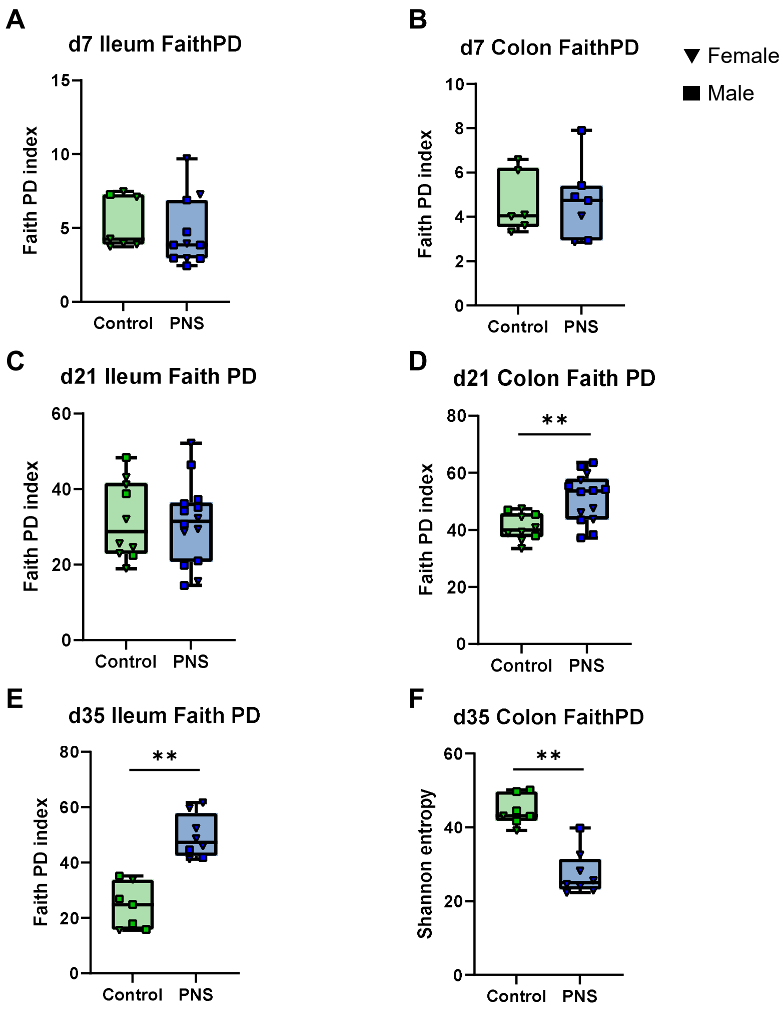

Supplementary Figure 13. Supplemental alpha diversity. Faith PD alpha diversity in ileum and colon contents at d7 **(A-B)**, d21 **(C-D)**, and d35 **(E-F)**. Differences in alpha diversity were analyzed in QIIME 2.0 by Kruskal-Wallis tests; $*p \leq 0.05$, $**p \leq 0.01$. Alpha diversity values are represented as box and whisker plots where boxes denote interquartile range and upper and lower whiskers denote minimum and maximum, respectively. Control offspring are denoted in green and PNS offspring are denoted in blue; females are denoted by triangles and males are denoted by squares. d7 ($n_{\text{control♀}} = 6$, $n_{\text{control♂}} = 1$; $n_{\text{PNS♀}} = 4$, $n_{\text{PNS♂}} = 7$), d21 ($n_{\text{control♀}} = 7$, $n_{\text{control♂}} = 4$; $n_{\text{PNS♀}} = 5$, $n_{\text{PNS♂}} = 9$), d35 ($n_{\text{control♀}} = 2$, $n_{\text{control♂}} = 6$; $n_{\text{PNS♀}} = 7$, $n_{\text{PNS♂}} = 2$). Missing values were dropped due to rarefaction.

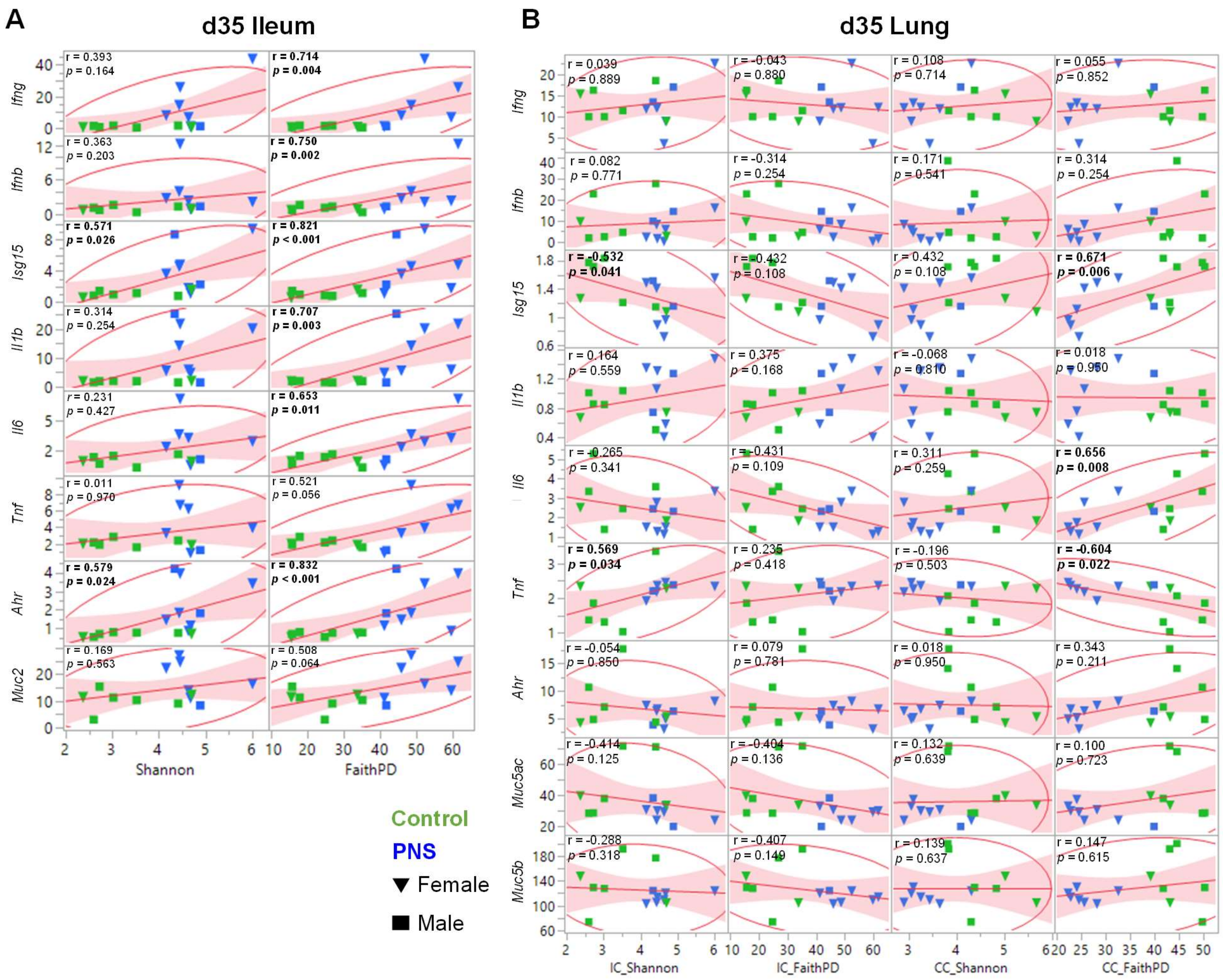

Supplementary Figure 14. Gene expression and alpha diversity. Spearman correlations were used to assess the association between **(A)** d35 ileum alpha diversity and d35 ileum gene expression and **(B)** d35 ileum and colon alpha diversity and d35 lung gene expression ($n_{control♀}$ = 2, $n_{control♂}$ = 6; $n_{PNS♀}$ = 7, $n_{PNS♂}$ = 2). The d35 timepoint is highlighted here as both ileum and colon d35 alpha diversity was significantly different between PNS and control offspring. All Spearman correlation r-values and *p*-values are displayed on respective scatterplots and are in bolded where $p \leq 0.05$. Within the scatterplot matrices, control data points are coded in green and PNS data points are coded in blue; females are denoted by triangles and males are denoted by squares. IC: ileum contents; CC: colon contents.

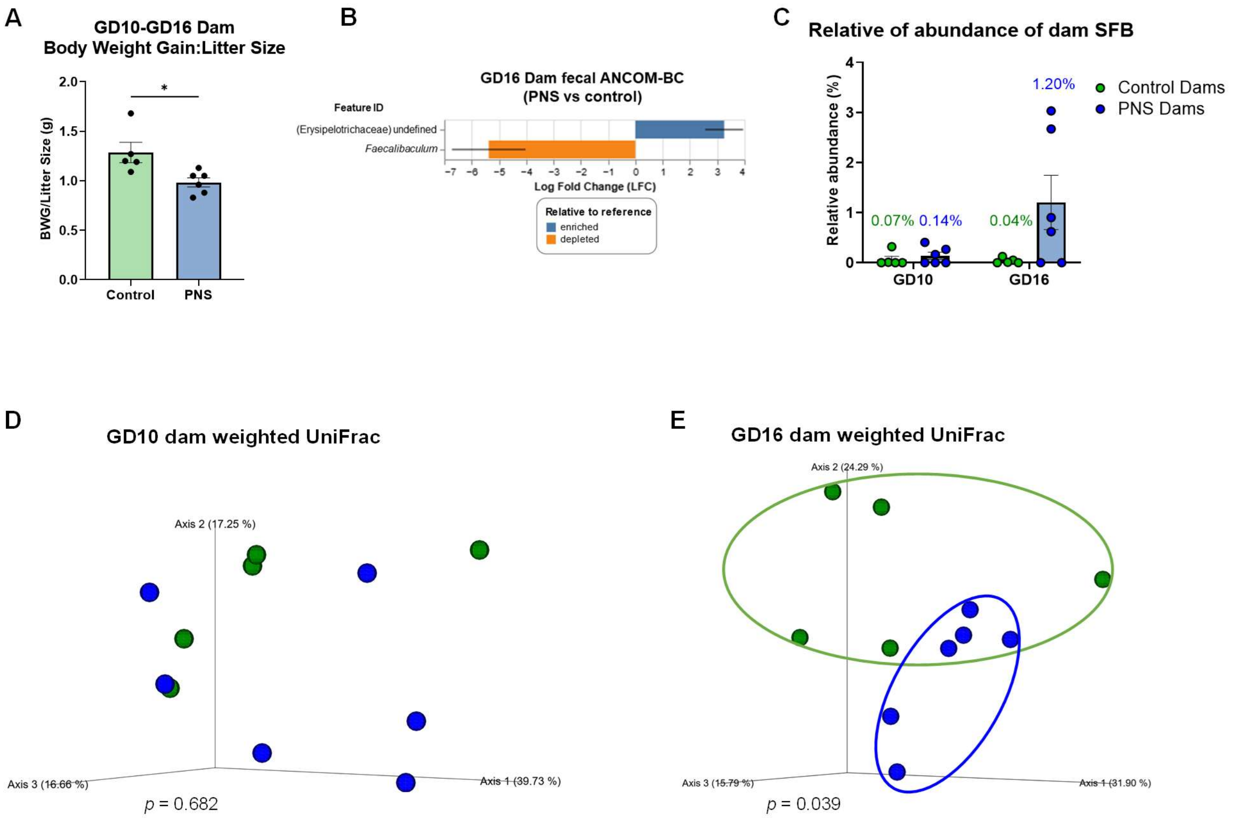

Supplementary Figure 15. Dam Metrics. Body weight gain during the prenatal stress period (GD10-GD16) in control (n =5) versus PNS (n = 6) dams relative to litter size **(A)**; differences in relative body weight gain were assessed via t-test. ANCOM-BC bar plot of differences in genus level taxa (feature) differential abundance between control and PNS dam fecal samples **(B)**. Analyses were conducted in QIIME 2.0 with Holm's FDR correction. Taxa denoted in bar plots were differentially enriched (blue) or depleted (orange) in PNS dams relative to control dams ($q \leq 0.05$). Relative abundance of segmented filamentous bacteria (SFB) in dams at GD10 versus GD16; mean values are displayed above respective bars **(C)**. No statistical testing was performed on raw relative abundance values due to the compositional nature of the data. Principal coordinate analysis plots of weighted UniFrac distance matrices for dam fecals at GD10 **(D)** and GD16 **(E)**. Differences in beta diversity distances between treatments were analyzed in QIIME 2.0 by PERMANOVA with 999 randomizations of the data, and *p*-values are displayed below respective graphs. Control dams are denoted by green spheres, PNS dams are denoted by blue spheres, and ellipses have been drawn around groups where beta diversity distances were significantly different ($p \leq 0.05$).

Supplementary Figure 16. d7 Ileal Relative Abundance. Bar graphs denote genus-level relative abundance across individual samples.

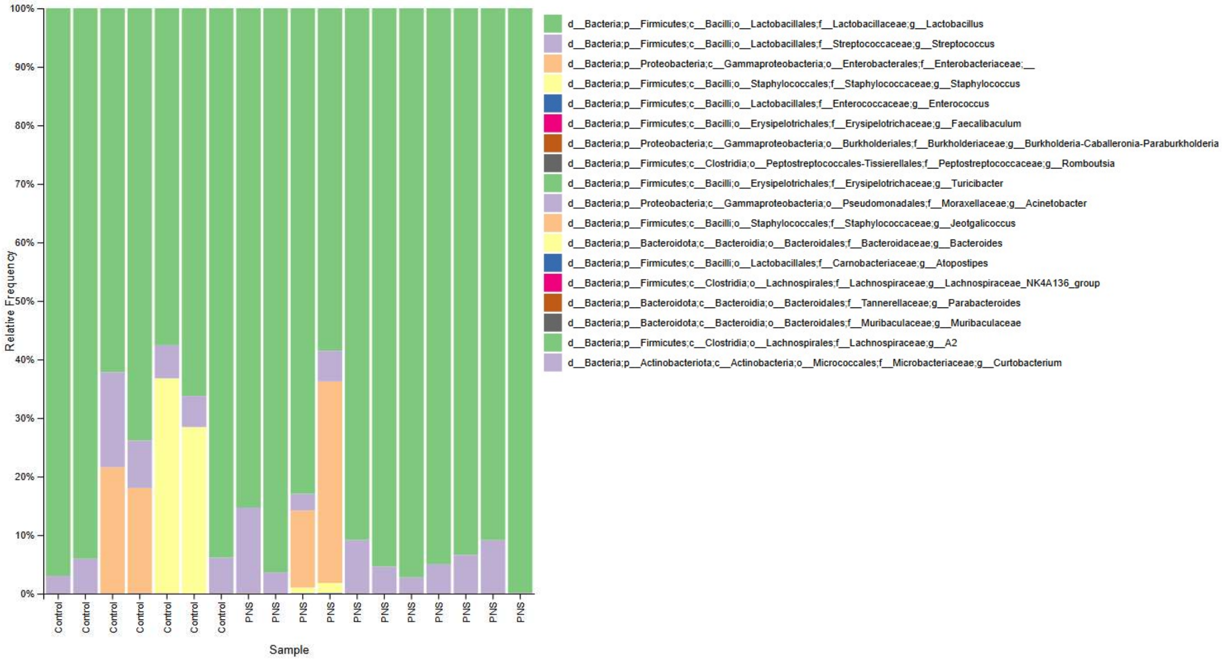

Supplementary Figure 17. d21 Ileal Relative Abundance. Bar graphs denote genus-level relative abundance across individual samples.

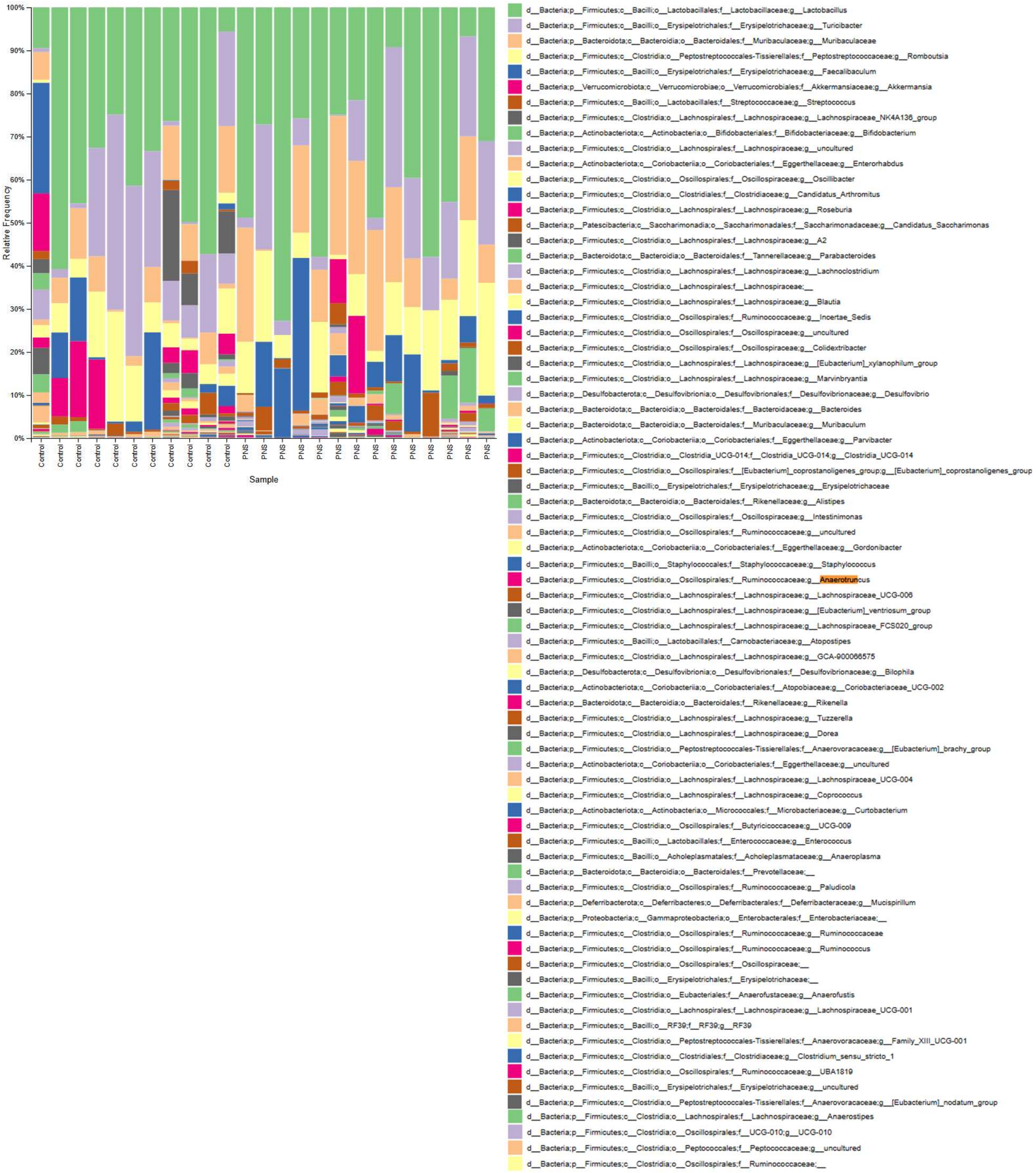

Supplementary Figure 18. d35 Ileal Relative Abundance. Bar graphs denote genus-level relative abundance across individual samples.

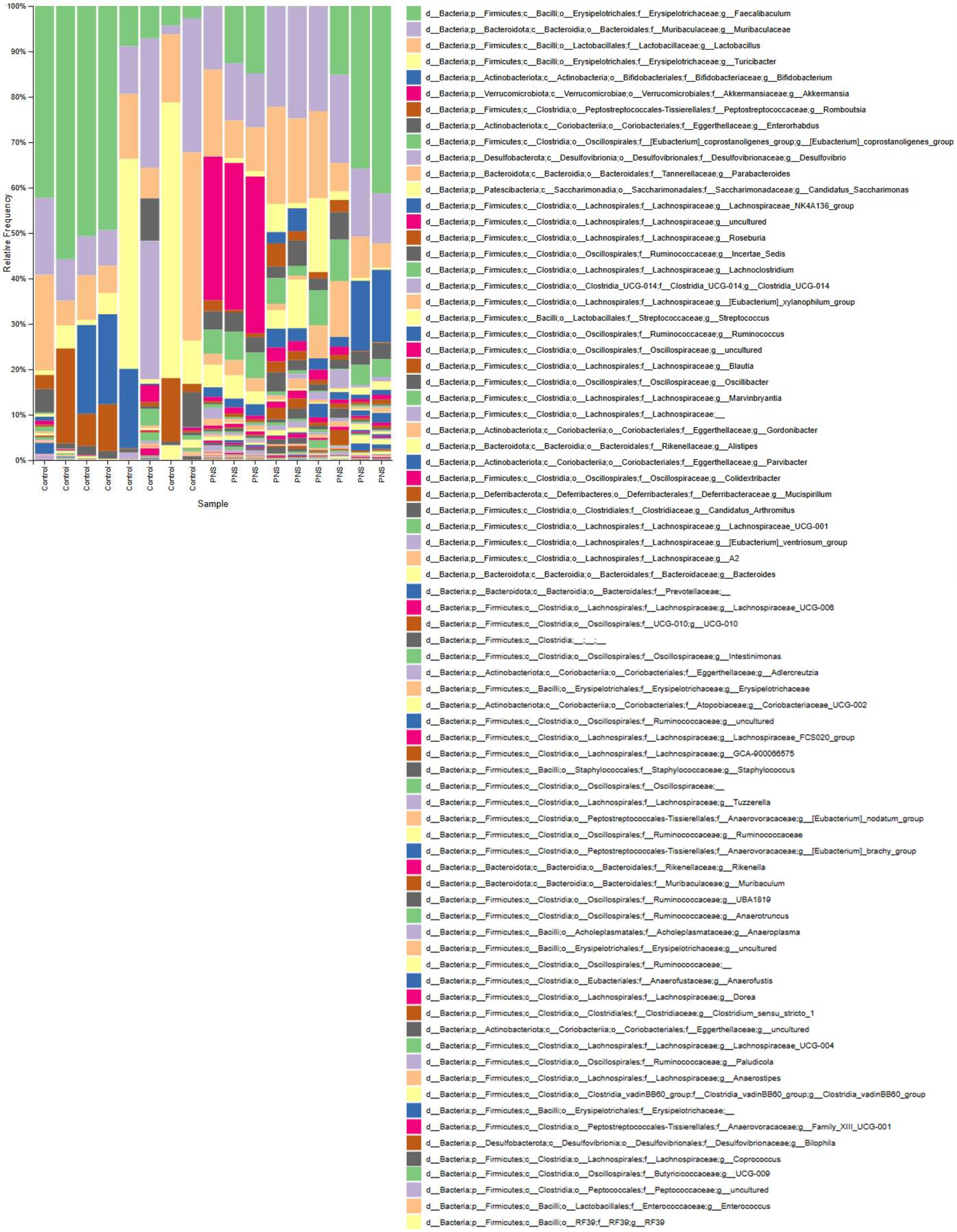

Supplementary Figure 19. d7 Colonic Relative Abundance. Bar graphs denote genus-level relative abundance across individual samples.

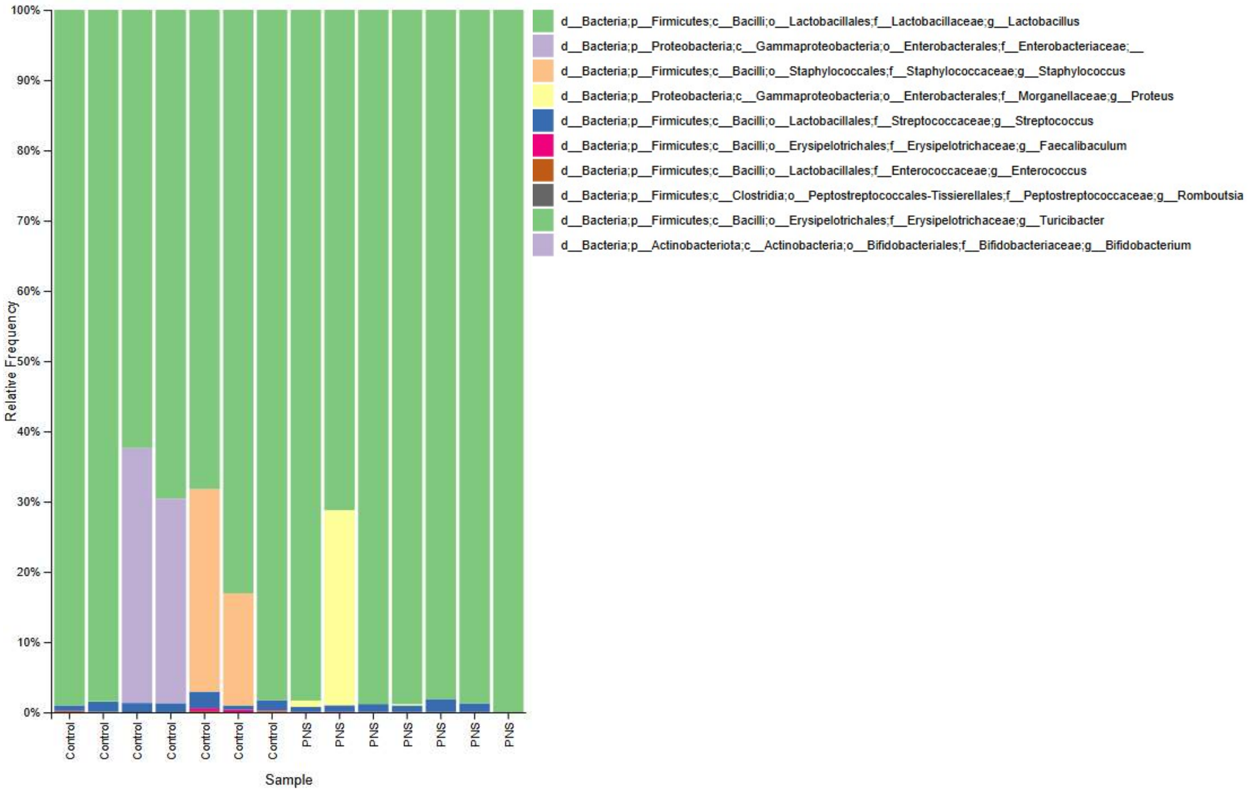

Supplementary Figure 20. d21 Colonic Relative Abundance. Bar graphs denote genus-level relative abundance across individual samples.

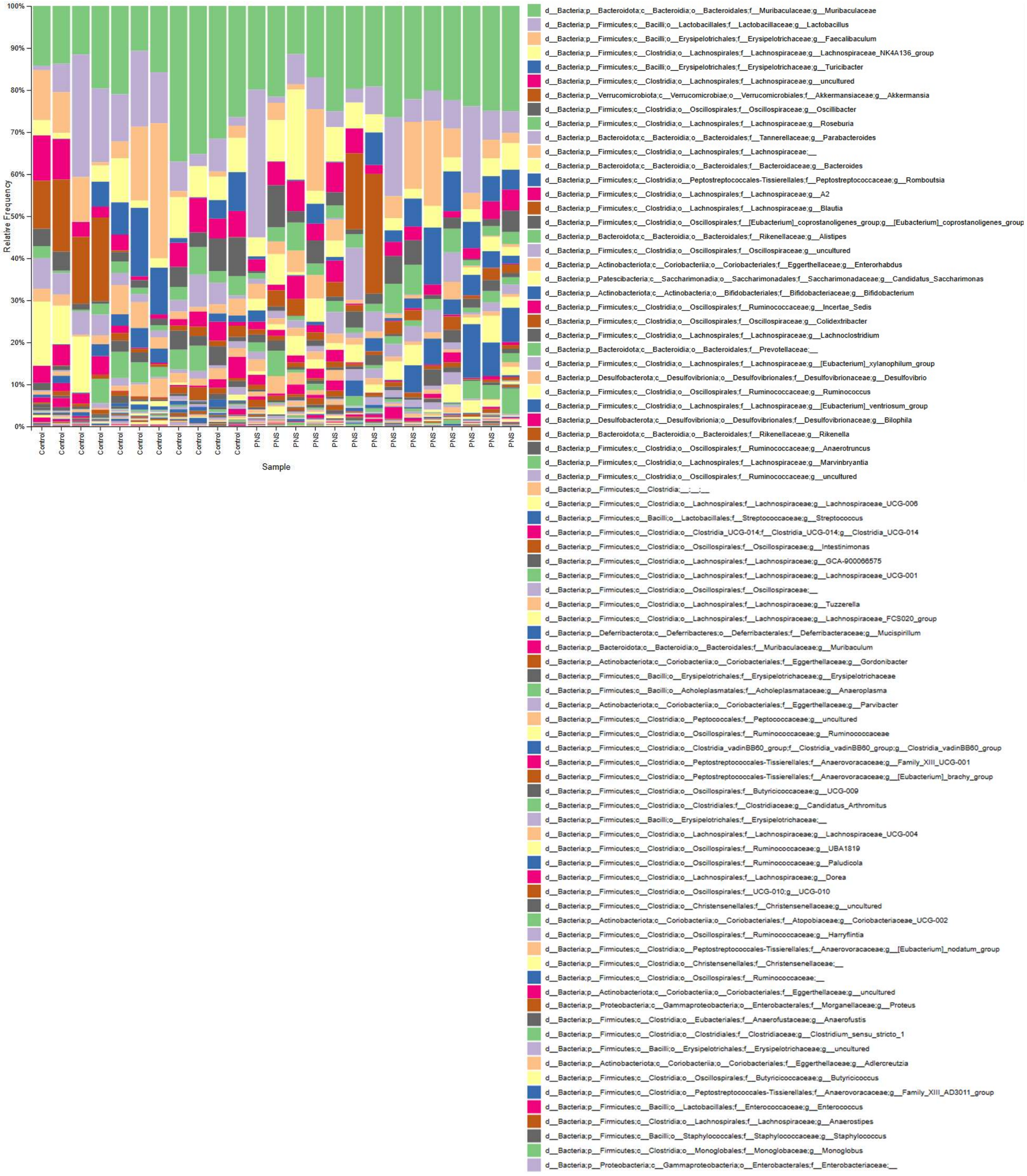

Supplementary Figure 21. d35 Colonic Relative Abundance. Bar graphs denote genus-level relative abundance across individual samples.

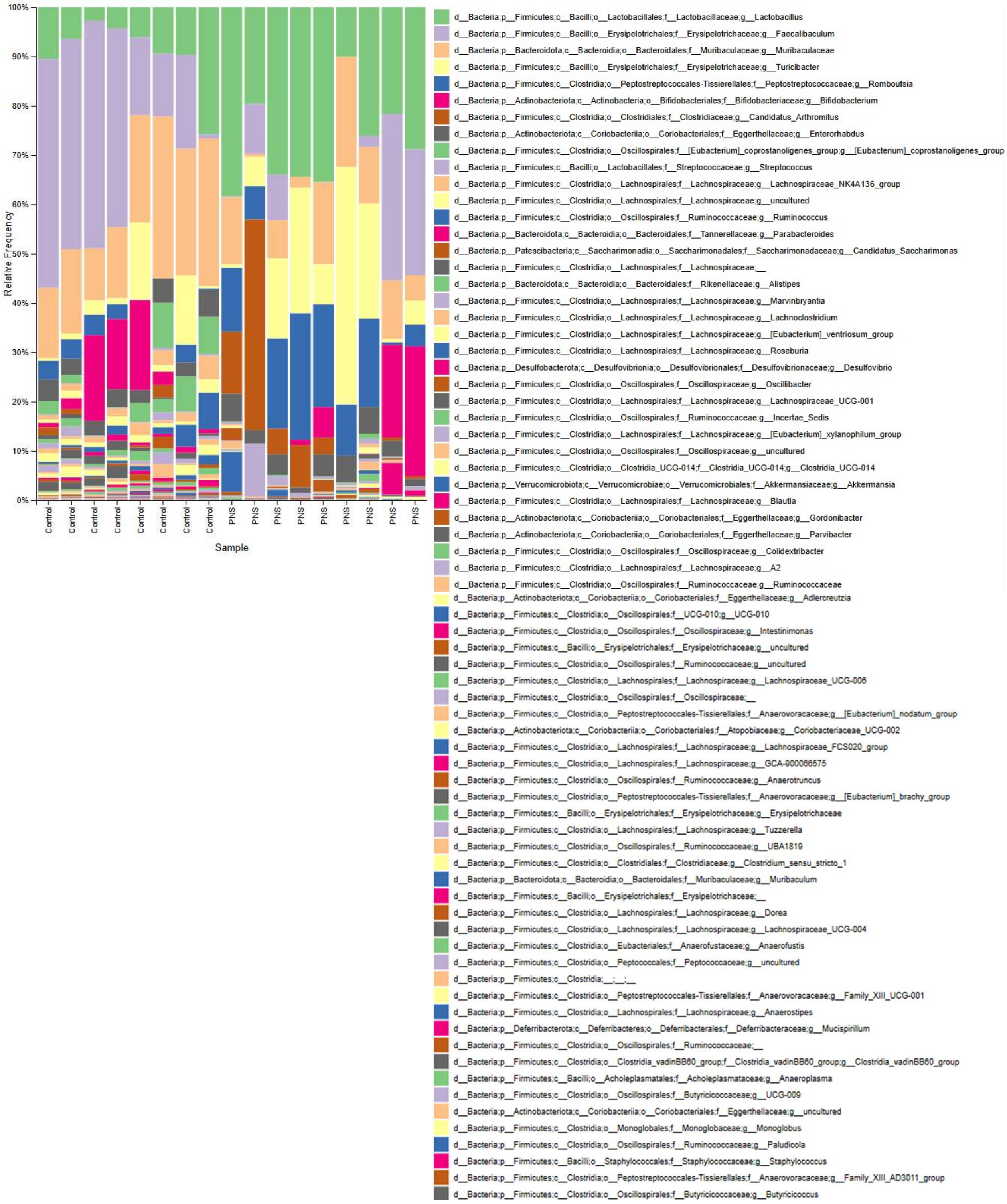

Supplementary Table 1. Microbiome diversity sex effects.

Diversity by Sex

Ileum (Sex within Treatment)	d7 Shannon Diversity	d7 Faith PD	d21 Shannon Diversity	d21 Faith PD	d35 Shannon Diversity	d35 Faith PD
PNS (F vs M)	0.131	0.218	0.641	0.739	1.000	0.182
Control (F vs M)	0.617	0.317	0.569	0.569	1.000	0.699

Ileum (Sex within Treatment)	d7 Unweighted UniFrac	d21 Unweighted UniFr	d35 Unweighted UniFrac
PNS (F vs M)	0.717	0.635	0.422
Control (F vs M)	0.445	0.098	0.707

Colon (Sex within Treatment)	d7 Shannon Diversity	d7 Faith PD	d21 Shannon Diversity	d21 Faith PD	d35 Shannon Diversity	d35 Faith PD
PNS (F vs M)	0.699	0.121	0.841	0.947	0.275	0.127
Control (F vs M)	Females only*	Females only*	0.425	0.425	0.053	0.245

*Only females left after rarefaction for diversity analyses

Colon (Sex within Treatment)	d7 Unweighted UniFrac	l21 Unweighted UniFra	d35 Unweighted UniFrac
PNS (F vs M)	0.454	0.546	0.129
Control (F vs M)	Females only*	**0.035**	0.227

*Only females left after rarefaction for diversity analyses

Sex within treatment statistically compared at each time point in QIIME 2.0 using respective alpha and beta diversity plugins.

Supplementary Table 2. d21 Ileum CLR-transformed significant DA and ileum gene expression spearman correlations.

p < 0.05

Variable	By Variable	Spearman ρ	Prob > |ρ|
Ifnγ	Candidatus Saccharimonas	0.366	0.079
Ifnγ	Candidatus Arthromitus (SFB)	0.383	0.065
Ifnγ	Parvibacter	0.561	**0.004**
Ifnγ	(Enterobacteriaceae) undefined genus	0.083	0.701
Ifnγ	(Lachnospiraceae) undefined genus	-0.413	**0.045**
Ifnγ	Anaerotruncus	-0.343	0.101
Ifnγ	(Lachnospiraceae) GCA-900066575	-0.263	0.215
Ifnγ	Parabacteroides	-0.184	0.389
Ifnβ	Candidatus Saccharimonas	0.207	0.332
Ifnβ	Candidatus Arthromitus (SFB)	0.290	0.170
Ifnβ	Parvibacter	0.410	**0.047**
Ifnβ	(Enterobacteriaceae) undefined genus	0.136	0.527
Ifnβ	(Lachnospiraceae) undefined genus	-0.437	**0.033**
Ifnβ	Anaerotruncus	-0.266	0.209
Ifnβ	(Lachnospiraceae) GCA-900066575	-0.106	0.622
Ifnβ	Parabacteroides	-0.164	0.445
Isg15	Candidatus Saccharimonas	0.502	**0.015**
Isg15	Candidatus Arthromitus (SFB)	0.476	**0.022**
Isg15	Parvibacter	0.326	0.129
Isg15	(Enterobacteriaceae) undefined genus	-0.606	**0.002**
Isg15	(Lachnospiraceae) undefined genus	-0.012	0.957
Isg15	Anaerotruncus	-0.418	**0.047**
Isg15	(Lachnospiraceae) GCA-900066575	-0.517	**0.012**
Isg15	Parabacteroides	-0.493	**0.017**
Il1β	Candidatus Saccharimonas	0.637	**0.001**
Il1β	Candidatus Arthromitus (SFB)	0.769	**<.0001**
Il1β	Parvibacter	0.644	**0.001**
Il1β	(Enterobacteriaceae) undefined genus	-0.436	**0.033**
Il1β	(Lachnospiraceae) undefined genus	-0.463	**0.023**
Il1β	Anaerotruncus	-0.324	0.123
Il1β	(Lachnospiraceae) GCA-900066575	-0.778	**<.0001**
Il1β	Parabacteroides	-0.620	**0.001**
Il6	Candidatus Saccharimonas	0.427	**0.037**
Il6	Candidatus Arthromitus (SFB)	0.537	**0.007**
Il6	Parvibacter	0.599	**0.002**
Il6	(Enterobacteriaceae) undefined genus	-0.172	0.422
Il6	(Lachnospiraceae) undefined genus	-0.501	**0.013**
Il6	Anaerotruncus	-0.466	**0.022**
Il6	(Lachnospiraceae) GCA-900066575	-0.431	**0.036**
Il6	Parabacteroides	-0.341	0.104
Tnfαα	Candidatus Saccharimonas	0.189	0.377
Tnfα	Candidatus Arthromitus (SFB)	0.142	0.509
Tnfα	Parvibacter	0.310	0.141
Tnfα	(Enterobacteriaceae) undefined genus	0.163	0.448
Tnfα	(Lachnospiraceae) undefined genus	-0.112	0.602
Tnfα	Anaerotruncus	-0.345	0.099
Tnfα	(Lachnospiraceae) GCA-900066575	-0.135	0.530
Tnfα	Parabacteroides	-0.204	0.340
Ahr	Candidatus Saccharimonas	0.553	**0.005**
Ahr	Candidatus Arthromitus (SFB)	0.569	**0.004**
Ahr	Parvibacter	0.428	**0.037**
Ahr	(Enterobacteriaceae) undefined genus	-0.422	**0.040**
Ahr	(Lachnospiraceae) undefined genus	-0.208	0.329
Ahr	Anaerotruncus	-0.414	**0.044**
Ahr	(Lachnospiraceae) GCA-900066575	-0.657	**0.001**
Ahr	Parabacteroides	-0.572	**0.004**
Muc2	Candidatus Saccharimonas	0.405	**0.044**
Muc2	Candidatus Arthromitus (SFB)	0.531	**0.006**
Muc2	Parvibacter	0.377	0.063
Muc2	(Enterobacteriaceae) undefined genus	-0.448	**0.025**
Muc2	(Lachnospiraceae) undefined genus	-0.278	0.179
Muc2	Anaerotruncus	-0.286	0.166
Muc2	(Lachnospiraceae) GCA-900066575	-0.532	**0.006**
Muc2	Parabacteroides	-0.434	**0.030**

Supplementary Table 3. d21 Ileum CLR-transformed significant DA and lung gene expression spearman correlations.

Variable	By Variable	Spearman ρ	Prob > |ρ|
Ifnγ	Candidatus Saccharimonas	0.301	0.162
Ifnγ	Candidatus Arthromitus (SFB)	0.061	0.781
Ifnγ	Parvibacter	0.058	0.792
Ifnγ	(Enterobacteriaceae) undefined genus	-0.266	0.220
Ifnγ	(Lachnospiraceae) undefined genus	-0.009	0.968
Ifnγ	Anaerotruncus	-0.016	0.943
Ifnγ	(Lachnospiraceae) GCA-900066575	-0.143	0.514
Ifnγ	Parabacteroides	-0.195	0.373
Ifnβ	Candidatus Saccharimonas	0.244	0.252
Ifnβ	Candidatus Arthromitus (SFB)	0.085	0.692
Ifnβ	Parvibacter	0.094	0.663
Ifnβ	(Enterobacteriaceae) undefined genus	-0.051	0.812
Ifnβ	(Lachnospiraceae) undefined genus	-0.128	0.552
Ifnβ	Anaerotruncus	0.154	0.473
Ifnβ	(Lachnospiraceae) GCA-900066575	0.119	0.579
Ifnβ	Parabacteroides	-0.130	0.546
Isg15	Candidatus Saccharimonas	0.262	0.217
Isg15	Candidatus Arthromitus (SFB)	0.384	0.064
Isg15	Parvibacter	0.095	0.660
Isg15	(Enterobacteriaceae) undefined genus	-0.470	**0.020**
Isg15	(Lachnospiraceae) undefined genus	-0.055	0.799
Isg15	Anaerotruncus	-0.059	0.784
Isg15	(Lachnospiraceae) GCA-900066575	-0.386	0.062
Isg15	Parabacteroides	-0.190	0.375
Il1β	Candidatus Saccharimonas	0.323	0.124
Il1β	Candidatus Arthromitus (SFB)	0.358	0.086
Il1β	Parvibacter	0.015	0.945
Il1β	(Enterobacteriaceae) undefined genus	-0.358	0.086
Il1β	(Lachnospiraceae) undefined genus	0.023	0.917
Il1β	Anaerotruncus	-0.020	0.926
Il1β	(Lachnospiraceae) GCA-900066575	-0.057	0.790
Il1β	Parabacteroides	-0.133	0.535
Il6	Candidatus Saccharimonas	0.085	0.700
Il6	Candidatus Arthromitus (SFB)	0.021	0.925
Il6	Parvibacter	-0.186	0.396
Il6	(Enterobacteriaceae) undefined genus	-0.292	0.177
Il6	(Lachnospiraceae) undefined genus	0.127	0.565
Il6	Anaerotruncus	0.174	0.427
Il6	(Lachnospiraceae) GCA-900066575	0.065	0.768
Il6	Parabacteroides	-0.027	0.904
Tnfα	Candidatus Saccharimonas	0.297	0.159
Tnfα	Candidatus Arthromitus (SFB)	0.323	0.124
Tnfα	Parvibacter	0.322	0.125
Tnfα	(Enterobacteriaceae) undefined genus	-0.133	0.535
Tnfα	(Lachnospiraceae) undefined genus	-0.084	0.695
Tnfα	Anaerotruncus	0.050	0.815
Tnfα	(Lachnospiraceae) GCA-900066575	-0.175	0.414
Tnfα	Parabacteroides	-0.476	**0.019**
Ahr	Candidatus Saccharimonas	0.653	**0.001**
Ahr	Candidatus Arthromitus (SFB)	0.442	**0.031**
Ahr	Parvibacter	0.364	0.081
Ahr	(Enterobacteriaceae) undefined genus	-0.211	0.322
Ahr	(Lachnospiraceae) undefined genus	-0.364	0.081
Ahr	Anaerotruncus	-0.480	**0.018**
Ahr	(Lachnospiraceae) GCA-900066575	-0.562	**0.004**
Ahr	Parabacteroides	-0.254	0.231
Muc5ac	Candidatus Saccharimonas	0.312	0.129
Muc5ac	Candidatus Arthromitus (SFB)	0.294	0.154
Muc5ac	Parvibacter	0.338	0.099
Muc5ac	(Enterobacteriaceae) undefined genus	-0.415	**0.039**
Muc5ac	(Lachnospiraceae) undefined genus	-0.140	0.505
Muc5ac	Anaerotruncus	-0.137	0.514
Muc5ac	(Lachnospiraceae) GCA-900066575	-0.299	0.147
Muc5ac	Parabacteroides	-0.342	0.094
Muc5b	Candidatus Saccharimonas	0.293	0.165
Muc5b	Candidatus Arthromitus (SFB)	0.272	0.198
Muc5b	Parvibacter	0.249	0.241
Muc5b	(Enterobacteriaceae) undefined genus	-0.374	0.072
Muc5b	(Lachnospiraceae) undefined genus	-0.077	0.722
Muc5b	Anaerotruncus	0.010	0.965
Muc5b	(Lachnospiraceae) GCA-900066575	-0.060	0.781
Muc5b	Parabacteroides	-0.262	0.217

$p < 0.05$

Supplementary Table 4. d21 Colon CLR-transformed significant DA and lung gene expression spearman correlations.

Variable	By Variable	Spearman ρ	Prob > |ρ|
Ifnγ	Candidatus Saccharimonas	0.401	0.058
Ifnγ	(Clostridia) undefined genus	0.168	0.444
Ifnγ	Rikenella	-0.289	0.182
Ifnγ	(Prevotellaceae) undefined	-0.004	0.986
Ifnγ	(Erysipelotrichaceae) undefined	0.082	0.710
Ifnγ	(Oscillospirales) UCG-010	0.335	0.118
Ifnγ	(Christensenellaceae) uncultured	-0.269	0.215
Ifnγ	Coriobacteriaceae UCG-002	-0.218	0.317
Ifnγ	(Christensenellaceae) undefined	-0.355	0.097
Ifnγ	(Oscillospiraceae) uncultured	0.272	0.210
Ifnγ	[Eubacterium] brachy group	-0.107	0.628
Ifnβ	Candidatus Saccharimonas	0.378	0.068
Ifnβ	(Clostridia) undefined genus	0.144	0.501
Ifnβ	Rikenella	0.040	0.853
Ifnβ	(Prevotellaceae) undefined	0.097	0.651
Ifnβ	(Erysipelotrichaceae) undefined	0.183	0.393
Ifnβ	(Oscillospirales) UCG-010	0.196	0.360
Ifnβ	(Christensenellaceae) uncultured	0.024	0.913
Ifnβ	Coriobacteriaceae UCG-002	0.161	0.453
Ifnβ	(Christensenellaceae) undefined	0.097	0.651
Ifnβ	(Oscillospiraceae) uncultured	-0.043	0.843
Ifnβ	[Eubacterium] brachy group	-0.204	0.340
Isg15	Candidatus Saccharimonas	0.389	0.061
Isg15	(Clostridia) undefined genus	-0.068	0.753
Isg15	Rikenella	-0.448	**0.028**
Isg15	(Prevotellaceae) undefined	-0.170	0.428
Isg15	(Erysipelotrichaceae) undefined	0.110	0.610
Isg15	(Oscillospirales) UCG-010	-0.004	0.987
Isg15	(Christensenellaceae) uncultured	-0.470	**0.021**
Isg15	Coriobacteriaceae UCG-002	-0.455	**0.026**
Isg15	(Christensenellaceae) undefined	-0.563	**0.004**
Isg15	(Oscillospiraceae) uncultured	0.300	0.154
Isg15	[Eubacterium] brachy group	0.119	0.579
Il1β	Candidatus Saccharimonas	0.524	**0.009**
Il1β	(Clostridia) undefined genus	-0.210	0.326
Il1β	Rikenella	-0.317	0.132
Il1β	(Prevotellaceae) undefined	-0.123	0.568
Il1β	(Erysipelotrichaceae) undefined	0.239	0.260
Il1β	(Oscillospirales) UCG-010	-0.129	0.549
Il1β	(Christensenellaceae) uncultured	-0.357	0.086
Il1β	Coriobacteriaceae UCG-002	-0.337	0.107
Il1β	(Christensenellaceae) undefined	-0.460	**0.024**
Il1β	(Oscillospiraceae) uncultured	0.097	0.651
Il1β	[Eubacterium] brachy group	0.086	0.689
Il6	Candidatus Saccharimonas	0.296	0.171
Il6	(Clostridia) undefined genus	0.004	0.986
Il6	Rikenella	-0.388	0.067
Il6	(Prevotellaceae) undefined	-0.025	0.911
Il6	(Erysipelotrichaceae) undefined	-0.057	0.795
Il6	(Oscillospirales) UCG-010	0.152	0.488
Il6	(Christensenellaceae) uncultured	-0.430	**0.041**
Il6	Coriobacteriaceae UCG-002	-0.319	0.138
Il6	(Christensenellaceae) undefined	-0.479	**0.021**
Il6	(Oscillospiraceae) uncultured	0.187	0.394
Il6	[Eubacterium] brachy group	0.047	0.830
Tnfα	Candidatus Saccharimonas	0.271	0.200
Tnfα	(Clostridia) undefined genus	0.283	0.181
Tnfα	Rikenella	0.292	0.166
Tnfα	(Prevotellaceae) undefined	0.170	0.426
Tnfα	(Erysipelotrichaceae) undefined	-0.104	0.628

***p* < 0.05**

Supplementary Table 4. d21 Colon CLR-transformed significant DA and lung gene expression spearman correlations.

Tnfα	(Oscillospirales) UCG-010	0.308	0.143
Tnfα	(Christensenellaceae) uncultured	0.236	0.268
Tnfα	Coriobacteriaceae UCG-002	0.451	**0.027**
Tnfα	(Christensenellaceae) undefined	0.222	0.298
Tnfα	(Oscillospiraceae) uncultured	0.107	0.619
Tnfα	[Eubacterium] brachy group	-0.148	0.491
Ahr	Candidatus Saccharimonas	0.443	**0.030**
Ahr	(Clostridia) undefined genus	0.222	0.298
Ahr	Rikenella	-0.208	0.330
Ahr	(Prevotellaceae) undefined	-0.135	0.530
Ahr	(Erysipelotrichaceae) undefined	0.357	0.087
Ahr	(Oscillospirales) UCG-010	0.046	0.831
Ahr	(Christensenellaceae) uncultured	-0.193	0.366
Ahr	Coriobacteriaceae UCG-002	-0.315	0.134
Ahr	(Christensenellaceae) undefined	-0.330	0.115
Ahr	(Oscillospiraceae) uncultured	0.344	0.099
Ahr	[Eubacterium] brachy group	-0.275	0.194
Muc5ac	Candidatus Saccharimonas	0.096	0.648
Muc5ac	(Clostridia) undefined genus	0.185	0.375
Muc5ac	Rikenella	0.105	0.616
Muc5ac	(Prevotellaceae) undefined	-0.102	0.627
Muc5ac	(Erysipelotrichaceae) undefined	0.309	0.134
Muc5ac	(Oscillospirales) UCG-010	0.057	0.787
Muc5ac	(Christensenellaceae) uncultured	0.063	0.765
Muc5ac	Coriobacteriaceae UCG-002	0.175	0.404
Muc5ac	(Christensenellaceae) undefined	0.076	0.718
Muc5ac	(Oscillospiraceae) uncultured	0.127	0.546
Muc5ac	[Eubacterium] brachy group	-0.280	0.175
Muc5b	Candidatus Saccharimonas	0.205	0.336
Muc5b	(Clostridia) undefined genus	0.130	0.546
Muc5b	Rikenella	0.100	0.642
Muc5b	(Prevotellaceae) undefined	-0.123	0.568
Muc5b	(Erysipelotrichaceae) undefined	0.397	0.055
Muc5b	(Oscillospirales) UCG-010	-0.020	0.926
Muc5b	(Christensenellaceae) uncultured	0.050	0.818
Muc5b	Coriobacteriaceae UCG-002	0.211	0.322
Muc5b	(Christensenellaceae) undefined	0.108	0.616
Muc5b	(Oscillospiraceae) uncultured	0.161	0.453
Muc5b	[Eubacterium] brachy group	-0.217	0.308

Supplementary Table 5. d35 Ileum CLR-transformed significant DA and ileum gene expression spearman correlations.

p < 0.05

Variable	By Variable	Spearman ρ	Prob > |ρ|
Ifnγ	[Eubacterium] coprostanoligenes group	0.409	0.116
Ifnγ	Parabacteroides	0.159	0.557
Ifnγ	Oscillibacter	0.497	0.050
Ifnγ	Colidextribacter	0.550	**0.027**
Ifnγ	Candidatus Arthromitus (SFB)	0.712	**0.002**
Ifnγ	[Eubacterium] xylanophilum group	0.279	0.295
Ifnγ	(Oscillospiraceae) undefined genus	0.527	**0.036**
Ifnγ	(Lachnospiraceae) NK4A136 group	0.377	0.151
Ifnγ	(Ruminococcaceae) undefined genus	0.477	0.062
Ifnγ	Tuzzerella	0.544	**0.029**
Ifnγ	(Lachnospiraceae) GCA-900066575	0.312	0.240
Ifnγ	(Lachnospiraceae) FCS020 group	0.374	0.154
Ifnγ	Enterorhabdus	-0.641	**0.007**
Ifnγ	(Muribaculaceae) undefined genus	-0.532	**0.034**
Ifnγ	Lactobacillus	-0.488	0.055
Ifnγ	(Anaerovoraceae) Family XIII UCG-001	-0.271	0.311
Ifnγ	Streptococcus	-0.647	**0.007**
Ifnγ	Enterococcus	-0.538	**0.032**
Ifnγ	Staphylococcus	-0.547	**0.028**
Ifnγ	Faecalibaculum	-0.597	**0.015**
Ifnβ	[Eubacterium] coprostanoligenes group	0.527	**0.036**
Ifnβ	Parabacteroides	0.250	0.350
Ifnβ	Oscillibacter	0.512	**0.043**
Ifnβ	Colidextribacter	0.565	**0.023**
Ifnβ	Candidatus Arthromitus (SFB)	0.638	**0.008**
Ifnβ	[Eubacterium] xylanophilum group	0.232	0.387
Ifnβ	(Oscillospiraceae) undefined genus	0.494	0.052
Ifnβ	(Lachnospiraceae) NK4A136 group	0.382	0.144
Ifnβ	(Ruminococcaceae) undefined genus	0.491	0.053
Ifnβ	Tuzzerella	0.550	**0.027**
Ifnβ	(Lachnospiraceae) GCA-900066575	0.341	0.196
Ifnβ	(Lachnospiraceae) FCS020 group	0.344	0.192
Ifnβ	Enterorhabdus	-0.735	**0.001**
Ifnβ	(Muribaculaceae) undefined genus	-0.653	**0.006**
Ifnβ	Lactobacillus	-0.615	**0.011**
Ifnβ	(Anaerovoraceae) Family XIII UCG-001	-0.209	0.438
Ifnβ	Streptococcus	-0.735	**0.001**
Ifnβ	Enterococcus	-0.635	**0.008**
Ifnβ	Staphylococcus	-0.650	**0.006**
Ifnβ	Faecalibaculum	-0.532	**0.034**
Isg15	[Eubacterium] coprostanoligenes group	0.558	**0.020**
Isg15	Parabacteroides	0.597	**0.011**
Isg15	Oscillibacter	0.607	**0.010**
Isg15	Colidextribacter	0.766	**0.000**
Isg15	Candidatus Arthromitus (SFB)	0.646	**0.005**
Isg15	[Eubacterium] xylanophilum group	0.705	**0.002**
Isg15	(Oscillospiraceae) undefined genus	0.678	**0.003**
Isg15	(Lachnospiraceae) NK4A136 group	0.553	**0.021**
Isg15	(Ruminococcaceae) undefined genus	0.690	**0.002**
Isg15	Tuzzerella	0.632	**0.007**
Isg15	(Lachnospiraceae) GCA-900066575	0.627	**0.007**
Isg15	(Lachnospiraceae) FCS020 group	0.565	**0.018**
Isg15	Enterorhabdus	-0.584	**0.014**
Isg15	(Muribaculaceae) undefined genus	-0.716	**0.001**
Isg15	Lactobacillus	-0.766	**0.000**
Isg15	(Anaerovoraceae) Family XIII UCG-001	-0.411	0.101
Isg15	Streptococcus	-0.818	**<.0001**
Isg15	Enterococcus	-0.715	**0.001**
Isg15	Staphylococcus	-0.801	**0.000**
Isg15	Faecalibaculum	-0.533	**0.027**
Il1β	[Eubacterium] coprostanoligenes group	0.395	0.117
Il1β	Parabacteroides	0.382	0.130
Il1β	Oscillibacter	0.284	0.269
Il1β	Colidextribacter	0.478	0.052
Il1β	Candidatus Arthromitus (SFB)	0.753	**0.001**
Il1β	[Eubacterium] xylanophilum group	0.588	**0.013**
Il1β	(Oscillospiraceae) undefined genus	0.456	0.066
Il1β	(Lachnospiraceae) NK4A136 group	0.390	0.122
Il1β	(Ruminococcaceae) undefined genus	0.490	**0.046**
Il1β	Tuzzerella	0.458	0.064
Il1β	(Lachnospiraceae) GCA-900066575	0.436	0.080
Il1β	(Lachnospiraceae) FCS020 group	0.279	0.277
Il1β	Enterorhabdus	-0.689	**0.002**
Il1β	(Muribaculaceae) undefined genus	-0.679	**0.003**
Il1β	Lactobacillus	-0.480	0.051
Il1β	(Anaerovoraceae) Family XIII UCG-001	-0.316	0.216
Il1β	Streptococcus	-0.804	**0.000**
Il1β	Enterococcus	-0.583	**0.014**
Il1β	Staphylococcus	-0.608	**0.010**
Il1β	Faecalibaculum	-0.517	**0.034**

Supplementary Table 5. d35 Ileum CLR-transformed significant DA and ileum gene expression spearman correlations.

Il6	[Eubacterium] coprostanoligenes group	0.329	0.213
Il6	Parabacteroides	0.006	0.983
Il6	Oscillibacter	0.332	0.209
Il6	Colidextribacter	0.388	0.137
Il6	Candidatus Arthromitus (SFB)	0.709	**0.002**
Il6	[Eubacterium] xylanophilum group	0.077	0.778
Il6	(Oscillospiraceae) undefined genus	0.318	0.231
Il6	(Lachnospiraceae) NK4A136 group	0.177	0.513
Il6	(Ruminococcaceae) undefined genus	0.297	0.264
Il6	Tuzzerella	0.397	0.128
Il6	(Lachnospiraceae) GCA-900066575	0.129	0.633
Il6	(Lachnospiraceae) FCS020 group	0.138	0.610
Il6	Enterorhabdus	-0.629	**0.009**
Il6	(Muribaculaceae) undefined genus	-0.606	**0.013**
Il6	Lactobacillus	-0.535	**0.033**
Il6	(Anaerovoracaceae) Family XIII UCG-001	-0.171	0.528
Il6	Streptococcus	-0.609	**0.012**
Il6	Enterococcus	-0.556	**0.025**
Il6	Staphylococcus	-0.547	**0.028**
Il6	Faecalibaculum	-0.397	0.128
Tnfα	[Eubacterium] coprostanoligenes group	0.077	0.778
Tnfα	Parabacteroides	-0.065	0.812
Tnfα	Oscillibacter	0.091	0.737
Tnfα	Colidextribacter	0.153	0.572
Tnfα	Candidatus Arthromitus (SFB)	0.847	**<.0001**
Tnfα	[Eubacterium] xylanophilum group	0.038	0.888
Tnfα	(Oscillospiraceae) undefined genus	0.038	0.888
Tnfα	(Lachnospiraceae) NK4A136 group	0.003	0.991
Tnfα	(Ruminococcaceae) undefined genus	0.147	0.587
Tnfα	Tuzzerella	0.250	0.350
Tnfα	(Lachnospiraceae) GCA-900066575	-0.044	0.871
Tnfα	(Lachnospiraceae) FCS020 group	-0.115	0.672
Tnfα	Enterorhabdus	-0.527	**0.036**
Tnfα	(Muribaculaceae) undefined genus	-0.518	**0.040**
Tnfα	Lactobacillus	-0.332	0.209
Tnfα	(Anaerovoracaceae) Family XIII UCG-001	-0.159	0.557
Tnfα	Streptococcus	-0.518	**0.040**
Tnfα	Enterococcus	-0.441	0.087
Tnfα	Staphylococcus	-0.400	0.125
Tnfα	Faecalibaculum	-0.174	0.520
Ahr	[Eubacterium] coprostanoligenes group	0.708	**0.002**
Ahr	Parabacteroides	0.596	**0.012**
Ahr	Oscillibacter	0.733	**0.001**
Ahr	Colidextribacter	0.833	**<.0001**
Ahr	Candidatus Arthromitus (SFB)	0.539	**0.026**
Ahr	[Eubacterium] xylanophilum group	0.574	**0.016**
Ahr	(Oscillospiraceae) undefined genus	0.735	**0.001**
Ahr	(Lachnospiraceae) NK4A136 group	0.623	**0.008**
Ahr	(Ruminococcaceae) undefined genus	0.789	**0.000**
Ahr	Tuzzerella	0.703	**0.002**
Ahr	(Lachnospiraceae) GCA-900066575	0.613	**0.009**
Ahr	(Lachnospiraceae) FCS020 group	0.578	**0.015**
Ahr	Enterorhabdus	-0.694	**0.002**
Ahr	(Muribaculaceae) undefined genus	-0.694	**0.002**
Ahr	Lactobacillus	-0.650	**0.005**
Ahr	(Anaerovoracaceae) Family XIII UCG-001	-0.397	0.115
Ahr	Streptococcus	-0.873	**<.0001**
Ahr	Enterococcus	-0.735	**0.001**
Ahr	Staphylococcus	-0.772	**0.000**
Ahr	Faecalibaculum	-0.711	**0.001**
Muc2	[Eubacterium] coprostanoligenes group	0.427	0.100
Muc2	Parabacteroides	0.271	0.311
Muc2	Oscillibacter	0.256	0.339
Muc2	Colidextribacter	0.468	0.068
Muc2	Candidatus Arthromitus (SFB)	0.774	**0.000**
Muc2	[Eubacterium] xylanophilum group	0.394	0.131
Muc2	(Oscillospiraceae) undefined genus	0.415	0.110
Muc2	(Lachnospiraceae) NK4A136 group	0.341	0.196
Muc2	(Ruminococcaceae) undefined genus	0.421	0.105
Muc2	Tuzzerella	0.482	0.059
Muc2	(Lachnospiraceae) GCA-900066575	0.403	0.122
Muc2	(Lachnospiraceae) FCS020 group	0.224	0.405
Muc2	Enterorhabdus	-0.550	**0.027**
Muc2	(Muribaculaceae) undefined genus	-0.615	**0.011**
Muc2	Lactobacillus	-0.512	**0.043**
Muc2	(Anaerovoracaceae) Family XIII UCG-001	-0.291	0.274
Muc2	Streptococcus	-0.641	**0.007**
Muc2	Enterococcus	-0.415	0.110
Muc2	Staphylococcus	-0.468	0.068
Muc2	Faecalibaculum	-0.394	0.131

Supplementary Table 6. d35 Ileum CLR-transformed significant DA and lung gene expression spearman correlations.

Variable	By Variable	Spearman ρ	Prob > |ρ|
Ifnγ	[Eubacterium] coprostanoligenes group	0.047	0.863
Ifnγ	Parabacteroides	0.000	1.000
Ifnγ	Oscillibacter	0.244	0.362
Ifnγ	Colidextribacter	0.244	0.362
Ifnγ	Candidatus Arthromitus (SFB)	0.229	0.393
Ifnγ	[Eubacterium] xylanophilum group	-0.174	0.520
Ifnγ	(Oscillospiraceae) undefined genus	0.132	0.625
Ifnγ	(Lachnospiraceae) NK4A136 group	0.018	0.948
Ifnγ	(Ruminococcaceae) undefined genus	0.259	0.333
Ifnγ	Tuzzerella	0.415	0.110
Ifnγ	(Lachnospiraceae) GCA-900066575	-0.106	0.696
Ifnγ	(Lachnospiraceae)FCS020 group	-0.053	0.846
Ifnγ	Enterorhabdus	0.206	0.444
Ifnγ	(Muribaculaceae) undefined genus	0.012	0.966
Ifnγ	Lactobacillus	0.100	0.713
Ifnγ	(Anaerovoracaceae) Family XIII UCG-001	-0.365	0.165
Ifnγ	Streptococcus	0.174	0.520
Ifnγ	Enterococcus	0.009	0.974
Ifnγ	Staphylococcus	0.103	0.704
Ifnγ	Faecalibaculum	-0.209	0.438
Ifnβ	[Eubacterium] coprostanoligenes group	-0.103	0.694
Ifnβ	Parabacteroides	0.086	0.743
Ifnβ	Oscillibacter	0.003	0.993
Ifnβ	Colidextribacter	0.039	0.881
Ifnβ	Candidatus Arthromitus (SFB)	-0.086	0.743
Ifnβ	[Eubacterium] xylanophilum group	-0.226	0.384
Ifnβ	(Oscillospiraceae) undefined genus	-0.052	0.845
Ifnβ	(Lachnospiraceae) NK4A136 group	-0.091	0.729
Ifnβ	(Ruminococcaceae) undefined genus	-0.015	0.955
Ifnβ	Tuzzerella	0.194	0.457
Ifnβ	(Lachnospiraceae) GCA-900066575	-0.199	0.445
Ifnβ	(Lachnospiraceae)FCS020 group	-0.191	0.462
Ifnβ	Enterorhabdus	0.471	0.057
Ifnβ	(Muribaculaceae) undefined genus	0.309	0.228
Ifnβ	Lactobacillus	0.375	0.138
Ifnβ	(Anaerovoracaceae) Family XIII UCG-001	-0.054	0.837
Ifnβ	Streptococcus	0.348	0.171
Ifnβ	Enterococcus	0.326	0.202
Ifnβ	Staphylococcus	0.382	0.130
Ifnβ	Faecalibaculum	-0.118	0.653
Isg15	[Eubacterium] coprostanoligenes group	-0.537	**0.026**
Isg15	Parabacteroides	-0.196	0.451
Isg15	Oscillibacter	-0.218	0.400
Isg15	Colidextribacter	-0.390	0.122
Isg15	Candidatus Arthromitus (SFB)	0.181	0.486
Isg15	[Eubacterium] xylanophilum group	-0.240	0.353
Isg15	(Oscillospiraceae) undefined genus	-0.451	0.069
Isg15	(Lachnospiraceae) NK4A136 group	-0.453	0.068
Isg15	(Ruminococcaceae) undefined genus	-0.223	0.390
Isg15	Tuzzerella	0.003	0.993
Isg15	(Lachnospiraceae) GCA-900066575	-0.547	**0.023**
Isg15	(Lachnospiraceae)FCS020 group	-0.414	0.098
Isg15	Enterorhabdus	0.439	0.078
Isg15	(Muribaculaceae) undefined genus	0.456	0.066
Isg15	Lactobacillus	0.483	**0.050**
Isg15	(Anaerovoracaceae) Family XIII UCG-001	0.005	0.985
Isg15	Streptococcus	0.380	0.133
Isg15	Enterococcus	0.346	0.174
Isg15	Staphylococcus	0.436	0.080
Isg15	Faecalibaculum	0.441	0.076
Il1β	[Eubacterium] coprostanoligenes group	0.125	0.633
Il1β	Parabacteroides	0.351	0.168
Il1β	Oscillibacter	0.449	0.071
Il1β	Colidextribacter	0.284	0.269
Il1β	Candidatus Arthromitus (SFB)	0.253	0.328

$p < 0.05$

Supplementary Table 6. d35 Ileum CLR-transformed significant DA and lung gene expression spearman correlations.

Il1β	[Eubacterium] xylanophilum group	0.328	0.198
Il1β	(Oscillospiraceae) undefined genus	0.240	0.353
Il1β	(Lachnospiraceae) NK4A136 group	0.213	0.411
Il1β	(Ruminococcaceae) undefined genus	0.453	0.068
Il1β	Tuzzerella	0.319	0.213
Il1β	(Lachnospiraceae) GCA-900066575	0.196	0.451
Il1β	(Lachnospiraceae)FCS020 group	0.275	0.286
Il1β	Enterorhabdus	-0.098	0.708
Il1β	(Muribaculaceae) undefined genus	-0.292	0.256
Il1β	Lactobacillus	-0.431	0.084
Il1β	(Anaerovoracaceae) Family XIII UCG-001	-0.191	0.462
Il1β	Streptococcus	-0.157	0.548
Il1β	Enterococcus	-0.392	0.120
Il1β	Staphylococcus	-0.392	0.120
Il1β	Faecalibaculum	0.025	0.926
Il6	[Eubacterium] coprostanoligenes group	-0.481	0.051
Il6	Parabacteroides	-0.244	0.345
Il6	Oscillibacter	-0.195	0.453
Il6	Colidextribacter	-0.326	0.201
Il6	Candidatus Arthromitus (SFB)	-0.074	0.779
Il6	[Eubacterium] xylanophilum group	-0.269	0.297
Il6	(Oscillospiraceae) undefined genus	-0.332	0.193
Il6	(Lachnospiraceae) NK4A136 group	-0.313	0.222
Il6	(Ruminococcaceae) undefined genus	-0.329	0.198
Il6	Tuzzerella	-0.151	0.563
Il6	(Lachnospiraceae) GCA-900066575	-0.481	0.051
Il6	(Lachnospiraceae)FCS020 group	-0.321	0.209
Il6	Enterorhabdus	0.660	**0.004**
Il6	(Muribaculaceae) undefined genus	0.446	0.073
Il6	Lactobacillus	0.438	0.079
Il6	(Anaerovoracaceae) Family XIII UCG-001	0.086	0.743
Il6	Streptococcus	0.595	**0.012**
Il6	Enterococcus	0.457	0.065
Il6	Staphylococcus	0.524	**0.031**
Il6	Faecalibaculum	0.240	0.353
Tnfα	[Eubacterium] coprostanoligenes group	0.462	0.072
Tnfα	Parabacteroides	0.147	0.587
Tnfα	Oscillibacter	0.259	0.333
Tnfα	Colidextribacter	0.418	0.108
Tnfα	Candidatus Arthromitus (SFB)	-0.177	0.513
Tnfα	[Eubacterium] xylanophilum group	-0.027	0.923
Tnfα	(Oscillospiraceae) undefined genus	0.485	0.057
Tnfα	(Lachnospiraceae) NK4A136 group	0.300	0.259
Tnfα	(Ruminococcaceae) undefined genus	0.279	0.295
Tnfα	Tuzzerella	0.306	0.249
Tnfα	(Lachnospiraceae) GCA-900066575	0.297	0.264
Tnfα	(Lachnospiraceae)FCS020 group	0.274	0.305
Tnfα	Enterorhabdus	-0.215	0.425
Tnfα	(Muribaculaceae) undefined genus	-0.256	0.339
Tnfα	Lactobacillus	-0.259	0.333
Tnfα	(Anaerovoracaceae) Family XIII UCG-001	0.012	0.966
Tnfα	Streptococcus	-0.132	0.625
Tnfα	Enterococcus	-0.194	0.471
Tnfα	Staphylococcus	-0.209	0.438
Tnfα	Faecalibaculum	-0.579	**0.019**
Ahr	[Eubacterium] coprostanoligenes group	-0.277	0.282
Ahr	Parabacteroides	0.118	0.653
Ahr	Oscillibacter	0.096	0.715
Ahr	Colidextribacter	-0.147	0.573
Ahr	Candidatus Arthromitus (SFB)	-0.108	0.680
Ahr	[Eubacterium] xylanophilum group	0.201	0.439
Ahr	(Oscillospiraceae) undefined genus	-0.125	0.633
Ahr	(Lachnospiraceae) NK4A136 group	0.015	0.955
Ahr	(Ruminococcaceae) undefined genus	0.047	0.859
Ahr	Tuzzerella	-0.169	0.516
Ahr	(Lachnospiraceae) GCA-900066575	-0.044	0.867

Supplementary Table 6. d35 Ileum CLR-transformed significant DA and lung gene expression spearman correlations.

Ahr	(Lachnospiraceae)FCS020 group	0.088	0.736
Ahr	Enterorhabdus	0.216	0.406
Ahr	(Muribaculaceae) undefined genus	0.140	0.593
Ahr	Lactobacillus	0.017	0.948
Ahr	(Anaerovoracaceae) Family XIII UCG-001	-0.020	0.941
Ahr	Streptococcus	0.189	0.468
Ahr	Enterococcus	-0.020	0.941
Ahr	Staphylococcus	-0.020	0.941
Ahr	Faecalibaculum	0.277	0.282
Muc5ac	[Eubacterium] coprostanoligenes group	-0.282	0.273
Muc5ac	Parabacteroides	-0.299	0.244
Muc5ac	Oscillibacter	-0.483	**0.050**
Muc5ac	Colidextribacter	-0.402	0.110
Muc5ac	Candidatus Arthromitus (SFB)	-0.203	0.434
Muc5ac	[Eubacterium] xylanophilum group	-0.162	0.535
Muc5ac	(Oscillospiraceae) undefined genus	-0.378	0.135
Muc5ac	(Lachnospiraceae) NK4A136 group	-0.137	0.599
Muc5ac	(Ruminococcaceae) undefined genus	-0.436	0.080
Muc5ac	Tuzzerella	-0.503	**0.040**
Muc5ac	(Lachnospiraceae) GCA-900066575	-0.282	0.273
Muc5ac	(Lachnospiraceae)FCS020 group	-0.279	0.277
Muc5ac	Enterorhabdus	0.355	0.162
Muc5ac	(Muribaculaceae) undefined genus	0.378	0.135
Muc5ac	Lactobacillus	0.650	**0.005**
Muc5ac	(Anaerovoracaceae) Family XIII UCG-001	-0.304	0.236
Muc5ac	Streptococcus	0.328	0.198
Muc5ac	Enterococcus	0.547	**0.023**
Muc5ac	Staphylococcus	0.574	**0.016**
Muc5ac	Faecalibaculum	0.108	0.680
Muc5b	[Eubacterium] coprostanoligenes group	-0.241	0.368
Muc5b	Parabacteroides	-0.203	0.451
Muc5b	Oscillibacter	-0.206	0.444
Muc5b	Colidextribacter	-0.203	0.451
Muc5b	Candidatus Arthromitus (SFB)	-0.177	0.513
Muc5b	[Eubacterium] xylanophilum group	-0.353	0.180
Muc5b	(Oscillospiraceae) undefined genus	-0.374	0.154
Muc5b	(Lachnospiraceae) NK4A136 group	-0.194	0.471
Muc5b	(Ruminococcaceae) undefined genus	-0.115	0.672
Muc5b	Tuzzerella	-0.138	0.610
Muc5b	(Lachnospiraceae) GCA-900066575	-0.188	0.485
Muc5b	(Lachnospiraceae)FCS020 group	-0.318	0.231
Muc5b	Enterorhabdus	0.344	0.192
Muc5b	(Muribaculaceae) undefined genus	0.353	0.180
Muc5b	Lactobacillus	0.685	**0.003**
Muc5b	(Anaerovoracaceae) Family XIII UCG-001	-0.171	0.528
Muc5b	Streptococcus	0.353	0.180
Muc5b	Enterococcus	0.400	0.125
Muc5b	Staphylococcus	0.521	**0.039**
Muc5b	Faecalibaculum	0.144	0.594

Supplementary Table 7. d35 Colon CLR-transformed significant DA and lung gene expression spearman correlations.

Variable	By Variable	Spearman ρ	Prob > |ρ|
Ifnγ	Candidatus Arthromitus (SFB)	0.200	0.458
Ifnγ	Romboutsia	0.291	0.274
Ifnγ	Muribaculum	-0.021	0.940
Ifnγ	Streptococcus	0.021	0.940
Ifnγ	Lactobacillus	0.129	0.633
Ifnγ	(Clostridia) undefined genus	0.344	0.192
Ifnγ	Enterorhabdus	-0.059	0.829
Ifnγ	(Lachnospiraceae) uncultured	0.174	0.520
Ifnγ	Blautia	0.259	0.333
Ifnγ	(Peptococcaceae) uncultured	-0.085	0.754
Ifnγ	(Oscillospiraceae) undefined	0.174	0.520
Ifnγ	(Lachnospiraceae) undefined	0.179	0.506
Ifnγ	[Eubacterium] brachy group	0.335	0.204
Ifnγ	Tuzzerella	0.188	0.485
Ifnγ	(Lachnospiraceae) FCS020 group	0.147	0.587
Ifnγ	(Oscillospiraceae) uncultured	0.027	0.923
Ifnγ	(Lachnospiraceae) GCA-9000066575	0.197	0.465
Ifnγ	(Lachnospiraceae) NK4A136 group	0.218	0.418
Ifnγ	Anaerotruncus	-0.071	0.795
Ifnγ	Intestinimonas	0.059	0.829
Ifnγ	(Erysipelotrichaceae) uncultured	0.221	0.412
Ifnγ	(Ruminococcaceae) undefined	-0.359	0.172
Ifnγ	Colidextribacter	0.238	0.374
Ifnγ	Oscillibacter	0.171	0.528
Ifnγ	(Ruminococcaceae) uncultured	-0.197	0.465
Ifnγ	[Eubacterium] ventriosum group	0.215	0.425
Ifnγ	Alistipes	-0.579	**0.019**
Ifnγ	Parabacteroides	0.041	0.880
Ifnβ	Candidatus Arthromitus (SFB)	0.007	0.978
Ifnβ	Romboutsia	0.093	0.722
Ifnβ	Muribaculum	-0.186	0.474
Ifnβ	Streptococcus	-0.233	0.369
Ifnβ	Lactobacillus	-0.302	0.240
Ifnβ	(Clostridia) undefined genus	0.056	0.830
Ifnβ	Enterorhabdus	-0.145	0.580
Ifnβ	(Lachnospiraceae) uncultured	0.439	0.078
Ifnβ	Blautia	0.324	0.205
Ifnβ	(Peptococcaceae) uncultured	0.118	0.653
Ifnβ	(Oscillospiraceae) undefined	0.245	0.343
Ifnβ	(Lachnospiraceae) undefined	0.451	0.069
Ifnβ	[Eubacterium] brachy group	0.373	0.141
Ifnβ	Tuzzerella	0.328	0.198
Ifnβ	(Lachnospiraceae) FCS020 group	0.142	0.586
Ifnβ	(Oscillospiraceae) uncultured	0.292	0.256
Ifnβ	(Lachnospiraceae) GCA-9000066575	0.265	0.305
Ifnβ	(Lachnospiraceae) NK4A136 group	0.402	0.110
Ifnβ	Anaerotruncus	0.159	0.541
Ifnβ	Intestinimonas	0.287	0.264
Ifnβ	(Erysipelotrichaceae) uncultured	0.194	0.457
Ifnβ	(Ruminococcaceae) undefined	-0.140	0.593
Ifnβ	Colidextribacter	0.439	0.078
Ifnβ	Oscillibacter	0.272	0.291
Ifnβ	(Ruminococcaceae) uncultured	0.037	0.889
Ifnβ	[Eubacterium] ventriosum group	0.336	0.188
Ifnβ	Alistipes	-0.358	0.159
Ifnβ	Parabacteroides	0.319	0.213
Isg15	Candidatus Arthromitus (SFB)	-0.277	0.282
Isg15	Romboutsia	-0.093	0.722
Isg15	Muribaculum	-0.431	0.084
Isg15	Streptococcus	-0.529	**0.029**
Isg15	Lactobacillus	-0.515	**0.035**
Isg15	(Clostridia) undefined genus	-0.424	0.090
Isg15	Enterorhabdus	-0.525	**0.031**

***p* < 0.05**

Supplementary Table 7. d35 Colon CLR-transformed significant DA and lung gene expression spearman correlations.

Isg15	(Lachnospiraceae) uncultured	0.253	0.328
Isg15	Blautia	0.233	0.369
Isg15	(Peptococcaceae) uncultured	0.324	0.205
Isg15	(Oscillospiraceae) undefined	0.255	0.324
Isg15	(Lachnospiraceae) undefined	0.392	0.120
Isg15	[Eubacterium] brachy group	0.559	**0.020**
Isg15	Tuzzerella	0.439	0.078
Isg15	(Lachnospiraceae) FCS020 group	0.181	0.486
Isg15	(Oscillospiraceae) uncultured	0.306	0.232
Isg15	(Lachnospiraceae) GCA-9000066575	0.110	0.674
Isg15	(Lachnospiraceae) NK4A136 group	0.360	0.155
Isg15	Anaerotruncus	0.201	0.439
Isg15	Intestinimonas	0.444	0.075
Isg15	(Erysipelotrichaceae) uncultured	0.039	0.881
Isg15	(Ruminococcaceae) undefined	0.331	0.195
Isg15	Colidextribacter	0.583	**0.014**
Isg15	Oscillibacter	0.314	0.220
Isg15	(Ruminococcaceae) uncultured	0.302	0.240
Isg15	[Eubacterium] ventriosum group	0.069	0.794
Isg15	Alistipes	0.392	0.120
Isg15	Parabacteroides	0.414	0.098
Il1β	Candidatus Arthromitus (SFB)	0.101	0.701
Il1β	Romboutsia	0.473	0.055
Il1β	Muribaculum	-0.083	0.751
Il1β	Streptococcus	0.253	0.328
Il1β	Lactobacillus	0.262	0.309
Il1β	(Clostridia) undefined genus	0.284	0.269
Il1β	Enterorhabdus	0.088	0.736
Il1β	(Lachnospiraceae) uncultured	-0.338	0.184
Il1β	Blautia	-0.174	0.504
Il1β	(Peptococcaceae) uncultured	-0.169	0.516
Il1β	(Oscillospiraceae) undefined	0.022	0.933
Il1β	(Lachnospiraceae) undefined	-0.186	0.474
Il1β	[Eubacterium] brachy group	-0.167	0.523
Il1β	Tuzzerella	-0.360	0.155
Il1β	(Lachnospiraceae) FCS020 group	-0.098	0.708
Il1β	(Oscillospiraceae) uncultured	-0.078	0.765
Il1β	(Lachnospiraceae) GCA-9000066575	-0.174	0.504
Il1β	(Lachnospiraceae) NK4A136 group	-0.157	0.548
Il1β	Anaerotruncus	-0.228	0.379
Il1β	Intestinimonas	-0.110	0.674
Il1β	(Erysipelotrichaceae) uncultured	-0.199	0.445
Il1β	(Ruminococcaceae) undefined	-0.147	0.573
Il1β	Colidextribacter	-0.081	0.758
Il1β	Oscillibacter	-0.255	0.324
Il1β	(Ruminococcaceae) uncultured	-0.272	0.291
Il1β	[Eubacterium] ventriosum group	-0.230	0.374
Il1β	Alistipes	0.172	0.510
Il1β	Parabacteroides	-0.184	0.480
Il6	Candidatus Arthromitus (SFB)	-0.260	0.314
Il6	Romboutsia	-0.096	0.715
Il6	Muribaculum	-0.471	0.056
Il6	Streptococcus	-0.513	**0.035**
Il6	Lactobacillus	-0.502	**0.040**
Il6	(Clostridia) undefined genus	-0.315	0.218
Il6	Enterorhabdus	-0.449	0.071
Il6	(Lachnospiraceae) uncultured	0.304	0.235
Il6	Blautia	0.397	0.114
Il6	(Peptococcaceae) uncultured	0.453	0.068
Il6	(Oscillospiraceae) undefined	0.197	0.448
Il6	(Lachnospiraceae) undefined	0.430	0.085
Il6	[Eubacterium] brachy group	0.407	0.105
Il6	Tuzzerella	0.399	0.113
Il6	(Lachnospiraceae) FCS020 group	0.222	0.392

Supplementary Table 7. d35 Colon CLR-transformed significant DA and lung gene expression spearman correlations.

Il6	(Oscillospiraceae) uncultured	0.567	**0.018**
Il6	(Lachnospiraceae) GCA-9000066575	0.271	0.293
Il6	(Lachnospiraceae) NK4A136 group	0.418	0.095
Il6	Anaerotruncus	0.281	0.275
Il6	Intestinimonas	0.504	**0.039**
Il6	(Erysipelotrichaceae) uncultured	0.504	**0.039**
Il6	(Ruminococcaceae) undefined	0.028	0.914
Il6	Colidextribacter	0.521	**0.032**
Il6	Oscillibacter	0.403	0.108
Il6	(Ruminococcaceae) uncultured	0.283	0.271
Il6	[Eubacterium] ventriosum group	0.548	**0.023**
Il6	Alistipes	0.007	0.978
Il6	Parabacteroides	0.583	**0.014**
Tnfα	Candidatus Arthromitus (SFB)	0.318	0.231
Tnfα	Romboutsia	0.244	0.362
Tnfα	Muribaculum	0.347	0.188
Tnfα	Streptococcus	0.447	0.083
Tnfα	Lactobacillus	0.409	0.116
Tnfα	(Clostridia) undefined genus	0.568	**0.022**
Tnfα	Enterorhabdus	0.450	0.080
Tnfα	(Lachnospiraceae) uncultured	0.150	0.579
Tnfα	Blautia	0.159	0.557
Tnfα	(Peptococcaceae) uncultured	-0.471	0.066
Tnfα	(Oscillospiraceae) undefined	-0.006	0.983
Tnfα	(Lachnospiraceae) undefined	-0.132	0.625
Tnfα	[Eubacterium] brachy group	-0.218	0.418
Tnfα	Tuzzerella	-0.221	0.412
Tnfα	(Lachnospiraceae) FCS020 group	-0.038	0.888
Tnfα	(Oscillospiraceae) uncultured	-0.174	0.520
Tnfα	(Lachnospiraceae) GCA-9000066575	0.094	0.729
Tnfα	(Lachnospiraceae) NK4A136 group	-0.050	0.854
Tnfα	Anaerotruncus	-0.224	0.405
Tnfα	Intestinimonas	-0.265	0.322
Tnfα	(Erysipelotrichaceae) uncultured	-0.029	0.914
Tnfα	(Ruminococcaceae) undefined	-0.329	0.213
Tnfα	Colidextribacter	-0.303	0.254
Tnfα	Oscillibacter	-0.109	0.688
Tnfα	(Ruminococcaceae) uncultured	-0.379	0.147
Tnfα	[Eubacterium] ventriosum group	-0.015	0.957
Tnfα	Alistipes	-0.768	**0.001**
Tnfα	Parabacteroides	-0.229	0.393
Ahr	Candidatus Arthromitus (SFB)	-0.201	0.439
Ahr	Romboutsia	0.179	0.492
Ahr	Muribaculum	-0.351	0.168
Ahr	Streptococcus	-0.140	0.593
Ahr	Lactobacillus	-0.186	0.474
Ahr	(Clostridia) undefined genus	-0.101	0.701
Ahr	Enterorhabdus	-0.213	0.411
Ahr	(Lachnospiraceae) uncultured	-0.152	0.560
Ahr	Blautia	0.003	0.993
Ahr	(Peptococcaceae) uncultured	0.255	0.324
Ahr	(Oscillospiraceae) undefined	0.078	0.765
Ahr	(Lachnospiraceae) undefined	0.137	0.599
Ahr	[Eubacterium] brachy group	0.010	0.970
Ahr	Tuzzerella	-0.103	0.694
Ahr	(Lachnospiraceae) FCS020 group	0.066	0.801
Ahr	(Oscillospiraceae) uncultured	0.245	0.343
Ahr	(Lachnospiraceae) GCA-9000066575	-0.042	0.874
Ahr	(Lachnospiraceae) NK4A136 group	0.007	0.978
Ahr	Anaerotruncus	0.142	0.586
Ahr	Intestinimonas	0.226	0.384
Ahr	(Erysipelotrichaceae) uncultured	0.003	0.993
Ahr	(Ruminococcaceae) undefined	0.083	0.751
Ahr	Colidextribacter	0.186	0.474

Supplementary Table 7. d35 Colon CLR-transformed significant DA and lung gene expression spearman correlations.

Ahr	Oscillibacter	-0.042	0.874
Ahr	(Ruminococcaceae) uncultured	0.123	0.639
Ahr	[Eubacterium] ventriosum group	0.005	0.985
Ahr	Alistipes	0.495	**0.043**
Ahr	Parabacteroides	0.088	0.736
Muc5ac	Candidatus Arthromitus (SFB)	-0.091	0.729
Muc5ac	Romboutsia	-0.311	0.224
Muc5ac	Muribaculum	-0.047	0.859
Muc5ac	Streptococcus	-0.348	0.171
Muc5ac	Lactobacillus	-0.277	0.282
Muc5ac	(Clostridia) undefined genus	-0.412	0.101
Muc5ac	Enterorhabdus	-0.309	0.228
Muc5ac	(Lachnospiraceae) uncultured	0.191	0.462
Muc5ac	Blautia	0.118	0.653
Muc5ac	(Peptococcaceae) uncultured	0.453	0.068
Muc5ac	(Oscillospiraceae) undefined	0.145	0.580
Muc5ac	(Lachnospiraceae) undefined	0.235	0.363
Muc5ac	[Eubacterium] brachy group	0.578	**0.015**
Muc5ac	Tuzzerella	0.517	**0.034**
Muc5ac	(Lachnospiraceae) FCS020 group	0.157	0.548
Muc5ac	(Oscillospiraceae) uncultured	0.240	0.353
Muc5ac	(Lachnospiraceae) GCA-9000066575	0.189	0.468
Muc5ac	(Lachnospiraceae) NK4A136 group	0.289	0.260
Muc5ac	Anaerotruncus	0.378	0.135
Muc5ac	Intestinimonas	0.297	0.248
Muc5ac	(Erysipelotrichaceae) uncultured	0.338	0.184
Muc5ac	(Ruminococcaceae) undefined	0.032	0.903
Muc5ac	Colidextribacter	0.306	0.232
Muc5ac	Oscillibacter	0.328	0.198
Muc5ac	(Ruminococcaceae) uncultured	0.414	0.098
Muc5ac	[Eubacterium] ventriosum group	0.493	**0.045**
Muc5ac	Alistipes	0.052	0.845
Muc5ac	Parabacteroides	0.294	0.252
Muc5b	Candidatus Arthromitus (SFB)	-0.121	0.656
Muc5b	Romboutsia	-0.082	0.762
Muc5b	Muribaculum	-0.203	0.451
Muc5b	Streptococcus	-0.388	0.137
Muc5b	Lactobacillus	-0.297	0.264
Muc5b	(Clostridia) undefined genus	-0.088	0.745
Muc5b	Enterorhabdus	-0.318	0.231
Muc5b	(Lachnospiraceae) uncultured	0.291	0.274
Muc5b	Blautia	0.309	0.245
Muc5b	(Peptococcaceae) uncultured	0.518	**0.040**
Muc5b	(Oscillospiraceae) undefined	0.197	0.465
Muc5b	(Lachnospiraceae) undefined	0.553	**0.026**
Muc5b	[Eubacterium] brachy group	0.547	**0.028**
Muc5b	Tuzzerella	0.541	**0.030**
Muc5b	(Lachnospiraceae) FCS020 group	0.371	0.158
Muc5b	(Oscillospiraceae) uncultured	0.185	0.492
Muc5b	(Lachnospiraceae) GCA-9000066575	0.435	0.092
Muc5b	(Lachnospiraceae) NK4A136 group	0.285	0.284
Muc5b	Anaerotruncus	0.368	0.161
Muc5b	Intestinimonas	0.271	0.311
Muc5b	(Erysipelotrichaceae) uncultured	0.456	0.076
Muc5b	(Ruminococcaceae) undefined	-0.062	0.820
Muc5b	Colidextribacter	0.382	0.144
Muc5b	Oscillibacter	0.318	0.231
Muc5b	(Ruminococcaceae) uncultured	0.321	0.226
Muc5b	[Eubacterium] ventriosum group	0.409	0.116
Muc5b	Alistipes	-0.227	0.399
Muc5b	Parabacteroides	0.162	0.550

Supplementary Table 8. Primer Sequences.

Primer	Sequence (5'-3')
Ifnβ_F	CAGCTCCAAGAAAGGACGAAC
Ifnβ_R	GGCAGTGTAACTCTTCTGCAT
Ifnγ_F	GGTCAACAACCCACAGGTCC
Ifnγ_R	ACTCCTTTTCCGCTTCCTGAG
Isg15_F	CATCCTGGTGAGGAACGAAAGG
Isg15_R	CTCAGCCAGAACTGGTCTTCGT
Il1β_F	GCACTACAGGCTCCGAGATGAAC
Il1β_R	TTGTCGTTGCTTGGTTCTCCTTGT
Il6_F	GACTGATGCTGGTGACAAC
Il6_R	ATCCTCTGTGAAGTCTCCTC
Tnfα_F	AGGCACTCCCCCAAAAGATG
Tnfα_R	GTAGACAGAAGAGCGTGGTGG
Ahr_F	GCAGAATCCCACATCCGCA
Ahr_R	CCTTCTTCATCCGTCAGTGGTC
Muc2_F	ACCTACCACAAGACCACCCT
Muc2_R	GAATGTCCCCCAAGACGAGG
Muc5ac_F	CCACTTTCTCCTTCTCCACACC
Muc5ac_R	GGTTGTCGATGCAGCCTTGCTT
Muc5b_F	CTGAAGACCTGTCGGAACCCAA
Muc5b_R	GCCACACACTTCATCTGGTCCT
(Housekeeping Gene) Eef2_F	TGTCAGTCATCGCCCATGTG
(Housekeeping Gene) Eef2_R	GGAGATGGCGGTGGATTTGA

Supplementary Gene Primers	Sequence (5'-3')
Il10_F	GCTGAAGACCCTCAGGATGCG
Il10_R	CCTGCTCCACTGCCTTGCTCT
Tgfβ1_F	AGCGGACTACTATGCTAAAGAGGTC
Tgfβ1_R	TCTCATAGATGGCGTTGTTGC
Foxp3_F	CCTGGTTGTGAGAAGGTCTTCG
Foxp3_R	TGCTCCAGAGACTGCACCACTT
Il4_F	ATCATCGGCATTTTGAACGAGGTC
Il4_R	ACCTTGGAAGCCCTACAGACGA
Il17a_F	ACTACCTCAACCGTTCCA
Il17a_R	GAGGGATATCTATCAGGGTC
Il18_F	CCTGAAGAAAATGGAGACCTGG
Il18_R	TCATATCCTCGAACACAGGCT
Il5_F	GATGAGGCTTCCTGTCCCTACT
Il5_R	TGACAGGTTTTGGAATAGCATTTCC
Il13_F	AACGGCAGCATGGTATGGAGTG
Il13_R	TGGGTCCTGTAGATGGCATTGC

Supplementary File 1. Offspring Lung Gene Expression Analysis

```
library(tidyverse)
library(lme4)
library(lmerTest)
library(emmeans)
```

Lung Tissue Gene Expression–Figure 1

Read in Lung Gene Expression Data

```
lung_dat <- read_csv("LG_expression.csv")
```

```
## Rows: 82 Columns: 22
## ── Column specification ────────────────────────────────────────────────────────
## Delimiter: ","
## chr  (5): SampleID, Treatment, Timepoint, Sex, Litter
## dbl (17): Ahr, Foxp3, Ifng, Ifnb, IL1b, IL4, IL5, IL6, IL10, IL13, IL18, Isg...
##
## ℹ Use `spec()` to retrieve the full column specification for this data.
## ℹ Specify the column types or set `show_col_types = FALSE` to quiet this message.
```

```
summary(lung_dat)
```

```
##    SampleID          Treatment          Timepoint             Sex
##  Length:82          Length:82          Length:82          Length:82
##  Class :character   Class :character   Class :character   Class :character
##  Mode  :character   Mode  :character   Mode  :character   Mode  :character
##
##
##
##
##     Litter               Ahr             Foxp3             Ifng
##  Length:82          Min.   : 0.380   Min.   : 0.451   Min.   : 0.295
##  Class :character   1st Qu.: 2.361   1st Qu.: 2.118   1st Qu.: 1.848
##  Mode  :character   Median : 4.217   Median :18.358   Median : 6.965
##                     Mean   : 4.400   Mean   :24.887   Mean   : 7.809
##                     3rd Qu.: 6.040   3rd Qu.:42.418   3rd Qu.:11.950
##                     Max.   :17.757   Max.   :94.688   Max.   :26.395
##                     NA's   :1        NA's   :2        NA's   :5
##       Ifnb             IL1b            IL4              IL5
##  Min.   : 0.159   Min.   :0.230   Min.   :0.0010   Min.   : 0.363
##  1st Qu.: 1.663   1st Qu.:0.761   1st Qu.:0.0680   1st Qu.: 0.984
##  Median : 5.931   Median :1.176   Median :0.1930   Median : 1.368
##  Mean   : 9.765   Mean   :1.284   Mean   :0.5272   Mean   : 2.297
##  3rd Qu.:14.370   3rd Qu.:1.438   3rd Qu.:0.7670   3rd Qu.: 2.785
##  Max.   :43.907   Max.   :4.443   Max.   :3.4540   Max.   :10.533
##  NA's   :2        NA's   :3       NA's   :9        NA's   :5
##       IL6             IL10             IL13             IL18
##  Min.   :0.294   Min.   : 0.415   Min.   : 0.342   Min.   :0.485
##  1st Qu.:1.226   1st Qu.: 1.647   1st Qu.: 1.133   1st Qu.:1.235
##  Median :1.998   Median : 7.111   Median : 1.952   Median :1.865
##  Mean   :2.303   Mean   :10.226   Mean   : 7.561   Mean   :2.583
##  3rd Qu.:2.853   3rd Qu.:13.111   3rd Qu.: 6.242   3rd Qu.:3.881
##  Max.   :7.529   Max.   :51.551   Max.   :63.822   Max.   :6.769
##  NA's   :4       NA's   :3        NA's   :3        NA's   :2
##      Isg15            Muc5ac           Muc5b             Tgfb1
##  Min.   :0.3300   Min.   : 0.275   Min.   :  0.247   Min.   :0.464
##  1st Qu.:0.6695   1st Qu.: 1.564   1st Qu.:  1.808   1st Qu.:1.213
##  Median :1.2020   Median :12.445   Median : 34.468   Median :2.125
##  Mean   :1.3620   Mean   :16.475   Mean   : 51.664   Mean   :2.208
##  3rd Qu.:1.7188   3rd Qu.:28.388   3rd Qu.:103.851   3rd Qu.:2.868
##  Max.   :4.3870   Max.   :71.952   Max.   :199.789   Max.   :5.323
##  NA's   :2        NA's   :1        NA's   :3         NA's   :1
##       Tnf             IL17
##  Min.   :0.391   Min.   : 0.056
##  1st Qu.:1.153   1st Qu.: 0.394
##  Median :1.933   Median : 4.942
##  Mean   :2.095   Mean   :10.669
##  3rd Qu.:2.564   3rd Qu.:17.004
##  Max.   :4.998   Max.   :73.810
##  NA's   :2       NA's   :10
```

```
# prep data
lung_dat$Timepoint <- factor(lung_dat$Timepoint, levels = c("d0", "d7", "d21", "d35"))
lung_dat$Treatment <- factor(lung_dat$Treatment, levels = c("Control", "PNS"))
lung_dat$Litter <- factor(lung_dat$Litter, levels = c("F5", "O4", "R4", "R5", "K6", "B4", "F4", "L4", "K5", "P5", "M6"))
lung_dat$Sex <- factor(lung_dat$Sex, levels = c("F", "M"))
```

Lung Tissue *Ifng* Expression

```
# Fit linear mixed-effects model
# Include random intercept for litter
Ifng_model <- lmer(Ifng ~ Timepoint * Treatment + (1 | Litter), data = lung_dat)
summary(Ifng_model)
```

```
## Linear mixed model fit by REML. t-tests use Satterthwaite's method [
## lmerModLmerTest]
## Formula: Ifng ~ Timepoint * Treatment + (1 | Litter)
##    Data: lung_dat
##
## REML criterion at convergence: 404.3
##
## Scaled residuals:
##     Min      1Q  Median      3Q     Max
## -2.1885 -0.5312  0.0046  0.4083  3.1956
##
## Random effects:
##  Groups   Name        Variance Std.Dev.
##  Litter   (Intercept)  0.9273  0.963
##  Residual             15.1380  3.891
## Number of obs: 77, groups:  Litter, 11
##
## Fixed effects:
##                            Estimate Std. Error      df t value Pr(>|t|)
## (Intercept)                  1.2354     1.3086 44.8037   0.944 0.350211
## Timepointd7                  3.6544     1.9279 63.9267   1.895 0.062551 .
## Timepointd21                 7.1272     1.7479 63.3705   4.078 0.000129 ***
## Timepointd35                11.6887     1.9279 63.9267   6.063 7.94e-08 ***
## TreatmentPNS                -0.4499     1.8052 46.1319  -0.249 0.804297
## Timepointd7:TreatmentPNS    4.3596     2.5782 64.1103   1.691 0.095704 .
## Timepointd21:TreatmentPNS   4.9675     2.3747 64.1585   2.092 0.040421 *
## Timepointd35:TreatmentPNS   0.2385     2.6115 64.2446   0.091 0.927509
## ---
## Signif. codes:  0 '***' 0.001 '**' 0.01 '*' 0.05 '.' 0.1 ' ' 1
##
## Correlation of Fixed Effects:
##             (Intr) Tmpnt7 Tmpn21 Tmpn35 TrtPNS T7:TPN T21:TP
## Timepointd7 -0.601
## Timepontd21 -0.668  0.444
## Timepontd35 -0.601  0.418  0.444
## TreatmntPNS -0.725  0.436  0.484  0.436
## Tmpnt7:TPNS  0.450 -0.748 -0.332 -0.313 -0.627
## Tmpn21:TPNS  0.491 -0.327 -0.736 -0.327 -0.683  0.475
## Tmpn35:TPNS  0.444 -0.309 -0.328 -0.738 -0.613  0.434  0.462
```

```
# Create a type III ANOVA table
Ifng_anova_table <- anova(Ifng_model, ddf = "Kenward-Roger")
print(Ifng_anova_table)
```

```
## Type III Analysis of Variance Table with Kenward-Roger's method
##                     Sum Sq Mean Sq NumDF  DenDF F value Pr(>F)
## Timepoint           1534.77  511.59     3 64.533 33.7909  3e-13 ***
## Treatment             48.09   48.09     1  8.569  3.1770 0.1100
## Timepoint:Treatment   97.30   32.43     3 64.533  2.1423 0.1035
## ---
## Signif. codes:  0 '***' 0.001 '**' 0.01 '*' 0.05 '.' 0.1 ' ' 1
```

```
#Because no interaction, test for main effects of treatment at individual time points
Ifng_emcatcat <- emmeans(Ifng_model, ~ Treatment | Timepoint)
#Is there a difference between treatment groups at each time point?
contrast(Ifng_emcatcat, "revpairwise", adjust="BH")
```

```
## Timepoint = d0:
##  contrast      estimate   SE   df t.ratio p.value
##  PNS - Control   -0.450 1.81 44.5  -0.248  0.8054
##
## Timepoint = d7:
##  contrast      estimate   SE   df t.ratio p.value
##  PNS - Control    3.910 2.03 52.0   1.923  0.0599
##
## Timepoint = d21:
##  contrast      estimate   SE   df t.ratio p.value
##  PNS - Control    4.518 1.75 41.5   2.577  0.0136
##
## Timepoint = d35:
##  contrast      estimate   SE   df t.ratio p.value
##  PNS - Control   -0.211 2.10 49.9  -0.101  0.9203
##
## Degrees-of-freedom method: kenward-roger
```

Lung Tissue *Ifnb* Expression

```
# Fit linear mixed-effects model
# Include random intercept for litter
Ifnb_model <- lmer(Ifnb ~ Timepoint * Treatment + (1 | Litter), data = lung_dat)
summary(Ifnb_model)
```

```
## Linear mixed model fit by REML. t-tests use Satterthwaite's method [
## lmerModLmerTest]
## Formula: Ifnb ~ Timepoint * Treatment + (1 | Litter)
##    Data: lung_dat
##
## REML criterion at convergence: 534.1
##
## Scaled residuals:
##     Min      1Q  Median      3Q     Max
## -2.1828 -0.4262 -0.0466  0.2864  2.7530
##
## Random effects:
##  Groups   Name        Variance Std.Dev.
##  Litter   (Intercept)  4.932   2.221
##  Residual             72.117   8.492
## Number of obs: 80, groups:  Litter, 11
##
## Fixed effects:
##                              Estimate Std. Error     df t value Pr(>|t|)
## (Intercept)                     1.766      2.875 42.483   0.614  0.54222
## Timepointd7                     4.452      4.210 65.800   1.057  0.29417
## Timepointd21                    7.675      3.816 65.008   2.011  0.04846 *
## Timepointd35                   11.899      4.060 66.749   2.931  0.00462 **
## TreatmentPNS                   -1.154      3.965 43.963  -0.291  0.77231
## Timepointd7:TreatmentPNS       16.782      5.568 65.662   3.014  0.00366 **
## Timepointd21:TreatmentPNS       6.578      5.138 65.124   1.280  0.20502
## Timepointd35:TreatmentPNS      -5.148      5.593 66.656  -0.921  0.36063
## ---
## Signif. codes:  0 '***' 0.001 '**' 0.01 '*' 0.05 '.' 0.1 ' ' 1
##
## Correlation of Fixed Effects:
##             (Intr) Tmpnt7 Tmpn21 Tmpn35 TrtPNS T7:TPN T21:TP
## Timepointd7 -0.597
## Timepontd21 -0.663  0.443
## Timepontd35 -0.624  0.434  0.464
## TreatmntPNS -0.725  0.433  0.481  0.452
## Tmpnt7:TPNS  0.452 -0.756 -0.335 -0.328 -0.629
## Tmpn21:TPNS  0.493 -0.329 -0.743 -0.344 -0.684  0.483
## Tmpn35:TPNS  0.453 -0.315 -0.337 -0.726 -0.624  0.448  0.477
```

```
# Create a type III ANOVA table
Ifnb_anova_table <- anova(Ifnb_model, ddf = "Kenward-Roger")
print(Ifnb_anova_table)
```

```
## Type III Analysis of Variance Table with Kenward-Roger's method
##                      Sum Sq Mean Sq NumDF  DenDF F value    Pr(>F)
## Timepoint           1942.94  647.65     3 67.220  8.9799 4.408e-05 ***
## Treatment            146.54  146.54     1  8.691  2.0319  0.188935
## Timepoint:Treatment 1137.98  379.33     3 67.220  5.2595  0.002552 **
## ---
## Signif. codes:  0 '***' 0.001 '**' 0.01 '*' 0.05 '.' 0.1 ' ' 1
```

```
# Because we have an interaction
Ifnb_emcatcat <- emmeans(Ifnb_model, ~ Timepoint * Treatment)

#Is there a difference between treatment groups at each time point?
contrast(Ifnb_emcatcat, "revpairwise", by="Timepoint", adjust="BH")
```

```
## Timepoint = d0:
##  contrast      estimate   SE   df t.ratio p.value
##  PNS - Control    -1.15 3.98 45.8  -0.290  0.7733
##
## Timepoint = d7:
##  contrast      estimate   SE   df t.ratio p.value
##  PNS - Control    15.63 4.38 52.4   3.569  0.0008
##
## Timepoint = d21:
##  contrast      estimate   SE   df t.ratio p.value
##  PNS - Control     5.42 3.79 41.7   1.431  0.1598
##
## Timepoint = d35:
##  contrast      estimate   SE   df t.ratio p.value
##  PNS - Control    -6.30 4.45 49.4  -1.415  0.1633
##
## Degrees-of-freedom method: kenward-roger
```

```
#Is there a difference between time points within each treatment?
contrast(Ifnb_emcatcat, "revpairwise", by="Treatment", adjust="BH")
```

```
## Treatment = Control:
##  contrast  estimate   SE   df t.ratio p.value
##  d7 - d0       4.45 4.23 66.4   1.052  0.3722
##  d21 - d0      7.67 3.83 65.7   2.002  0.1483
##  d21 - d7      3.22 4.30 70.0   0.749  0.4564
##  d35 - d0     11.90 4.09 67.3   2.910  0.0293
##  d35 - d7      7.45 4.41 63.9   1.690  0.1919
##  d35 - d21     4.22 4.13 69.7   1.022  0.3722
##
## Treatment = PNS:
##  contrast  estimate   SE   df t.ratio p.value
##  d7 - d0      21.23 3.67 66.1   5.790  <.0001
##  d21 - d0     14.25 3.46 65.9   4.119  0.0003
##  d21 - d7     -6.98 3.43 63.8  -2.035  0.0583
##  d35 - d0      6.75 3.88 67.1   1.741  0.0862
##  d35 - d7    -14.48 3.93 69.7  -3.683  0.0009
##  d35 - d21    -7.50 3.74 69.7  -2.008  0.0583
##
## Degrees-of-freedom method: kenward-roger
## P value adjustment: BH method for 6 tests
```

Lung Tissue *Isg15* Expression

```
# Fit linear mixed-effects model
# Include random intercept for litter
Isg15_model <- lmer(Isg15 ~ Timepoint * Treatment + (1 | Litter), data = lung_dat)
summary(Isg15_model)
```

```
## Linear mixed model fit by REML. t-tests use Satterthwaite's method [
## lmerModLmerTest]
## Formula: Isg15 ~ Timepoint * Treatment + (1 | Litter)
##    Data: lung_dat
##
## REML criterion at convergence: 122.2
##
## Scaled residuals:
##     Min      1Q  Median      3Q     Max
## -1.9220 -0.5645 -0.0627  0.5334  3.3772
##
## Random effects:
##  Groups   Name        Variance Std.Dev.
##  Litter   (Intercept) 0.07073  0.2660
##  Residual             0.21371  0.4623
## Number of obs: 80, groups:  Litter, 11
##
## Fixed effects:
##                            Estimate Std. Error       df t value Pr(>|t|)
## (Intercept)                 1.09064    0.18958 23.73690   5.753 6.54e-06 ***
## Timepointd7                -0.69007    0.23121 64.30623  -2.985  0.00401 **
## Timepointd21                0.93632    0.20904 63.58604   4.479 3.18e-05 ***
## Timepointd35                0.30413    0.22352 64.84992   1.361  0.17833
## TreatmentPNS               -0.01328    0.26004 24.77041  -0.051  0.95968
## Timepointd7:TreatmentPNS    0.13591    0.30540 63.92632   0.445  0.65780
## Timepointd21:TreatmentPNS  0.53778    0.28134 63.45974   1.911  0.06046 .
## Timepointd35:TreatmentPNS -0.33067    0.30917 65.12296  -1.070  0.28877
## ---
## Signif. codes:  0 '***' 0.001 '**' 0.01 '*' 0.05 '.' 0.1 ' ' 1
##
## Correlation of Fixed Effects:
##             (Intr) Tmpnt7 Tmpn21 Tmpn35 TrtPNS T7:TPN T21:TP
## Timepointd7 -0.490
## Timepontd21 -0.550  0.428
## Timepontd35 -0.515  0.443  0.452
## TreatmntPNS -0.729  0.358  0.401  0.375
## Tmpnt7:TPNS  0.371 -0.757 -0.324 -0.336 -0.523
## Tmpn21:TPNS  0.409 -0.318 -0.743 -0.336 -0.571  0.476
## Tmpn35:TPNS  0.372 -0.320 -0.327 -0.723 -0.518  0.451  0.470
```

```
# Create a type III ANOVA table
Isg15_anova_table <- anova(Isg15_model, ddf = "Kenward-Roger")
print(Isg15_anova_table)
```

```
## Type III Analysis of Variance Table with Kenward-Roger's method
##                     Sum Sq Mean Sq NumDF  DenDF F value Pr(>F)
## Timepoint           33.836 11.2787     3 65.325 52.7739 <2e-16 ***
## Treatment            0.030  0.0299     1  8.953  0.1400 0.7169
## Timepoint:Treatment  1.822  0.6074     3 65.325  2.8422 0.0445 *
## ---
## Signif. codes:  0 '***' 0.001 '**' 0.01 '*' 0.05 '.' 0.1 ' ' 1
```

```
# Because we have an interaction
Isg15_emcatcat <- emmeans(Isg15_model, ~ Timepoint * Treatment)

#Is there a difference between treatment groups at each time point?
contrast(Isg15_emcatcat, "revpairwise", by="Timepoint", adjust="BH")
```

```
## Timepoint = d0:
##  contrast      estimate    SE   df t.ratio p.value
##  PNS - Control  -0.0133 0.260 26.6  -0.051  0.9597
##
## Timepoint = d7:
##  contrast      estimate    SE   df t.ratio p.value
##  PNS - Control   0.1226 0.280 32.2   0.439  0.6639
##
## Timepoint = d21:
##  contrast      estimate    SE   df t.ratio p.value
##  PNS - Control   0.5245 0.252 23.8   2.083  0.0482
##
## Timepoint = d35:
##  contrast      estimate    SE   df t.ratio p.value
##  PNS - Control  -0.3439 0.284 32.5  -1.211  0.2348
##
## Degrees-of-freedom method: kenward-roger
```

```
#Is there a difference between time points within each treatment?
contrast(Isg15_emcatcat, "revpairwise", by="Treatment", adjust="BH")
```

```
## Treatment = Control:
##  contrast  estimate    SE   df t.ratio p.value
##  d7 - d0    -0.6901 0.232 65.0  -2.974  0.0062
##  d21 - d0    0.9363 0.210 64.3   4.468  0.0001
##  d21 - d7    1.6264 0.238 67.4   6.830  <.0001
##  d35 - d0    0.3041 0.225 65.5   1.354  0.1803
##  d35 - d7    0.9942 0.240 63.4   4.140  0.0002
##  d35 - d21  -0.6322 0.229 67.3  -2.765  0.0088
##
## Treatment = PNS:
##  contrast  estimate    SE   df t.ratio p.value
##  d7 - d0    -0.5542 0.200 64.2  -2.770  0.0110
##  d21 - d0    1.4741 0.189 64.1   7.811  <.0001
##  d21 - d7    2.0283 0.187 63.3  10.853  <.0001
##  d35 - d0   -0.0265 0.215 66.0  -0.123  0.9021
##  d35 - d7    0.5276 0.216 66.3   2.442  0.0207
##  d35 - d21  -1.5006 0.205 66.3  -7.309  <.0001
##
## Degrees-of-freedom method: kenward-roger
## P value adjustment: BH method for 6 tests
```

Lung Tissue *Il1b* Expression

```
# Fit linear mixed-effects model
# Include random intercept for litter
IL1b_model <- lmer(IL1b ~ Timepoint * Treatment + (1 | Litter), data = lung_dat)
```

```
## boundary (singular) fit: see help('isSingular')
```

```
summary(IL1b_model)
```

```
## Linear mixed model fit by REML. t-tests use Satterthwaite's method [
## lmerModLmerTest]
## Formula: IL1b ~ Timepoint * Treatment + (1 | Litter)
##    Data: lung_dat
##
## REML criterion at convergence: 162.8
##
## Scaled residuals:
##     Min      1Q  Median      3Q     Max
## -1.8352 -0.6127 -0.0878  0.3435  3.4900
##
## Random effects:
##  Groups   Name        Variance Std.Dev.
##  Litter   (Intercept) 0.0000   0.0000
##  Residual             0.4497   0.6706
## Number of obs: 79, groups:  Litter, 11
##
## Fixed effects:
##                              Estimate Std. Error       df t value Pr(>|t|)
## (Intercept)                   1.22360    0.21206 71.00000   5.770 1.91e-07 ***
## Timepointd7                  -0.64310    0.34629 71.00000  -1.857   0.0674 .
## Timepointd21                  0.15160    0.29989 71.00000   0.506   0.6148
## Timepointd35                 -0.42273    0.31808 71.00000  -1.329   0.1881
## TreatmentPNS                  0.13557    0.28713 71.00000   0.472   0.6383
## Timepointd7:TreatmentPNS      0.36573    0.44984 71.00000   0.813   0.4189
## Timepointd21:TreatmentPNS     0.59188    0.39941 71.00000   1.482   0.1428
## Timepointd35:TreatmentPNS     0.06067    0.43430 71.00000   0.140   0.8893
## ---
## Signif. codes:  0 '***' 0.001 '**' 0.01 '*' 0.05 '.' 0.1 ' ' 1
##
## Correlation of Fixed Effects:
##             (Intr) Tmpnt7 Tmpn21 Tmpn35 TrtPNS T7:TPN T21:TP
## Timepointd7 -0.612
## Timepontd21 -0.707  0.433
## Timepontd35 -0.667  0.408  0.471
## TreatmntPNS -0.739  0.452  0.522  0.492
## Tmpnt7:TPNS  0.471 -0.770 -0.333 -0.314 -0.638
## Tmpn21:TPNS  0.531 -0.325 -0.751 -0.354 -0.719  0.459
## Tmpn35:TPNS  0.488 -0.299 -0.345 -0.732 -0.661  0.422  0.475
## optimizer (nloptwrap) convergence code: 0 (OK)
## boundary (singular) fit: see help('isSingular')
```

```
# Create a type III ANOVA table
IL1b_anova_table <- anova(IL1b_model, ddf = "Kenward-Roger")
print(IL1b_anova_table)
```

```
## Type III Analysis of Variance Table with Kenward-Roger's method
##                     Sum Sq Mean Sq NumDF  DenDF F value    Pr(>F)
## Timepoint          10.0472  3.3491     3 67.325  7.4468 0.0002226 ***
## Treatment           2.7809  2.7809     1  8.678  6.1842 0.0355191 *
## Timepoint:Treatment  1.1994  0.3998     3 67.325  0.8889 0.4514515
## ---
## Signif. codes:  0 '***' 0.001 '**' 0.01 '*' 0.05 '.' 0.1 ' ' 1
```

```
#Because no interaction, test for main effects of treatment at individual time points
IL1b_emcatcat <- emmeans(IL1b_model, ~ Treatment | Timepoint)
#Is there a difference between treatment groups at each time point?
contrast(IL1b_emcatcat, "revpairwise", adjust="BH")
```

```
## Timepoint = d0:
##  contrast      estimate    SE   df t.ratio p.value
##  PNS - Control    0.136 0.289 54.2   0.469  0.6410
##
## Timepoint = d7:
##  contrast      estimate    SE   df t.ratio p.value
##  PNS - Control    0.501 0.350 62.9   1.433  0.1567
##
## Timepoint = d21:
##  contrast      estimate    SE   df t.ratio p.value
##  PNS - Control    0.727 0.279 52.0   2.608  0.0119
##
## Timepoint = d35:
##  contrast      estimate    SE   df t.ratio p.value
##  PNS - Control    0.196 0.332 54.7   0.592  0.5565
##
## Degrees-of-freedom method: kenward-roger
```

Lung Tissue *Il6* Expression

```
# Fit linear mixed-effects model
# Include random intercept for litter
IL6_model <- lmer(IL6 ~ Timepoint * Treatment + (1 | Litter), data = lung_dat)
summary(IL6_model)
```

```
## Linear mixed model fit by REML. t-tests use Satterthwaite's method [
## lmerModLmerTest]
## Formula: IL6 ~ Timepoint * Treatment + (1 | Litter)
##    Data: lung_dat
##
## REML criterion at convergence: 239.8
##
## Scaled residuals:
##     Min      1Q  Median      3Q     Max
## -1.4767 -0.5856 -0.1231  0.5180  3.1594
##
## Random effects:
##  Groups   Name        Variance Std.Dev.
##  Litter   (Intercept) 0.2889   0.5375
##  Residual             1.2308   1.1094
## Number of obs: 78, groups:  Litter, 11
##
## Fixed effects:
##                            Estimate Std. Error      df t value Pr(>|t|)
## (Intercept)                 1.1306     0.4278 28.7429   2.643 0.013171 *
## Timepointd7                 0.8887     0.5537 63.1346   1.605 0.113514
## Timepointd21                1.4641     0.5010 62.4080   2.923 0.004830 **
## Timepointd35                1.8909     0.5350 63.7310   3.534 0.000768 ***
## TreatmentPNS               -0.3804     0.5879 29.9689  -0.647 0.522497
## Timepointd7:TreatmentPNS    1.6988     0.7391 62.7378   2.299 0.024870 *
## Timepointd21:TreatmentPNS   1.2055     0.6817 62.7988   1.768 0.081858 .
## Timepointd35:TreatmentPNS  -0.8572     0.7400 64.1612  -1.158 0.251042
## ---
## Signif. codes:  0 '***' 0.001 '**' 0.01 '*' 0.05 '.' 0.1 ' ' 1
##
## Correlation of Fixed Effects:
##             (Intr) Tmpnt7 Tmpn21 Tmpn35 TrtPNS T7:TPN T21:TP
## Timepointd7 -0.522
## Timepontd21 -0.584  0.432
## Timepontd35 -0.548  0.441  0.455
## TreatmntPNS -0.728  0.380  0.425  0.399
## Tmpnt7:TPNS  0.391 -0.749 -0.323 -0.331 -0.549
## Tmpn21:TPNS  0.430 -0.317 -0.735 -0.334 -0.602  0.469
## Tmpn35:TPNS  0.396 -0.319 -0.329 -0.723 -0.550  0.443  0.465
```

```
# Create a type III ANOVA table
IL6_anova_table <- anova(IL6_model, ddf = "Kenward-Roger")
print(IL6_anova_table)
```

```
## Type III Analysis of Variance Table with Kenward-Roger's method
##                     Sum Sq Mean Sq NumDF  DenDF F value    Pr(>F)
## Timepoint           51.019 17.0065     3 64.004 13.8164 4.758e-07 ***
## Treatment            0.123  0.1225     1  8.836  0.0996  0.759678
## Timepoint:Treatment 16.965  5.6549     3 64.004  4.5942  0.005645 **
## ---
## Signif. codes:  0 '***' 0.001 '**' 0.01 '*' 0.05 '.' 0.1 ' ' 1
```

```
# Because we have an interaction
IL6_emcatcat <- emmeans(IL6_model, ~ Timepoint * Treatment)

#Is there a difference between treatment groups at each time point?
contrast(IL6_emcatcat, "revpairwise", by="Timepoint", adjust="BH")
```

```
## Timepoint = d0:
##  contrast      estimate    SE   df t.ratio p.value
##  PNS - Control   -0.380 0.589 30.5  -0.646  0.5233
##
## Timepoint = d7:
##  contrast      estimate    SE   df t.ratio p.value
##  PNS - Control    1.318 0.647 38.0   2.038  0.0485
##
## Timepoint = d21:
##  contrast      estimate    SE   df t.ratio p.value
##  PNS - Control    0.825 0.574 28.1   1.437  0.1618
##
## Timepoint = d35:
##  contrast      estimate    SE   df t.ratio p.value
##  PNS - Control   -1.238 0.648 36.5  -1.908  0.0642
##
## Degrees-of-freedom method: kenward-roger
```

```
#Is there a difference between time points within each treatment?
contrast(IL6_emcatcat, "revpairwise", by="Treatment", adjust="BH")
```

```
## Treatment = Control:
##  contrast  estimate    SE   df t.ratio p.value
##  d7 - d0     0.8887 0.556 63.3   1.598  0.1725
##  d21 - d0    1.4641 0.503 62.6   2.913  0.0149
##  d21 - d7    0.5754 0.570 66.0   1.010  0.3794
##  d35 - d0    1.8909 0.538 63.9   3.515  0.0049
##  d35 - d7    1.0022 0.576 61.5   1.739  0.1725
##  d35 - d21   0.4268 0.547 65.9   0.780  0.4381
##
## Treatment = PNS:
##  contrast  estimate    SE   df t.ratio p.value
##  d7 - d0     2.5875 0.491 62.4   5.270  <.0001
##  d21 - d0    2.6696 0.465 63.4   5.744  <.0001
##  d21 - d7    0.0821 0.471 62.0   0.174  0.8621
##  d35 - d0    1.0337 0.515 64.8   2.006  0.0589
##  d35 - d7   -1.5538 0.532 65.7  -2.918  0.0072
##  d35 - d21  -1.6359 0.505 66.1  -3.240  0.0037
##
## Degrees-of-freedom method: kenward-roger
## P value adjustment: BH method for 6 tests
```

Lung Tissue *Tnfa* Expression

```
# Fit linear mixed-effects model
# Include random intercept for litter
Tnf_model <- lmer(Tnf ~ Timepoint * Treatment + (1 | Litter), data = lung_dat)
summary(Tnf_model)
```

```
## Linear mixed model fit by REML. t-tests use Satterthwaite's method [
## lmerModLmerTest]
## Formula: Tnf ~ Timepoint * Treatment + (1 | Litter)
##    Data: lung_dat
##
## REML criterion at convergence: 176.4
##
## Scaled residuals:
##      Min       1Q   Median       3Q      Max
## -2.09519 -0.49347  0.00092  0.55031  2.44928
##
## Random effects:
##  Groups   Name        Variance Std.Dev.
##  Litter   (Intercept) 0.05042  0.2245
##  Residual             0.49224  0.7016
## Number of obs: 80, groups:  Litter, 11
##
## Fixed effects:
##                          Estimate Std. Error       df t value Pr(>|t|)
## (Intercept)               1.06365    0.24472 38.91273   4.346 9.63e-05 ***
## Timepointd7              -0.09924    0.34851 65.66089  -0.285   0.7767
## Timepointd21              1.92661    0.31575 64.85552   6.102 6.51e-08 ***
## Timepointd35              0.85249    0.33628 66.50785   2.535   0.0136 *
## TreatmentPNS              0.29783    0.33122 39.03540   0.899   0.3741
## Timepointd7:TreatmentPNS   0.70208    0.45632 65.15314   1.539   0.1287
## Timepointd21:TreatmentPNS  0.17302    0.42007 64.39662   0.412   0.6818
## Timepointd35:TreatmentPNS  0.14628    0.46792 67.18489   0.313   0.7555
## ---
## Signif. codes:  0 '***' 0.001 '**' 0.01 '*' 0.05 '.' 0.1 ' ' 1
##
## Correlation of Fixed Effects:
##             (Intr) Tmpnt7 Tmpn21 Tmpn35 TrtPNS T7:TPN T21:TP
## Timepointd7 -0.579
## Timepontd21 -0.645  0.440
## Timepontd35 -0.605  0.436  0.461
## TreatmntPNS -0.739  0.428  0.476  0.447
## Tmpnt7:TPNS  0.442 -0.764 -0.336 -0.333 -0.602
## Tmpn21:TPNS  0.485 -0.331 -0.752 -0.347 -0.656  0.470
## Tmpn35:TPNS  0.435 -0.313 -0.331 -0.719 -0.587  0.430  0.456
```

```
# Create a type III ANOVA table
Tnf_anova_table <- anova(Tnf_model, ddf = "Kenward-Roger")
print(Tnf_anova_table)
```

```
## Type III Analysis of Variance Table with Kenward-Roger's method
##                     Sum Sq Mean Sq NumDF  DenDF F value    Pr(>F)
## Timepoint           52.186 17.3954     3 66.849 35.3365 8.438e-14 ***
## Treatment            3.337  3.3370     1  8.868  6.7792    0.0289 *
## Timepoint:Treatment  1.255  0.4182     3 66.849  0.8495    0.4717
## ---
## Signif. codes:  0 '***' 0.001 '**' 0.01 '*' 0.05 '.' 0.1 ' ' 1
```

```
#Because no interaction, test for main effects of treatment at individual time points
Tnf_emcatcat <- emmeans(Tnf_model, ~ Treatment | Timepoint)
#Is there a difference between treatment groups at each time point?
contrast(Tnf_emcatcat, "revpairwise", adjust="BH")
```

```
## Timepoint = d0:
##  contrast      estimate    SE   df t.ratio p.value
##  PNS - Control    0.298 0.332 40.5   0.896  0.3754
##
## Timepoint = d7:
##  contrast      estimate    SE   df t.ratio p.value
##  PNS - Control    1.000 0.371 48.7   2.697  0.0096
##
## Timepoint = d21:
##  contrast      estimate    SE   df t.ratio p.value
##  PNS - Control    0.471 0.323 37.7   1.458  0.1531
##
## Timepoint = d35:
##  contrast      estimate    SE   df t.ratio p.value
##  PNS - Control    0.444 0.388 48.4   1.146  0.2575
##
## Degrees-of-freedom method: kenward-roger
```

Lung Tissue *Ahr* Expression

```
# Fit linear mixed-effects model
# Include random intercept for litter
Ahr_model <- lmer(Ahr ~ Timepoint * Treatment + (1 | Litter), data = lung_dat)
summary(Ahr_model)
```

```
## Linear mixed model fit by REML. t-tests use Satterthwaite's method [
## lmerModLmerTest]
## Formula: Ahr ~ Timepoint * Treatment + (1 | Litter)
##    Data: lung_dat
##
## REML criterion at convergence: 327.6
##
## Scaled residuals:
##     Min      1Q  Median      3Q     Max
## -2.0458 -0.4483 -0.0667  0.4021  4.3335
##
## Random effects:
##  Groups   Name        Variance Std.Dev.
##  Litter   (Intercept) 0.4157   0.6447
##  Residual             3.7707   1.9418
## Number of obs: 81, groups:  Litter, 11
##
## Fixed effects:
##                              Estimate Std. Error      df t value Pr(>|t|)
## (Intercept)                    1.1557     0.6818 42.7020   1.695  0.09733 .
## Timepointd7                    2.6388     0.9650 67.8484   2.735  0.00796 **
## Timepointd21                   2.4403     0.8742 67.1769   2.792  0.00682 **
## Timepointd35                   7.4061     0.9312 68.5342   7.953 2.54e-11 ***
## TreatmentPNS                   0.3213     0.9228 42.8131   0.348  0.72938
## Timepointd7:TreatmentPNS       0.6825     1.2634 67.4198   0.540  0.59082
## Timepointd21:TreatmentPNS      2.7248     1.1629 66.7882   2.343  0.02211 *
## Timepointd35:TreatmentPNS     -3.0825     1.2736 68.8336  -2.420  0.01815 *
## ---
## Signif. codes:  0 '***' 0.001 '**' 0.01 '*' 0.05 '.' 0.1 ' ' 1
##
## Correlation of Fixed Effects:
##             (Intr) Tmpnt7 Tmpn21 Tmpn35 TrtPNS T7:TPN T21:TP
## Timepointd7 -0.575
## Timepontd21 -0.641  0.439
## Timepontd35 -0.601  0.437  0.461
## TreatmntPNS -0.739  0.425  0.473  0.444
## Tmpnt7:TPNS  0.439 -0.764 -0.336 -0.333 -0.598
## Tmpn21:TPNS  0.481 -0.330 -0.752 -0.346 -0.651  0.470
## Tmpn35:TPNS  0.440 -0.319 -0.337 -0.731 -0.593  0.439  0.465
```

```
# Create a type III ANOVA table
Ahr_anova_table <- anova(Ahr_model, ddf = "Kenward-Roger")
print(Ahr_anova_table)
```

```
## Type III Analysis of Variance Table with Kenward-Roger's method
##                      Sum Sq Mean Sq NumDF  DenDF F value    Pr(>F)
## Timepoint           340.84 113.612     3 67.635 30.1280 1.746e-12 ***
## Treatment             1.73   1.729     1  8.852  0.4586 0.5155891
## Timepoint:Treatment  79.18  26.394     3 67.635  6.9993 0.0003606 ***
## ---
## Signif. codes:  0 '***' 0.001 '**' 0.01 '*' 0.05 '.' 0.1 ' ' 1
```

```
# Because we have an interaction
Ahr_emcatcat <- emmeans(Ahr_model, ~ Timepoint * Treatment)

#Is there a difference between treatment groups at each time point?
contrast(Ahr_emcatcat, "revpairwise", by="Timepoint", adjust="BH")
```

```
## Timepoint = d0:
##  contrast      estimate    SE   df t.ratio p.value
##  PNS - Control    0.321 0.925 40.1   0.347  0.7303
##
## Timepoint = d7:
##  contrast      estimate    SE   df t.ratio p.value
##  PNS - Control    1.004 1.030 48.5   0.973  0.3354
##
## Timepoint = d21:
##  contrast      estimate    SE   df t.ratio p.value
##  PNS - Control    3.046 0.900 37.3   3.385  0.0017
##
## Timepoint = d35:
##  contrast      estimate    SE   df t.ratio p.value
##  PNS - Control   -2.761 1.050 46.7  -2.631  0.0115
##
## Degrees-of-freedom method: kenward-roger
```

```
#Is there a difference between time points within each treatment?
contrast(Ahr_emcatcat, "revpairwise", by="Treatment", adjust="BH")
```

```
## Treatment = Control:
##  contrast  estimate    SE   df t.ratio p.value
##  d7 - d0      2.639 0.970 67.1   2.721  0.0099
##  d21 - d0     2.440 0.878 66.3   2.780  0.0099
##  d21 - d7    -0.199 0.989 70.4  -0.201  0.8414
##  d35 - d0     7.406 0.937 67.9   7.902  <.0001
##  d35 - d7     4.767 1.010 64.7   4.729  <.0001
##  d35 - d21    4.966 0.949 70.2   5.232  <.0001
##
## Treatment = PNS:
##  contrast  estimate    SE   df t.ratio p.value
##  d7 - d0      3.321 0.819 65.9   4.057  0.0003
##  d21 - d0     5.165 0.769 65.3   6.718  <.0001
##  d21 - d7     1.844 0.785 64.7   2.350  0.0328
##  d35 - d0     4.324 0.877 68.6   4.931  <.0001
##  d35 - d7     1.002 0.901 69.7   1.112  0.3241
##  d35 - d21   -0.842 0.857 69.7  -0.982  0.3293
##
## Degrees-of-freedom method: kenward-roger
## P value adjustment: BH method for 6 tests
```

Lung Tissue *Muc5ac* Expression

```
##Muc5ac
# Fit linear mixed-effects model
Muc5ac_model <- lmer(Muc5ac ~ Timepoint * Treatment + (1 | Litter), data = lung_dat) # include random intercept for litter
summary(Muc5ac_model)
```

```
## Linear mixed model fit by REML. t-tests use Satterthwaite's method [
## lmerModLmerTest]
## Formula: Muc5ac ~ Timepoint * Treatment + (1 | Litter)
##    Data: lung_dat
##
## REML criterion at convergence: 574.4
##
## Scaled residuals:
##     Min      1Q  Median      3Q     Max
## -1.8268 -0.5697 -0.0424  0.3098  3.2625
##
## Random effects:
##  Groups   Name        Variance Std.Dev.
##  Litter   (Intercept)   4.839   2.20
##  Residual             115.178  10.73
## Number of obs: 81, groups:  Litter, 11
##
## Fixed effects:
##                           Estimate Std. Error       df t value Pr(>|t|)
## (Intercept)                 1.0288     3.5440  49.2709   0.290   0.7728
## Timepointd7                 3.4441     5.3102  67.6692   0.649   0.5188
## Timepointd21               10.1487     4.6999  66.1764   2.159   0.0345 *
## Timepointd35               46.3671     5.1180  68.6113   9.060 2.44e-13 ***
## TreatmentPNS                0.1709     4.8850  51.7078   0.035   0.9722
## Timepointd7:TreatmentPNS   14.6279     7.0143  66.7806   2.085   0.0409 *
## Timepointd21:TreatmentPNS  10.1131     6.3891  65.6857   1.583   0.1183
## Timepointd35:TreatmentPNS -18.6030     7.0629  69.6414  -2.634   0.0104 *
## ---
## Signif. codes:  0 '***' 0.001 '**' 0.01 '*' 0.05 '.' 0.1 ' ' 1
##
## Correlation of Fixed Effects:
##             (Intr) Tmpnt7 Tmpn21 Tmpn35 TrtPNS T7:TPN T21:TP
## Timepointd7 -0.613
## Timepontd21 -0.696  0.459
## Timepontd35 -0.639  0.432  0.479
## TreatmntPNS -0.725  0.445  0.505  0.464
## Tmpnt7:TPNS  0.464 -0.757 -0.348 -0.327 -0.643
## Tmpn21:TPNS  0.512 -0.338 -0.736 -0.352 -0.707  0.490
## Tmpn35:TPNS  0.463 -0.313 -0.347 -0.725 -0.641  0.448  0.487
```

```
# Create a type III ANOVA table
Muc5ac_anova_table <- anova(Muc5ac_model, ddf = "Kenward-Roger")
print(Muc5ac_anova_table)
```

```
## Type III Analysis of Variance Table with Kenward-Roger's method
##                     Sum Sq Mean Sq NumDF  DenDF F value    Pr(>F)    
## Timepoint          12811.9  4270.6     3 68.052 37.0748 2.565e-14 ***
## Treatment             42.6    42.6     1  8.705  0.3702 0.5584335    
## Timepoint:Treatment  2811.9   937.3     3 68.052  8.1370 0.0001048 ***
## ---
## Signif. codes:  0 '***' 0.001 '**' 0.01 '*' 0.05 '.' 0.1 ' ' 1
```

```
# Because we have an interaction
Muc5ac_emcatcat <- emmeans(Muc5ac_model, ~ Timepoint * Treatment)
#Is there a difference between treatment groups at each time point?
contrast(Muc5ac_emcatcat, "revpairwise", by="Timepoint", adjust="BH")
```

```
## Timepoint = d0:
##  contrast      estimate   SE   df t.ratio p.value
##  PNS - Control    0.171 4.90 51.4   0.035  0.9723
## 
## Timepoint = d7:
##  contrast      estimate   SE   df t.ratio p.value
##  PNS - Control   14.799 5.42 56.6   2.730  0.0084
## 
## Timepoint = d21:
##  contrast      estimate   SE   df t.ratio p.value
##  PNS - Control   10.284 4.54 44.9   2.266  0.0283
## 
## Timepoint = d35:
##  contrast      estimate   SE   df t.ratio p.value
##  PNS - Control  -18.432 5.51 52.8  -3.346  0.0015
## 
## Degrees-of-freedom method: kenward-roger
```

```
#Is there a difference between time points within each treatment?
contrast(Muc5ac_emcatcat, "revpairwise", by="Treatment", adjust="BH")
```

```
## Treatment = Control:
##  contrast  estimate   SE   df t.ratio p.value
##  d7 - d0       3.44 5.34 67.6   0.645  0.5211
##  d21 - d0     10.15 4.72 66.0   2.151  0.0526
##  d21 - d7      6.70 5.29 70.2   1.268  0.2507
##  d35 - d0     46.37 5.15 68.5   8.995  <.0001
##  d35 - d7     42.92 5.57 65.0   7.709  <.0001
##  d35 - d21    36.22 5.07 70.0   7.140  <.0001
## 
## Treatment = PNS:
##  contrast  estimate   SE   df t.ratio p.value
##  d7 - d0      18.07 4.59 65.4   3.934  0.0004
##  d21 - d0     20.26 4.33 65.0   4.674  <.0001
##  d21 - d7      2.19 4.33 64.9   0.505  0.6151
##  d35 - d0     27.76 4.93 70.6   5.628  <.0001
##  d35 - d7      9.69 4.95 71.3   1.956  0.0815
##  d35 - d21     7.50 4.71 71.2   1.594  0.1385
## 
## Degrees-of-freedom method: kenward-roger 
## P value adjustment: BH method for 6 tests
```

Lung Tissue *Muc5b* Expression

```
# Fit linear mixed-effects model
Muc5b_model <- lmer(Muc5b ~ Timepoint * Treatment + (1 | Litter), data = lung_dat) # include random intercept for litter
```

```
## boundary (singular) fit: see help('isSingular')
```

```
summary(Muc5b_model)
```

```
## Linear mixed model fit by REML. t-tests use Satterthwaite's method [
## lmerModLmerTest]
## Formula: Muc5b ~ Timepoint * Treatment + (1 | Litter)
##    Data: lung_dat
##
## REML criterion at convergence: 665.7
##
## Scaled residuals:
##      Min       1Q   Median       3Q      Max
## -3.02850 -0.36355 -0.01728  0.28007  3.03448
##
## Random effects:
##  Groups   Name        Variance Std.Dev.
##  Litter   (Intercept)   0.0     0.00
##  Residual             535.3    23.14
## Number of obs: 79, groups:  Litter, 11
##
## Fixed effects:
##                            Estimate Std. Error       df t value Pr(>|t|)
## (Intercept)                  1.2039     7.3163  71.0000   0.165   0.8698
## Timepointd7                  0.9468    11.4016  71.0000   0.083   0.9341
## Timepointd21                52.6352    10.3468  71.0000   5.087 2.85e-06 ***
## Timepointd35               142.5580    10.9744  71.0000  12.990  < 2e-16 ***
## TreatmentPNS                 0.3848    10.1089  71.0000   0.038   0.9697
## Timepointd7:TreatmentPNS   13.4630    15.0772  71.0000   0.893   0.3749
## Timepointd21:TreatmentPNS  34.5299    13.9267  71.0000   2.479   0.0155 *
## Timepointd35:TreatmentPNS -27.9152    15.3626  71.0000  -1.817   0.0734 .
## ---
## Signif. codes:  0 '***' 0.001 '**' 0.01 '*' 0.05 '.' 0.1 ' ' 1
##
## Correlation of Fixed Effects:
##             (Intr) Tmpnt7 Tmpn21 Tmpn35 TrtPNS T7:TPN T21:TP
## Timepointd7 -0.642
## Timepontd21 -0.707  0.454
## Timepontd35 -0.667  0.428  0.471
## TreatmntPNS -0.724  0.464  0.512  0.482
## Tmpnt7:TPNS  0.485 -0.756 -0.343 -0.324 -0.670
## Tmpn21:TPNS  0.525 -0.337 -0.743 -0.350 -0.726  0.487
## Tmpn35:TPNS  0.476 -0.306 -0.337 -0.714 -0.658  0.441  0.478
## optimizer (nloptwrap) convergence code: 0 (OK)
## boundary (singular) fit: see help('isSingular')
```

```
# Create a type III ANOVA table
Muc5b_anova_table <- anova(Muc5b_model, ddf = "Kenward-Roger")
print(Muc5b_anova_table)
```

```
## Type III Analysis of Variance Table with Kenward-Roger's method
##                     Sum Sq Mean Sq NumDF  DenDF  F value    Pr(>F)
## Timepoint           186459   62153     3 67.256 116.0926 < 2.2e-16 ***
## Treatment              542     542     1  8.487   1.0127  0.342092
## Timepoint:Treatment   9450    3150     3 67.256   5.8838  0.001253 **
## ---
## Signif. codes:  0 '***' 0.001 '**' 0.01 '*' 0.05 '.' 0.1 ' ' 1
```

```
# Because we have an interaction
Muc5b_emcatcat <- emmeans(Muc5b_model, ~ Timepoint * Treatment)
#Is there a difference between treatment groups at each time point?
contrast(Muc5b_emcatcat, "revpairwise", by="Timepoint", adjust="BH")
```

```
## Timepoint = d0:
##  contrast      estimate    SE   df t.ratio p.value
##  PNS - Control    0.385 10.20 56.5   0.038  0.9699
##
## Timepoint = d7:
##  contrast      estimate    SE   df t.ratio p.value
##  PNS - Control   13.848 11.30 60.0   1.228  0.2244
##
## Timepoint = d21:
##  contrast      estimate    SE   df t.ratio p.value
##  PNS - Control   34.915  9.62 51.9   3.628  0.0007
##
## Timepoint = d35:
##  contrast      estimate    SE   df t.ratio p.value
##  PNS - Control  -27.530 11.80 55.3  -2.335  0.0232
##
## Degrees-of-freedom method: kenward-roger
```

```
#Is there a difference between time points within each treatment?
contrast(Muc5b_emcatcat, "revpairwise", by="Treatment", adjust="BH")
```

```
## Treatment = Control:
##  contrast  estimate    SE   df t.ratio p.value
##  d7 - d0      0.947 11.50 66.0   0.083  0.9345
##  d21 - d0    52.635 10.40 65.5   5.056  <.0001
##  d21 - d7    51.688 11.60 69.9   4.467  <.0001
##  d35 - d0   142.558 11.10 67.1  12.886  <.0001
##  d35 - d7   141.611 12.00 63.2  11.805  <.0001
##  d35 - d21   89.923 11.10 69.5   8.092  <.0001
##
## Treatment = PNS:
##  contrast  estimate    SE   df t.ratio p.value
##  d7 - d0     14.410  9.90 63.8   1.456  0.1504
##  d21 - d0    87.165  9.34 63.3   9.331  <.0001
##  d21 - d7    72.755  9.34 63.3   7.788  <.0001
##  d35 - d0   114.643 11.00 70.2  10.456  <.0001
##  d35 - d7   100.233 11.00 70.8   9.105  <.0001
##  d35 - d21   27.478 10.50 70.5   2.622  0.0128
##
## Degrees-of-freedom method: kenward-roger
## P value adjustment: BH method for 6 tests
```

Supplementary Gene Expression

Lung Tissue *Il10* Expression

```
# Fit linear mixed-effects model
# Include random intercept for litter
IL10_model <- lmer(IL10 ~ Timepoint * Treatment + (1 | Litter), data = lung_dat)
summary(IL10_model)
```

```
## Linear mixed model fit by REML. t-tests use Satterthwaite's method [
## lmerModLmerTest]
## Formula: IL10 ~ Timepoint * Treatment + (1 | Litter)
##    Data: lung_dat
##
## REML criterion at convergence: 462.3
##
## Scaled residuals:
##     Min      1Q  Median      3Q     Max
## -2.9564 -0.4372  0.0094  0.4177  3.9604
##
## Random effects:
##  Groups   Name        Variance Std.Dev.
##  Litter   (Intercept) 15.02    3.875
##  Residual             24.76    4.976
## Number of obs: 79, groups:  Litter, 11
##
## Fixed effects:
##                          Estimate Std. Error       df t value Pr(>|t|)
## (Intercept)               1.36275    2.35305 18.29600   0.579  0.56956
## Timepointd7              24.27959    2.49664 63.04966   9.725 3.65e-14 ***
## Timepointd21              4.99636    2.25471 62.52641   2.216  0.03034 *
## Timepointd35              6.85637    2.41558 63.40217   2.838  0.00608 **
## TreatmentPNS             -0.03755    3.21110 18.85742  -0.012  0.99079
## Timepointd7:TreatmentPNS   1.37174    3.28393 62.59120   0.418  0.67759
## Timepointd21:TreatmentPNS  1.78983    3.04993 62.32368   0.587  0.55943
## Timepointd35:TreatmentPNS -2.21391    3.34664 63.64721  -0.662  0.51066
## ---
## Signif. codes:  0 '***' 0.001 '**' 0.01 '*' 0.05 '.' 0.1 ' ' 1
##
## Correlation of Fixed Effects:
##             (Intr) Tmpnt7 Tmpn21 Tmpn35 TrtPNS T7:TPN T21:TP
## Timepointd7 -0.424
## Timepontd21 -0.478  0.423
## Timepontd35 -0.446  0.447  0.447
## TreatmntPNS -0.733  0.311  0.350  0.327
## Tmpnt7:TPNS  0.323 -0.760 -0.322 -0.340 -0.452
## Tmpn21:TPNS  0.353 -0.313 -0.739 -0.331 -0.490  0.467
## Tmpn35:TPNS  0.322 -0.322 -0.323 -0.722 -0.449  0.449  0.456
```

```
# Create a type III ANOVA table
IL10_anova_table <- anova(IL10_model, ddf = "Kenward-Roger")
print(IL10_anova_table)
```

```
## Type III Analysis of Variance Table with Kenward-Roger's method
##                     Sum Sq Mean Sq NumDF  DenDF F value Pr(>F)
## Timepoint           6224.8 2074.93     3 63.612 83.7871 <2e-16 ***
## Treatment              0.1    0.14     1  8.957  0.0058 0.9410
## Timepoint:Treatment   41.3   13.76     3 63.612  0.5556 0.6463
## ---
## Signif. codes:  0 '***' 0.001 '**' 0.01 '*' 0.05 '.' 0.1 ' ' 1
```

```
#Because no interaction, test for main effects of treatment at individual time points
IL10_emcatcat <- emmeans(IL10_model, ~ Treatment | Timepoint)
#Is there a difference between treatment groups at each time point?
contrast(IL10_emcatcat, "revpairwise", adjust="BH")
```

```
## Timepoint = d0:
##  contrast      estimate   SE   df t.ratio p.value
##  PNS - Control  -0.0375 3.21 19.6  -0.012  0.9908
##
## Timepoint = d7:
##  contrast      estimate   SE   df t.ratio p.value
##  PNS - Control   1.3342 3.41 23.5   0.392  0.6988
##
## Timepoint = d21:
##  contrast      estimate   SE   df t.ratio p.value
##  PNS - Control   1.7523 3.17 18.4   0.553  0.5866
##
## Timepoint = d35:
##  contrast      estimate   SE   df t.ratio p.value
##  PNS - Control  -2.2515 3.45 24.1  -0.652  0.5207
##
## Degrees-of-freedom method: kenward-roger
```

Lung Tissue *Tgfb1* Expression

```
# Fit linear mixed-effects model
# Include random intercept for litter
Tgfb1_model <- lmer(Tgfb1 ~ Timepoint * Treatment + (1 | Litter), data = lung_dat)
summary(Tgfb1_model)
```

```
## Linear mixed model fit by REML. t-tests use Satterthwaite's method [
## lmerModLmerTest]
## Formula: Tgfb1 ~ Timepoint * Treatment + (1 | Litter)
##    Data: lung_dat
##
## REML criterion at convergence: 181.6
##
## Scaled residuals:
##      Min       1Q   Median       3Q      Max
## -2.12949 -0.47944 -0.09555  0.49577  2.28261
##
## Random effects:
##  Groups   Name        Variance Std.Dev.
##  Litter   (Intercept) 0.4197   0.6478
##  Residual             0.4244   0.6514
## Number of obs: 81, groups:  Litter, 11
##
## Fixed effects:
##                             Estimate Std. Error       df t value Pr(>|t|)
## (Intercept)                  1.07861    0.35696 14.64599   3.022 0.008775 **
## Timepointd7                  0.89389    0.32747 64.48619   2.730 0.008163 **
## Timepointd21                 1.93256    0.29551 64.08135   6.540 1.18e-08 ***
## Timepointd35                 1.24252    0.31699 64.74225   3.920 0.000216 ***
## TreatmentPNS                -0.08811    0.48313 14.62823  -0.182 0.857787
## Timepointd7:TreatmentPNS     0.42033    0.42763 64.21283   0.983 0.329332
## Timepointd21:TreatmentPNS    0.34720    0.39241 63.91381   0.885 0.379586
## Timepointd35:TreatmentPNS   -0.25354    0.43379 64.72015  -0.584 0.560938
## ---
## Signif. codes:  0 '***' 0.001 '**' 0.01 '*' 0.05 '.' 0.1 ' ' 1
##
## Correlation of Fixed Effects:
##             (Intr) Tmpnt7 Tmpn21 Tmpn35 TrtPNS T7:TPN T21:TP
## Timepointd7 -0.366
## Timepontd21 -0.412  0.420
## Timepontd35 -0.385  0.449  0.445
## TreatmntPNS -0.739  0.270  0.305  0.285
## Tmpnt7:TPNS  0.280 -0.766 -0.321 -0.344 -0.383
## Tmpn21:TPNS  0.311 -0.316 -0.753 -0.335 -0.419  0.459
## Tmpn35:TPNS  0.281 -0.328 -0.325 -0.731 -0.378  0.441  0.452
```

```
# Create a type III ANOVA table
Tgfb1_anova_table <- anova(Tgfb1_model, ddf = "Kenward-Roger")
print(Tgfb1_anova_table)
```

```
## Type III Analysis of Variance Table with Kenward-Roger's method
##                     Sum Sq Mean Sq NumDF  DenDF F value    Pr(>F)
## Timepoint           48.878 16.2927     3 65.061 38.3935 2.109e-14 ***
## Treatment            0.004  0.0039     1  9.009  0.0092    0.9256
## Timepoint:Treatment  1.285  0.4283     3 65.061  1.0092    0.3944
## ---
## Signif. codes:  0 '***' 0.001 '**' 0.01 '*' 0.05 '.' 0.1 ' ' 1
```

```
#Because no interaction, test for main effects of treatment at individual time points
Tgfb1_emcatcat <- emmeans(Tgfb1_model, ~ Treatment | Timepoint)
#Is there a difference between treatment groups at each time point?
contrast(Tgfb1_emcatcat, "revpairwise", adjust="BH")
```

```
## Timepoint = d0:
##  contrast      estimate    SE   df t.ratio p.value
##  PNS - Control  -0.0881 0.483 15.5  -0.182  0.8577
##
## Timepoint = d7:
##  contrast      estimate    SE   df t.ratio p.value
##  PNS - Control   0.3322 0.508 18.5   0.653  0.5215
##
## Timepoint = d21:
##  contrast      estimate    SE   df t.ratio p.value
##  PNS - Control   0.2591 0.478 14.8   0.542  0.5959
##
## Timepoint = d35:
##  contrast      estimate    SE   df t.ratio p.value
##  PNS - Control  -0.3417 0.514 19.0  -0.665  0.5140
##
## Degrees-of-freedom method: kenward-roger
```

Lung Tissue *Foxp3* Expression

```
# Fit linear mixed-effects model
Foxp3_model <- lmer(Foxp3 ~ Timepoint * Treatment + (1 | Litter), data = lung_dat) # include random intercept for litter
summary(Foxp3_model)
```

```
## Linear mixed model fit by REML. t-tests use Satterthwaite's method [
## lmerModLmerTest]
## Formula: Foxp3 ~ Timepoint * Treatment + (1 | Litter)
##    Data: lung_dat
##
## REML criterion at convergence: 587.5
##
## Scaled residuals:
##     Min      1Q  Median      3Q     Max
## -2.2068 -0.4793  0.0122  0.4044  3.7759
##
## Random effects:
##  Groups   Name        Variance Std.Dev.
##  Litter   (Intercept)  23.31    4.828
##  Residual             144.74   12.031
## Number of obs: 80, groups:  Litter, 11
##
## Fixed effects:
##                              Estimate Std. Error       df t value Pr(>|t|)
## (Intercept)                    1.8347     4.3993  34.5188   0.417  0.67923
## Timepointd7                    9.0068     5.9914  65.6699   1.503  0.13756
## Timepointd21                  32.2236     5.4243  64.9052   5.941 1.23e-07 ***
## Timepointd35                  42.9711     5.7854  66.3666   7.427 2.72e-10 ***
## TreatmentPNS                  -0.4546     5.9536  34.5815  -0.076  0.93957
## Timepointd7:TreatmentPNS       0.9669     7.9304  65.2878   0.122  0.90333
## Timepointd21:TreatmentPNS     23.7358     7.2136  64.5025   3.290  0.00162 **
## Timepointd35:TreatmentPNS    -15.8601     7.9162  66.6866  -2.003  0.04919 *
## ---
## Signif. codes:  0 '***' 0.001 '**' 0.01 '*' 0.05 '.' 0.1 ' ' 1
##
## Correlation of Fixed Effects:
##             (Intr) Tmpnt7 Tmpn21 Tmpn35 TrtPNS T7:TPN T21:TP
## Timepointd7 -0.552
## Timepontd21 -0.616  0.436
## Timepontd35 -0.578  0.439  0.458
## TreatmntPNS -0.739  0.408  0.455  0.427
## Tmpnt7:TPNS  0.417 -0.755 -0.329 -0.332 -0.568
## Tmpn21:TPNS  0.463 -0.328 -0.752 -0.344 -0.626  0.462
## Tmpn35:TPNS  0.422 -0.321 -0.334 -0.731 -0.569  0.437  0.462
```

```
# Create a type III ANOVA table
Foxp3_anova_table <- anova(Foxp3_model, ddf = "Kenward-Roger")
print(Foxp3_anova_table)
```

```
## Type III Analysis of Variance Table with Kenward-Roger's method
##                      Sum Sq Mean Sq NumDF  DenDF F value    Pr(>F)
## Timepoint           26814.3  8938.1     3 66.185 61.7492 < 2.2e-16 ***
## Treatment              27.2    27.2     1  8.871  0.1881    0.6749
## Timepoint:Treatment  3807.1  1269.0     3 66.185  8.7671 5.629e-05 ***
## ---
## Signif. codes:  0 '***' 0.001 '**' 0.01 '*' 0.05 '.' 0.1 ' ' 1
```

```
# Because we have an interaction
Foxp3_emcatcat <- emmeans(Foxp3_model, ~ Timepoint * Treatment)
#Is there a difference between treatment groups at each time point?
contrast(Foxp3_emcatcat, "revpairwise", by="Timepoint", adjust="BH")
```

```
## Timepoint = d0:
##  contrast      estimate   SE   df t.ratio p.value
##  PNS - Control   -0.455 5.97 34.8  -0.076  0.9397
##
## Timepoint = d7:
##  contrast      estimate   SE   df t.ratio p.value
##  PNS - Control    0.512 6.72 44.4   0.076  0.9396
##
## Timepoint = d21:
##  contrast      estimate   SE   df t.ratio p.value
##  PNS - Control   23.281 5.82 32.3   4.002  0.0003
##
## Timepoint = d35:
##  contrast      estimate   SE   df t.ratio p.value
##  PNS - Control  -16.315 6.73 42.0  -2.425  0.0197
##
## Degrees-of-freedom method: kenward-roger
```

```
#Is there a difference between time points within each treatment?
contrast(Foxp3_emcatcat, "revpairwise", by="Treatment", adjust="BH")
```

```
## Treatment = Control:
##  contrast  estimate   SE   df t.ratio p.value
##  d7 - d0       9.01 6.02 65.8   1.496  0.1393
##  d21 - d0     32.22 5.44 65.0   5.919  <.0001
##  d21 - d7     23.22 6.15 68.8   3.774  0.0005
##  d35 - d0     42.97 5.82 66.4   7.383  <.0001
##  d35 - d7     33.96 6.25 63.6   5.437  <.0001
##  d35 - d21    10.75 5.91 68.6   1.820  0.0878
##
## Treatment = PNS:
##  contrast  estimate   SE   df t.ratio p.value
##  d7 - d0       9.97 5.22 64.9   1.912  0.0603
##  d21 - d0     55.96 4.77 64.1  11.743  <.0001
##  d21 - d7     45.99 5.02 64.4   9.155  <.0001
##  d35 - d0     27.11 5.45 67.1   4.977  <.0001
##  d35 - d7     17.14 5.68 67.0   3.016  0.0043
##  d35 - d21   -28.85 5.33 68.2  -5.416  <.0001
##
## Degrees-of-freedom method: kenward-roger
## P value adjustment: BH method for 6 tests
```

Lung Tissue *Il18* Expression

```
# Fit linear mixed-effects model
# Include random intercept for litter
IL18_model <- lmer(IL18 ~ Timepoint * Treatment + (1 | Litter), data = lung_dat)
summary(IL18_model)
```

```
## Linear mixed model fit by REML. t-tests use Satterthwaite's method [
## lmerModLmerTest]
## Formula: IL18 ~ Timepoint * Treatment + (1 | Litter)
##    Data: lung_dat
##
## REML criterion at convergence: 222.7
##
## Scaled residuals:
##      Min       1Q   Median       3Q      Max
## -2.19802 -0.52022  0.03859  0.46107  2.77717
##
## Random effects:
##  Groups   Name        Variance Std.Dev.
##  Litter   (Intercept) 0.2480   0.4980
##  Residual             0.8738   0.9348
## Number of obs: 80, groups:  Litter, 11
##
## Fixed effects:
##                          Estimate Std. Error       df t value Pr(>|t|)
## (Intercept)               1.06047    0.37232 26.03224   2.848  0.00847 **
## Timepointd7               0.54657    0.46711 64.65642   1.170  0.24625
## Timepointd21              2.48765    0.42244 63.91852   5.889 1.57e-07 ***
## Timepointd35              2.58117    0.45147 65.23348   5.717 2.92e-07 ***
## TreatmentPNS             -0.09329    0.50384 26.03875  -0.185  0.85454
## Timepointd7:TreatmentPNS   0.22379    0.61792 64.24914   0.362  0.71841
## Timepointd21:TreatmentPNS  0.17435    0.56150 63.56539   0.311  0.75719
## Timepointd35:TreatmentPNS  0.69959    0.61780 65.35564   1.132  0.26161
## ---
## Signif. codes:  0 '***' 0.001 '**' 0.01 '*' 0.05 '.' 0.1 ' ' 1
##
## Correlation of Fixed Effects:
##             (Intr) Tmpnt7 Tmpn21 Tmpn35 TrtPNS T7:TPN T21:TP
## Timepointd7 -0.505
## Timepontd21 -0.566  0.430
## Timepontd35 -0.530  0.442  0.453
## TreatmntPNS -0.739  0.373  0.418  0.392
## Tmpnt7:TPNS  0.382 -0.756 -0.325 -0.334 -0.522
## Tmpn21:TPNS  0.426 -0.323 -0.752 -0.341 -0.576  0.459
## Tmpn35:TPNS  0.387 -0.323 -0.331 -0.731 -0.522  0.433  0.458
```

```
# Create a type III ANOVA table
IL18_anova_table <- anova(IL18_model, ddf = "Kenward-Roger")
print(IL18_anova_table)
```

```
## Type III Analysis of Variance Table with Kenward-Roger's method
##                     Sum Sq Mean Sq NumDF  DenDF F value    Pr(>F)
## Timepoint          119.009  39.670     3 65.508 45.3953 5.443e-16 ***
## Treatment            0.207   0.207     1  8.949  0.2374    0.6378
## Timepoint:Treatment   1.158   0.386     3 65.508  0.4415    0.7241
## ---
## Signif. codes:  0 '***' 0.001 '**' 0.01 '*' 0.05 '.' 0.1 ' ' 1
```

```
#Because no interaction, test for main effects of treatment at individual time points
IL18_emcatcat <- emmeans(IL18_model, ~ Treatment | Timepoint)
#Is there a difference between treatment groups at each time point?
contrast(IL18_emcatcat, "revpairwise", adjust="BH")
```

```
## Timepoint = d0:
##  contrast      estimate    SE   df t.ratio p.value
##  PNS - Control  -0.0933 0.505 27.4  -0.185  0.8547
##
## Timepoint = d7:
##  contrast      estimate    SE   df t.ratio p.value
##  PNS - Control   0.1305 0.559 35.8   0.233  0.8169
##
## Timepoint = d21:
##  contrast      estimate    SE   df t.ratio p.value
##  PNS - Control   0.0811 0.494 25.5   0.164  0.8710
##
## Timepoint = d35:
##  contrast      estimate    SE   df t.ratio p.value
##  PNS - Control   0.6063 0.561 34.7   1.081  0.2871
##
## Degrees-of-freedom method: kenward-roger
```

Lung Tissue *Il4* Expression

```
# Fit linear mixed-effects model
# Include random intercept for litter
IL4_model <- lmer(IL4 ~ Timepoint * Treatment + (1 | Litter), data = lung_dat)
summary(IL4_model)
```

```
## Linear mixed model fit by REML. t-tests use Satterthwaite's method [
## lmerModLmerTest]
## Formula: IL4 ~ Timepoint * Treatment + (1 | Litter)
##    Data: lung_dat
##
## REML criterion at convergence: 133.6
##
## Scaled residuals:
##     Min      1Q  Median      3Q     Max
## -1.3635 -0.4349 -0.0496  0.1841  4.5637
##
## Random effects:
##  Groups   Name        Variance Std.Dev.
##  Litter   (Intercept) 0.1192   0.3452
##  Residual             0.2933   0.5416
## Number of obs: 73, groups:  Litter, 11
##
## Fixed effects:
##                          Estimate Std. Error       df t value Pr(>|t|)
## (Intercept)               0.96801    0.23828 21.56830   4.062 0.000535 ***
## Timepointd7              -0.79602    0.27814 57.17634  -2.862 0.005874 **
## Timepointd21             -0.43809    0.25692 55.35707  -1.705 0.093772 .
## Timepointd35             -0.83423    0.28020 57.87734  -2.977 0.004241 **
## TreatmentPNS              0.15390    0.32254 21.45778   0.477 0.638074
## Timepointd7:TreatmentPNS   0.44710    0.36787 56.84092   1.215 0.229255
## Timepointd21:TreatmentPNS -0.37665    0.34218 55.93227  -1.101 0.275719
## Timepointd35:TreatmentPNS -0.01544    0.38865 57.79030  -0.040 0.968445
## ---
## Signif. codes:  0 '***' 0.001 '**' 0.01 '*' 0.05 '.' 0.1 ' ' 1
##
## Correlation of Fixed Effects:
##             (Intr) Tmpnt7 Tmpn21 Tmpn35 TrtPNS T7:TPN T21:TP
## Timepointd7 -0.495
## Timepontd21 -0.539  0.454
## Timepontd35 -0.500  0.454  0.459
## TreatmntPNS -0.739  0.366  0.398  0.369
## Tmpnt7:TPNS  0.374 -0.756 -0.344 -0.343 -0.511
## Tmpn21:TPNS  0.405 -0.341 -0.751 -0.345 -0.549  0.484
## Tmpn35:TPNS  0.360 -0.327 -0.331 -0.721 -0.478  0.435  0.451
```

```
# Create a type III ANOVA table
IL4_anova_table <- anova(IL4_model, ddf = "Kenward-Roger")
print(IL4_anova_table)
```

```
## Type III Analysis of Variance Table with Kenward-Roger's method
##                      Sum Sq Mean Sq NumDF  DenDF F value    Pr(>F)    
## Timepoint           6.6791 2.22638     3 58.013  7.5902 0.0002294 ***
## Treatment           0.1334 0.13342     1  8.954  0.4549 0.5170615    
## Timepoint:Treatment 1.5152 0.50508     3 58.013  1.7219 0.1724867    
## ---
## Signif. codes:  0 '***' 0.001 '**' 0.01 '*' 0.05 '.' 0.1 ' ' 1
```

```
#Because no interaction, test for main effects of treatment at individual time points
IL4_emcatcat <- emmeans(IL4_model, ~ Treatment | Timepoint)
#Is there a difference between treatment groups at each time point?
contrast(IL4_emcatcat, "revpairwise", adjust="BH")
```

```
## Timepoint = d0:
##  contrast      estimate    SE   df t.ratio p.value
##  PNS - Control    0.154 0.323 23.8   0.477  0.6379
## 
## Timepoint = d7:
##  contrast      estimate    SE   df t.ratio p.value
##  PNS - Control    0.601 0.345 28.3   1.743  0.0923
## 
## Timepoint = d21:
##  contrast      estimate    SE   df t.ratio p.value
##  PNS - Control   -0.223 0.316 22.2  -0.704  0.4886
## 
## Timepoint = d35:
##  contrast      estimate    SE   df t.ratio p.value
##  PNS - Control    0.138 0.370 32.9   0.374  0.7107
## 
## Degrees-of-freedom method: kenward-roger
```

Lung Tissue *Il5* Expression

```
# Fit linear mixed-effects model
# Include random intercept for litter
IL5_model <- lmer(IL5 ~ Timepoint * Treatment + (1 | Litter), data = lung_dat)
summary(IL5_model)
```

```
## Linear mixed model fit by REML. t-tests use Satterthwaite's method [
## lmerModLmerTest]
## Formula: IL5 ~ Timepoint * Treatment + (1 | Litter)
##    Data: lung_dat
## 
## REML criterion at convergence: 270.5
## 
## Scaled residuals: 
##     Min      1Q  Median      3Q     Max 
## -1.7876 -0.5639 -0.0136  0.2796  3.3628 
## 
## Random effects:
##  Groups   Name        Variance Std.Dev.
##  Litter   (Intercept) 1.664    1.290   
##  Residual             1.761    1.327   
## Number of obs: 77, groups:  Litter, 11
## 
## Fixed effects:
##                             Estimate Std. Error      df t value Pr(>|t|)   
## (Intercept)                   1.0164     0.7163 14.5030   1.419  0.17708   
## Timepointd7                   2.1922     0.6682 60.6448   3.281  0.00172 **
## Timepointd21                  1.0750     0.5971 59.6252   1.800  0.07685 . 
## Timepointd35                  0.1622     0.6472 60.9538   0.251  0.80299   
## TreatmentPNS                  0.3227     0.9776 14.9323   0.330  0.74593   
## Timepointd7:TreatmentPNS      2.1880     0.8926 60.4719   2.451  0.01715 * 
## Timepointd21:TreatmentPNS     0.7058     0.8157 59.9988   0.865  0.39038   
## Timepointd35:TreatmentPNS     0.4980     0.9073 60.9388   0.549  0.58512   
## ---
## Signif. codes:  0 '***' 0.001 '**' 0.01 '*' 0.05 '.' 0.1 ' ' 1
## 
## Correlation of Fixed Effects:
##             (Intr) Tmpnt7 Tmpn21 Tmpn35 TrtPNS T7:TPN T21:TP
## Timepointd7 -0.371                                          
## Timepontd21 -0.422  0.445                                   
## Timepontd35 -0.390  0.451  0.468                            
## TreatmntPNS -0.733  0.272  0.309  0.286                     
## Tmpnt7:TPNS  0.278 -0.749 -0.333 -0.338 -0.396              
## Tmpn21:TPNS  0.309 -0.326 -0.732 -0.343 -0.436  0.481       
## Tmpn35:TPNS  0.278 -0.322 -0.334 -0.713 -0.384  0.442  0.467
```

```
# Create a type III ANOVA table
IL5_anova_table <- anova(IL5_model, ddf = "Kenward-Roger")
print(IL5_anova_table)
```

```
## Type III Analysis of Variance Table with Kenward-Roger's method
##                      Sum Sq Mean Sq NumDF  DenDF F value    Pr(>F)    
## Timepoint           108.467  36.156     3 60.998 20.5323 2.577e-09 ***
## Treatment             3.378   3.378     1  8.989  1.9182    0.1995    
## Timepoint:Treatment  11.222   3.741     3 60.998  2.1242    0.1064    
## ---
## Signif. codes:  0 '***' 0.001 '**' 0.01 '*' 0.05 '.' 0.1 ' ' 1
```

```
#Because no interaction, test for main effects of treatment at individual time points
IL5_emcatcat <- emmeans(IL5_model, ~ Treatment | Timepoint)
#Is there a difference between treatment groups at each time point?
contrast(IL5_emcatcat, "revpairwise", adjust="BH")
```

```
## Timepoint = d0:
##  contrast      estimate    SE   df t.ratio p.value
##  PNS - Control    0.323 0.978 15.9   0.330  0.7458
##
## Timepoint = d7:
##  contrast      estimate    SE   df t.ratio p.value
##  PNS - Control    2.511 1.030 19.1   2.434  0.0249
##
## Timepoint = d21:
##  contrast      estimate    SE   df t.ratio p.value
##  PNS - Control    1.028 0.963 15.0   1.068  0.3023
##
## Timepoint = d35:
##  contrast      estimate    SE   df t.ratio p.value
##  PNS - Control    0.821 1.050 20.0   0.782  0.4436
##
## Degrees-of-freedom method: kenward-roger
```

Lung Tissue *Il13* Expression

```
# Fit linear mixed-effects model
# Include random intercept for litter
IL13_model <- lmer(IL13 ~ Timepoint * Treatment + (1 | Litter), data = lung_dat)
```

```
## boundary (singular) fit: see help('isSingular')
```

```
summary(IL13_model)
```

```
## Linear mixed model fit by REML. t-tests use Satterthwaite's method [
## lmerModLmerTest]
## Formula: IL13 ~ Timepoint * Treatment + (1 | Litter)
##    Data: lung_dat
##
## REML criterion at convergence: 496
##
## Scaled residuals:
##     Min      1Q  Median      3Q     Max
## -2.9710 -0.1468 -0.0260  0.0929  4.7550
##
## Random effects:
##  Groups   Name        Variance Std.Dev.
##  Litter   (Intercept)  0.00    0.000
##  Residual             49.02    7.001
## Number of obs: 79, groups:  Litter, 11
##
## Fixed effects:
##                             Estimate Std. Error      df t value Pr(>|t|)
## (Intercept)                   1.1343     2.2140 71.0000   0.512   0.6100
## Timepointd7                  18.6871     3.4503 71.0000   5.416 7.88e-07 ***
## Timepointd21                  1.4200     3.1311 71.0000   0.454   0.6516
## Timepointd35                  0.2851     3.3211 71.0000   0.086   0.9318
## TreatmentPNS                 -0.2126     3.0591 71.0000  -0.069   0.9448
## Timepointd7:TreatmentPNS     10.9213     4.5626 71.0000   2.394   0.0193 *
## Timepointd21:TreatmentPNS     1.5608     4.2463 71.0000   0.368   0.7143
## Timepointd35:TreatmentPNS     0.3061     4.5752 71.0000   0.067   0.9468
## ---
## Signif. codes:  0 '***' 0.001 '**' 0.01 '*' 0.05 '.' 0.1 ' ' 1
##
## Correlation of Fixed Effects:
##             (Intr) Tmpnt7 Tmpn21 Tmpn35 TrtPNS T7:TPN T21:TP
## Timepointd7 -0.642
## Timepontd21 -0.707  0.454
## Timepontd35 -0.667  0.428  0.471
## TreatmntPNS -0.724  0.464  0.512  0.482
## Tmpnt7:TPNS  0.485 -0.756 -0.343 -0.324 -0.670
## Tmpn21:TPNS  0.521 -0.335 -0.737 -0.348 -0.720  0.483
## Tmpn35:TPNS  0.484 -0.311 -0.342 -0.726 -0.669  0.448  0.482
## optimizer (nloptwrap) convergence code: 0 (OK)
## boundary (singular) fit: see help('isSingular')
```

```
# Create a type III ANOVA table
IL13_anova_table <- anova(IL13_model, ddf = "Kenward-Roger")
print(IL13_anova_table)
```

```
## Type III Analysis of Variance Table with Kenward-Roger's method
##                     Sum Sq Mean Sq NumDF  DenDF F value  Pr(>F)
## Timepoint           7162.8 2387.61     3 66.360 48.7038 < 2e-16 ***
## Treatment            166.5  166.45     1  8.223  3.3957 0.10161
## Timepoint:Treatment  354.1  118.04     3 66.360  2.4079 0.07489 .
## ---
## Signif. codes:  0 '***' 0.001 '**' 0.01 '*' 0.05 '.' 0.1 ' ' 1
```

```r
#Because no interaction, test for main effects of treatment at individual time points
IL13_emcatcat <- emmeans(IL13_model, ~ Treatment | Timepoint)
#Is there a difference between treatment groups at each time point?
contrast(IL13_emcatcat, "revpairwise", adjust="BH")
```

```
## Timepoint = d0:
##  contrast      estimate   SE   df t.ratio p.value
##  PNS - Control  -0.2126 3.07 56.9  -0.069  0.9451
##
## Timepoint = d7:
##  contrast      estimate   SE   df t.ratio p.value
##  PNS - Control  10.7088 3.41 60.3   3.139  0.0026
##
## Timepoint = d21:
##  contrast      estimate   SE   df t.ratio p.value
##  PNS - Control   1.3482 2.95 54.8   0.456  0.6499
##
## Timepoint = d35:
##  contrast      estimate   SE   df t.ratio p.value
##  PNS - Control   0.0935 3.46 55.0   0.027  0.9785
##
## Degrees-of-freedom method: kenward-roger
```

Lung Tissue *Il17a* Expression

```r
# Fit linear mixed-effects model
# Include random intercept for litter
IL17_model <- lmer(IL17 ~ Timepoint * Treatment + (1 | Litter), data = lung_dat)
summary(IL17_model)
```

```
## Linear mixed model fit by REML. t-tests use Satterthwaite's method [
## lmerModLmerTest]
## Formula: IL17 ~ Timepoint * Treatment + (1 | Litter)
##    Data: lung_dat
##
## REML criterion at convergence: 510.6
##
## Scaled residuals:
##     Min      1Q  Median      3Q     Max
## -1.7490 -0.5178 -0.2113  0.3785  4.4212
##
## Random effects:
##  Groups   Name        Variance Std.Dev.
##  Litter   (Intercept)  49.31    7.022
##  Residual             108.05   10.395
## Number of obs: 72, groups:  Litter, 11
##
## Fixed effects:
##                             Estimate Std. Error        df t value Pr(>|t|)
## (Intercept)                  2.40968    4.57324  19.56949   0.527 0.604179
## Timepointd7                 19.59865    5.21957  56.51296   3.755 0.000412 ***
## Timepointd21                14.71747    5.03079  56.24613   2.925 0.004952 **
## Timepointd35                 0.09085    5.98982  59.01998   0.015 0.987950
## TreatmentPNS                -0.06609    6.33255  21.19265  -0.010 0.991770
## Timepointd7:TreatmentPNS  -10.26573    6.94289  55.91269  -1.479 0.144860
## Timepointd21:TreatmentPNS   2.47798    6.68939  55.77760   0.370 0.712462
## Timepointd35:TreatmentPNS   0.19289    7.86829  58.26458   0.025 0.980526
## ---
## Signif. codes:  0 '***' 0.001 '**' 0.01 '*' 0.05 '.' 0.1 ' ' 1
##
## Correlation of Fixed Effects:
##             (Intr) Tmpnt7 Tmpn21 Tmpn35 TrtPNS T7:TPN T21:TP
## Timepointd7 -0.456
## Timepontd21 -0.481  0.399
## Timepontd35 -0.414  0.379  0.349
## TreatmntPNS -0.722  0.329  0.347  0.299
## Tmpnt7:TPNS  0.343 -0.752 -0.300 -0.285 -0.498
## Tmpn21:TPNS  0.362 -0.300 -0.752 -0.262 -0.519  0.465
## Tmpn35:TPNS  0.315 -0.288 -0.266 -0.761 -0.452  0.420  0.408
```

```r
# Create a type III ANOVA table
IL17_anova_table <- anova(IL17_model, ddf = "Kenward-Roger")
print(IL17_anova_table)
```

```
## Type III Analysis of Variance Table with Kenward-Roger's method
##                     Sum Sq Mean Sq NumDF  DenDF F value    Pr(>F)
## Timepoint           3820.1 1273.35     3 57.529 11.7845 4.025e-06 ***
## Treatment             16.8   16.77     1  9.062  0.1553    0.7027
## Timepoint:Treatment  405.0  135.01     3 57.529  1.2495    0.3003
## ---
## Signif. codes:  0 '***' 0.001 '**' 0.01 '*' 0.05 '.' 0.1 ' ' 1
```

```r
#Because no interaction, test for main effects of treatment at individual time points
IL17_emcatcat <- emmeans(IL17_model, ~ Treatment | Timepoint)
#Is there a difference between treatment groups at each time point?
contrast(IL17_emcatcat, "revpairwise", adjust="BH")
```

```
## Timepoint = d0:
##  contrast      estimate   SE   df t.ratio p.value
##  PNS - Control  -0.0661 6.34 22.2  -0.010  0.9918
##
## Timepoint = d7:
##  contrast      estimate   SE   df t.ratio p.value
##  PNS - Control -10.3318 6.69 25.7  -1.544  0.1348
##
## Timepoint = d21:
##  contrast      estimate   SE   df t.ratio p.value
##  PNS - Control   2.4119 6.41 22.5   0.377  0.7100
##
## Timepoint = d35:
##  contrast      estimate   SE   df t.ratio p.value
##  PNS - Control   0.1268 7.60 34.6   0.017  0.9868
##
## Degrees-of-freedom method: kenward-roger
```

Sex Differences in Lung Tissue Gene Expression

Lung Tissue *Ifng* Expression Sex Differences

```
# Does lung Ifny expression differ by sex
#Include random intercept for litter
Ifng_sex_effects <- lmer(Ifng ~ Timepoint * Treatment * Sex + (1 | Litter), data = lung_dat)
Ifng_anova_table_for_sex <- anova(Ifng_sex_effects, ddf = "Kenward-Roger")
print(Ifng_anova_table_for_sex)
```

```
## Type III Analysis of Variance Table with Kenward-Roger's method
##                         Sum Sq Mean Sq NumDF  DenDF F value   Pr(>F)
## Timepoint              239.318 119.659     2 40.932  5.6738 0.006685 **
## Treatment               71.274  71.274     1 10.851  3.3798 0.093503 .
## Sex                      0.377   0.377     1 40.676  0.0179 0.894294
## Timepoint:Treatment     28.766  14.383     2 40.932  0.6820 0.511261
## Timepoint:Sex           21.367  10.684     2 42.576  0.5061 0.606421
## Treatment:Sex            0.083   0.083     1 40.676  0.0039 0.950203
## Timepoint:Treatment:Sex  24.580  12.290     2 42.576  0.5822 0.563049
## ---
## Signif. codes:  0 '***' 0.001 '**' 0.01 '*' 0.05 '.' 0.1 ' ' 1
```

```
Ifng_emcatcat_sex <- emmeans(Ifng_sex_effects, ~ Timepoint * Treatment * Sex)
#Is there a difference in males or females between treatment group at each time point?
contrast(Ifng_emcatcat_sex, "revpairwise", by=c("Sex", "Timepoint"), adjust="BH")
```

```
## Sex = F, Timepoint = d7:
##  contrast      estimate   SE   df t.ratio p.value
##  PNS - Control    3.703 3.54 40.7   1.045  0.3020
##
## Sex = M, Timepoint = d7:
##  contrast      estimate   SE   df t.ratio p.value
##  PNS - Control    6.922 5.31 44.0   1.303  0.1994
##
## Sex = F, Timepoint = d21:
##  contrast      estimate   SE   df t.ratio p.value
##  PNS - Control    6.530 3.05 31.7   2.141  0.0400
##
## Sex = M, Timepoint = d21:
##  contrast      estimate   SE   df t.ratio p.value
##  PNS - Control    1.809 3.53 34.5   0.513  0.6112
##
## Sex = F, Timepoint = d35:
##  contrast      estimate   SE   df t.ratio p.value
##  PNS - Control    0.369 4.01 42.5   0.092  0.9271
##
## Sex = M, Timepoint = d35:
##  contrast      estimate   SE   df t.ratio p.value
##  PNS - Control    1.284 4.16 43.2   0.308  0.7593
##
## Degrees-of-freedom method: kenward-roger
```

```
#Is there a difference in males or females within treatment group at each time point?
contrast(Ifng_emcatcat_sex, "revpairwise", by=c("Treatment", "Timepoint"), adjust="BH")
```

```
## Treatment = Control, Timepoint = d7:
##  contrast estimate   SE   df t.ratio p.value
##  M - F      -3.693 5.28 41.9  -0.699  0.4884
##
## Treatment = PNS, Timepoint = d7:
##  contrast estimate   SE   df t.ratio p.value
##  M - F      -0.475 3.37 41.9  -0.141  0.8886
##
## Treatment = Control, Timepoint = d21:
##  contrast estimate   SE   df t.ratio p.value
##  M - F       2.909 3.68 38.3   0.791  0.4336
##
## Treatment = PNS, Timepoint = d21:
##  contrast estimate   SE   df t.ratio p.value
##  M - F      -1.813 2.84 43.3  -0.639  0.5263
##
## Treatment = Control, Timepoint = d35:
##  contrast estimate   SE   df t.ratio p.value
##  M - F       1.704 4.01 40.0   0.425  0.6731
##
## Treatment = PNS, Timepoint = d35:
##  contrast estimate   SE   df t.ratio p.value
##  M - F       2.619 3.84 39.6   0.683  0.4987
##
## Degrees-of-freedom method: kenward-roger
```

Lung Tissue *Ifnb* Expression Sex Differences

```
# Does lung Ifnb expression differ by sex
#Include random intercept for litter
Ifnb_sex_effects <- lmer(Ifnb ~ Timepoint * Treatment * Sex + (1 | Litter), data = lung_dat)
Ifnb_anova_table_for_sex <- anova(Ifnb_sex_effects, ddf = "Kenward-Roger")
print(Ifnb_anova_table_for_sex)
```

```
## Type III Analysis of Variance Table with Kenward-Roger's method
##                         Sum Sq Mean Sq NumDF  DenDF F value  Pr(>F)
## Timepoint                65.32   32.66     2 43.328  0.3528 0.70474
## Treatment               341.94  341.94     1 11.075  3.6932 0.08073 .
## Sex                       0.01    0.01     1 44.466  0.0001 0.99179
## Timepoint:Treatment     604.16  302.08     2 43.328  3.2626 0.04788 *
## Timepoint:Sex           346.64  173.32     2 45.514  1.8706 0.16569
## Treatment:Sex             3.05    3.05     1 44.466  0.0330 0.85674
## Timepoint:Treatment:Sex  56.59   28.30     2 45.514  0.3054 0.73833
## ---
## Signif. codes:  0 '***' 0.001 '**' 0.01 '*' 0.05 '.' 0.1 ' ' 1
```

```
Ifnb_emcatcat_sex <- emmeans(Ifnb_sex_effects, ~ Timepoint * Treatment * Sex)
#Is there a difference in males or females between treatment group at each time point?
contrast(Ifnb_emcatcat_sex, "revpairwise", by=c("Sex", "Timepoint"), adjust="BH")
```

```
## Sex = F, Timepoint = d7:
##  contrast      estimate    SE   df t.ratio p.value
##  PNS - Control   21.013  6.94 39.5   3.026  0.0043
##
## Sex = M, Timepoint = d7:
##  contrast      estimate    SE   df t.ratio p.value
##  PNS - Control   17.668 11.10 47.0   1.586  0.1194
##
## Sex = F, Timepoint = d21:
##  contrast      estimate    SE   df t.ratio p.value
##  PNS - Control    2.697  6.43 33.6   0.420  0.6775
##
## Sex = M, Timepoint = d21:
##  contrast      estimate    SE   df t.ratio p.value
##  PNS - Control    8.018  7.28 37.2   1.101  0.2778
##
## Sex = F, Timepoint = d35:
##  contrast      estimate    SE   df t.ratio p.value
##  PNS - Control    0.767  8.43 45.2   0.091  0.9279
##
## Sex = M, Timepoint = d35:
##  contrast      estimate    SE   df t.ratio p.value
##  PNS - Control   -4.744  8.56 45.5  -0.554  0.5822
##
## Degrees-of-freedom method: kenward-roger
```

```
#Is there a difference in males or females within treatment group at each time point?
contrast(Ifnb_emcatcat_sex, "revpairwise", by=c("Treatment", "Timepoint"), adjust="BH")
```

```
## Treatment = Control, Timepoint = d7:
##  contrast estimate    SE   df t.ratio p.value
##  M - F       -7.01 11.10 44.8  -0.634  0.5294
##
## Treatment = PNS, Timepoint = d7:
##  contrast estimate    SE   df t.ratio p.value
##  M - F      -10.35  6.56 46.0  -1.578  0.1215
##
## Treatment = Control, Timepoint = d21:
##  contrast estimate    SE   df t.ratio p.value
##  M - F       -2.01  7.70 42.4  -0.261  0.7955
##
## Treatment = PNS, Timepoint = d21:
##  contrast estimate    SE   df t.ratio p.value
##  M - F        3.31  5.74 45.2   0.578  0.5664
##
## Treatment = Control, Timepoint = d35:
##  contrast estimate    SE   df t.ratio p.value
##  M - F       10.88  8.27 43.9   1.315  0.1952
##
## Treatment = PNS, Timepoint = d35:
##  contrast estimate    SE   df t.ratio p.value
##  M - F        5.37  8.02 42.5   0.670  0.5068
##
## Degrees-of-freedom method: kenward-roger
```

Lung Tissue *Isg15* Expression Sex Differences

```
# Does lung Isg15 expression differ by sex
#Include random intercept for litter
Isg15_sex_effects <- lmer(Isg15 ~ Timepoint * Treatment * Sex + (1 | Litter), data = lung_dat)
Isg15_anova_table_for_sex <- anova(Isg15_sex_effects, ddf = "Kenward-Roger")
print(Isg15_anova_table_for_sex)
```

```
## Type III Analysis of Variance Table with Kenward-Roger's method
##                         Sum Sq Mean Sq NumDF  DenDF F value    Pr(>F)
## Timepoint              25.1491 12.5746     2 40.913 61.3410 4.865e-13 ***
## Treatment               0.0809  0.0809     1 10.240  0.3948   0.54354
## Sex                     0.0002  0.0002     1 41.675  0.0008   0.97784
## Timepoint:Treatment     1.0070  0.5035     2 40.913  2.4560   0.09833 .
## Timepoint:Sex           0.2373  0.1186     2 43.348  0.5785   0.56499
## Treatment:Sex           0.6334  0.6334     1 41.675  3.0901   0.08611 .
## Timepoint:Treatment:Sex  0.3292  0.1646     2 43.348  0.8026   0.45469
## ---
## Signif. codes:  0 '***' 0.001 '**' 0.01 '*' 0.05 '.' 0.1 ' ' 1
```

```
Isg15_emcatcat_sex <- emmeans(Isg15_sex_effects, ~ Timepoint * Treatment * Sex)
#Is there a difference in males or females between treatment group at each time point?
contrast(Isg15_emcatcat_sex, "revpairwise", by=c("Sex", "Timepoint"), adjust="BH")
```

```
## Sex = F, Timepoint = d7:
##  contrast      estimate    SE   df t.ratio p.value
##  PNS - Control    0.385 0.374 31.3   1.029  0.3112
##
## Sex = M, Timepoint = d7:
##  contrast      estimate    SE   df t.ratio p.value
##  PNS - Control    0.128 0.558 46.4   0.229  0.8201
##
## Sex = F, Timepoint = d21:
##  contrast      estimate    SE   df t.ratio p.value
##  PNS - Control    1.038 0.357 26.3   2.908  0.0073
##
## Sex = M, Timepoint = d21:
##  contrast      estimate    SE   df t.ratio p.value
##  PNS - Control   -0.043 0.395 31.4  -0.109  0.9140
##
## Sex = F, Timepoint = d35:
##  contrast      estimate    SE   df t.ratio p.value
##  PNS - Control   -0.125 0.438 40.1  -0.285  0.7774
##
## Sex = M, Timepoint = d35:
##  contrast      estimate    SE   df t.ratio p.value
##  PNS - Control   -0.418 0.444 40.8  -0.943  0.3515
##
## Degrees-of-freedom method: kenward-roger
```

```
#Is there a difference in males or females within treatment group at each time point?
contrast(Isg15_emcatcat_sex, "revpairwise", by=c("Treatment", "Timepoint"), adjust="BH")
```

```
## Treatment = Control, Timepoint = d7:
##  contrast estimate    SE   df t.ratio p.value
##  M - F     -0.1079 0.527 41.7  -0.205  0.8387
##
## Treatment = PNS, Timepoint = d7:
##  contrast estimate    SE   df t.ratio p.value
##  M - F     -0.3659 0.313 42.3  -1.170  0.2486
##
## Treatment = Control, Timepoint = d21:
##  contrast estimate    SE   df t.ratio p.value
##  M - F      0.5561 0.394 46.9   1.411  0.1647
##
## Treatment = PNS, Timepoint = d21:
##  contrast estimate    SE   df t.ratio p.value
##  M - F     -0.5249 0.274 41.9  -1.917  0.0620
##
## Treatment = Control, Timepoint = d35:
##  contrast estimate    SE   df t.ratio p.value
##  M - F      0.3551 0.393 41.0   0.903  0.3718
##
## Treatment = PNS, Timepoint = d35:
##  contrast estimate    SE   df t.ratio p.value
##  M - F      0.0616 0.379 40.0   0.162  0.8718
##
## Degrees-of-freedom method: kenward-roger
```

Lung Tissue *Il1b* Expression Sex Differences

```
# Does lung Il1b expression differ by sex
#Include random intercept for litter
IL1b_sex_effects <- lmer(IL1b ~ Timepoint * Treatment * Sex + (1 | Litter), data = lung_dat)
```

```
## boundary (singular) fit: see help('isSingular')
```

```
IL1b_anova_table_for_sex <- anova(IL1b_sex_effects, ddf = "Kenward-Roger")
print(IL1b_anova_table_for_sex)
```

```
## Type III Analysis of Variance Table with Kenward-Roger's method
##                          Sum Sq Mean Sq NumDF  DenDF F value    Pr(>F)
## Timepoint               11.0973  5.5486     2 43.537 20.6194 5.011e-07 ***
## Treatment                2.9506  2.9506     1 10.737 10.9656  0.007157 **
## Sex                      0.4986  0.4986     1 44.862  1.8530  0.180233
## Timepoint:Treatment      0.9404  0.4702     2 43.537  1.7474  0.186232
## Timepoint:Sex            1.7735  0.8868     2 43.236  3.2903  0.046761 *
## Treatment:Sex            0.9600  0.9600     1 44.862  3.5678  0.065387 .
## Timepoint:Treatment:Sex  3.1291  1.5645     2 43.236  5.8051  0.005846 **
## ---
## Signif. codes:  0 '***' 0.001 '**' 0.01 '*' 0.05 '.' 0.1 ' ' 1
```

```
IL1b_emcatcat_sex <- emmeans(IL1b_sex_effects, ~ Timepoint * Treatment * Sex)
#Is there a difference in males or females between treatment group at each time point?
contrast(IL1b_emcatcat_sex, "revpairwise", by=c("Sex", "Timepoint"), adjust="BH")
```

```
## Sex = F, Timepoint = d7:
##  contrast      estimate    SE   df t.ratio p.value
##  PNS - Control    0.365 0.360 40.6   1.012  0.3173
##
## Sex = M, Timepoint = d7:
##  contrast      estimate    SE   df t.ratio p.value
##  PNS - Control    0.692 0.585 44.5   1.182  0.2433
##
## Sex = F, Timepoint = d21:
##  contrast      estimate    SE   df t.ratio p.value
##  PNS - Control    2.034 0.312 35.0   6.510  <.0001
##
## Sex = M, Timepoint = d21:
##  contrast      estimate    SE   df t.ratio p.value
##  PNS - Control   -0.151 0.358 36.7  -0.422  0.6754
##
## Sex = F, Timepoint = d35:
##  contrast      estimate    SE   df t.ratio p.value
##  PNS - Control    0.288 0.429 43.6   0.672  0.5052
##
## Sex = M, Timepoint = d35:
##  contrast      estimate    SE   df t.ratio p.value
##  PNS - Control    0.174 0.438 43.8   0.397  0.6932
##
## Degrees-of-freedom method: kenward-roger
```

```
#Is there a difference in males or females within treatment group at each time point?
contrast(IL1b_emcatcat_sex, "revpairwise", by=c("Treatment", "Timepoint"), adjust="BH")
```

```
## Treatment = Control, Timepoint = d7:
##  contrast estimate    SE   df t.ratio p.value
##  M - F     -0.1374 0.592 44.8  -0.232  0.8176
##
## Treatment = PNS, Timepoint = d7:
##  contrast estimate    SE   df t.ratio p.value
##  M - F      0.1893 0.358 44.6   0.529  0.5994
##
## Treatment = Control, Timepoint = d21:
##  contrast estimate    SE   df t.ratio p.value
##  M - F      0.2864 0.379 34.0   0.755  0.4554
##
## Treatment = PNS, Timepoint = d21:
##  contrast estimate    SE   df t.ratio p.value
##  M - F     -1.8983 0.302 45.0  -6.291  <.0001
##
## Treatment = Control, Timepoint = d35:
##  contrast estimate    SE   df t.ratio p.value
##  M - F      0.1265 0.439 44.5   0.288  0.7747
##
## Treatment = PNS, Timepoint = d35:
##  contrast estimate    SE   df t.ratio p.value
##  M - F      0.0121 0.430 43.5   0.028  0.9777
##
## Degrees-of-freedom method: kenward-roger
```

Lung Tissue *Il6* Expression Sex Differences

```
# Does lung IL6 expression differ by sex
#Include random intercept for litter
IL6_sex_effects <- lmer(IL6 ~ Timepoint * Treatment * Sex + (1 | Litter), data = lung_dat)
IL6_anova_table_for_sex <- anova(IL6_sex_effects, ddf = "Kenward-Roger")
print(IL6_anova_table_for_sex)
```

```
## Type III Analysis of Variance Table with Kenward-Roger's method
##                         Sum Sq Mean Sq NumDF  DenDF F value  Pr(>F)
## Timepoint               6.3444  3.1722     2 39.677  2.0764 0.13879
## Treatment               1.2423  1.2423     1 10.332  0.8132 0.38772
## Sex                     0.0007  0.0007     1 39.855  0.0005 0.98285
## Timepoint:Treatment    10.2223  5.1112     2 39.677  3.3456 0.04543 *
## Timepoint:Sex           2.4885  1.2443     2 41.906  0.8141 0.44993
## Treatment:Sex           0.0159  0.0159     1 39.855  0.0104 0.91928
## Timepoint:Treatment:Sex  2.5091  1.2546     2 41.906  0.8208 0.44702
## ---
## Signif. codes:  0 '***' 0.001 '**' 0.01 '*' 0.05 '.' 0.1 ' ' 1
```

```
IL6_emcatcat_sex <- emmeans(IL6_sex_effects, ~ Timepoint * Treatment * Sex)
#Is there a difference in males or females between treatment group at each time point?
contrast(IL6_emcatcat_sex, "revpairwise", by=c("Sex", "Timepoint"), adjust="BH")
```

```
## Sex = F, Timepoint = d7:
##  contrast      estimate    SE   df t.ratio p.value
##  PNS - Control    1.023 1.050 36.3   0.978  0.3347
##
## Sex = M, Timepoint = d7:
##  contrast      estimate    SE   df t.ratio p.value
##  PNS - Control    2.665 1.500 44.9   1.781  0.0818
##
## Sex = F, Timepoint = d21:
##  contrast      estimate    SE   df t.ratio p.value
##  PNS - Control    1.213 0.933 27.5   1.300  0.2044
##
## Sex = M, Timepoint = d21:
##  contrast      estimate    SE   df t.ratio p.value
##  PNS - Control    0.497 1.060 32.8   0.470  0.6414
##
## Sex = F, Timepoint = d35:
##  contrast      estimate    SE   df t.ratio p.value
##  PNS - Control   -0.357 1.160 40.5  -0.307  0.7607
##
## Sex = M, Timepoint = d35:
##  contrast      estimate    SE   df t.ratio p.value
##  PNS - Control   -1.542 1.180 41.0  -1.305  0.1991
##
## Degrees-of-freedom method: kenward-roger
```

```
#Is there a difference in males or females within treatment group at each time point?
contrast(IL6_emcatcat_sex, "revpairwise", by=c("Treatment", "Timepoint"), adjust="BH")
```

```
## Treatment = Control, Timepoint = d7:
##  contrast estimate    SE   df t.ratio p.value
##  M - F      -1.407 1.440 40.2  -0.980  0.3331
##
## Treatment = PNS, Timepoint = d7:
##  contrast estimate    SE   df t.ratio p.value
##  M - F       0.235 0.918 40.4   0.256  0.7994
##
## Treatment = Control, Timepoint = d21:
##  contrast estimate    SE   df t.ratio p.value
##  M - F       0.163 1.060 44.6   0.154  0.8786
##
## Treatment = PNS, Timepoint = d21:
##  contrast estimate    SE   df t.ratio p.value
##  M - F      -0.554 0.778 41.7  -0.712  0.4804
##
## Treatment = Control, Timepoint = d35:
##  contrast estimate    SE   df t.ratio p.value
##  M - F       1.346 1.070 39.5   1.256  0.2165
##
## Treatment = PNS, Timepoint = d35:
##  contrast estimate    SE   df t.ratio p.value
##  M - F       0.161 1.040 38.5   0.156  0.8771
##
## Degrees-of-freedom method: kenward-roger
```

Lung Tissue *Tnfa* Expression Sex Differences

```
# Does lung Tnf expression differ by sex
#Include random intercept for litter
Tnf_sex_effects <- lmer(Tnf ~ Timepoint * Treatment * Sex + (1 | Litter), data = lung_dat)
Tnf_anova_table_for_sex <- anova(Tnf_sex_effects, ddf = "Kenward-Roger")
print(Tnf_anova_table_for_sex)
```

```
## Type III Analysis of Variance Table with Kenward-Roger's method
##                         Sum Sq Mean Sq NumDF  DenDF F value    Pr(>F)
## Timepoint              25.8706 12.9353     2 42.860 21.4311 3.537e-07 ***
## Treatment               2.6911  2.6911     1 10.972  4.4587   0.05848 .
## Sex                     0.4441  0.4441     1 43.519  0.7358   0.39572
## Timepoint:Treatment     1.2430  0.6215     2 42.860  1.0297   0.36579
## Timepoint:Sex           0.3521  0.1760     2 44.585  0.2914   0.74864
## Treatment:Sex           0.3719  0.3719     1 43.519  0.6162   0.43673
## Timepoint:Treatment:Sex  2.6284  1.3142     2 44.585  2.1755   0.12546
## ---
## Signif. codes:  0 '***' 0.001 '**' 0.01 '*' 0.05 '.' 0.1 ' ' 1
```

```
Tnf_emcatcat_sex <- emmeans(Tnf_sex_effects, ~ Timepoint * Treatment * Sex)
#Is there a difference in males or females between treatment group at each time point?
contrast(Tnf_emcatcat_sex, "revpairwise", by=c("Sex", "Timepoint"), adjust="BH")
```

```
## Sex = F, Timepoint = d7:
##  contrast      estimate    SE   df t.ratio p.value
##  PNS - Control   1.1780 0.556 38.8   2.120  0.0404
##
## Sex = M, Timepoint = d7:
##  contrast      estimate    SE   df t.ratio p.value
##  PNS - Control   1.2447 0.895 46.0   1.390  0.1712
##
## Sex = F, Timepoint = d21:
##  contrast      estimate    SE   df t.ratio p.value
##  PNS - Control   1.3236 0.512 33.5   2.586  0.0142
##
## Sex = M, Timepoint = d21:
##  contrast      estimate    SE   df t.ratio p.value
##  PNS - Control  -0.4850 0.581 36.8  -0.835  0.4091
##
## Sex = F, Timepoint = d35:
##  contrast      estimate    SE   df t.ratio p.value
##  PNS - Control   0.0728 0.687 44.6   0.106  0.9161
##
## Sex = M, Timepoint = d35:
##  contrast      estimate    SE   df t.ratio p.value
##  PNS - Control   0.5826 0.687 44.7   0.849  0.4006
##
## Degrees-of-freedom method: kenward-roger
```

```
#Is there a difference in males or females within treatment group at each time point?
contrast(Tnf_emcatcat_sex, "revpairwise", by=c("Treatment", "Timepoint"), adjust="BH")
```

```
## Treatment = Control, Timepoint = d7:
##  contrast estimate    SE   df t.ratio p.value
##  M - F     -0.4679 0.892 44.1  -0.525  0.6024
##
## Treatment = PNS, Timepoint = d7:
##  contrast estimate    SE   df t.ratio p.value
##  M - F     -0.4012 0.531 45.7  -0.756  0.4538
##
## Treatment = Control, Timepoint = d21:
##  contrast estimate    SE   df t.ratio p.value
##  M - F      0.9414 0.616 40.9   1.528  0.1342
##
## Treatment = PNS, Timepoint = d21:
##  contrast estimate    SE   df t.ratio p.value
##  M - F     -0.8672 0.463 44.5  -1.875  0.0674
##
## Treatment = Control, Timepoint = d35:
##  contrast estimate    SE   df t.ratio p.value
##  M - F     -0.5307 0.667 43.2  -0.795  0.4309
##
## Treatment = PNS, Timepoint = d35:
##  contrast estimate    SE   df t.ratio p.value
##  M - F     -0.0209 0.655 41.0  -0.032  0.9746
##
## Degrees-of-freedom method: kenward-roger
```

Lung Tissue *Ahr* Expression Sex Differences

```
# Does lung Ahr expression differ by sex
#Include random intercept for litter
Ahr_sex_effects <- lmer(Ahr ~ Timepoint * Treatment * Sex + (1 | Litter), data = lung_dat)
Ahr_anova_table_for_sex <- anova(Ahr_sex_effects, ddf = "Kenward-Roger")
print(Ahr_anova_table_for_sex)
```

```
## Type III Analysis of Variance Table with Kenward-Roger's method
##                        Sum Sq Mean Sq NumDF  DenDF F value  Pr(>F)
## Timepoint              28.429 14.2147     2 44.389  2.7994 0.07162 .
## Treatment               4.560  4.5604     1 11.332  0.8981 0.36305
## Sex                     2.641  2.6410     1 45.684  0.5201 0.47446
## Timepoint:Treatment    40.556 20.2780     2 44.389  3.9935 0.02542 *
## Timepoint:Sex           8.292  4.1462     2 45.782  0.8157 0.44864
## Treatment:Sex           9.812  9.8120     1 45.684  1.9324 0.17123
## Timepoint:Treatment:Sex 14.024  7.0122     2 45.782  1.3796 0.26195
## ---
## Signif. codes:  0 '***' 0.001 '**' 0.01 '*' 0.05 '.' 0.1 ' ' 1
```

```
Ahr_emcatcat_sex <- emmeans(Ahr_sex_effects, ~ Timepoint * Treatment * Sex)
#Is there a difference in males or females between treatment group at each time point?
contrast(Ahr_emcatcat_sex, "revpairwise", by=c("Sex", "Timepoint"), adjust="BH")
```

```
## Sex = F, Timepoint = d7:
##  contrast      estimate   SE   df t.ratio p.value
##  PNS - Control    1.111 1.57 40.9   0.710  0.4820
##
## Sex = M, Timepoint = d7:
##  contrast      estimate   SE   df t.ratio p.value
##  PNS - Control    1.828 2.56 46.9   0.714  0.4789
##
## Sex = F, Timepoint = d21:
##  contrast      estimate   SE   df t.ratio p.value
##  PNS - Control    3.515 1.43 35.5   2.457  0.0191
##
## Sex = M, Timepoint = d21:
##  contrast      estimate   SE   df t.ratio p.value
##  PNS - Control    2.136 1.63 38.2   1.311  0.1978
##
## Sex = F, Timepoint = d35:
##  contrast      estimate   SE   df t.ratio p.value
##  PNS - Control    0.872 1.92 45.6   0.454  0.6517
##
## Sex = M, Timepoint = d35:
##  contrast      estimate   SE   df t.ratio p.value
##  PNS - Control   -4.761 1.95 45.9  -2.439  0.0187
##
## Degrees-of-freedom method: kenward-roger
```

```
#Is there a difference in males or females within treatment group at each time point?
contrast(Ahr_emcatcat_sex, "revpairwise", by=c("Treatment", "Timepoint"), adjust="BH")
```

```
## Treatment = Control, Timepoint = d7:
##  contrast estimate   SE   df t.ratio p.value
##  M - F      -1.082 2.57 46.0  -0.421  0.6757
##
## Treatment = PNS, Timepoint = d7:
##  contrast estimate   SE   df t.ratio p.value
##  M - F      -0.365 1.53 47.0  -0.239  0.8120
##
## Treatment = Control, Timepoint = d21:
##  contrast estimate   SE   df t.ratio p.value
##  M - F       1.209 1.73 39.5   0.698  0.4895
##
## Treatment = PNS, Timepoint = d21:
##  contrast estimate   SE   df t.ratio p.value
##  M - F      -0.170 1.33 46.4  -0.128  0.8989
##
## Treatment = Control, Timepoint = d35:
##  contrast estimate   SE   df t.ratio p.value
##  M - F       4.654 1.93 45.2   2.417  0.0198
##
## Treatment = PNS, Timepoint = d35:
##  contrast estimate   SE   df t.ratio p.value
##  M - F      -0.979 1.87 43.8  -0.523  0.6039
##
## Degrees-of-freedom method: kenward-roger
```

Lung Tissue *Muc5ac* Expression Sex Differences

```
# Does lung Muc5ac expression differ by sex
#Include random intercept for litter
Muc5ac_sex_effects <- lmer(Muc5ac ~ Timepoint * Treatment * Sex + (1 | Litter), data = lung_dat)
Muc5ac_anova_table_for_sex <- anova(Muc5ac_sex_effects, ddf = "Kenward-Roger")
print(Muc5ac_anova_table_for_sex)
```

```
## Type III Analysis of Variance Table with Kenward-Roger's method
##                         Sum Sq Mean Sq NumDF  DenDF F value   Pr(>F)
## Timepoint               3809.1 1904.54     2 45.336 12.0275 6.45e-05 ***
## Treatment                 99.1   99.06     1 11.157  0.6256 0.445467
## Sex                        7.5    7.48     1 46.400  0.0473 0.828844
## Timepoint:Treatment     1688.4  844.20     2 45.336  5.3313 0.008329 **
## Timepoint:Sex            201.2  100.62     2 46.751  0.6348 0.534560
## Treatment:Sex            115.4  115.36     1 46.400  0.7286 0.397736
## Timepoint:Treatment:Sex  221.8  110.89     2 46.751  0.6996 0.501915
## ---
## Signif. codes:  0 '***' 0.001 '**' 0.01 '*' 0.05 '.' 0.1 ' ' 1
```

```
Muc5ac_emcatcat_sex <- emmeans(Muc5ac_sex_effects, ~ Timepoint * Treatment * Sex)
#Is there a difference in males or females between treatment group at each time point?
contrast(Muc5ac_emcatcat_sex, "revpairwise", by=c("Sex", "Timepoint"), adjust="BH")
```

```
## Sex = F, Timepoint = d7:
##  contrast      estimate    SE   df t.ratio p.value
##  PNS - Control    21.94  8.68 41.8   2.527  0.0154
##
## Sex = M, Timepoint = d7:
##  contrast      estimate    SE   df t.ratio p.value
##  PNS - Control    11.87 14.20 47.8   0.833  0.4090
##
## Sex = F, Timepoint = d21:
##  contrast      estimate    SE   df t.ratio p.value
##  PNS - Control     7.02  7.92 36.4   0.887  0.3809
##
## Sex = M, Timepoint = d21:
##  contrast      estimate    SE   df t.ratio p.value
##  PNS - Control    12.03  8.20 34.9   1.468  0.1510
##
## Sex = F, Timepoint = d35:
##  contrast      estimate    SE   df t.ratio p.value
##  PNS - Control    -7.70 10.70 46.6  -0.722  0.4739
##
## Sex = M, Timepoint = d35:
##  contrast      estimate    SE   df t.ratio p.value
##  PNS - Control   -23.82 10.80 46.9  -2.196  0.0331
##
## Degrees-of-freedom method: kenward-roger
```

```
#Is there a difference in males or females within treatment group at each time point?
contrast(Muc5ac_emcatcat_sex, "revpairwise", by=c("Treatment", "Timepoint"), adjust="BH")
```

```
## Treatment = Control, Timepoint = d7:
##  contrast estimate    SE   df t.ratio p.value
##  M - F      -1.243 14.30 47.3  -0.087  0.9312
##
## Treatment = PNS, Timepoint = d7:
##  contrast estimate    SE   df t.ratio p.value
##  M - F     -11.321  8.50 48.0  -1.332  0.1890
##
## Treatment = Control, Timepoint = d21:
##  contrast estimate    SE   df t.ratio p.value
##  M - F       0.374  8.82 36.2   0.042  0.9664
##
## Treatment = PNS, Timepoint = d21:
##  contrast estimate    SE   df t.ratio p.value
##  M - F       5.384  7.42 47.5   0.726  0.4714
##
## Treatment = Control, Timepoint = d35:
##  contrast estimate    SE   df t.ratio p.value
##  M - F      14.164 10.70 46.3   1.319  0.1936
##
## Treatment = PNS, Timepoint = d35:
##  contrast estimate    SE   df t.ratio p.value
##  M - F      -1.960 10.50 45.1  -0.188  0.8521
##
## Degrees-of-freedom method: kenward-roger
```

Lung Tissue *Muc5b* Expression Sex Differences

```
# Does lung Muc5b expression differ by sex
#Include random intercept for litter
Muc5b_sex_effects <- lmer(Muc5b ~ Timepoint * Treatment * Sex + (1 | Litter), data = lung_dat)
```

```
## boundary (singular) fit: see help('isSingular')
```

```
Muc5b_anova_table_for_sex <- anova(Muc5b_sex_effects, ddf = "Kenward-Roger")
print(Muc5b_anova_table_for_sex)
```

```
## Type III Analysis of Variance Table with Kenward-Roger's method
##                         Sum Sq Mean Sq NumDF  DenDF F value    Pr(>F)
## Timepoint                62689 31344.3     2 45.164 39.9881 1.012e-10 ***
## Treatment                  791   790.9     1 12.876  1.0091    0.3336
## Sex                        292   292.5     1 45.274  0.3731    0.5444
## Timepoint:Treatment       3669  1834.6     2 45.164  2.3405    0.1079
## Timepoint:Sex              581   290.5     2 44.089  0.3702    0.6928
## Treatment:Sex               97    96.8     1 45.274  0.1234    0.7270
## Timepoint:Treatment:Sex    304   152.0     2 44.089  0.1938    0.8246
## ---
## Signif. codes:  0 '***' 0.001 '**' 0.01 '*' 0.05 '.' 0.1 ' ' 1
```

```
Muc5b_emcatcat_sex <- emmeans(Muc5b_sex_effects, ~ Timepoint * Treatment * Sex)
#Is there a difference in males or females between treatment group at each time point?
contrast(Muc5b_emcatcat_sex, "revpairwise", by=c("Sex", "Timepoint"), adjust="BH")
```

```
## Sex = F, Timepoint = d7:
##  contrast      estimate   SE   df t.ratio p.value
##  PNS - Control    22.78 18.8 39.8   1.213  0.2323
##
## Sex = M, Timepoint = d7:
##  contrast      estimate   SE   df t.ratio p.value
##  PNS - Control     7.92 31.3 45.4   0.253  0.8011
##
## Sex = F, Timepoint = d21:
##  contrast      estimate   SE   df t.ratio p.value
##  PNS - Control    28.04 16.9 35.7   1.660  0.1057
##
## Sex = M, Timepoint = d21:
##  contrast      estimate   SE   df t.ratio p.value
##  PNS - Control    36.75 19.3 37.4   1.900  0.0651
##
## Sex = F, Timepoint = d35:
##  contrast      estimate   SE   df t.ratio p.value
##  PNS - Control   -10.88 23.2 44.5  -0.469  0.6414
##
## Sex = M, Timepoint = d35:
##  contrast      estimate   SE   df t.ratio p.value
##  PNS - Control   -25.54 31.7 45.2  -0.807  0.4239
##
## Degrees-of-freedom method: kenward-roger
```

```
#Is there a difference in males or females within treatment group at each time point?
contrast(Muc5b_emcatcat_sex, "revpairwise", by=c("Treatment", "Timepoint"), adjust="BH")
```

```
## Treatment = Control, Timepoint = d7:
##  contrast estimate   SE   df t.ratio p.value
##  M - F        1.06 31.7 46.0   0.034  0.9734
##
## Treatment = PNS, Timepoint = d7:
##  contrast estimate   SE   df t.ratio p.value
##  M - F      -13.80 18.8 44.7  -0.733  0.4677
##
## Treatment = Control, Timepoint = d21:
##  contrast estimate   SE   df t.ratio p.value
##  M - F        3.73 20.5 34.6   0.182  0.8568
##
## Treatment = PNS, Timepoint = d21:
##  contrast estimate   SE   df t.ratio p.value
##  M - F       12.44 16.3 46.0   0.761  0.4504
##
## Treatment = Control, Timepoint = d35:
##  contrast estimate   SE   df t.ratio p.value
##  M - F       23.71 23.8 45.5   0.998  0.3234
##
## Treatment = PNS, Timepoint = d35:
##  contrast estimate   SE   df t.ratio p.value
##  M - F        9.05 31.2 45.1   0.290  0.7729
##
## Degrees-of-freedom method: kenward-roger
```

Lung Tissue *Il10* Expression Sex Differences

```
# Does lung IL10 expression differ by sex
#Include random intercept for litter
IL10_sex_effects <- lmer(IL10 ~ Timepoint * Treatment * Sex + (1 | Litter), data = lung_dat)
IL10_anova_table_for_sex <- anova(IL10_sex_effects, ddf = "Kenward-Roger")
print(IL10_anova_table_for_sex)
```

```
## Type III Analysis of Variance Table with Kenward-Roger's method
##                         Sum Sq Mean Sq NumDF  DenDF F value    Pr(>F)
## Timepoint              2028.86 1014.43     2 39.086 36.7325 1.055e-09 ***
## Treatment                 1.17    1.17     1  9.736  0.0425    0.8410
## Sex                       0.48    0.48     1 39.425  0.0173    0.8961
## Timepoint:Treatment      67.63   33.81     2 39.086  1.2244    0.3050
## Timepoint:Sex            11.94    5.97     2 40.964  0.2161    0.8066
## Treatment:Sex            70.52   70.52     1 39.425  2.5536    0.1180
## Timepoint:Treatment:Sex  75.87   37.93     2 40.964  1.3733    0.2647
## ---
## Signif. codes:  0 '***' 0.001 '**' 0.01 '*' 0.05 '.' 0.1 ' ' 1
```

```
IL10_emcatcat_sex <- emmeans(IL10_sex_effects, ~ Timepoint * Treatment * Sex)
#Is there a difference in males or females between treatment group at each time point?
contrast(IL10_emcatcat_sex, "revpairwise", by=c("Sex", "Timepoint"), adjust="BH")
```

```
## Sex = F, Timepoint = d7:
##  contrast      estimate   SE   df t.ratio p.value
##  PNS - Control   -2.380 4.88 24.4  -0.487  0.6303
##
## Sex = M, Timepoint = d7:
##  contrast      estimate   SE   df t.ratio p.value
##  PNS - Control   10.400 6.85 43.0   1.519  0.1361
##
## Sex = F, Timepoint = d21:
##  contrast      estimate   SE   df t.ratio p.value
##  PNS - Control    2.676 4.71 21.2   0.568  0.5762
##
## Sex = M, Timepoint = d21:
##  contrast      estimate   SE   df t.ratio p.value
##  PNS - Control    0.165 5.19 26.5   0.032  0.9748
##
## Sex = F, Timepoint = d35:
##  contrast      estimate   SE   df t.ratio p.value
##  PNS - Control   -6.685 5.55 33.0  -1.205  0.2369
##
## Sex = M, Timepoint = d35:
##  contrast      estimate   SE   df t.ratio p.value
##  PNS - Control    0.381 5.62 33.7   0.068  0.9464
##
## Degrees-of-freedom method: kenward-roger
```

```
#Is there a difference in males or females within treatment group at each time point?
contrast(IL10_emcatcat_sex, "revpairwise", by=c("Treatment", "Timepoint"), adjust="BH")
```

```
## Treatment = Control, Timepoint = d7:
##  contrast estimate   SE   df t.ratio p.value
##  M - F     -8.3512 6.15 39.4  -1.359  0.1820
##
## Treatment = PNS, Timepoint = d7:
##  contrast estimate   SE   df t.ratio p.value
##  M - F      4.4293 3.66 39.9   1.210  0.2335
##
## Treatment = Control, Timepoint = d21:
##  contrast estimate   SE   df t.ratio p.value
##  M - F      2.4788 4.73 45.4   0.524  0.6026
##
## Treatment = PNS, Timepoint = d21:
##  contrast estimate   SE   df t.ratio p.value
##  M - F     -0.0321 3.34 40.3  -0.010  0.9924
##
## Treatment = Control, Timepoint = d35:
##  contrast estimate   SE   df t.ratio p.value
##  M - F     -3.5079 4.58 38.9  -0.766  0.4483
##
## Treatment = PNS, Timepoint = d35:
##  contrast estimate   SE   df t.ratio p.value
##  M - F      3.5574 4.42 38.3   0.806  0.4254
##
## Degrees-of-freedom method: kenward-roger
```

Lung Tissue *Tgfb1* Expression Sex Differences

```
# Does lung Tgfb1 expression differ by sex
#Include random intercept for litter
Tgfb1_sex_effects <- lmer(Tgfb1 ~ Timepoint * Treatment * Sex + (1 | Litter), data = lung_dat)
Tgfb1_anova_table_for_sex <- anova(Tgfb1_sex_effects, ddf = "Kenward-Roger")
print(Tgfb1_anova_table_for_sex)
```

```
## Type III Analysis of Variance Table with Kenward-Roger's method
##                         Sum Sq Mean Sq NumDF  DenDF F value    Pr(>F)
## Timepoint              18.2138  9.1069     2 38.566 36.8367 1.132e-09 ***
## Treatment               0.0342  0.0342     1  9.255  0.1385   0.71813
## Sex                     0.0008  0.0008     1 38.795  0.0034   0.95409
## Timepoint:Treatment     1.6720  0.8360     2 38.566  3.3815   0.04435 *
## Timepoint:Sex           0.8212  0.4106     2 39.362  1.6608   0.20303
## Treatment:Sex           0.3531  0.3531     1 38.795  1.4284   0.23928
## Timepoint:Treatment:Sex  0.7702  0.3851     2 39.362  1.5577   0.22332
## ---
## Signif. codes:  0 '***' 0.001 '**' 0.01 '*' 0.05 '.' 0.1 ' ' 1
```

```
Tgfb1_emcatcat_sex <- emmeans(Tgfb1_sex_effects, ~ Timepoint * Treatment * Sex)
#Is there a difference in males or females between treatment group at each time point?
contrast(Tgfb1_emcatcat_sex, "revpairwise", by=c("Sex", "Timepoint"), adjust="BH")
```

```
## Sex = F, Timepoint = d7:
##  contrast      estimate    SE   df t.ratio p.value
##  PNS - Control   0.6533 0.710 13.6   0.920  0.3734
##
## Sex = M, Timepoint = d7:
##  contrast      estimate    SE   df t.ratio p.value
##  PNS - Control   1.0890 0.844 24.2   1.291  0.2089
##
## Sex = F, Timepoint = d21:
##  contrast      estimate    SE   df t.ratio p.value
##  PNS - Control   0.7069 0.701 12.9   1.008  0.3319
##
## Sex = M, Timepoint = d21:
##  contrast      estimate    SE   df t.ratio p.value
##  PNS - Control  -0.4875 0.728 14.7  -0.670  0.5133
##
## Sex = F, Timepoint = d35:
##  contrast      estimate    SE   df t.ratio p.value
##  PNS - Control  -0.0271 0.753 16.8  -0.036  0.9717
##
## Sex = M, Timepoint = d35:
##  contrast      estimate    SE   df t.ratio p.value
##  PNS - Control  -0.5023 0.757 17.1  -0.664  0.5158
##
## Degrees-of-freedom method: kenward-roger
```

```
#Is there a difference in males or females within treatment group at each time point?
contrast(Tgfb1_emcatcat_sex, "revpairwise", by=c("Treatment", "Timepoint"), adjust="BH")
```

```
## Treatment = Control, Timepoint = d7:
##  contrast estimate    SE   df t.ratio p.value
##  M - F      -0.695 0.585 38.7  -1.189  0.2419
##
## Treatment = PNS, Timepoint = d7:
##  contrast estimate    SE   df t.ratio p.value
##  M - F      -0.259 0.347 38.8  -0.748  0.4590
##
## Treatment = Control, Timepoint = d21:
##  contrast estimate    SE   df t.ratio p.value
##  M - F       0.756 0.465 41.7   1.626  0.1115
##
## Treatment = PNS, Timepoint = d21:
##  contrast estimate    SE   df t.ratio p.value
##  M - F      -0.439 0.304 38.7  -1.444  0.1568
##
## Treatment = Control, Timepoint = d35:
##  contrast estimate    SE   df t.ratio p.value
##  M - F       0.586 0.435 38.5   1.348  0.1856
##
## Treatment = PNS, Timepoint = d35:
##  contrast estimate    SE   df t.ratio p.value
##  M - F       0.111 0.418 38.3   0.266  0.7920
##
## Degrees-of-freedom method: kenward-roger
```

Lung Tissue *Foxp3* Expression Sex Differences

```
# Does lung Foxp3 expression differ by sex
#Include random intercept for litter
Foxp3_sex_effects <- lmer(Foxp3 ~ Timepoint * Treatment * Sex + (1 | Litter), data = lung_dat)
Foxp3_anova_table_for_sex <- anova(Foxp3_sex_effects, ddf = "Kenward-Roger")
print(Foxp3_anova_table_for_sex)
```

```
## Type III Analysis of Variance Table with Kenward-Roger's method
##                         Sum Sq Mean Sq NumDF  DenDF F value    Pr(>F)
## Timepoint               8376.2  4188.1     2 42.277 22.9533 1.782e-07 ***
## Treatment                 72.0    72.0     1 10.934  0.3946  0.542782
## Sex                        6.8     6.8     1 43.577  0.0374  0.847634
## Timepoint:Treatment     2610.8  1305.4     2 42.277  7.1544  0.002108 **
## Timepoint:Sex            173.0    86.5     2 44.410  0.4736  0.625848
## Treatment:Sex            558.0   558.0     1 43.577  3.0584  0.087363 .
## Timepoint:Treatment:Sex  366.5   183.3     2 44.410  1.0036  0.374727
## ---
## Signif. codes:  0 '***' 0.001 '**' 0.01 '*' 0.05 '.' 0.1 ' ' 1
```

```
Foxp3_emcatcat_sex <- emmeans(Foxp3_sex_effects, ~ Timepoint * Treatment * Sex)
#Is there a difference in males or females between treatment group at each time point?
contrast(Foxp3_emcatcat_sex, "revpairwise", by=c("Sex", "Timepoint"), adjust="BH")
```

```
## Sex = F, Timepoint = d7:
##  contrast      estimate   SE   df t.ratio p.value
##  PNS - Control    -1.66 10.8 40.0  -0.153  0.8790
##
## Sex = M, Timepoint = d7:
##  contrast      estimate   SE   df t.ratio p.value
##  PNS - Control     2.62 15.7 46.0   0.167  0.8682
##
## Sex = F, Timepoint = d21:
##  contrast      estimate   SE   df t.ratio p.value
##  PNS - Control    38.16  9.1 32.5   4.192  0.0002
##
## Sex = M, Timepoint = d21:
##  contrast      estimate   SE   df t.ratio p.value
##  PNS - Control     9.92 10.3 36.2   0.963  0.3420
##
## Sex = F, Timepoint = d35:
##  contrast      estimate   SE   df t.ratio p.value
##  PNS - Control    -1.48 11.9 44.1  -0.124  0.9018
##
## Sex = M, Timepoint = d35:
##  contrast      estimate   SE   df t.ratio p.value
##  PNS - Control   -26.16 12.1 44.5  -2.166  0.0357
##
## Degrees-of-freedom method: kenward-roger
```

```
#Is there a difference in males or females within treatment group at each time point?
contrast(Foxp3_emcatcat_sex, "revpairwise", by=c("Treatment", "Timepoint"), adjust="BH")
```

```
## Treatment = Control, Timepoint = d7:
##  contrast estimate    SE   df t.ratio p.value
##  M - F       -1.55 15.50 43.6  -0.099  0.9213
##
## Treatment = PNS, Timepoint = d7:
##  contrast estimate    SE   df t.ratio p.value
##  M - F        2.73 10.40 45.6   0.263  0.7934
##
## Treatment = Control, Timepoint = d21:
##  contrast estimate    SE   df t.ratio p.value
##  M - F       10.13 10.90 41.9   0.930  0.3577
##
## Treatment = PNS, Timepoint = d21:
##  contrast estimate    SE   df t.ratio p.value
##  M - F      -18.11  8.07 44.0  -2.244  0.0299
##
## Treatment = Control, Timepoint = d35:
##  contrast estimate    SE   df t.ratio p.value
##  M - F       18.42 11.60 42.7   1.584  0.1206
##
## Treatment = PNS, Timepoint = d35:
##  contrast estimate    SE   df t.ratio p.value
##  M - F       -6.26 11.30 41.3  -0.555  0.5820
##
## Degrees-of-freedom method: kenward-roger
```

Lung Tissue *Il18* Expression Sex Differences

```
# Does lung IL18 expression differ by sex
#Include random intercept for litter
IL18_sex_effects <- lmer(IL18 ~ Timepoint * Treatment * Sex + (1 | Litter), data = lung_dat)
IL18_anova_table_for_sex <- anova(IL18_sex_effects, ddf = "Kenward-Roger")
print(IL18_anova_table_for_sex)
```

```
## Type III Analysis of Variance Table with Kenward-Roger's method
##                         Sum Sq Mean Sq NumDF  DenDF F value    Pr(>F)    
## Timepoint              30.5916 15.2958     2 39.711 15.7148 9.385e-06 ***
## Treatment               0.1929  0.1929     1 10.216  0.1982   0.66547    
## Sex                     2.7878  2.7878     1 40.199  2.8642   0.09831 .  
## Timepoint:Treatment     0.4489  0.2244     2 39.711  0.2306   0.79513    
## Timepoint:Sex           0.3145  0.1573     2 41.959  0.1615   0.85136    
## Treatment:Sex           0.7588  0.7588     1 40.199  0.7796   0.38252    
## Timepoint:Treatment:Sex  0.5853  0.2926     2 41.959  0.3006   0.74198    
## ---
## Signif. codes:  0 '***' 0.001 '**' 0.01 '*' 0.05 '.' 0.1 ' ' 1
```

```
IL18_emcatcat_sex <- emmeans(IL18_sex_effects, ~ Timepoint * Treatment * Sex)
#Is there a difference in males or females between treatment group at each time point?
contrast(IL18_emcatcat_sex, "revpairwise", by=c("Sex", "Timepoint"), adjust="BH")
```

```
## Sex = F, Timepoint = d7:
##  contrast      estimate    SE   df t.ratio p.value
##  PNS - Control    0.537 0.877 33.5   0.613  0.5443
##
## Sex = M, Timepoint = d7:
##  contrast      estimate    SE   df t.ratio p.value
##  PNS - Control    0.661 1.230 45.3   0.539  0.5925
##
## Sex = F, Timepoint = d21:
##  contrast      estimate    SE   df t.ratio p.value
##  PNS - Control    0.794 0.795 25.2   0.998  0.3275
##
## Sex = M, Timepoint = d21:
##  contrast      estimate    SE   df t.ratio p.value
##  PNS - Control   -0.471 0.877 30.3  -0.537  0.5953
##
## Sex = F, Timepoint = d35:
##  contrast      estimate    SE   df t.ratio p.value
##  PNS - Control    0.345 0.968 38.6   0.356  0.7238
##
## Sex = M, Timepoint = d35:
##  contrast      estimate    SE   df t.ratio p.value
##  PNS - Control   -0.314 0.980 39.2  -0.320  0.7504
##
## Degrees-of-freedom method: kenward-roger
```

```
#Is there a difference in males or females within treatment group at each time point?
contrast(IL18_emcatcat_sex, "revpairwise", by=c("Treatment", "Timepoint"), adjust="BH")
```

```
## Treatment = Control, Timepoint = d7:
##  contrast estimate    SE   df t.ratio p.value
##  M - F      -0.677 1.150 40.4  -0.589  0.5592
##
## Treatment = PNS, Timepoint = d7:
##  contrast estimate    SE   df t.ratio p.value
##  M - F      -0.553 0.733 40.2  -0.755  0.4545
##
## Treatment = Control, Timepoint = d21:
##  contrast estimate    SE   df t.ratio p.value
##  M - F       0.295 0.865 46.0   0.341  0.7346
##
## Treatment = PNS, Timepoint = d21:
##  contrast estimate    SE   df t.ratio p.value
##  M - F      -0.970 0.598 40.6  -1.623  0.1124
##
## Treatment = Control, Timepoint = d35:
##  contrast estimate    SE   df t.ratio p.value
##  M - F      -0.443 0.858 39.7  -0.516  0.6084
##
## Treatment = PNS, Timepoint = d35:
##  contrast estimate    SE   df t.ratio p.value
##  M - F      -1.101 0.827 38.9  -1.331  0.1910
##
## Degrees-of-freedom method: kenward-roger
```

Lung Tissue *Il4* Expression Sex Differences

```
# Does lung IL4 expression differ by sex
#Include random intercept for litter
IL4_sex_effects <- lmer(IL4 ~ Timepoint * Treatment * Sex + (1 | Litter), data = lung_dat)
IL4_anova_table_for_sex <- anova(IL4_sex_effects, ddf = "Kenward-Roger")
print(IL4_anova_table_for_sex)
```

```
## Type III Analysis of Variance Table with Kenward-Roger's method
##                          Sum Sq  Mean Sq NumDF  DenDF F value Pr(>F)
## Timepoint               0.15262 0.076312     2 33.777  0.2488 0.7812
## Treatment               0.07472 0.074722     1 10.134  0.2436 0.6321
## Sex                     0.04579 0.045791     1 34.277  0.1493 0.7016
## Timepoint:Treatment     0.52574 0.262869     2 33.777  0.8570 0.4334
## Timepoint:Sex           0.23257 0.116286     2 35.359  0.3791 0.6872
## Treatment:Sex           0.00014 0.000145     1 34.277  0.0005 0.9828
## Timepoint:Treatment:Sex 0.01938 0.009688     2 35.359  0.0316 0.9689
```

```
IL4_emcatcat_sex <- emmeans(IL4_sex_effects, ~ Timepoint * Treatment * Sex)
#Is there a difference in males or females between treatment group at each time point?
contrast(IL4_emcatcat_sex, "revpairwise", by=c("Sex", "Timepoint"), adjust="BH")
```

```
## Sex = F, Timepoint = d7:
##  contrast      estimate    SE   df t.ratio p.value
##  PNS - Control   0.4316 0.563 25.9   0.767  0.4500
##
## Sex = M, Timepoint = d7:
##  contrast      estimate    SE   df t.ratio p.value
##  PNS - Control   0.5732 0.730 38.7   0.785  0.4370
##
## Sex = F, Timepoint = d21:
##  contrast      estimate    SE   df t.ratio p.value
##  PNS - Control  -0.0321 0.512 20.3  -0.063  0.9506
##
## Sex = M, Timepoint = d21:
##  contrast      estimate    SE   df t.ratio p.value
##  PNS - Control  -0.1498 0.556 24.1  -0.269  0.7899
##
## Sex = F, Timepoint = d35:
##  contrast      estimate    SE   df t.ratio p.value
##  PNS - Control   0.2253 0.602 30.6   0.374  0.7109
##
## Sex = M, Timepoint = d35:
##  contrast      estimate    SE   df t.ratio p.value
##  PNS - Control   0.1743 0.747 39.0   0.233  0.8167
##
## Degrees-of-freedom method: kenward-roger
```

```
#Is there a difference in males or females within treatment group at each time point?
contrast(IL4_emcatcat_sex, "revpairwise", by=c("Treatment", "Timepoint"), adjust="BH")
```

```
## Treatment = Control, Timepoint = d7:
##  contrast estimate    SE   df t.ratio p.value
##  M - F    -0.00401 0.649 34.0  -0.006  0.9951
##
## Treatment = PNS, Timepoint = d7:
##  contrast estimate    SE   df t.ratio p.value
##  M - F     0.13760 0.442 35.5   0.311  0.7575
##
## Treatment = Control, Timepoint = d21:
##  contrast estimate    SE   df t.ratio p.value
##  M - F    -0.26425 0.507 39.8  -0.521  0.6052
##
## Treatment = PNS, Timepoint = d21:
##  contrast estimate    SE   df t.ratio p.value
##  M - F    -0.38190 0.342 34.1  -1.117  0.2719
##
## Treatment = Control, Timepoint = d35:
##  contrast estimate    SE   df t.ratio p.value
##  M - F     0.04155 0.491 33.5   0.085  0.9330
##
## Treatment = PNS, Timepoint = d35:
##  contrast estimate    SE   df t.ratio p.value
##  M - F    -0.00944 0.627 33.1  -0.015  0.9881
##
## Degrees-of-freedom method: kenward-roger
```

Lung Tissue *Il5* Expression Sex Differences

```
# Does lung IL5 expression differ by sex
#Include random intercept for litter
IL5_sex_effects <- lmer(IL5 ~ Timepoint * Treatment * Sex + (1 | Litter), data = lung_dat)
IL5_anova_table_for_sex <- anova(IL5_sex_effects, ddf = "Kenward-Roger")
print(IL5_anova_table_for_sex)
```

```
## Type III Analysis of Variance Table with Kenward-Roger's method
##                        Sum Sq Mean Sq NumDF  DenDF F value    Pr(>F)
## Timepoint              45.083 22.5414     2 36.063 12.4074 7.938e-05 ***
## Treatment               3.245  3.2448     1  9.825  1.7860   0.21154
## Sex                     5.974  5.9743     1 36.330  3.2884   0.07804 .
## Timepoint:Treatment     5.750  2.8749     2 36.063  1.5824   0.21941
## Timepoint:Sex           0.795  0.3973     2 37.460  0.2187   0.80462
## Treatment:Sex           1.075  1.0751     1 36.330  0.5918   0.44671
## Timepoint:Treatment:Sex  3.874  1.9368     2 37.460  1.0660   0.35460
## ---
## Signif. codes:  0 '***' 0.001 '**' 0.01 '*' 0.05 '.' 0.1 ' ' 1
```

```
IL5_emcatcat_sex <- emmeans(IL5_sex_effects, ~ Timepoint * Treatment * Sex)
#Is there a difference in males or females between treatment group at each time point?
contrast(IL5_emcatcat_sex, "revpairwise", by=c("Sex", "Timepoint"), adjust="BH")
```

```
## Sex = F, Timepoint = d7:
##  contrast      estimate   SE   df t.ratio p.value
##  PNS - Control    4.381 1.54 21.2   2.842  0.0097
##
## Sex = M, Timepoint = d7:
##  contrast      estimate   SE   df t.ratio p.value
##  PNS - Control    1.450 1.92 35.3   0.757  0.4543
##
## Sex = F, Timepoint = d21:
##  contrast      estimate   SE   df t.ratio p.value
##  PNS - Control    1.034 1.44 17.0   0.719  0.4818
##
## Sex = M, Timepoint = d21:
##  contrast      estimate   SE   df t.ratio p.value
##  PNS - Control    1.668 1.47 17.8   1.134  0.2717
##
## Sex = F, Timepoint = d35:
##  contrast      estimate   SE   df t.ratio p.value
##  PNS - Control    0.656 1.61 24.4   0.406  0.6883
##
## Sex = M, Timepoint = d35:
##  contrast      estimate   SE   df t.ratio p.value
##  PNS - Control    0.684 1.94 35.6   0.353  0.7261
##
## Degrees-of-freedom method: kenward-roger
```

```
#Is there a difference in males or females within treatment group at each time point?
contrast(IL5_emcatcat_sex, "revpairwise", by=c("Treatment", "Timepoint"), adjust="BH")
```

```
## Treatment = Control, Timepoint = d7:
##  contrast estimate    SE   df t.ratio p.value
##  M - F      0.0649 1.580 36.4   0.041  0.9675
##
## Treatment = PNS, Timepoint = d7:
##  contrast estimate    SE   df t.ratio p.value
##  M - F     -2.8663 1.060 37.0  -2.698  0.0105
##
## Treatment = Control, Timepoint = d21:
##  contrast estimate    SE   df t.ratio p.value
##  M - F     -1.0499 1.160 41.4  -0.905  0.3709
##
## Treatment = PNS, Timepoint = d21:
##  contrast estimate    SE   df t.ratio p.value
##  M - F     -0.4160 0.833 36.4  -0.499  0.6206
##
## Treatment = Control, Timepoint = d35:
##  contrast estimate    SE   df t.ratio p.value
##  M - F     -0.5551 1.170 36.0  -0.474  0.6386
##
## Treatment = PNS, Timepoint = d35:
##  contrast estimate    SE   df t.ratio p.value
##  M - F     -0.5271 1.520 35.8  -0.347  0.7309
##
## Degrees-of-freedom method: kenward-roger
```

Lung Tissue *Il13* Expression Sex Differences

```
# Does lung IL13 expression differ by sex
#Include random intercept for litter
IL13_sex_effects <- lmer(IL13 ~ Timepoint * Treatment * Sex + (1 | Litter), data = lung_dat)
```

```
## boundary (singular) fit: see help('isSingular')
```

```
IL13_anova_table_for_sex <- anova(IL13_sex_effects, ddf = "Kenward-Roger")
print(IL13_anova_table_for_sex)
```

```
## Type III Analysis of Variance Table with Kenward-Roger's method
##                         Sum Sq Mean Sq NumDF  DenDF F value    Pr(>F)
## Timepoint               4268.2 2134.10     2 43.846 36.4840 4.681e-10 ***
## Treatment                208.9  208.92     1 10.392  3.5716   0.08697 .
## Sex                      116.3  116.30     1 45.343  1.9882   0.16535
## Timepoint:Treatment      287.3  143.66     2 43.846  2.4560   0.09750 .
## Timepoint:Sex            101.0   50.51     2 43.971  0.8622   0.42925
## Treatment:Sex             76.9   76.87     1 45.343  1.3142   0.25765
## Timepoint:Treatment:Sex  136.1   68.06     2 43.971  1.1619   0.32231
## ---
## Signif. codes:  0 '***' 0.001 '**' 0.01 '*' 0.05 '.' 0.1 ' ' 1
```

```
IL13_emcatcat_sex <- emmeans(IL13_sex_effects, ~ Timepoint * Treatment * Sex)
#Is there a difference in males or females between treatment group at each time point?
contrast(IL13_emcatcat_sex, "revpairwise", by=c("Sex", "Timepoint"), adjust="BH")
```

```
## Sex = F, Timepoint = d7:
##  contrast      estimate   SE   df t.ratio p.value
##  PNS - Control  21.8513 5.12 40.1   4.267  0.0001
##
## Sex = M, Timepoint = d7:
##  contrast      estimate   SE   df t.ratio p.value
##  PNS - Control   4.1846 8.52 45.4   0.491  0.6255
##
## Sex = F, Timepoint = d21:
##  contrast      estimate   SE   df t.ratio p.value
##  PNS - Control   1.7323 4.77 38.6   0.363  0.7184
##
## Sex = M, Timepoint = d21:
##  contrast      estimate   SE   df t.ratio p.value
##  PNS - Control   1.3655 4.86 34.7   0.281  0.7804
##
## Sex = F, Timepoint = d35:
##  contrast      estimate   SE   df t.ratio p.value
##  PNS - Control  -0.8394 6.33 44.6  -0.133  0.8952
##
## Sex = M, Timepoint = d35:
##  contrast      estimate   SE   df t.ratio p.value
##  PNS - Control  -0.0998 6.45 44.8  -0.015  0.9877
##
## Degrees-of-freedom method: kenward-roger
```

```
#Is there a difference in males or females within treatment group at each time point?
contrast(IL13_emcatcat_sex, "revpairwise", by=c("Treatment", "Timepoint"), adjust="BH")
```

```
## Treatment = Control, Timepoint = d7:
##  contrast estimate   SE   df t.ratio p.value
##  M - F       0.203 8.64 46.0   0.023  0.9814
##
## Treatment = PNS, Timepoint = d7:
##  contrast estimate   SE   df t.ratio p.value
##  M - F     -17.464 5.13 44.8  -3.402  0.0014
##
## Treatment = Control, Timepoint = d21:
##  contrast estimate   SE   df t.ratio p.value
##  M - F      -0.735 5.29 33.3  -0.139  0.8903
##
## Treatment = PNS, Timepoint = d21:
##  contrast estimate   SE   df t.ratio p.value
##  M - F      -1.102 4.55 45.9  -0.242  0.8099
##
## Treatment = Control, Timepoint = d35:
##  contrast estimate   SE   df t.ratio p.value
##  M - F      -1.456 6.50 45.5  -0.224  0.8236
##
## Treatment = PNS, Timepoint = d35:
##  contrast estimate   SE   df t.ratio p.value
##  M - F      -0.717 6.34 44.5  -0.113  0.9105
##
## Degrees-of-freedom method: kenward-roger
```

Lung Tissue *Il17a* Expression Sex Differences

```
# Does lung IL17 expression differ by sex
#Include random intercept for litter
IL17_sex_effects <- lmer(IL17 ~ Timepoint * Treatment * Sex + (1 | Litter), data = lung_dat)
IL17_anova_table_for_sex <- anova(IL17_sex_effects, ddf = "Kenward-Roger")
print(IL17_anova_table_for_sex)
```

```
## Type III Analysis of Variance Table with Kenward-Roger's method
##                         Sum Sq Mean Sq NumDF  DenDF F value   Pr(>F)
## Timepoint               783.66  391.83     2 34.538  4.2779 0.021884 *
## Treatment                 0.60    0.60     1 10.586  0.0065 0.937222
## Sex                       2.31    2.31     1 36.055  0.0252 0.874682
## Timepoint:Treatment     107.40   53.70     2 34.538  0.5863 0.561832
## Timepoint:Sex           121.30   60.65     2 35.466  0.6619 0.522133
## Treatment:Sex            35.73   35.73     1 36.055  0.3901 0.536160
## Timepoint:Treatment:Sex 1106.86  553.43     2 35.466  6.0396 0.005529 **
## ---
## Signif. codes:  0 '***' 0.001 '**' 0.01 '*' 0.05 '.' 0.1 ' ' 1
```

```
IL17_emcatcat_sex <- emmeans(IL17_sex_effects, ~ Timepoint * Treatment * Sex)
#Is there a difference in males or females between treatment group at each time point?
contrast(IL17_emcatcat_sex, "revpairwise", by=c("Sex", "Timepoint"), adjust="BH")
```

```
## Sex = F, Timepoint = d7:
##  contrast      estimate    SE   df t.ratio p.value
##  PNS - Control   -13.34  8.72 23.5  -1.530  0.1393
##
## Sex = M, Timepoint = d7:
##  contrast      estimate    SE   df t.ratio p.value
##  PNS - Control     2.79 12.40 38.7   0.224  0.8240
##
## Sex = F, Timepoint = d21:
##  contrast      estimate    SE   df t.ratio p.value
##  PNS - Control    18.03  8.50 21.2   2.121  0.0459
##
## Sex = M, Timepoint = d21:
##  contrast      estimate    SE   df t.ratio p.value
##  PNS - Control   -13.54 10.90 30.1  -1.246  0.2222
##
## Sex = F, Timepoint = d35:
##  contrast      estimate    SE   df t.ratio p.value
##  PNS - Control   -10.86 12.80 38.0  -0.851  0.4003
##
## Sex = M, Timepoint = d35:
##  contrast      estimate    SE   df t.ratio p.value
##  PNS - Control    20.19 13.00 39.1   1.557  0.1275
##
## Degrees-of-freedom method: kenward-roger
```

```
#Is there a difference in males or females within treatment group at each time point?
contrast(IL17_emcatcat_sex, "revpairwise", by=c("Treatment", "Timepoint"), adjust="BH")
```

```
## Treatment = Control, Timepoint = d7:
##  contrast estimate    SE   df t.ratio p.value
##  M - F      -11.93 11.40 34.1  -1.049  0.3014
##
## Treatment = PNS, Timepoint = d7:
##  contrast estimate    SE   df t.ratio p.value
##  M - F        4.19  6.67 33.9   0.628  0.5343
##
## Treatment = Control, Timepoint = d21:
##  contrast estimate    SE   df t.ratio p.value
##  M - F       11.68 10.60 40.0   1.105  0.2758
##
## Treatment = PNS, Timepoint = d21:
##  contrast estimate    SE   df t.ratio p.value
##  M - F      -19.89  6.13 34.7  -3.244  0.0026
##
## Treatment = Control, Timepoint = d35:
##  contrast estimate    SE   df t.ratio p.value
##  M - F       -9.54 11.80 34.5  -0.809  0.4241
##
## Treatment = PNS, Timepoint = d35:
##  contrast estimate    SE   df t.ratio p.value
##  M - F       21.52 11.10 33.4   1.934  0.0617
##
## Degrees-of-freedom method: kenward-roger
```

Supplementary File 2. Offspring Ileum Gene Expression Analysis

```
library(tidyverse)
library(lme4)
library(lmerTest)
library(emmeans)
```

Ileum Tissue Gene Expression–Figure 2

Read in Ileum Gene Expression Data

```
ileum_dat <- read_csv("IT_expression.csv")
```

```
## Rows: 82 Columns: 21
## ── Column specification ────────────────────────────────────────────────────────
## Delimiter: ","
## chr  (5): SampleID, Treatment, Timepoint, Sex, Litter
## dbl (16): Ahr, Foxp3, Ifng, Ifnb, IL1b, IL4, IL5, IL6, IL10, IL13, IL18, Isg...
##
## ℹ Use `spec()` to retrieve the full column specification for this data.
## ℹ Specify the column types or set `show_col_types = FALSE` to quiet this message.
```

```
summary(ileum_dat)
```

```
##    SampleID          Treatment          Timepoint             Sex
##  Length:82          Length:82          Length:82          Length:82
##  Class :character   Class :character   Class :character   Class :character
##  Mode  :character   Mode  :character   Mode  :character   Mode  :character
##
##
##
##
##     Litter               Ahr             Foxp3              Ifng
##  Length:82          Min.   :0.3430   Min.   :  0.101   Min.   : 0.274
##  Class :character   1st Qu.:0.8835   1st Qu.:  2.401   1st Qu.: 0.759
##  Mode  :character   Median :1.4990   Median : 14.763   Median : 1.480
##                     Mean   :1.7103   Mean   : 27.700   Mean   : 4.060
##                     3rd Qu.:2.1225   3rd Qu.: 39.110   3rd Qu.: 3.789
##                     Max.   :6.2600   Max.   :195.777   Max.   :43.923
##                     NA's   :3        NA's   :4         NA's   :5
##       Ifnb              IL1b              IL4                IL5
##  Min.   : 0.1610   Min.   :  0.218   Min.   :  0.056   Min.   :0.0030
##  1st Qu.: 0.5493   1st Qu.:  1.548   1st Qu.:  2.369   1st Qu.:0.3317
##  Median : 1.1700   Median :  3.004   Median : 10.694   Median :0.4795
##  Mean   : 2.2359   Mean   : 15.473   Mean   : 32.073   Mean   :0.8084
##  3rd Qu.: 3.1892   3rd Qu.: 11.158   3rd Qu.: 51.919   3rd Qu.:0.7930
##  Max.   :12.3150   Max.   :139.989   Max.   :153.116   Max.   :7.2200
##  NA's   :6         NA's   :2         NA's   :7         NA's   :4
##       IL6              IL10             IL13             IL18
##  Min.   :0.1720   Min.   : 0.138   Min.   :0.1140   Min.   : 0.1370
##  1st Qu.:0.6435   1st Qu.: 1.338   1st Qu.:0.3985   1st Qu.: 0.8195
##  Median :1.0120   Median : 3.429   Median :0.6065   Median : 1.6100
##  Mean   :1.4347   Mean   : 8.296   Mean   :0.7302   Mean   : 1.8583
##  3rd Qu.:1.7550   3rd Qu.: 6.981   3rd Qu.:1.0272   3rd Qu.: 2.2080
##  Max.   :7.1360   Max.   :98.148   Max.   :1.7500   Max.   :11.8230
##  NA's   :4        NA's   :3        NA's   :6        NA's   :3
##      Isg15            Muc2            Tgfb1             Tnf
##  Min.   :0.266   Min.   : 0.209   Min.   :0.2990   Min.   :0.239
##  1st Qu.:1.024   1st Qu.: 1.298   1st Qu.:0.9908   1st Qu.:1.231
##  Median :2.007   Median : 5.703   Median :1.4970   Median :2.032
##  Mean   :2.410   Mean   : 8.473   Mean   :1.6154   Mean   :2.739
##  3rd Qu.:3.401   3rd Qu.:13.682   3rd Qu.:1.9877   3rd Qu.:3.872
##  Max.   :9.460   Max.   :27.762   Max.   :4.4300   Max.   :9.167
##  NA's   :3       NA's   :3                         NA's   :5
##       IL17
##  Min.   : 0.0100
##  1st Qu.: 0.2615
##  Median : 0.7080
##  Mean   : 4.5832
##  3rd Qu.: 1.6053
##  Max.   :44.6600
##  NA's   :22
```

```
# prep data
ileum_dat$Timepoint <- factor(ileum_dat$Timepoint, levels = c("d0", "d7", "d21", "d35"))
ileum_dat$Treatment <- factor(ileum_dat$Treatment, levels = c("Control", "PNS"))
ileum_dat$Litter <- factor(ileum_dat$Litter, levels = c("F5", "O4", "R4", "R5", "K6", "B4", "F4", "L4", "K5", "P5", "M6"))
ileum_dat$Sex <- factor(ileum_dat$Sex, levels = c("F", "M"))
```

Ileum Tissue *Ifng* Expression

```
# Fit linear mixed-effects model
# Include random intercept for litter
Ifng_model <- lmer(Ifng ~ Timepoint * Treatment + (1 | Litter), data = ileum_dat)
summary(Ifng_model)
```

```
## Linear mixed model fit by REML. t-tests use Satterthwaite's method [
## lmerModLmerTest]
## Formula: Ifng ~ Timepoint * Treatment + (1 | Litter)
##    Data: ileum_dat
##
## REML criterion at convergence: 443.4
##
## Scaled residuals:
##     Min      1Q  Median      3Q     Max
## -2.3746 -0.2611 -0.0245  0.1874  5.0216
##
## Random effects:
##  Groups   Name        Variance Std.Dev.
##  Litter   (Intercept)  9.622   3.102
##  Residual             23.514   4.849
## Number of obs: 77, groups:  Litter, 11
##
## Fixed effects:
##                             Estimate Std. Error      df t value Pr(>|t|)
## (Intercept)                   1.1968     2.0801 21.6240   0.575    0.571
## Timepointd7                   0.2117     2.5594 61.7713   0.083    0.934
## Timepointd21                  0.9643     2.1353 60.3255   0.452    0.653
## Timepointd35                  0.1555     2.3480 61.8203   0.066    0.947
## TreatmentPNS                  1.5200     2.8513 22.4997   0.533    0.599
## Timepointd7:TreatmentPNS      2.8501     3.3379 61.2665   0.854    0.397
## Timepointd21:TreatmentPNS     0.6613     2.9422 60.8836   0.225    0.823
## Timepointd35:TreatmentPNS    14.7343     3.2888 61.8032   4.480 3.29e-05 ***
## ---
## Signif. codes:  0 '***' 0.001 '**' 0.01 '*' 0.05 '.' 0.1 ' ' 1
##
## Correlation of Fixed Effects:
##             (Intr) Tmpnt7 Tmpn21 Tmpn35 TrtPNS T7:TPN T21:TP
## Timepointd7 -0.451
## Timepontd21 -0.541  0.428
## Timepontd35 -0.493  0.428  0.468
## TreatmntPNS -0.730  0.329  0.394  0.359
## Tmpnt7:TPNS  0.346 -0.767 -0.328 -0.328 -0.484
## Tmpn21:TPNS  0.392 -0.311 -0.726 -0.339 -0.551  0.463
## Tmpn35:TPNS  0.352 -0.305 -0.334 -0.714 -0.486  0.433  0.469
```

```
# Create a type III ANOVA table
Ifng_anova_table <- anova(Ifng_model, ddf = "Kenward-Roger")
print(Ifng_anova_table)
```

```
## Type III Analysis of Variance Table with Kenward-Roger's method
##                     Sum Sq Mean Sq NumDF  DenDF F value    Pr(>F)
## Timepoint           545.84  181.95     3 61.914  7.7378 0.0001808 ***
## Treatment           177.71  177.71     1  9.020  7.5579 0.0224633 *
## Timepoint:Treatment 578.78  192.93     3 61.914  8.2047 0.0001112 ***
## ---
## Signif. codes:  0 '***' 0.001 '**' 0.01 '*' 0.05 '.' 0.1 ' ' 1
```

```
# Because we have an interaction
Ifng_emcatcat <- emmeans(Ifng_model, ~ Timepoint * Treatment)

#Is there a difference between treatment groups at each time point?
contrast(Ifng_emcatcat, "revpairwise", by="Timepoint", adjust="BH")
```

```
## Timepoint = d0:
##  contrast      estimate   SE   df t.ratio p.value
##  PNS - Control     1.52 2.86 23.3   0.532  0.5995
##
## Timepoint = d7:
##  contrast      estimate   SE   df t.ratio p.value
##  PNS - Control     4.37 3.18 32.0   1.374  0.1791
##
## Timepoint = d21:
##  contrast      estimate   SE   df t.ratio p.value
##  PNS - Control     2.18 2.75 20.6   0.793  0.4366
##
## Timepoint = d35:
##  contrast      estimate   SE   df t.ratio p.value
##  PNS - Control    16.25 3.15 30.2   5.163  <.0001
##
## Degrees-of-freedom method: kenward-roger
```

```
#Is there a difference between time points within each treatment?
contrast(Ifng_emcatcat, "revpairwise", by="Treatment", adjust="BH")
```

```
## Treatment = Control:
##  contrast  estimate   SE   df t.ratio p.value
##  d7 - d0     0.2117 2.57 62.1   0.082  0.9831
##  d21 - d0    0.9643 2.14 60.7   0.451  0.9831
##  d21 - d7    0.7526 2.55 63.0   0.295  0.9831
##  d35 - d0    0.1555 2.36 62.2   0.066  0.9831
##  d35 - d7   -0.0561 2.63 60.4  -0.021  0.9831
##  d35 - d21  -0.8088 2.33 63.0  -0.346  0.9831
##
## Treatment = PNS:
##  contrast  estimate   SE   df t.ratio p.value
##  d7 - d0     3.0617 2.15 60.9   1.426  0.2384
##  d21 - d0    1.6256 2.03 61.9   0.800  0.4917
##  d21 - d7   -1.4361 2.08 61.5  -0.692  0.4917
##  d35 - d0   14.8898 2.31 62.1   6.438  <.0001
##  d35 - d7   11.8281 2.36 62.2   5.008  <.0001
##  d35 - d21  13.2642 2.25 62.7   5.889  <.0001
##
## Degrees-of-freedom method: kenward-roger
## P value adjustment: BH method for 6 tests
```

Ileum Tissue *Ifnb* Expression

```
# Fit linear mixed-effects model
# Include random intercept for litter
Ifnb_model <- lmer(Ifnb ~ Timepoint * Treatment + (1 | Litter), data = ileum_dat)
summary(Ifnb_model)
```

```
## Linear mixed model fit by REML. t-tests use Satterthwaite's method [
## lmerModLmerTest]
## Formula: Ifnb ~ Timepoint * Treatment + (1 | Litter)
##    Data: ileum_dat
##
## REML criterion at convergence: 307.7
##
## Scaled residuals:
##     Min      1Q  Median      3Q     Max
## -1.7936 -0.5903 -0.0816  0.3946  4.1769
##
## Random effects:
##  Groups   Name        Variance Std.Dev.
##  Litter   (Intercept) 2.997    1.731
##  Residual             3.213    1.792
## Number of obs: 76, groups:  Litter, 11
##
## Fixed effects:
##                            Estimate Std. Error       df t value Pr(>|t|)
## (Intercept)                 1.36751    0.98618 15.74524   1.387  0.18486
## Timepointd7                 0.02280    0.96031 59.08655   0.024  0.98114
## Timepointd21                0.41422    0.82452 59.57273   0.502  0.61725
## Timepointd35               -0.15154    0.88289 59.05921  -0.172  0.86431
## TreatmentPNS                0.01264    1.33195 15.61758   0.009  0.99255
## Timepointd7:TreatmentPNS    3.04445    1.24570 58.97841   2.444  0.01754 *
## Timepointd21:TreatmentPNS  1.06909    1.11536 59.49787   0.959  0.34168
## Timepointd35:TreatmentPNS  3.90968    1.22910 59.30517   3.181  0.00234 **
## ---
## Signif. codes:  0 '***' 0.001 '**' 0.01 '*' 0.05 '.' 0.1 ' ' 1
##
## Correlation of Fixed Effects:
##             (Intr) Tmpnt7 Tmpn21 Tmpn35 TrtPNS T7:TPN T21:TP
## Timepointd7 -0.378
## Timepontd21 -0.465  0.444
## Timepontd35 -0.413  0.445  0.484
## TreatmntPNS -0.740  0.280  0.344  0.305
## Tmpnt7:TPNS  0.292 -0.771 -0.342 -0.343 -0.398
## Tmpn21:TPNS  0.344 -0.328 -0.739 -0.358 -0.460  0.471
## Tmpn35:TPNS  0.296 -0.319 -0.348 -0.718 -0.399  0.442  0.477
```

```
# Create a type III ANOVA table
Ifnb_anova_table <- anova(Ifnb_model, ddf = "Kenward-Roger")
print(Ifnb_anova_table)
```

```
## Type III Analysis of Variance Table with Kenward-Roger's method
##                     Sum Sq Mean Sq NumDF  DenDF F value   Pr(>F)
## Timepoint           33.176  11.059     3 60.161  3.4418 0.022221 *
## Treatment           10.118  10.118     1  9.036  3.1491 0.109577
## Timepoint:Treatment 40.321  13.440     3 60.161  4.1830 0.009349 **
## ---
## Signif. codes:  0 '***' 0.001 '**' 0.01 '*' 0.05 '.' 0.1 ' ' 1
```

```
# Because we have an interaction
Ifnb_emcatcat <- emmeans(Ifnb_model, ~ Timepoint * Treatment)

#Is there a difference between treatment groups at each time point?
contrast(Ifnb_emcatcat, "revpairwise", by="Timepoint", adjust="BH")
```

```
## Timepoint = d0:
##  contrast      estimate   SE   df t.ratio p.value
##  PNS - Control   0.0126 1.33 16.6   0.009  0.9925
##
## Timepoint = d7:
##  contrast      estimate   SE   df t.ratio p.value
##  PNS - Control   3.0571 1.42 20.6   2.156  0.0431
##
## Timepoint = d21:
##  contrast      estimate   SE   df t.ratio p.value
##  PNS - Control   1.0817 1.29 14.6   0.842  0.4136
##
## Timepoint = d35:
##  contrast      estimate   SE   df t.ratio p.value
##  PNS - Control   3.9223 1.41 19.9   2.784  0.0115
##
## Degrees-of-freedom method: kenward-roger
```

```
#Is there a difference between time points within each treatment?
contrast(Ifnb_emcatcat, "revpairwise", by="Treatment", adjust="BH")
```

```
## Treatment = Control:
##  contrast  estimate    SE   df t.ratio p.value
##  d7 - d0     0.0228 0.962 59.7   0.024  0.9812
##  d21 - d0    0.4142 0.826 60.2   0.501  0.9812
##  d21 - d7    0.3914 0.952 61.2   0.411  0.9812
##  d35 - d0   -0.1515 0.884 59.7  -0.171  0.9812
##  d35 - d7   -0.1743 0.974 59.2  -0.179  0.9812
##  d35 - d21  -0.5658 0.873 61.4  -0.648  0.9812
##
## Treatment = PNS:
##  contrast  estimate    SE   df t.ratio p.value
##  d7 - d0     3.0672 0.794 59.5   3.862  0.0008
##  d21 - d0    1.4833 0.753 60.0   1.971  0.0641
##  d21 - d7   -1.5839 0.769 59.9  -2.060  0.0641
##  d35 - d0    3.7581 0.857 60.2   4.384  0.0003
##  d35 - d7    0.6909 0.875 60.2   0.789  0.4331
##  d35 - d21   2.2748 0.835 60.5   2.723  0.0169
##
## Degrees-of-freedom method: kenward-roger
## P value adjustment: BH method for 6 tests
```

Ileum Tissue *Isg15* Expression

```
# Fit linear mixed-effects model
# Include random intercept for Litter
Isg15_model <- lmer(Isg15 ~ Timepoint * Treatment + (1 | Litter), data = ileum_dat)
summary(Isg15_model)
```

```
## Linear mixed model fit by REML. t-tests use Satterthwaite's method [
## lmerModLmerTest]
## Formula: Isg15 ~ Timepoint * Treatment + (1 | Litter)
##    Data: ileum_dat
##
## REML criterion at convergence: 268.5
##
## Scaled residuals:
##     Min      1Q  Median      3Q     Max
## -1.9942 -0.5539 -0.0586  0.5259  3.2337
##
## Random effects:
##  Groups   Name        Variance Std.Dev.
##  Litter   (Intercept) 0.536    0.7321
##  Residual             1.722    1.3122
## Number of obs: 79, groups:  Litter, 11
##
## Fixed effects:
##                           Estimate Std. Error      df t value Pr(>|t|)
## (Intercept)                 1.2011     0.5317 25.1834   2.259 0.032797 *
## Timepointd7                 1.3827     0.6560 63.7318   2.108 0.038993 *
## Timepointd21                1.1394     0.5932 63.0370   1.921 0.059292 .
## Timepointd35               -0.3367     0.6342 64.2635  -0.531 0.597317
## TreatmentPNS                0.2857     0.7297 26.2594   0.392 0.698585
## Timepointd7:TreatmentPNS   -0.4885     0.8669 63.4429  -0.564 0.575049
## Timepointd21:TreatmentPNS   1.1108     0.8072 63.3129   1.376 0.173643
## Timepointd35:TreatmentPNS   3.0258     0.8772 64.5557   3.449 0.000995 ***
## ---
## Signif. codes:  0 '***' 0.001 '**' 0.01 '*' 0.05 '.' 0.1 ' ' 1
##
## Correlation of Fixed Effects:
##             (Intr) Tmpnt7 Tmpn21 Tmpn35 TrtPNS T7:TPN T21:TP
## Timepointd7 -0.496
## Timepontd21 -0.557  0.429
## Timepontd35 -0.521  0.443  0.452
## TreatmntPNS -0.729  0.362  0.406  0.380
## Tmpnt7:TPNS  0.376 -0.757 -0.325 -0.335 -0.530
## Tmpn21:TPNS  0.409 -0.315 -0.735 -0.332 -0.574  0.475
## Tmpn35:TPNS  0.377 -0.320 -0.327 -0.723 -0.524  0.451  0.463
```

```
# Create a type III ANOVA table
Isg15_anova_table <- anova(Isg15_model, ddf = "Kenward-Roger")
print(Isg15_anova_table)
```

```
## Type III Analysis of Variance Table with Kenward-Roger's method
##                     Sum Sq Mean Sq NumDF  DenDF F value   Pr(>F)    
## Timepoint           31.798 10.5994     3 64.547  6.1558 0.000955 ***
## Treatment            8.515  8.5148     1  8.898  4.9453 0.053562 .  
## Timepoint:Treatment 30.505 10.1682     3 64.547  5.9054 0.001262 ** 
## ---
## Signif. codes:  0 '***' 0.001 '**' 0.01 '*' 0.05 '.' 0.1 ' ' 1
```

```
# Because we have an interaction
Isg15_emcatcat <- emmeans(Isg15_model, ~ Timepoint * Treatment)

#Is there a difference between treatment groups at each time point?
contrast(Isg15_emcatcat, "revpairwise", by="Timepoint", adjust="BH")
```

```
## Timepoint = d0:
##  contrast      estimate    SE   df t.ratio p.value
##  PNS - Control    0.286 0.731 27.1   0.391  0.6989
## 
## Timepoint = d7:
##  contrast      estimate    SE   df t.ratio p.value
##  PNS - Control   -0.203 0.786 32.8  -0.258  0.7979
## 
## Timepoint = d21:
##  contrast      estimate    SE   df t.ratio p.value
##  PNS - Control    1.396 0.714 25.1   1.956  0.0617
## 
## Timepoint = d35:
##  contrast      estimate    SE   df t.ratio p.value
##  PNS - Control    3.311 0.799 33.0   4.145  0.0002
## 
## Degrees-of-freedom method: kenward-roger
```

```
#Is there a difference between time points within each treatment?
contrast(Isg15_emcatcat, "revpairwise", by="Treatment", adjust="BH")
```

```
## Treatment = Control:
##  contrast  estimate    SE   df t.ratio p.value
##  d7 - d0      1.383 0.658 64.1   2.100  0.0794
##  d21 - d0     1.139 0.595 63.4   1.916  0.0899
##  d21 - d7    -0.243 0.676 66.5  -0.360  0.7198
##  d35 - d0    -0.337 0.637 64.6  -0.528  0.7189
##  d35 - d7    -1.719 0.682 62.4  -2.522  0.0784
##  d35 - d21   -1.476 0.649 66.4  -2.275  0.0784
## 
## Treatment = PNS:
##  contrast  estimate    SE   df t.ratio p.value
##  d7 - d0      0.894 0.568 63.4   1.573  0.1448
##  d21 - d0     2.250 0.550 64.0   4.093  0.0004
##  d21 - d7     1.356 0.539 62.3   2.516  0.0217
##  d35 - d0     2.689 0.610 65.2   4.408  0.0002
##  d35 - d7     1.795 0.614 65.7   2.922  0.0095
##  d35 - d21    0.439 0.598 66.4   0.734  0.4658
## 
## Degrees-of-freedom method: kenward-roger 
## P value adjustment: BH method for 6 tests
```

Ileum Tissue *Il1b* Expression

```
# Fit linear mixed-effects model
# Include random intercept for litter
IL1b_model <- lmer(IL1b ~ Timepoint * Treatment + (1 | Litter), data = ileum_dat)
```

```
## boundary (singular) fit: see help('isSingular')
```

```
summary(IL1b_model)
```

```
## Linear mixed model fit by REML. t-tests use Satterthwaite's method [
## lmerModLmerTest]
## Formula: IL1b ~ Timepoint * Treatment + (1 | Litter)
##    Data: ileum_dat
##
## REML criterion at convergence: 675.5
##
## Scaled residuals:
##     Min      1Q  Median      3Q     Max
## -2.3962 -0.0819 -0.0057  0.0723  3.9264
##
## Random effects:
##  Groups   Name        Variance Std.Dev.
##  Litter   (Intercept)   0.0     0.00
##  Residual             539.2    23.22
## Number of obs: 80, groups:  Litter, 11
##
## Fixed effects:
##                            Estimate Std. Error      df t value Pr(>|t|)
## (Intercept)                  1.1128     7.7405 72.0000   0.144    0.886
## Timepointd7                 56.9792    11.7026 72.0000   4.869 6.43e-06 ***
## Timepointd21                 1.5499    10.4373 72.0000   0.149    0.882
## Timepointd35                 0.8073    11.2837 72.0000   0.072    0.943
## TreatmentPNS                 0.3316    10.2398 72.0000   0.032    0.974
## Timepointd7:TreatmentPNS   -9.6110    15.1957 72.0000  -0.632    0.529
## Timepointd21:TreatmentPNS   5.5213    13.9769 72.0000   0.395    0.694
## Timepointd35:TreatmentPNS  10.1464    15.2373 72.0000   0.666    0.508
## ---
## Signif. codes:  0 '***' 0.001 '**' 0.01 '*' 0.05 '.' 0.1 ' ' 1
##
## Correlation of Fixed Effects:
##             (Intr) Tmpnt7 Tmpn21 Tmpn35 TrtPNS T7:TPN T21:TP
## Timepointd7 -0.661
## Timepontd21 -0.742  0.491
## Timepontd35 -0.686  0.454  0.509
## TreatmntPNS -0.756  0.500  0.561  0.519
## Tmpnt7:TPNS  0.509 -0.770 -0.378 -0.349 -0.674
## Tmpn21:TPNS  0.554 -0.366 -0.747 -0.380 -0.733  0.494
## Tmpn35:TPNS  0.508 -0.336 -0.377 -0.741 -0.672  0.453  0.492
## optimizer (nloptwrap) convergence code: 0 (OK)
## boundary (singular) fit: see help('isSingular')
```

```
# Create a type III ANOVA table
IL1b_anova_table <- anova(IL1b_model, ddf = "Kenward-Roger")
print(IL1b_anova_table)
```

```
## Type III Analysis of Variance Table with Kenward-Roger's method
##                     Sum Sq Mean Sq NumDF  DenDF F value    Pr(>F)
## Timepoint            31957 10652.3     3 67.808 19.7524 2.609e-09 ***
## Treatment               64    64.1     1  8.248  0.1189    0.7388
## Timepoint:Treatment    937   312.2     3 67.808  0.5789    0.6309
## ---
## Signif. codes:  0 '***' 0.001 '**' 0.01 '*' 0.05 '.' 0.1 ' ' 1
```

```
#Because no interaction, test for main effects of treatment at individual time points
IL1b_emcatcat <- emmeans(IL1b_model, ~ Treatment | Timepoint)
#Is there a difference between treatment groups at each time point?
contrast(IL1b_emcatcat, "revpairwise", adjust="BH")
```

```
## Timepoint = d0:
##  contrast      estimate    SE   df t.ratio p.value
##  PNS - Control    0.332 10.30 56.2   0.032  0.9745
##
## Timepoint = d7:
##  contrast      estimate    SE   df t.ratio p.value
##  PNS - Control   -9.279 11.30 61.1  -0.820  0.4152
##
## Timepoint = d21:
##  contrast      estimate    SE   df t.ratio p.value
##  PNS - Control    5.853  9.53 53.9   0.614  0.5417
##
## Timepoint = d35:
##  contrast      estimate    SE   df t.ratio p.value
##  PNS - Control   10.478 11.50 55.8   0.914  0.3648
##
## Degrees-of-freedom method: kenward-roger
```

Ileum Tissue *Il6* Expression

```
# Fit linear mixed-effects model
# Include random intercept for litter
IL6_model <- lmer(IL6 ~ Timepoint * Treatment + (1 | Litter), data = ileum_dat)
summary(IL6_model)
```

```
## Linear mixed model fit by REML. t-tests use Satterthwaite's method [
## lmerModLmerTest]
## Formula: IL6 ~ Timepoint * Treatment + (1 | Litter)
##    Data: ileum_dat
##
## REML criterion at convergence: 221.2
##
## Scaled residuals:
##     Min      1Q  Median      3Q     Max
## -2.1445 -0.4711 -0.0736  0.3694  3.7483
##
## Random effects:
##  Groups   Name        Variance Std.Dev.
##  Litter   (Intercept) 0.1110   0.3331
##  Residual             0.9928   0.9964
## Number of obs: 78, groups:  Litter, 11
##
## Fixed effects:
##                             Estimate Std. Error       df t value Pr(>|t|)
## (Intercept)                  1.13448    0.35033 37.71374   3.238 0.002510 **
## Timepointd7                 -0.32275    0.52085 64.77649  -0.620 0.537660
## Timepointd21                -0.23197    0.43731 62.32300  -0.530 0.597690
## Timepointd35                -0.06825    0.47787 64.61760  -0.143 0.886876
## TreatmentPNS                -0.37636    0.48265 39.15057  -0.780 0.440204
## Timepointd7:TreatmentPNS     1.18961    0.67444 64.24955   1.764 0.082509 .
## Timepointd21:TreatmentPNS    1.51948    0.60105 63.17740   2.528 0.013983 *
## Timepointd35:TreatmentPNS    2.50049    0.66926 64.67776   3.736 0.000397 ***
## ---
## Signif. codes:  0 '***' 0.001 '**' 0.01 '*' 0.05 '.' 0.1 ' ' 1
##
## Correlation of Fixed Effects:
##             (Intr) Tmpnt7 Tmpn21 Tmpn35 TrtPNS T7:TPN T21:TP
## Timepointd7 -0.551
## Timepontd21 -0.656  0.435
## Timepontd35 -0.601  0.419  0.475
## TreatmntPNS -0.726  0.400  0.476  0.436
## Tmpnt7:TPNS  0.425 -0.772 -0.336 -0.323 -0.592
## Tmpn21:TPNS  0.477 -0.317 -0.728 -0.346 -0.664  0.474
## Tmpn35:TPNS  0.429 -0.299 -0.339 -0.714 -0.592  0.432  0.475
```

```
# Create a type III ANOVA table
IL6_anova_table <- anova(IL6_model, ddf = "Kenward-Roger")
print(IL6_anova_table)
```

```
## Type III Analysis of Variance Table with Kenward-Roger's method
##                     Sum Sq Mean Sq NumDF  DenDF F value   Pr(>F)
## Timepoint          13.0031  4.3344     3 64.499  4.3658 0.007334 **
## Treatment           8.8111  8.8111     1  8.924  8.8753 0.015615 *
## Timepoint:Treatment 14.4352  4.8117     3 64.499  4.8467 0.004201 **
## ---
## Signif. codes:  0 '***' 0.001 '**' 0.01 '*' 0.05 '.' 0.1 ' ' 1
```

```
# Because we have an interaction
IL6_emcatcat <- emmeans(IL6_model, ~ Timepoint * Treatment)

#Is there a difference between treatment groups at each time point?
contrast(IL6_emcatcat, "revpairwise", by="Timepoint", adjust="BH")
```

```
## Timepoint = d0:
##  contrast      estimate    SE   df t.ratio p.value
##  PNS - Control   -0.376 0.485 39.7  -0.777  0.4419
##
## Timepoint = d7:
##  contrast      estimate    SE   df t.ratio p.value
##  PNS - Control    0.813 0.553 50.1   1.471  0.1476
##
## Timepoint = d21:
##  contrast      estimate    SE   df t.ratio p.value
##  PNS - Control    1.143 0.458 35.0   2.495  0.0175
##
## Timepoint = d35:
##  contrast      estimate    SE   df t.ratio p.value
##  PNS - Control    2.124 0.551 47.0   3.852  0.0004
##
## Degrees-of-freedom method: kenward-roger
```

```
#Is there a difference between time points within each treatment?
contrast(IL6_emcatcat, "revpairwise", by="Treatment", adjust="BH")
```

```
## Treatment = Control:
##  contrast  estimate    SE   df t.ratio p.value
##  d7 - d0    -0.3227 0.525 64.9  -0.615  0.8876
##  d21 - d0   -0.2320 0.439 62.5  -0.529  0.8876
##  d21 - d7    0.0908 0.519 66.2   0.175  0.8876
##  d35 - d0   -0.0683 0.481 64.8  -0.142  0.8876
##  d35 - d7    0.2545 0.541 62.0   0.471  0.8876
##  d35 - d21   0.1637 0.475 66.1   0.345  0.8876
##
## Treatment = PNS:
##  contrast  estimate    SE   df t.ratio p.value
##  d7 - d0     0.8669 0.431 63.6   2.012  0.0582
##  d21 - d0    1.2875 0.415 64.3   3.102  0.0057
##  d21 - d7    0.4206 0.411 62.5   1.023  0.3101
##  d35 - d0    2.4322 0.472 64.9   5.152  <.0001
##  d35 - d7    1.5654 0.476 66.0   3.292  0.0048
##  d35 - d21   1.1447 0.458 65.5   2.497  0.0226
##
## Degrees-of-freedom method: kenward-roger
## P value adjustment: BH method for 6 tests
```

Ileum Tissue *Tnfa* Expression

```
# Fit linear mixed-effects model
# Include random intercept for litter
Tnf_model <- lmer(Tnf ~ Timepoint * Treatment + (1 | Litter), data = ileum_dat)
summary(Tnf_model)
```

```
## Linear mixed model fit by REML. t-tests use Satterthwaite's method [
## lmerModLmerTest]
## Formula: Tnf ~ Timepoint * Treatment + (1 | Litter)
##    Data: ileum_dat
##
## REML criterion at convergence: 260.3
##
## Scaled residuals:
##     Min      1Q  Median      3Q     Max
## -2.2445 -0.4638 -0.0692  0.5228  3.2827
##
## Random effects:
##  Groups   Name        Variance Std.Dev.
##  Litter   (Intercept) 0.582    0.7629
##  Residual             1.683    1.2973
## Number of obs: 77, groups:  Litter, 11
##
## Fixed effects:
##                             Estimate Std. Error      df t value Pr(>|t|)
## (Intercept)                   1.0081     0.5562 27.1441   1.812  0.08100 .
## Timepointd7                   0.4586     0.7039 63.2929   0.652  0.51706
## Timepointd21                  2.6588     0.5884 61.0256   4.518 2.92e-05 ***
## Timepointd35                  1.3120     0.6463 63.0519   2.030  0.04659 *
## TreatmentPNS                  0.2246     0.7508 26.8915   0.299  0.76713
## Timepointd7:TreatmentPNS      1.7728     0.9002 62.6602   1.969  0.05333 .
## Timepointd21:TreatmentPNS    -0.3862     0.7953 60.9100  -0.486  0.62901
## Timepointd35:TreatmentPNS     2.4549     0.8917 62.6177   2.753  0.00772 **
## ---
## Signif. codes:  0 '***' 0.001 '**' 0.01 '*' 0.05 '.' 0.1 ' ' 1
##
## Correlation of Fixed Effects:
##             (Intr) Tmpnt7 Tmpn21 Tmpn35 TrtPNS T7:TPN T21:TP
## Timepointd7 -0.501
## Timepontd21 -0.588  0.462
## Timepontd35 -0.541  0.459  0.499
## TreatmntPNS -0.741  0.371  0.435  0.401
## Tmpnt7:TPNS  0.391 -0.782 -0.361 -0.359 -0.525
## Tmpn21:TPNS  0.435 -0.342 -0.740 -0.370 -0.582  0.487
## Tmpn35:TPNS  0.392 -0.333 -0.362 -0.725 -0.520  0.452  0.485
```

```
# Create a type III ANOVA table
Tnf_anova_table <- anova(Tnf_model, ddf = "Kenward-Roger")
print(Tnf_anova_table)
```

```
## Type III Analysis of Variance Table with Kenward-Roger's method
##                     Sum Sq Mean Sq NumDF  DenDF F value    Pr(>F)
## Timepoint           81.747 27.2489     3 62.136 16.1912 6.921e-08 ***
## Treatment            7.600  7.5998     1  9.002  4.5159  0.062515 .
## Timepoint:Treatment 24.123  8.0411     3 62.136  4.7780  0.004639 **
## ---
## Signif. codes:  0 '***' 0.001 '**' 0.01 '*' 0.05 '.' 0.1 ' ' 1
```

```
# Because we have an interaction
Tnf_emcatcat <- emmeans(Tnf_model, ~ Timepoint * Treatment)

#Is there a difference between treatment groups at each time point?
contrast(Tnf_emcatcat, "revpairwise", by="Timepoint", adjust="BH")
```

```
## Timepoint = d0:
##  contrast      estimate    SE   df t.ratio p.value
##  PNS - Control    0.225 0.752 26.7   0.299  0.7676
##
## Timepoint = d7:
##  contrast      estimate    SE   df t.ratio p.value
##  PNS - Control    1.997 0.817 33.5   2.444  0.0200
##
## Timepoint = d21:
##  contrast      estimate    SE   df t.ratio p.value
##  PNS - Control   -0.162 0.708 22.2  -0.228  0.8217
##
## Timepoint = d35:
##  contrast      estimate    SE   df t.ratio p.value
##  PNS - Control    2.680 0.818 32.5   3.276  0.0025
##
## Degrees-of-freedom method: kenward-roger
```

```
#Is there a difference between time points within each treatment?
contrast(Tnf_emcatcat, "revpairwise", by="Treatment", adjust="BH")
```

```
## Treatment = Control:
##  contrast  estimate    SE   df t.ratio p.value
##  d7 - d0     0.4586 0.709 63.2   0.647  0.5199
##  d21 - d0    2.6588 0.590 61.0   4.510  0.0002
##  d21 - d7    2.2002 0.682 63.4   3.225  0.0060
##  d35 - d0    1.3120 0.650 63.0   2.018  0.0718
##  d35 - d7    0.8533 0.705 60.5   1.211  0.2768
##  d35 - d21  -1.3469 0.624 63.4  -2.159  0.0693
##
## Treatment = PNS:
##  contrast  estimate    SE   df t.ratio p.value
##  d7 - d0     2.2315 0.563 61.5   3.964  0.0004
##  d21 - d0    2.2727 0.536 60.7   4.241  0.0002
##  d21 - d7    0.0412 0.537 61.0   0.077  0.9391
##  d35 - d0    3.7669 0.617 62.1   6.105  <.0001
##  d35 - d7    1.5354 0.624 63.2   2.460  0.0200
##  d35 - d21   1.4943 0.599 62.4   2.493  0.0200
##
## Degrees-of-freedom method: kenward-roger
## P value adjustment: BH method for 6 tests
```

Ileum Tissue *Ahr* Expression

```
# Fit linear mixed-effects model
# Include random intercept for litter
Ahr_model <- lmer(Ahr ~ Timepoint * Treatment + (1 | Litter), data = ileum_dat)
summary(Ahr_model)
```

```
## Linear mixed model fit by REML. t-tests use Satterthwaite's method [
## lmerModLmerTest]
## Formula: Ahr ~ Timepoint * Treatment + (1 | Litter)
##    Data: ileum_dat
##
## REML criterion at convergence: 183.7
##
## Scaled residuals:
##     Min      1Q  Median      3Q     Max
## -2.2947 -0.4825 -0.1014  0.4302  3.1179
##
## Random effects:
##  Groups   Name        Variance Std.Dev.
##  Litter   (Intercept) 0.09218  0.3036
##  Residual             0.54698  0.7396
## Number of obs: 79, groups:  Litter, 11
##
## Fixed effects:
##                           Estimate Std. Error       df t value Pr(>|t|)
## (Intercept)                1.06098    0.28421 37.43814   3.733 0.000627 ***
## Timepointd7                2.42395    0.37430 63.38026   6.476 1.59e-08 ***
## Timepointd21               0.04389    0.33663 65.22377   0.130 0.896676
## Timepointd35              -0.44785    0.36204 64.05353  -1.237 0.220599
## TreatmentPNS               0.16444    0.38231 37.52137   0.430 0.669562
## Timepointd7:TreatmentPNS  -1.24656    0.49013 63.22737  -2.543 0.013439 *
## Timepointd21:TreatmentPNS  0.49046    0.45399 64.47564   1.080 0.284017
## Timepointd35:TreatmentPNS  1.58956    0.49669 65.45313   3.200 0.002118 **
## ---
## Signif. codes:  0 '***' 0.001 '**' 0.01 '*' 0.05 '.' 0.1 ' ' 1
##
## Correlation of Fixed Effects:
##             (Intr) Tmpnt7 Tmpn21 Tmpn35 TrtPNS T7:TPN T21:TP
## Timepointd7 -0.568
## Timepontd21 -0.656  0.475
## Timepontd35 -0.594  0.457  0.497
## TreatmntPNS -0.743  0.422  0.488  0.442
## Tmpnt7:TPNS  0.434 -0.764 -0.363 -0.349 -0.590
## Tmpn21:TPNS  0.487 -0.352 -0.741 -0.368 -0.650  0.494
## Tmpn35:TPNS  0.433 -0.333 -0.362 -0.729 -0.587  0.459  0.493
```

```
# Create a type III ANOVA table
Ahr_anova_table <- anova(Ahr_model, ddf = "Kenward-Roger")
print(Ahr_anova_table)
```

```
## Type III Analysis of Variance Table with Kenward-Roger's method
##                      Sum Sq Mean Sq NumDF  DenDF F value    Pr(>F)    
## Timepoint            34.371 11.4569     3 65.026 20.9450 1.313e-09 ***
## Treatment             1.183  1.1833     1  8.773  2.1634    0.1763    
## Timepoint:Treatment 17.167  5.7223     3 65.026 10.4613 1.051e-05 ***
## ---
## Signif. codes:  0 '***' 0.001 '**' 0.01 '*' 0.05 '.' 0.1 ' ' 1
```

```
# Because we have an interaction
Ahr_emcatcat <- emmeans(Ahr_model, ~ Timepoint * Treatment)

#Is there a difference between treatment groups at each time point?
contrast(Ahr_emcatcat, "revpairwise", by="Timepoint", adjust="BH")
```

```
## Timepoint = d0:
##  contrast      estimate    SE   df t.ratio p.value
##  PNS - Control    0.164 0.383 37.0   0.429  0.6704
## 
## Timepoint = d7:
##  contrast      estimate    SE   df t.ratio p.value
##  PNS - Control   -1.082 0.409 41.5  -2.648  0.0114
## 
## Timepoint = d21:
##  contrast      estimate    SE   df t.ratio p.value
##  PNS - Control    0.655 0.357 30.6   1.836  0.0761
## 
## Timepoint = d35:
##  contrast      estimate    SE   df t.ratio p.value
##  PNS - Control    1.754 0.416 40.5   4.215  0.0001
## 
## Degrees-of-freedom method: kenward-roger
```

```
#Is there a difference between time points within each treatment?
contrast(Ahr_emcatcat, "revpairwise", by="Treatment", adjust="BH")
```

```
## Treatment = Control:
##  contrast  estimate    SE   df t.ratio p.value
##  d7 - d0     2.4239 0.375 63.2   6.464  <.0001
##  d21 - d0    0.0439 0.338 65.1   0.130  0.8972
##  d21 - d7   -2.3801 0.369 67.3  -6.449  <.0001
##  d35 - d0   -0.4479 0.363 63.9  -1.233  0.2665
##  d35 - d7   -2.8718 0.384 62.6  -7.478  <.0001
##  d35 - d21  -0.4917 0.354 67.3  -1.388  0.2546
## 
## Treatment = PNS:
##  contrast  estimate    SE   df t.ratio p.value
##  d7 - d0     1.1774 0.317 62.8   3.715  0.0026
##  d21 - d0    0.5343 0.305 63.3   1.750  0.1019
##  d21 - d7   -0.6430 0.305 63.3  -2.106  0.0783
##  d35 - d0    1.1417 0.343 66.8   3.325  0.0043
##  d35 - d7   -0.0357 0.345 67.3  -0.103  0.9179
##  d35 - d21   0.6074 0.331 66.3   1.837  0.1019
## 
## Degrees-of-freedom method: kenward-roger 
## P value adjustment: BH method for 6 tests
```

Ileum Tissue *Muc2* Expression

```
##Muc2
# Fit linear mixed-effects model
Muc2_model <- lmer(Muc2 ~ Timepoint * Treatment + (1 | Litter), data = ileum_dat) # include random intercept for litter
summary(Muc2_model)
```

```
## Linear mixed model fit by REML. t-tests use Satterthwaite's method [
## lmerModLmerTest]
## Formula: Muc2 ~ Timepoint * Treatment + (1 | Litter)
##    Data: ileum_dat
##
## REML criterion at convergence: 413.1
##
## Scaled residuals:
##      Min       1Q   Median       3Q      Max
## -2.10247 -0.41354  0.01396  0.52086  2.63802
##
## Random effects:
##  Groups   Name        Variance Std.Dev.
##  Litter   (Intercept)  2.074   1.440
##  Residual             13.945   3.734
## Number of obs: 79, groups:  Litter, 11
##
## Fixed effects:
##                             Estimate Std. Error      df t value Pr(>|t|)
## (Intercept)                   1.2123     1.4053 39.6373   0.863  0.39352
## Timepointd7                  -0.3931     1.9020 64.8654  -0.207  0.83691
## Timepointd21                 11.2166     1.6825 62.6049   6.667 7.78e-09 ***
## Timepointd35                  8.4663     1.8382 65.4363   4.606 1.95e-05 ***
## TreatmentPNS                  0.3102     1.9006 39.0533   0.163  0.87119
## Timepointd7:TreatmentPNS    1.8617     2.4906 64.6026   0.747  0.45748
## Timepointd21:TreatmentPNS   4.5990     2.2661 63.2856   2.030  0.04662 *
## Timepointd35:TreatmentPNS   8.1533     2.5442 65.3389   3.205  0.00209 **
## ---
## Signif. codes:  0 '***' 0.001 '**' 0.01 '*' 0.05 '.' 0.1 ' ' 1
##
## Correlation of Fixed Effects:
##             (Intr) Tmpnt7 Tmpn21 Tmpn35 TrtPNS T7:TPN T21:TP
## Timepointd7 -0.582
## Timepontd21 -0.659  0.478
## Timepontd35 -0.607  0.464  0.499
## TreatmntPNS -0.739  0.430  0.487  0.449
## Tmpnt7:TPNS  0.444 -0.764 -0.365 -0.354 -0.603
## Tmpn21:TPNS  0.489 -0.355 -0.742 -0.370 -0.663  0.503
## Tmpn35:TPNS  0.438 -0.335 -0.360 -0.723 -0.588  0.458  0.488
```

```
# Create a type III ANOVA table
Muc2_anova_table <- anova(Muc2_model, ddf = "Kenward-Roger")
print(Muc2_anova_table)
```

```
## Type III Analysis of Variance Table with Kenward-Roger's method
##                     Sum Sq Mean Sq NumDF  DenDF F value  Pr(>F)
## Timepoint           3173.3 1057.78     3 64.922 75.8517 < 2e-16 ***
## Treatment            143.4  143.45     1  8.951 10.2869 0.01078 *
## Timepoint:Treatment  161.8   53.94     3 64.922  3.8677 0.01312 *
## ---
## Signif. codes:  0 '***' 0.001 '**' 0.01 '*' 0.05 '.' 0.1 ' ' 1
```

```
# Because we have an interaction
Muc2_emcatcat <- emmeans(Muc2_model, ~ Timepoint * Treatment)

#Is there a difference between treatment groups at each time point?
contrast(Muc2_emcatcat, "revpairwise", by="Timepoint", adjust="BH")
```

```
## Timepoint = d0:
##  contrast      estimate   SE   df t.ratio p.value
##  PNS - Control     0.31 1.91 39.3   0.163  0.8715
##
## Timepoint = d7:
##  contrast      estimate   SE   df t.ratio p.value
##  PNS - Control     2.17 2.04 43.8   1.067  0.2918
##
## Timepoint = d21:
##  contrast      estimate   SE   df t.ratio p.value
##  PNS - Control     4.91 1.75 31.4   2.813  0.0084
##
## Timepoint = d35:
##  contrast      estimate   SE   df t.ratio p.value
##  PNS - Control     8.46 2.12 45.0   4.001  0.0002
##
## Degrees-of-freedom method: kenward-roger
```

```
#Is there a difference between time points within each treatment?
contrast(Muc2_emcatcat, "revpairwise", by="Treatment", adjust="BH")
```

```
## Treatment = Control:
##  contrast  estimate   SE   df t.ratio p.value
##  d7 - d0     -0.393 1.91 64.9  -0.206  0.8377
##  d21 - d0    11.217 1.68 62.7   6.659  <.0001
##  d21 - d7    11.610 1.86 66.8   6.250  <.0001
##  d35 - d0     8.466 1.85 65.5   4.578  <.0001
##  d35 - d7     8.859 1.94 62.6   4.569  <.0001
##  d35 - d21   -2.750 1.78 66.8  -1.542  0.1532
##
## Treatment = PNS:
##  contrast  estimate   SE   df t.ratio p.value
##  d7 - d0      1.469 1.62 64.3   0.909  0.4401
##  d21 - d0    15.816 1.52 64.2  10.374  <.0001
##  d21 - d7    14.347 1.51 62.6   9.506  <.0001
##  d35 - d0    16.620 1.77 65.3   9.387  <.0001
##  d35 - d7    15.151 1.78 66.4   8.492  <.0001
##  d35 - d21    0.804 1.71 66.7   0.472  0.6388
##
## Degrees-of-freedom method: kenward-roger
## P value adjustment: BH method for 6 tests
```

Supplementary Gene Expression

Ileum Tissue *Il10* Expression

```
# Fit linear mixed-effects model
# Include random intercept for litter
IL10_model <- lmer(IL10 ~ Timepoint * Treatment + (1 | Litter), data = ileum_dat)
summary(IL10_model)
```

```
## Linear mixed model fit by REML. t-tests use Satterthwaite's method [
## lmerModLmerTest]
## Formula: IL10 ~ Timepoint * Treatment + (1 | Litter)
##    Data: ileum_dat
##
## REML criterion at convergence: 556.7
##
## Scaled residuals:
##     Min      1Q  Median      3Q     Max
## -3.3394 -0.2441 -0.0100  0.1126  4.9315
##
## Random effects:
##  Groups   Name        Variance Std.Dev.
##  Litter   (Intercept)   6.196   2.489
##  Residual             110.615  10.517
## Number of obs: 79, groups:  Litter, 11
##
## Fixed effects:
##                           Estimate Std. Error        df t value Pr(>|t|)
## (Intercept)                1.388157   3.519829 43.444888   0.394    0.695
## Timepointd7                1.983329   5.209413 64.771481   0.381    0.705
## Timepointd21               3.817428   4.608346 63.111247   0.828    0.411
## Timepointd35               8.139984   5.022526 65.789955   1.621    0.110
## TreatmentPNS              -0.463872   4.856009 44.905786  -0.096    0.924
## Timepointd7:TreatmentPNS  -0.008137   6.889481 64.684525  -0.001    0.999
## Timepointd21:TreatmentPNS  2.397660   6.317880 63.160750   0.380    0.706
## Timepointd35:TreatmentPNS 34.695969   7.043096 66.668662   4.926 5.82e-06 ***
## ---
## Signif. codes:  0 '***' 0.001 '**' 0.01 '*' 0.05 '.' 0.1 ' ' 1
##
## Correlation of Fixed Effects:
##             (Intr) Tmpnt7 Tmpn21 Tmpn35 TrtPNS T7:TPN T21:TP
## Timepointd7 -0.605
## Timepontd21 -0.687  0.458
## Timepontd35 -0.631  0.433  0.478
## TreatmntPNS -0.725  0.438  0.498  0.457
## Tmpnt7:TPNS  0.457 -0.756 -0.346 -0.327 -0.636
## Tmpn21:TPNS  0.501 -0.334 -0.729 -0.349 -0.694  0.487
## Tmpn35:TPNS  0.450 -0.309 -0.341 -0.713 -0.621  0.441  0.474
```

```
# Create a type III ANOVA table
IL10_anova_table <- anova(IL10_model, ddf = "Kenward-Roger")
print(IL10_anova_table)
```

```
## Type III Analysis of Variance Table with Kenward-Roger's method
##                     Sum Sq Mean Sq NumDF  DenDF F value    Pr(>F)
## Timepoint           6716.3  2238.8     3 66.139 20.2376 2.044e-09 ***
## Treatment           1035.3  1035.3     1  8.627  9.3591   0.01427 *
## Timepoint:Treatment 3479.3  1159.8     3 66.139 10.4839 9.928e-06 ***
## ---
## Signif. codes:  0 '***' 0.001 '**' 0.01 '*' 0.05 '.' 0.1 ' ' 1
```

```
# Because we have an interaction
IL10_emcatcat <- emmeans(IL10_model, ~ Timepoint * Treatment)

#Is there a difference between treatment groups at each time point?
contrast(IL10_emcatcat, "revpairwise", by="Timepoint", adjust="BH")
```

```
## Timepoint = d0:
##  contrast      estimate   SE   df t.ratio p.value
##  PNS - Control   -0.464 4.88 46.8  -0.095  0.9247
##
## Timepoint = d7:
##  contrast      estimate   SE   df t.ratio p.value
##  PNS - Control   -0.472 5.37 53.2  -0.088  0.9303
##
## Timepoint = d21:
##  contrast      estimate   SE   df t.ratio p.value
##  PNS - Control    1.934 4.58 42.8   0.422  0.6748
##
## Timepoint = d35:
##  contrast      estimate   SE   df t.ratio p.value
##  PNS - Control   34.232 5.62 51.4   6.088  <.0001
##
## Degrees-of-freedom method: kenward-roger
```

```
#Is there a difference between time points within each treatment?
contrast(IL10_emcatcat, "revpairwise", by="Treatment", adjust="BH")
```

```
## Treatment = Control:
##  contrast  estimate   SE   df t.ratio p.value
##  d7 - d0       1.98 5.24 65.4   0.379  0.7250
##  d21 - d0      3.82 4.62 63.9   0.825  0.6183
##  d21 - d7      1.83 5.19 67.9   0.353  0.7250
##  d35 - d0      8.14 5.06 66.3   1.609  0.6183
##  d35 - d7      6.16 5.46 62.9   1.128  0.6183
##  d35 - d21     4.32 4.98 67.8   0.868  0.6183
##
## Treatment = PNS:
##  contrast  estimate   SE   df t.ratio p.value
##  d7 - d0       1.98 4.54 65.2   0.435  0.6650
##  d21 - d0      6.22 4.34 64.0   1.432  0.2354
##  d21 - d7      4.24 4.32 62.8   0.981  0.3962
##  d35 - d0     42.84 5.00 67.9   8.568  <.0001
##  d35 - d7     40.86 5.05 69.4   8.088  <.0001
##  d35 - d21    36.62 4.88 69.4   7.502  <.0001
##
## Degrees-of-freedom method: kenward-roger
## P value adjustment: BH method for 6 tests
```

Ileum Tissue *Tgfb1* Expression

```
# Fit linear mixed-effects model
# Include random intercept for litter
Tgfb1_model <- lmer(Tgfb1 ~ Timepoint * Treatment + (1 | Litter), data = ileum_dat)
summary(Tgfb1_model)
```

```
## Linear mixed model fit by REML. t-tests use Satterthwaite's method [
## lmerModLmerTest]
## Formula: Tgfb1 ~ Timepoint * Treatment + (1 | Litter)
##    Data: ileum_dat
##
## REML criterion at convergence: 150.2
##
## Scaled residuals:
##     Min      1Q  Median      3Q     Max
## -2.1043 -0.5049  0.0422  0.6095  3.3557
##
## Random effects:
##  Groups   Name        Variance Std.Dev.
##  Litter   (Intercept) 0.1318   0.3630
##  Residual             0.2918   0.5402
## Number of obs: 82, groups:  Litter, 11
##
## Fixed effects:
##                            Estimate Std. Error       df t value Pr(>|t|)
## (Intercept)                 1.11543    0.23700 21.54950   4.706 0.000113 ***
## Timepointd7                 1.62758    0.27052 66.36108   6.016 8.54e-08 ***
## Timepointd21                0.07069    0.23788 65.35584   0.297 0.767295
## Timepointd35                0.23737    0.26170 66.84116   0.907 0.367644
## TreatmentPNS                0.02802    0.32072 21.53445   0.087 0.931185
## Timepointd7:TreatmentPNS  -0.23586    0.35364 65.97473  -0.667 0.507127
## Timepointd21:TreatmentPNS  0.12280    0.31992 65.27324   0.384 0.702340
## Timepointd35:TreatmentPNS  0.34313    0.35817 66.89263   0.958 0.341507
## ---
## Signif. codes:  0 '***' 0.001 '**' 0.01 '*' 0.05 '.' 0.1 ' ' 1
##
## Correlation of Fixed Effects:
##             (Intr) Tmpnt7 Tmpn21 Tmpn35 TrtPNS T7:TPN T21:TP
## Timepointd7 -0.458
## Timepontd21 -0.528  0.444
## Timepontd35 -0.481  0.445  0.467
## TreatmntPNS -0.739  0.339  0.391  0.356
## Tmpnt7:TPNS  0.350 -0.765 -0.340 -0.340 -0.479
## Tmpn21:TPNS  0.393 -0.330 -0.744 -0.347 -0.531  0.474
## Tmpn35:TPNS  0.352 -0.325 -0.341 -0.731 -0.473  0.440  0.465
```

```
# Create a type III ANOVA table
Tgfb1_anova_table <- anova(Tgfb1_model, ddf = "Kenward-Roger")
print(Tgfb1_anova_table)
```

```
## Type III Analysis of Variance Table with Kenward-Roger's method
##                      Sum Sq Mean Sq NumDF  DenDF F value    Pr(>F)
## Timepoint           25.0825  8.3608     3 66.679 28.6489 5.114e-12 ***
## Treatment            0.0334  0.0334     1  9.020  0.1144    0.7429
## Timepoint:Treatment  0.7310  0.2437     3 66.679  0.8350    0.4794
## ---
## Signif. codes:  0 '***' 0.001 '**' 0.01 '*' 0.05 '.' 0.1 ' ' 1
```

```
#Because no interaction, test for main effects of treatment at individual time points
Tgfb1_emcatcat <- emmeans(Tgfb1_model, ~ Treatment | Timepoint)
#Is there a difference between treatment groups at each time point?
contrast(Tgfb1_emcatcat, "revpairwise", adjust="BH")
```

```
## Timepoint = d0:
##  contrast      estimate    SE   df t.ratio p.value
##  PNS - Control    0.028 0.321 22.2   0.087  0.9312
##
## Timepoint = d7:
##  contrast      estimate    SE   df t.ratio p.value
##  PNS - Control   -0.208 0.346 27.9  -0.600  0.5530
##
## Timepoint = d21:
##  contrast      estimate    SE   df t.ratio p.value
##  PNS - Control    0.151 0.310 19.8   0.486  0.6322
##
## Timepoint = d35:
##  contrast      estimate    SE   df t.ratio p.value
##  PNS - Control    0.371 0.351 28.5   1.057  0.2995
##
## Degrees-of-freedom method: kenward-roger
```

Ileum Tissue *Foxp3* Expression

```
# Fit linear mixed-effects model
Foxp3_model <- lmer(Foxp3 ~ Timepoint * Treatment + (1 | Litter), data = ileum_dat) # include random intercept for litter
summary(Foxp3_model)
```

```
## Linear mixed model fit by REML. t-tests use Satterthwaite's method [
## lmerModLmerTest]
## Formula: Foxp3 ~ Timepoint * Treatment + (1 | Litter)
##    Data: ileum_dat
##
## REML criterion at convergence: 686.4
##
## Scaled residuals:
##     Min      1Q  Median      3Q     Max
## -1.7100 -0.5890 -0.0650  0.4259  3.6742
##
## Random effects:
##  Groups   Name        Variance Std.Dev.
##  Litter   (Intercept) 377.8    19.44
##  Residual             672.2    25.93
## Number of obs: 78, groups:  Litter, 11
##
## Fixed effects:
##                           Estimate Std. Error       df t value Pr(>|t|)
## (Intercept)                 0.2456    12.3887  17.2019   0.020  0.98441
## Timepointd7                21.1229    13.1602  59.2654   1.605  0.11380
## Timepointd21               79.0362    12.1616  60.7587   6.499  1.7e-08 ***
## Timepointd35                1.6992    13.1602  59.2654   0.129  0.89770
## TreatmentPNS                1.1684    16.7107  17.0398   0.070  0.94507
## Timepointd7:TreatmentPNS    8.7197    17.2873  59.3319   0.504  0.61585
## Timepointd21:TreatmentPNS -48.8448    16.0882  60.0307  -3.036  0.00354 **
## Timepointd35:TreatmentPNS  33.8132    17.8345  60.1189   1.896  0.06278 .
## ---
## Signif. codes:  0 '***' 0.001 '**' 0.01 '*' 0.05 '.' 0.1 ' ' 1
##
## Correlation of Fixed Effects:
##             (Intr) Tmpnt7 Tmpn21 Tmpn35 TrtPNS T7:TPN T21:TP
## Timepointd7 -0.453
## Timepontd21 -0.518  0.450
## Timepontd35 -0.453  0.445  0.450
## TreatmntPNS -0.741  0.336  0.384  0.336
## Tmpnt7:TPNS  0.345 -0.761 -0.343 -0.339 -0.475
## Tmpn21:TPNS  0.392 -0.340 -0.756 -0.340 -0.523  0.487
## Tmpn35:TPNS  0.334 -0.329 -0.332 -0.738 -0.458  0.452  0.467
```

```
# Create a type III ANOVA table
Foxp3_anova_table <- anova(Foxp3_model, ddf = "Kenward-Roger")
print(Foxp3_anova_table)
```

```
## Type III Analysis of Variance Table with Kenward-Roger's method
##                      Sum Sq Mean Sq NumDF  DenDF F value    Pr(>F)
## Timepoint             31791 10597.0     3 62.642 15.7632 9.564e-08 ***
## Treatment                 1     0.6     1  9.014  0.0009 0.9761977
## Timepoint:Treatment  16438  5479.3     3 62.642  8.1505 0.0001157 ***
## ---
## Signif. codes:  0 '***' 0.001 '**' 0.01 '*' 0.05 '.' 0.1 ' ' 1
```

```
# Because we have an interaction
Foxp3_emcatcat <- emmeans(Foxp3_model, ~ Timepoint * Treatment)

#Is there a difference between treatment groups at each time point?
contrast(Foxp3_emcatcat, "revpairwise", by="Timepoint", adjust="BH")
```

```
## Timepoint = d0:
##  contrast      estimate   SE   df t.ratio p.value
##  PNS - Control     1.17 16.7 21.1   0.070  0.9450
##
## Timepoint = d7:
##  contrast      estimate   SE   df t.ratio p.value
##  PNS - Control     9.89 17.5 24.0   0.566  0.5764
##
## Timepoint = d21:
##  contrast      estimate   SE   df t.ratio p.value
##  PNS - Control   -47.68 16.0 18.2  -2.972  0.0081
##
## Timepoint = d35:
##  contrast      estimate   SE   df t.ratio p.value
##  PNS - Control    34.98 18.1 26.3   1.936  0.0637
##
## Degrees-of-freedom method: kenward-roger
```

```
#Is there a difference between time points within each treatment?
contrast(Foxp3_emcatcat, "revpairwise", by="Treatment", adjust="BH")
```

```
## Treatment = Control:
##  contrast  estimate   SE   df t.ratio p.value
##  d7 - d0     21.123 13.2 61.7   1.603  0.1710
##  d21 - d0    79.036 12.2 62.9   6.476  <.0001
##  d21 - d7    57.913 13.4 64.6   4.323  0.0001
##  d35 - d0     1.699 13.2 61.7   0.129  0.8978
##  d35 - d7   -19.424 13.9 61.0  -1.402  0.1993
##  d35 - d21  -77.337 13.4 64.6  -5.773  <.0001
##
## Treatment = PNS:
##  contrast  estimate   SE   df t.ratio p.value
##  d7 - d0     29.843 11.2 61.8   2.658  0.0200
##  d21 - d0    30.191 10.5 61.5   2.864  0.0172
##  d21 - d7     0.349 10.5 61.2   0.033  0.9736
##  d35 - d0    35.512 12.1 63.2   2.937  0.0172
##  d35 - d7     5.670 12.1 63.3   0.467  0.7758
##  d35 - d21    5.321 11.5 63.3   0.461  0.7758
##
## Degrees-of-freedom method: kenward-roger
## P value adjustment: BH method for 6 tests
```

Ileum Tissue *Il18* Expression

```
# Fit linear mixed-effects model
# Include random intercept for Litter
IL18_model <- lmer(IL18 ~ Timepoint * Treatment + (1 | Litter), data = ileum_dat)
summary(IL18_model)
```

```
## Linear mixed model fit by REML. t-tests use Satterthwaite's method [
## lmerModLmerTest]
## Formula: IL18 ~ Timepoint * Treatment + (1 | Litter)
##    Data: ileum_dat
##
## REML criterion at convergence: 248.9
##
## Scaled residuals:
##     Min      1Q  Median      3Q     Max
## -2.0516 -0.4313 -0.0435  0.4274  5.7577
##
## Random effects:
##  Groups   Name        Variance Std.Dev.
##  Litter   (Intercept) 0.06887  0.2624
##  Residual             1.45866  1.2077
## Number of obs: 79, groups:  Litter, 11
##
## Fixed effects:
##                             Estimate Std. Error      df t value Pr(>|t|)
## (Intercept)                   1.0993     0.4009 48.3575   2.742  0.00853 **
## Timepointd7                   1.8364     0.5978 66.0900   3.072  0.00309 **
## Timepointd21                 -0.3094     0.5290 64.7763  -0.585  0.56070
## Timepointd35                  0.9671     0.5978 66.0900   1.618  0.11050
## TreatmentPNS                  0.0449     0.5426 48.5724   0.083  0.93439
## Timepointd7:TreatmentPNS   -1.1601     0.7832 65.8034  -1.481  0.14331
## Timepointd21:TreatmentPNS   0.7496     0.7183 65.3857   1.044  0.30053
## Timepointd35:TreatmentPNS   2.5746     0.8167 67.3257   3.152  0.00242 **
## ---
## Signif. codes:  0 '***' 0.001 '**' 0.01 '*' 0.05 '.' 0.1 ' ' 1
##
## Correlation of Fixed Effects:
##             (Intr) Tmpnt7 Tmpn21 Tmpn35 TrtPNS T7:TPN T21:TP
## Timepointd7 -0.610
## Timepontd21 -0.693  0.459
## Timepontd35 -0.610  0.417  0.459
## TreatmntPNS -0.739  0.451  0.512  0.451
## Tmpnt7:TPNS  0.466 -0.763 -0.350 -0.318 -0.633
## Tmpn21:TPNS  0.510 -0.338 -0.737 -0.338 -0.692  0.477
## Tmpn35:TPNS  0.446 -0.305 -0.336 -0.732 -0.604  0.422  0.453
```

```
# Create a type III ANOVA table
IL18_anova_table <- anova(IL18_model, ddf = "Kenward-Roger")
print(IL18_anova_table)
```

```
## Type III Analysis of Variance Table with Kenward-Roger's method
##                     Sum Sq Mean Sq NumDF  DenDF F value    Pr(>F)
## Timepoint           58.499 19.4996     3 66.356 13.3663 6.381e-07 ***
## Treatment            4.739  4.7385     1  8.682  3.2486 0.1062165
## Timepoint:Treatment 28.882  9.6272     3 66.356  6.5992 0.0005704 ***
## ---
## Signif. codes:  0 '***' 0.001 '**' 0.01 '*' 0.05 '.' 0.1 ' ' 1
```

```
# Because we have an interaction
IL18_emcatcat <- emmeans(IL18_model, ~ Timepoint * Treatment)

#Is there a difference between treatment groups at each time point?
contrast(IL18_emcatcat, "revpairwise", by="Timepoint", adjust="BH")
```

```
## Timepoint = d0:
##  contrast      estimate    SE   df t.ratio p.value
##  PNS - Control   0.0449 0.545 46.7   0.082  0.9347
##
## Timepoint = d7:
##  contrast      estimate    SE   df t.ratio p.value
##  PNS - Control  -1.1152 0.613 54.1  -1.820  0.0743
##
## Timepoint = d21:
##  contrast      estimate    SE   df t.ratio p.value
##  PNS - Control   0.7945 0.523 43.3   1.520  0.1357
##
## Timepoint = d35:
##  contrast      estimate    SE   df t.ratio p.value
##  PNS - Control   2.6195 0.663 53.9   3.952  0.0002
##
## Degrees-of-freedom method: kenward-roger
```

```
#Is there a difference between time points within each treatment?
contrast(IL18_emcatcat, "revpairwise", by="Treatment", adjust="BH")
```

```
## Treatment = Control:
##  contrast  estimate    SE   df t.ratio p.value
##  d7 - d0      1.836 0.601 65.5   3.054  0.0098
##  d21 - d0    -0.309 0.531 64.0  -0.583  0.5622
##  d21 - d7    -2.146 0.596 68.1  -3.602  0.0036
##  d35 - d0     0.967 0.601 65.5   1.609  0.1688
##  d35 - d7    -0.869 0.646 62.2  -1.347  0.2196
##  d35 - d21    1.276 0.596 68.1   2.143  0.0714
##
## Treatment = PNS:
##  contrast  estimate    SE   df t.ratio p.value
##  d7 - d0      0.676 0.509 64.7   1.329  0.2828
##  d21 - d0     0.440 0.489 65.5   0.899  0.4461
##  d21 - d7    -0.236 0.496 62.8  -0.476  0.6356
##  d35 - d0     3.542 0.564 68.2   6.281  <.0001
##  d35 - d7     2.865 0.579 69.8   4.945  <.0001
##  d35 - d21    3.102 0.566 70.8   5.481  <.0001
##
## Degrees-of-freedom method: kenward-roger
## P value adjustment: BH method for 6 tests
```

Ileum Tissue *Il4* Expression

```
# Fit linear mixed-effects model
# Include random intercept for litter
IL4_model <- lmer(IL4 ~ Timepoint * Treatment + (1 | Litter), data = ileum_dat)
summary(IL4_model)
```

```
## Linear mixed model fit by REML. t-tests use Satterthwaite's method [
## lmerModLmerTest]
## Formula: IL4 ~ Timepoint * Treatment + (1 | Litter)
##    Data: ileum_dat
##
## REML criterion at convergence: 696.3
##
## Scaled residuals:
##      Min       1Q   Median       3Q      Max
## -1.43387 -0.73307 -0.05931  0.26114  2.80372
##
## Random effects:
##  Groups   Name        Variance Std.Dev.
##  Litter   (Intercept)   99.84   9.992
##  Residual             1392.50  37.316
## Number of obs: 75, groups:  Litter, 11
##
## Fixed effects:
##                           Estimate Std. Error       df t value Pr(>|t|)
## (Intercept)                 0.8803    14.8519  51.0738   0.059  0.95297
## Timepointd7                55.4907    20.0224  58.3118   2.771  0.00748 **
## Timepointd21               45.0697    18.1252  58.7150   2.487  0.01576 *
## Timepointd35                5.7161    20.0224  58.3118   0.285  0.77628
## TreatmentPNS                4.7859    19.0863  47.0732   0.251  0.80310
## Timepointd7:TreatmentPNS  -11.8535    25.5968  57.8792  -0.463  0.64504
## Timepointd21:TreatmentPNS -14.7016    23.7416  58.2818  -0.619  0.53818
## Timepointd35:TreatmentPNS  32.8432    26.6351  60.3791   1.233  0.22233
## ---
## Signif. codes:  0 '***' 0.001 '**' 0.01 '*' 0.05 '.' 0.1 ' ' 1
##
## Correlation of Fixed Effects:
##             (Intr) Tmpnt7 Tmpn21 Tmpn35 TrtPNS T7:TPN T21:TP
## Timepointd7 -0.668
## Timepontd21 -0.747  0.545
## Timepontd35 -0.668  0.504  0.545
## TreatmntPNS -0.778  0.520  0.581  0.520
## Tmpnt7:TPNS  0.523 -0.782 -0.426 -0.394 -0.667
## Tmpn21:TPNS  0.570 -0.416 -0.763 -0.416 -0.724  0.535
## Tmpn35:TPNS  0.503 -0.379 -0.410 -0.752 -0.640  0.481  0.512
```

```
# Create a type III ANOVA table
IL4_anova_table <- anova(IL4_model, ddf = "Kenward-Roger")
print(IL4_anova_table)
```

```
## Type III Analysis of Variance Table with Kenward-Roger's method
##                     Sum Sq Mean Sq NumDF  DenDF F value   Pr(>F)
## Timepoint          23863.5  7954.5     3 61.782  5.7117 0.001621 **
## Treatment            473.9   473.9     1  8.770  0.3403 0.574335
## Timepoint:Treatment  5597.7  1865.9     3 61.782  1.3398 0.269651
## ---
## Signif. codes:  0 '***' 0.001 '**' 0.01 '*' 0.05 '.' 0.1 ' ' 1
```

```
#Because no interaction, test for main effects of treatment at individual time points
IL4_emcatcat <- emmeans(IL4_model, ~ Treatment | Timepoint)
#Is there a difference between treatment groups at each time point?
contrast(IL4_emcatcat, "revpairwise", adjust="BH")
```

```
## Timepoint = d0:
##  contrast      estimate   SE   df t.ratio p.value
##  PNS - Control     4.79 19.1 50.7   0.250  0.8036
##
## Timepoint = d7:
##  contrast      estimate   SE   df t.ratio p.value
##  PNS - Control    -7.07 19.3 48.2  -0.366  0.7158
##
## Timepoint = d21:
##  contrast      estimate   SE   df t.ratio p.value
##  PNS - Control    -9.92 16.5 37.7  -0.599  0.5525
##
## Timepoint = d35:
##  contrast      estimate   SE   df t.ratio p.value
##  PNS - Control    37.63 20.9 49.0   1.803  0.0775
##
## Degrees-of-freedom method: kenward-roger
```

Ileum Tissue *Il5* Expression

```
# Fit linear mixed-effects model
# Include random intercept for litter
IL5_model <- lmer(IL5 ~ Timepoint * Treatment + (1 | Litter), data = ileum_dat)
summary(IL5_model)
```

```
## Linear mixed model fit by REML. t-tests use Satterthwaite's method [
## lmerModLmerTest]
## Formula: IL5 ~ Timepoint * Treatment + (1 | Litter)
##    Data: ileum_dat
##
## REML criterion at convergence: 213.5
##
## Scaled residuals:
##     Min      1Q  Median      3Q     Max
## -2.1609 -0.1394 -0.0084  0.2566  4.0996
##
## Random effects:
##  Groups   Name        Variance Std.Dev.
##  Litter   (Intercept) 0.5090   0.7135
##  Residual             0.7685   0.8767
## Number of obs: 78, groups:  Litter, 11
##
## Fixed effects:
##                            Estimate Std. Error      df t value Pr(>|t|)
## (Intercept)                  0.9252     0.4361 18.9585   2.121   0.0473 *
## Timepointd7                 -0.4990     0.4533 62.3570  -1.101   0.2753
## Timepointd21                -0.5947     0.4091 61.4386  -1.453   0.1512
## Timepointd35                -0.4348     0.4389 62.6054  -0.991   0.3257
## TreatmentPNS                 1.3415     0.5833 18.1733   2.300   0.0335 *
## Timepointd7:TreatmentPNS   -0.9655     0.5917 61.8946  -1.632   0.1078
## Timepointd21:TreatmentPNS  -1.1121     0.5417 61.3459  -2.053   0.0443 *
## Timepointd35:TreatmentPNS  -0.9135     0.5930 62.5560  -1.541   0.1285
## ---
## Signif. codes:  0 '***' 0.001 '**' 0.01 '*' 0.05 '.' 0.1 ' ' 1
##
## Correlation of Fixed Effects:
##             (Intr) Tmpnt7 Tmpn21 Tmpn35 TrtPNS T7:TPN T21:TP
## Timepointd7 -0.446
## Timepontd21 -0.495  0.456
## Timepontd35 -0.467  0.479  0.479
## TreatmntPNS -0.748  0.333  0.370  0.349
## Tmpnt7:TPNS  0.342 -0.766 -0.349 -0.367 -0.446
## Tmpn21:TPNS  0.374 -0.344 -0.755 -0.362 -0.486  0.467
## Tmpn35:TPNS  0.345 -0.355 -0.354 -0.740 -0.445  0.458  0.471
```

```
# Create a type III ANOVA table
IL5_anova_table <- anova(IL5_model, ddf = "Kenward-Roger")
print(IL5_anova_table)
```

```
## Type III Analysis of Variance Table with Kenward-Roger's method
##                      Sum Sq Mean Sq NumDF  DenDF F value    Pr(>F)
## Timepoint           15.9688  5.3229     3 62.562  6.9261 0.0004234 ***
## Treatment            1.1793  1.1793     1  8.951  1.5345 0.2469432
## Timepoint:Treatment  3.8212  1.2737     3 62.562  1.6573 0.1852977
## ---
## Signif. codes:  0 '***' 0.001 '**' 0.01 '*' 0.05 '.' 0.1 ' ' 1
```

```
#Because no interaction, test for main effects of treatment at individual time points
IL5_emcatcat <- emmeans(IL5_model, ~ Treatment | Timepoint)
#Is there a difference between treatment groups at each time point?
contrast(IL5_emcatcat, "revpairwise", adjust="BH")
```

```
## Timepoint = d0:
##  contrast      estimate    SE   df t.ratio p.value
##  PNS - Control    1.341 0.584 19.0   2.298  0.0331
##
## Timepoint = d7:
##  contrast      estimate    SE   df t.ratio p.value
##  PNS - Control    0.376 0.619 23.0   0.607  0.5498
##
## Timepoint = d21:
##  contrast      estimate    SE   df t.ratio p.value
##  PNS - Control    0.229 0.572 17.6   0.401  0.6932
##
## Timepoint = d35:
##  contrast      estimate    SE   df t.ratio p.value
##  PNS - Control    0.428 0.622 22.9   0.689  0.4980
##
## Degrees-of-freedom method: kenward-roger
```

Ileum Tissue *Il13* Expression

```
# Fit linear mixed-effects model
# Include random intercept for litter
IL13_model <- lmer(IL13 ~ Timepoint * Treatment + (1 | Litter), data = ileum_dat)
summary(IL13_model)
```

```
## Linear mixed model fit by REML. t-tests use Satterthwaite's method [
## lmerModLmerTest]
## Formula: IL13 ~ Timepoint * Treatment + (1 | Litter)
##    Data: ileum_dat
##
## REML criterion at convergence: 80
##
## Scaled residuals:
##      Min       1Q   Median       3Q      Max
## -1.94414 -0.68043 -0.04224  0.42599  2.16060
##
## Random effects:
##  Groups   Name        Variance Std.Dev.
##  Litter   (Intercept) 0.006456 0.08035
##  Residual             0.141034 0.37554
## Number of obs: 76, groups:  Litter, 11
##
## Fixed effects:
##                            Estimate Std. Error       df t value Pr(>|t|)
## (Intercept)                 0.99117    0.13088 49.84369   7.573 7.79e-10 ***
## Timepointd7                -0.54672    0.19049 64.65729  -2.870  0.00554 **
## Timepointd21               -0.50857    0.17304 62.32129  -2.939  0.00461 **
## Timepointd35               -0.45171    0.19049 64.65729  -2.371  0.02071 *
## TreatmentPNS               -0.08259    0.17647 49.78327  -0.468  0.64181
## Timepointd7:TreatmentPNS    0.55730    0.25182 63.90355   2.213  0.03047 *
## Timepointd21:TreatmentPNS   0.45098    0.23211 63.08093   1.943  0.05649 .
## Timepointd35:TreatmentPNS   0.08438    0.25518 64.48770   0.331  0.74198
## ---
## Signif. codes:  0 '***' 0.001 '**' 0.01 '*' 0.05 '.' 0.1 ' ' 1
##
## Correlation of Fixed Effects:
##             (Intr) Tmpnt7 Tmpn21 Tmpn35 TrtPNS T7:TPN T21:TP
## Timepointd7 -0.634
## Timepontd21 -0.699  0.480
## Timepontd35 -0.634  0.442  0.480
## TreatmntPNS -0.742  0.470  0.519  0.470
## Tmpnt7:TPNS  0.480 -0.756 -0.363 -0.334 -0.647
## Tmpn21:TPNS  0.521 -0.358 -0.745 -0.358 -0.703  0.492
## Tmpn35:TPNS  0.473 -0.330 -0.358 -0.746 -0.636  0.449  0.484
```

```
# Create a type III ANOVA table
IL13_anova_table <- anova(IL13_model, ddf = "Kenward-Roger")
print(IL13_anova_table)
```

```
## Type III Analysis of Variance Table with Kenward-Roger's method
##                      Sum Sq Mean Sq NumDF  DenDF F value  Pr(>F)
## Timepoint           1.60113 0.53371     3 63.228  3.7842 0.01461 *
## Treatment           0.49014 0.49014     1  8.383  3.4753 0.09760 .
## Timepoint:Treatment 0.99391 0.33130     3 63.228  2.3491 0.08090 .
## ---
## Signif. codes:  0 '***' 0.001 '**' 0.01 '*' 0.05 '.' 0.1 ' ' 1
```

```
#Because no interaction, test for main effects of treatment at individual time points
IL13_emcatcat <- emmeans(IL13_model, ~ Treatment | Timepoint)
#Is there a difference between treatment groups at each time point?
contrast(IL13_emcatcat, "revpairwise", adjust="BH")
```

```
## Timepoint = d0:
##  contrast      estimate    SE   df t.ratio p.value
##  PNS - Control -0.08259 0.178 47.4  -0.464  0.6444
##
## Timepoint = d7:
##  contrast      estimate    SE   df t.ratio p.value
##  PNS - Control  0.47470 0.194 52.9   2.448  0.0177
##
## Timepoint = d21:
##  contrast      estimate    SE   df t.ratio p.value
##  PNS - Control  0.36839 0.166 44.2   2.220  0.0316
##
## Timepoint = d35:
##  contrast      estimate    SE   df t.ratio p.value
##  PNS - Control  0.00178 0.200 50.2   0.009  0.9929
##
## Degrees-of-freedom method: kenward-roger
```

Ileum Tissue *Il17a* Expression

```
# Fit linear mixed-effects model
# Include random intercept for litter
IL17_model <- lmer(IL17 ~ Timepoint * Treatment + (1 | Litter), data = ileum_dat)
summary(IL17_model)
```

```
## Linear mixed model fit by REML. t-tests use Satterthwaite's method [
## lmerModLmerTest]
## Formula: IL17 ~ Timepoint * Treatment + (1 | Litter)
##    Data: ileum_dat
##
## REML criterion at convergence: 373.5
##
## Scaled residuals:
##     Min      1Q  Median      3Q     Max
## -2.3879 -0.1843 -0.0273  0.0658  3.2013
##
## Random effects:
##  Groups   Name        Variance Std.Dev.
##  Litter   (Intercept)  4.06    2.015
##  Residual             55.02    7.418
## Number of obs: 60, groups:  Litter, 11
##
## Fixed effects:
##                             Estimate Std. Error      df t value Pr(>|t|)
## (Intercept)                   1.0142     5.4149 51.7226   0.187   0.8522
## Timepointd7                  -0.2195     6.0833 51.7494  -0.036   0.9714
## Timepointd21                  0.2666     5.8739 50.0790   0.045   0.9640
## Timepointd35                 -1.0622     7.6398 51.9959  -0.139   0.8900
## TreatmentPNS                 -1.0035     6.0187 50.8372  -0.167   0.8682
## Timepointd7:TreatmentPNS      1.2093     6.9520 51.3172   0.174   0.8626
## Timepointd21:TreatmentPNS     4.8266     6.7694 49.7576   0.713   0.4792
## Timepointd35:TreatmentPNS    21.5183     8.4195 51.9459   2.556   0.0136 *
## ---
## Signif. codes:  0 '***' 0.001 '**' 0.01 '*' 0.05 '.' 0.1 ' ' 1
##
## Correlation of Fixed Effects:
##             (Intr) Tmpnt7 Tmpn21 Tmpn35 TrtPNS T7:TPN T21:TP
## Timepointd7 -0.872
## Timepontd21 -0.894  0.801
## Timepontd35 -0.706  0.634  0.646
## TreatmntPNS -0.900  0.784  0.804  0.635
## Tmpnt7:TPNS  0.763 -0.875 -0.701 -0.555 -0.836
## Tmpn21:TPNS  0.776 -0.695 -0.868 -0.561 -0.851  0.741
## Tmpn35:TPNS  0.640 -0.575 -0.586 -0.907 -0.698  0.609  0.619
```

```
# Create a type III ANOVA table
IL17_anova_table <- anova(IL17_model, ddf = "Kenward-Roger")
print(IL17_anova_table)
```

```
## Type III Analysis of Variance Table with Kenward-Roger's method
##                     Sum Sq Mean Sq NumDF  DenDF F value  Pr(>F)
## Timepoint           421.29  140.43     3 49.118  2.5509 0.06629 .
## Treatment           259.57  259.57     1 14.947  4.7176 0.04636 *
## Timepoint:Treatment 504.85  168.28     3 49.118  3.0569 0.03687 *
## ---
## Signif. codes:  0 '***' 0.001 '**' 0.01 '*' 0.05 '.' 0.1 ' ' 1
```

```
# Because we have an interaction
IL17_emcatcat <- emmeans(IL17_model, ~ Timepoint * Treatment)

#Is there a difference between treatment groups at each time point?
contrast(IL17_emcatcat, "revpairwise", by="Timepoint", adjust="BH")
```

```
## Timepoint = d0:
##  contrast      estimate   SE   df t.ratio p.value
##  PNS - Control   -1.004 6.12 50.3  -0.164  0.8703
##
## Timepoint = d7:
##  contrast      estimate   SE   df t.ratio p.value
##  PNS - Control    0.206 3.85 36.4   0.053  0.9577
##
## Timepoint = d21:
##  contrast      estimate   SE   df t.ratio p.value
##  PNS - Control    3.823 3.58 33.9   1.067  0.2934
##
## Timepoint = d35:
##  contrast      estimate   SE   df t.ratio p.value
##  PNS - Control   20.515 6.15 49.7   3.338  0.0016
##
## Degrees-of-freedom method: kenward-roger
```

```
#Is there a difference between time points within each treatment?
contrast(IL17_emcatcat, "revpairwise", by="Treatment", adjust="BH")
```

```
## Treatment = Control:
##  contrast  estimate   SE   df t.ratio p.value
##  d7 - d0     -0.220 6.21 51.6  -0.035  0.9719
##  d21 - d0     0.267 5.95 49.1   0.045  0.9719
##  d21 - d7     0.486 3.82 48.6   0.127  0.9719
##  d35 - d0    -1.062 7.85 52.0  -0.135  0.9719
##  d35 - d7    -0.843 6.14 49.7  -0.137  0.9719
##  d35 - d21   -1.329 6.01 50.7  -0.221  0.9719
##
## Treatment = PNS:
##  contrast  estimate   SE   df t.ratio p.value
##  d7 - d0      0.990 3.41 47.0   0.290  0.7732
##  d21 - d0     5.093 3.41 47.1   1.493  0.2131
##  d21 - d7     4.103 3.20 45.6   1.283  0.2473
##  d35 - d0    20.456 3.59 48.3   5.692  <.0001
##  d35 - d7    19.466 3.47 49.7   5.617  <.0001
##  d35 - d21   15.363 3.38 45.9   4.545  0.0001
##
## Degrees-of-freedom method: kenward-roger
## P value adjustment: BH method for 6 tests
```

Sex Differences in Ileum Tissue Gene Expression

Ileum Tissue *Ifng* Expression Sex Differences

```
# Does ileum Ifny expression differ by sex
#Include random intercept for litter
Ifng_sex_effects <- lmer(Ifng ~ Timepoint * Treatment * Sex + (1 | Litter), data = ileum_dat)
```

```
## fixed-effect model matrix is rank deficient so dropping 1 column / coefficient
```

```
Ifng_anova_table_for_sex <- anova(Ifng_sex_effects, ddf = "Kenward-Roger")
```

```
## Missing cells for: Timepointd7:TreatmentControl:SexM.
## Interpret type III hypotheses with care.
```

```
print(Ifng_anova_table_for_sex)
```

```
## Type III Analysis of Variance Table with Kenward-Roger's method
##                         Sum Sq Mean Sq NumDF  DenDF F value  Pr(>F)
## Timepoint               97.995  48.998     2 39.789  1.6549 0.20400
## Treatment              100.675 100.675     1 11.962  3.4003 0.09008 .
## Sex                     11.828  11.828     1 43.488  0.3995 0.53067
## Timepoint:Treatment    142.384  71.192     2 40.537  2.4044 0.10309
## Timepoint:Sex           98.684  49.342     2 41.556  1.6664 0.20130
## Treatment:Sex           16.894  16.894     1 43.020  0.5706 0.45413
## Timepoint:Treatment:Sex 124.321 124.321     1 40.982  4.1990 0.04689 *
## ---
## Signif. codes:  0 '***' 0.001 '**' 0.01 '*' 0.05 '.' 0.1 ' ' 1
```

```
Ifng_emcatcat_sex <- emmeans(Ifng_sex_effects, ~ Timepoint * Treatment * Sex)
#Is there a difference in males or females between treatment group at each time point?
contrast(Ifng_emcatcat_sex, "revpairwise", by=c("Sex", "Timepoint"), adjust="BH")
```

```
## Sex = F, Timepoint = d7:
##  contrast      estimate   SE   df t.ratio p.value
##  PNS - Control    3.722 4.36 32.1   0.854  0.3993
##
## Sex = M, Timepoint = d7:
##  contrast      estimate   SE   df t.ratio p.value
##  PNS - Control   nonEst   NA   NA      NA      NA
##
## Sex = F, Timepoint = d21:
##  contrast      estimate   SE   df t.ratio p.value
##  PNS - Control   -0.991 4.13 26.9  -0.240  0.8124
##
## Sex = M, Timepoint = d21:
##  contrast      estimate   SE   df t.ratio p.value
##  PNS - Control    4.871 4.32 28.5   1.127  0.2692
##
## Sex = F, Timepoint = d35:
##  contrast      estimate   SE   df t.ratio p.value
##  PNS - Control   18.011 5.13 40.3   3.508  0.0011
##
## Sex = M, Timepoint = d35:
##  contrast      estimate   SE   df t.ratio p.value
##  PNS - Control    4.749 6.70 44.8   0.709  0.4819
##
## Degrees-of-freedom method: kenward-roger
```

```
#Is there a difference in males or females within treatment group at each time point?
contrast(Ifng_emcatcat_sex, "revpairwise", by=c("Treatment", "Timepoint"), adjust="BH")
```

```
## Treatment = Control, Timepoint = d7:
##  contrast estimate   SE   df t.ratio p.value
##  M - F      nonEst   NA   NA      NA      NA
##
## Treatment = PNS, Timepoint = d7:
##  contrast estimate   SE   df t.ratio p.value
##  M - F      0.8406 3.97 42.3   0.212  0.8332
##
## Treatment = Control, Timepoint = d21:
##  contrast estimate   SE   df t.ratio p.value
##  M - F     -1.3902 4.35 44.0  -0.320  0.7508
##
## Treatment = PNS, Timepoint = d21:
##  contrast estimate   SE   df t.ratio p.value
##  M - F      4.4717 3.33 40.0   1.343  0.1868
##
## Treatment = Control, Timepoint = d35:
##  contrast estimate   SE   df t.ratio p.value
##  M - F      0.0898 4.71 39.1   0.019  0.9849
##
## Treatment = PNS, Timepoint = d35:
##  contrast estimate   SE   df t.ratio p.value
##  M - F    -13.1715 6.27 40.1  -2.099  0.0421
##
## Degrees-of-freedom method: kenward-roger
```

Ileum Tissue *Ifnb* Expression Sex Differences

```
# Does ileum Ifnb expression differ by sex
#Include random intercept for litter
Ifnb_sex_effects <- lmer(Ifnb ~ Timepoint * Treatment * Sex + (1 | Litter), data = ileum_dat)
```

```
## fixed-effect model matrix is rank deficient so dropping 1 column / coefficient
```

```
Ifnb_anova_table_for_sex <- anova(Ifnb_sex_effects, ddf = "Kenward-Roger")
```

```
## Missing cells for: Timepointd7:TreatmentControl:SexM.
## Interpret type III hypotheses with care.
```

```
print(Ifnb_anova_table_for_sex)
```

```
## Type III Analysis of Variance Table with Kenward-Roger's method
##                         Sum Sq Mean Sq NumDF  DenDF F value Pr(>F)
## Timepoint              10.2577  5.1289     2 37.821  1.4405 0.2495
## Treatment               5.8236  5.8236     1 10.351  1.6356 0.2289
## Sex                     1.5818  1.5818     1 39.814  0.4443 0.5089
## Timepoint:Treatment     9.1726  4.5863     2 38.107  1.2881 0.2875
## Timepoint:Sex           9.4353  4.7177     2 38.550  1.3250 0.2776
## Treatment:Sex           0.0556  0.0556     1 39.479  0.0156 0.9012
## Timepoint:Treatment:Sex  4.2687  4.2687     1 38.476  1.1989 0.2803
```

```
Ifnb_emcatcat_sex <- emmeans(Ifnb_sex_effects, ~ Timepoint * Treatment * Sex)
#Is there a difference in males or females between treatment group at each time point?
contrast(Ifnb_emcatcat_sex, "revpairwise", by=c("Sex", "Timepoint"), adjust="BH")
```

```
## Sex = F, Timepoint = d7:
##  contrast      estimate   SE   df t.ratio p.value
##  PNS - Control  1.27579 1.93 20.3   0.660  0.5165
##
## Sex = M, Timepoint = d7:
##  contrast      estimate   SE   df t.ratio p.value
##  PNS - Control   nonEst   NA   NA      NA      NA
##
## Sex = F, Timepoint = d21:
##  contrast      estimate   SE   df t.ratio p.value
##  PNS - Control  0.00659 1.88 18.1   0.003  0.9972
##
## Sex = M, Timepoint = d21:
##  contrast      estimate   SE   df t.ratio p.value
##  PNS - Control  2.02416 1.94 19.6   1.044  0.3091
##
## Sex = F, Timepoint = d35:
##  contrast      estimate   SE   df t.ratio p.value
##  PNS - Control  4.31980 2.15 27.4   2.009  0.0545
##
## Sex = M, Timepoint = d35:
##  contrast      estimate   SE   df t.ratio p.value
##  PNS - Control  2.73624 2.62 38.9   1.043  0.3035
##
## Degrees-of-freedom method: kenward-roger
```

```
#Is there a difference in males or females within treatment group at each time point?
contrast(Ifnb_emcatcat_sex, "revpairwise", by=c("Treatment", "Timepoint"), adjust="BH")
```

```
## Treatment = Control, Timepoint = d7:
##  contrast estimate   SE   df t.ratio p.value
##  M - F      nonEst   NA   NA      NA      NA
##
## Treatment = PNS, Timepoint = d7:
##  contrast estimate   SE   df t.ratio p.value
##  M - F       2.769 1.39 38.7   1.985  0.0543
##
## Treatment = Control, Timepoint = d21:
##  contrast estimate   SE   df t.ratio p.value
##  M - F      -0.613 1.61 43.5  -0.380  0.7057
##
## Treatment = PNS, Timepoint = d21:
##  contrast estimate   SE   df t.ratio p.value
##  M - F       1.404 1.16 37.6   1.207  0.2349
##
## Treatment = Control, Timepoint = d35:
##  contrast estimate   SE   df t.ratio p.value
##  M - F      -0.135 1.64 37.1  -0.082  0.9350
##
## Treatment = PNS, Timepoint = d35:
##  contrast estimate   SE   df t.ratio p.value
##  M - F      -1.718 2.19 37.5  -0.785  0.4375
##
## Degrees-of-freedom method: kenward-roger
```

Ileum Tissue *Isg15* Expression Sex Differences

```
# Does ileum Isg15 expression differ by sex
#Include random intercept for litter
Isg15_sex_effects <- lmer(Isg15 ~ Timepoint * Treatment * Sex + (1 | Litter), data = ileum_dat)
Isg15_anova_table_for_sex <- anova(Isg15_sex_effects, ddf = "Kenward-Roger")
print(Isg15_anova_table_for_sex)
```

```
## Type III Analysis of Variance Table with Kenward-Roger's method
##                         Sum Sq Mean Sq NumDF  DenDF F value   Pr(>F)
## Timepoint               3.5043  1.7522     2 39.164  1.1403 0.330122
## Treatment               5.5549  5.5549     1  9.615  3.6150 0.087612 .
## Sex                     1.8427  1.8427     1 39.420  1.1992 0.280124
## Timepoint:Treatment    18.1604  9.0802     2 39.164  5.9091 0.005718 **
## Timepoint:Sex           1.0653  0.5327     2 41.424  0.3465 0.709157
## Treatment:Sex           0.7930  0.7930     1 39.420  0.5161 0.476761
## Timepoint:Treatment:Sex  4.1213  2.0607     2 41.424  1.3407 0.272789
## ---
## Signif. codes:  0 '***' 0.001 '**' 0.01 '*' 0.05 '.' 0.1 ' ' 1
```

```
Isg15_emcatcat_sex <- emmeans(Isg15_sex_effects, ~ Timepoint * Treatment * Sex)
#Is there a difference in males or females between treatment group at each time point?
contrast(Isg15_emcatcat_sex, "revpairwise", by=c("Sex", "Timepoint"), adjust="BH")
```

```
## Sex = F, Timepoint = d7:
##  contrast      estimate   SE   df t.ratio p.value
##  PNS - Control  -0.0205 1.16 24.0  -0.018  0.9861
##
## Sex = M, Timepoint = d7:
##  contrast      estimate   SE   df t.ratio p.value
##  PNS - Control  -0.0710 1.62 42.7  -0.044  0.9653
##
## Sex = F, Timepoint = d21:
##  contrast      estimate   SE   df t.ratio p.value
##  PNS - Control   1.6712 1.15 21.9   1.448  0.1618
##
## Sex = M, Timepoint = d21:
##  contrast      estimate   SE   df t.ratio p.value
##  PNS - Control   1.0604 1.17 22.7   0.908  0.3735
##
## Sex = F, Timepoint = d35:
##  contrast      estimate   SE   df t.ratio p.value
##  PNS - Control   2.4473 1.31 32.5   1.864  0.0714
##
## Sex = M, Timepoint = d35:
##  contrast      estimate   SE   df t.ratio p.value
##  PNS - Control   4.9223 1.33 33.1   3.688  0.0008
##
## Degrees-of-freedom method: kenward-roger
```

```
#Is there a difference in males or females within treatment group at each time point?
contrast(Isg15_emcatcat_sex, "revpairwise", by=c("Treatment", "Timepoint"), adjust="BH")
```

```
## Treatment = Control, Timepoint = d7:
##  contrast estimate    SE   df t.ratio p.value
##  M - F      0.0686 1.460 39.7   0.047  0.9627
##
## Treatment = PNS, Timepoint = d7:
##  contrast estimate    SE   df t.ratio p.value
##  M - F      0.0181 0.864 39.9   0.021  0.9834
##
## Treatment = Control, Timepoint = d21:
##  contrast estimate    SE   df t.ratio p.value
##  M - F      0.6937 1.100 46.0   0.633  0.5300
##
## Treatment = PNS, Timepoint = d21:
##  contrast estimate    SE   df t.ratio p.value
##  M - F      0.0828 0.788 40.3   0.105  0.9168
##
## Treatment = Control, Timepoint = d35:
##  contrast estimate    SE   df t.ratio p.value
##  M - F     -0.2868 1.080 38.9  -0.266  0.7920
##
## Treatment = PNS, Timepoint = d35:
##  contrast estimate    SE   df t.ratio p.value
##  M - F      2.1882 1.040 38.2   2.101  0.0423
##
## Degrees-of-freedom method: kenward-roger
```

Ileum Tissue *Il1b* Expression Sex Differences

```
# Does ileum Il1b expression differ by sex
#Include random intercept for litter
IL1b_sex_effects <- lmer(IL1b ~ Timepoint * Treatment * Sex + (1 | Litter), data = ileum_dat)
```

```
## boundary (singular) fit: see help('isSingular')
```

```
IL1b_anova_table_for_sex <- anova(IL1b_sex_effects, ddf = "Kenward-Roger")
print(IL1b_anova_table_for_sex)
```

```
## Type III Analysis of Variance Table with Kenward-Roger's method
##                         Sum Sq Mean Sq NumDF  DenDF F value    Pr(>F)
## Timepoint              26350.6 13175.3     2 45.289 20.9351 3.645e-07 ***
## Treatment                773.0   773.0     1 10.799  1.2282   0.29182
## Sex                     3847.7  3847.7     1 46.750  6.1139   0.01711 *
## Timepoint:Treatment     4901.8  2450.9     2 45.289  3.8944   0.02750 *
## Timepoint:Sex           5942.4  2971.2     2 44.977  4.7149   0.01384 *
## Treatment:Sex            433.6   433.6     1 46.750  0.6890   0.41073
## Timepoint:Treatment:Sex  880.6   440.3     2 44.977  0.6987   0.50257
## ---
## Signif. codes:  0 '***' 0.001 '**' 0.01 '*' 0.05 '.' 0.1 ' ' 1
```

```
IL1b_emcatcat_sex <- emmeans(IL1b_sex_effects, ~ Timepoint * Treatment * Sex)
#Is there a difference in males or females between treatment group at each time point?
contrast(IL1b_emcatcat_sex, "revpairwise", by=c("Sex", "Timepoint"), adjust="BH")
```

```
## Sex = F, Timepoint = d7:
##  contrast      estimate   SE   df t.ratio p.value
##  PNS - Control   -21.55 16.8 40.8  -1.285  0.2060
##
## Sex = M, Timepoint = d7:
##  contrast      estimate   SE   df t.ratio p.value
##  PNS - Control   -65.19 27.9 46.4  -2.332  0.0241
##
## Sex = F, Timepoint = d21:
##  contrast      estimate   SE   df t.ratio p.value
##  PNS - Control     4.96 15.1 36.6   0.328  0.7449
##
## Sex = M, Timepoint = d21:
##  contrast      estimate   SE   df t.ratio p.value
##  PNS - Control     6.19 15.9 35.2   0.389  0.6996
##
## Sex = F, Timepoint = d35:
##  contrast      estimate   SE   df t.ratio p.value
##  PNS - Control    10.10 20.8 45.5   0.486  0.6293
##
## Sex = M, Timepoint = d35:
##  contrast      estimate   SE   df t.ratio p.value
##  PNS - Control    11.51 21.2 45.7   0.544  0.5890
##
## Degrees-of-freedom method: kenward-roger
```

```
#Is there a difference in males or females within treatment group at each time point?
contrast(IL1b_emcatcat_sex, "revpairwise", by=c("Treatment", "Timepoint"), adjust="BH")
```

```
## Treatment = Control, Timepoint = d7:
##  contrast estimate   SE   df t.ratio p.value
##  M - F      81.133 28.3 47.0   2.863  0.0063
##
## Treatment = PNS, Timepoint = d7:
##  contrast estimate   SE   df t.ratio p.value
##  M - F      37.494 16.7 45.7   2.240  0.0300
##
## Treatment = Control, Timepoint = d21:
##  contrast estimate   SE   df t.ratio p.value
##  M - F       0.532 16.8 31.2   0.032  0.9749
##
## Treatment = PNS, Timepoint = d21:
##  contrast estimate   SE   df t.ratio p.value
##  M - F       1.768 15.0 46.9   0.118  0.9066
##
## Treatment = Control, Timepoint = d35:
##  contrast estimate   SE   df t.ratio p.value
##  M - F      -0.122 21.3 46.5  -0.006  0.9955
##
## Treatment = PNS, Timepoint = d35:
##  contrast estimate   SE   df t.ratio p.value
##  M - F       1.293 20.7 45.5   0.062  0.9506
##
## Degrees-of-freedom method: kenward-roger
```

Ileum Tissue *Il6* Expression Sex Differences

```
# Does ileum IL6 expression differ by sex
#Include random intercept for litter
IL6_sex_effects <- lmer(IL6 ~ Timepoint * Treatment * Sex + (1 | Litter), data = ileum_dat)
```

```
## fixed-effect model matrix is rank deficient so dropping 1 column / coefficient
```

```
IL6_anova_table_for_sex <- anova(IL6_sex_effects, ddf = "Kenward-Roger")
```

```
## Missing cells for: Timepointd7:TreatmentControl:SexM.
## Interpret type III hypotheses with care.
```

```
print(IL6_anova_table_for_sex)
```

```
## Type III Analysis of Variance Table with Kenward-Roger's method
##                        Sum Sq Mean Sq NumDF  DenDF F value  Pr(>F)
## Timepoint              2.1960  1.0980     2 41.524  0.9918 0.37952
## Treatment              4.1744  4.1744     1 13.070  3.7708 0.07402 .
## Sex                    0.2932  0.2932     1 45.490  0.2648 0.60931
## Timepoint:Treatment    2.5475  1.2738     2 42.498  1.1506 0.32612
## Timepoint:Sex          3.1534  1.5767     2 43.522  1.4241 0.25172
## Treatment:Sex          0.0006  0.0006     1 45.312  0.0005 0.98151
## Timepoint:Treatment:Sex 3.2600  3.2600     1 43.090  2.9448 0.09334 .
## ---
## Signif. codes:  0 '***' 0.001 '**' 0.01 '*' 0.05 '.' 0.1 ' ' 1
```

```
IL6_emcatcat_sex <- emmeans(IL6_sex_effects, ~ Timepoint * Treatment * Sex)
#Is there a difference in males or females between treatment group at each time point?
contrast(IL6_emcatcat_sex, "revpairwise", by=c("Sex", "Timepoint"), adjust="BH")
```

```
## Sex = F, Timepoint = d7:
##  contrast      estimate    SE   df t.ratio p.value
##  PNS - Control   0.0184 0.791 36.6   0.023  0.9815
##
## Sex = M, Timepoint = d7:
##  contrast      estimate    SE   df t.ratio p.value
##  PNS - Control   nonEst    NA   NA      NA      NA
##
## Sex = F, Timepoint = d21:
##  contrast      estimate    SE   df t.ratio p.value
##  PNS - Control   0.2561 0.742 30.6   0.345  0.7322
##
## Sex = M, Timepoint = d21:
##  contrast      estimate    SE   df t.ratio p.value
##  PNS - Control   1.8116 0.778 31.6   2.327  0.0265
##
## Sex = F, Timepoint = d35:
##  contrast      estimate    SE   df t.ratio p.value
##  PNS - Control   2.3981 0.949 43.4   2.527  0.0152
##
## Sex = M, Timepoint = d35:
##  contrast      estimate    SE   df t.ratio p.value
##  PNS - Control   0.8861 1.260 46.0   0.705  0.4846
##
## Degrees-of-freedom method: kenward-roger
```

```
#Is there a difference in males or females within treatment group at each time point?
contrast(IL6_emcatcat_sex, "revpairwise", by=c("Treatment", "Timepoint"), adjust="BH")
```

```
## Treatment = Control, Timepoint = d7:
##  contrast estimate    SE   df t.ratio p.value
##  M - F      nonEst    NA   NA      NA      NA
##
## Treatment = PNS, Timepoint = d7:
##  contrast estimate    SE   df t.ratio p.value
##  M - F      1.1947 0.722 43.7   1.654  0.1053
##
## Treatment = Control, Timepoint = d21:
##  contrast estimate    SE   df t.ratio p.value
##  M - F     -0.3137 0.808 42.2  -0.388  0.6999
##
## Treatment = PNS, Timepoint = d21:
##  contrast estimate    SE   df t.ratio p.value
##  M - F      1.2418 0.640 42.4   1.940  0.0591
##
## Treatment = Control, Timepoint = d35:
##  contrast estimate    SE   df t.ratio p.value
##  M - F      0.0159 0.907 41.4   0.018  0.9861
##
## Treatment = PNS, Timepoint = d35:
##  contrast estimate    SE   df t.ratio p.value
##  M - F     -1.4961 1.210 42.6  -1.239  0.2221
##
## Degrees-of-freedom method: kenward-roger
```

Ileum Tissue *Tnfa* Expression Sex Differences

```
# Does ileum Tnf expression differ by sex
#Include random intercept for litter
Tnf_sex_effects <- lmer(Tnf ~ Timepoint * Treatment * Sex + (1 | Litter), data = ileum_dat)
```

```
## fixed-effect model matrix is rank deficient so dropping 1 column / coefficient
```

```
Tnf_anova_table_for_sex <- anova(Tnf_sex_effects, ddf = "Kenward-Roger")
```

```
## Missing cells for: Timepointd7:TreatmentControl:SexM.
## Interpret type III hypotheses with care.
```

```
print(Tnf_anova_table_for_sex)
```

```
## Type III Analysis of Variance Table with Kenward-Roger's method
##                         Sum Sq Mean Sq NumDF  DenDF F value Pr(>F)
## Timepoint               6.0525  3.0262     2 41.089  1.3895 0.2607
## Treatment               2.0919  2.0919     1 12.634  0.9605 0.3455
## Sex                     0.5091  0.5091     1 44.958  0.2337 0.6311
## Timepoint:Treatment     3.7229  1.8615     2 41.934  0.8547 0.4327
## Timepoint:Sex           8.2109  4.1054     2 42.859  1.8849 0.1642
## Treatment:Sex           3.2319  3.2319     1 44.737  1.4839 0.2295
## Timepoint:Treatment:Sex 2.9410  2.9410     1 42.545  1.3504 0.2517
```

```
Tnf_emcatcat_sex <- emmeans(Tnf_sex_effects, ~ Timepoint * Treatment * Sex)
#Is there a difference in males or females between treatment group at each time point?
contrast(Tnf_emcatcat_sex, "revpairwise", by=c("Sex", "Timepoint"), adjust="BH")
```

```
## Sex = F, Timepoint = d7:
##  contrast      estimate   SE   df t.ratio p.value
##  PNS - Control  1.37786 1.14 34.9   1.206  0.2360
##
## Sex = M, Timepoint = d7:
##  contrast      estimate   SE   df t.ratio p.value
##  PNS - Control   nonEst   NA   NA      NA      NA
##
## Sex = F, Timepoint = d21:
##  contrast      estimate   SE   df t.ratio p.value
##  PNS - Control -0.01302 1.08 29.1  -0.012  0.9904
##
## Sex = M, Timepoint = d21:
##  contrast      estimate   SE   df t.ratio p.value
##  PNS - Control -0.15681 1.13 30.4  -0.139  0.8905
##
## Sex = F, Timepoint = d35:
##  contrast      estimate   SE   df t.ratio p.value
##  PNS - Control  3.06668 1.36 42.4   2.256  0.0293
##
## Sex = M, Timepoint = d35:
##  contrast      estimate   SE   df t.ratio p.value
##  PNS - Control -0.00385 1.79 45.9  -0.002  0.9983
##
## Degrees-of-freedom method: kenward-roger
```

```
#Is there a difference in males or females within treatment group at each time point?
contrast(Tnf_emcatcat_sex, "revpairwise", by=c("Treatment", "Timepoint"), adjust="BH")
```

```
## Treatment = Control, Timepoint = d7:
##  contrast estimate   SE   df t.ratio p.value
##  M - F      nonEst   NA   NA      NA      NA
##
## Treatment = PNS, Timepoint = d7:
##  contrast estimate   SE   df t.ratio p.value
##  M - F      0.9401 1.02 42.8   0.925  0.3599
##
## Treatment = Control, Timepoint = d21:
##  contrast estimate   SE   df t.ratio p.value
##  M - F     -0.1778 1.16 43.8  -0.154  0.8785
##
## Treatment = PNS, Timepoint = d21:
##  contrast estimate   SE   df t.ratio p.value
##  M - F     -0.3216 0.90 41.7  -0.357  0.7227
##
## Treatment = Control, Timepoint = d35:
##  contrast estimate   SE   df t.ratio p.value
##  M - F      0.0386 1.27 40.8   0.030  0.9760
##
## Treatment = PNS, Timepoint = d35:
##  contrast estimate   SE   df t.ratio p.value
##  M - F     -3.0319 1.70 41.8  -1.786  0.0813
##
## Degrees-of-freedom method: kenward-roger
```

Ileum Tissue *Ahr* Expression Sex Differences

```
# Does ileum Ahr expression differ by sex
#Include random intercept for litter
Ahr_sex_effects <- lmer(Ahr ~ Timepoint * Treatment * Sex + (1 | Litter), data = ileum_dat)
Ahr_anova_table_for_sex <- anova(Ahr_sex_effects, ddf = "Kenward-Roger")
print(Ahr_anova_table_for_sex)
```

```
## Type III Analysis of Variance Table with Kenward-Roger's method
##                         Sum Sq Mean Sq NumDF  DenDF F value    Pr(>F)
## Timepoint              17.8567  8.9284     2 41.260 15.0782 1.215e-05 ***
## Treatment               0.4587  0.4587     1 10.224  0.7746    0.3990
## Sex                     2.2040  2.2040     1 41.804  3.7220    0.0605 .
## Timepoint:Treatment    14.7142  7.3571     2 41.260 12.4247 5.975e-05 ***
## Timepoint:Sex           0.0813  0.0406     2 43.996  0.0686    0.9338
## Treatment:Sex           0.2717  0.2717     1 41.804  0.4589    0.5019
## Timepoint:Treatment:Sex  2.1318  1.0659     2 43.996  1.7994    0.1774
## ---
## Signif. codes:  0 '***' 0.001 '**' 0.01 '*' 0.05 '.' 0.1 ' ' 1
```

```
Ahr_emcatcat_sex <- emmeans(Ahr_sex_effects, ~ Timepoint * Treatment * Sex)
#Is there a difference in males or females between treatment group at each time point?
contrast(Ahr_emcatcat_sex, "revpairwise", by=c("Sex", "Timepoint"), adjust="BH")
```

```
## Sex = F, Timepoint = d7:
##  contrast      estimate    SE   df t.ratio p.value
##  PNS - Control   -1.028 0.614 33.4  -1.674  0.1035
##
## Sex = M, Timepoint = d7:
##  contrast      estimate    SE   df t.ratio p.value
##  PNS - Control   -1.818 0.933 46.7  -1.950  0.0573
##
## Sex = F, Timepoint = d21:
##  contrast      estimate    SE   df t.ratio p.value
##  PNS - Control    0.465 0.583 27.9   0.799  0.4312
##
## Sex = M, Timepoint = d21:
##  contrast      estimate    SE   df t.ratio p.value
##  PNS - Control    0.567 0.610 29.5   0.930  0.3599
##
## Sex = F, Timepoint = d35:
##  contrast      estimate    SE   df t.ratio p.value
##  PNS - Control    1.104 0.724 41.9   1.524  0.1351
##
## Sex = M, Timepoint = d35:
##  contrast      estimate    SE   df t.ratio p.value
##  PNS - Control    2.840 0.735 42.3   3.862  0.0004
##
## Degrees-of-freedom method: kenward-roger
```

```
#Is there a difference in males or females within treatment group at each time point?
contrast(Ahr_emcatcat_sex, "revpairwise", by=c("Treatment", "Timepoint"), adjust="BH")
```

```
## Treatment = Control, Timepoint = d7:
##  contrast estimate    SE   df t.ratio p.value
##  M - F       0.945 0.894 42.3   1.057  0.2964
##
## Treatment = PNS, Timepoint = d7:
##  contrast estimate    SE   df t.ratio p.value
##  M - F       0.155 0.531 43.1   0.293  0.7710
##
## Treatment = Control, Timepoint = d21:
##  contrast estimate    SE   df t.ratio p.value
##  M - F       0.526 0.613 45.8   0.857  0.3960
##
## Treatment = PNS, Timepoint = d21:
##  contrast estimate    SE   df t.ratio p.value
##  M - F       0.627 0.469 41.9   1.336  0.1887
##
## Treatment = Control, Timepoint = d35:
##  contrast estimate    SE   df t.ratio p.value
##  M - F      -0.502 0.665 41.3  -0.755  0.4548
##
## Treatment = PNS, Timepoint = d35:
##  contrast estimate    SE   df t.ratio p.value
##  M - F       1.235 0.644 40.5   1.916  0.0624
##
## Degrees-of-freedom method: kenward-roger
```

Ileum Tissue *Muc2* Expression Sex Differences

```
# Does ileum Muc2 expression differ by sex
#Include random intercept for litter
Muc2_sex_effects <- lmer(Muc2 ~ Timepoint * Treatment * Sex + (1 | Litter), data = ileum_dat)
Muc2_anova_table_for_sex <- anova(Muc2_sex_effects, ddf = "Kenward-Roger")
print(Muc2_anova_table_for_sex)
```

```
## Type III Analysis of Variance Table with Kenward-Roger's method
##                         Sum Sq Mean Sq NumDF  DenDF F value    Pr(>F)
## Timepoint              1181.91  590.96     2 41.863 39.4742 2.321e-10 ***
## Treatment                71.24   71.24     1 11.728  4.7590   0.05026 .
## Sex                      56.61   56.61     1 43.467  3.7813   0.05832 .
## Timepoint:Treatment      17.67    8.84     2 41.863  0.5903   0.55870
## Timepoint:Sex            44.44   22.22     2 44.836  1.4837   0.23773
## Treatment:Sex            25.21   25.21     1 43.467  1.6841   0.20122
## Timepoint:Treatment:Sex   13.65    6.83     2 44.836  0.4558   0.63685
## ---
## Signif. codes:  0 '***' 0.001 '**' 0.01 '*' 0.05 '.' 0.1 ' ' 1
```

```
Muc2_emcatcat_sex <- emmeans(Muc2_sex_effects, ~ Timepoint * Treatment * Sex)
#Is there a difference in males or females between treatment group at each time point?
contrast(Muc2_emcatcat_sex, "revpairwise", by=c("Sex", "Timepoint"), adjust="BH")
```

```
## Sex = F, Timepoint = d7:
##  contrast      estimate   SE   df t.ratio p.value
##  PNS - Control     2.04 2.96 36.2   0.687  0.4963
##
## Sex = M, Timepoint = d7:
##  contrast      estimate   SE   df t.ratio p.value
##  PNS - Control     2.40 4.60 46.9   0.521  0.6050
##
## Sex = F, Timepoint = d21:
##  contrast      estimate   SE   df t.ratio p.value
##  PNS - Control     8.40 2.79 30.2   3.013  0.0052
##
## Sex = M, Timepoint = d21:
##  contrast      estimate   SE   df t.ratio p.value
##  PNS - Control     2.65 2.89 30.5   0.918  0.3658
##
## Sex = F, Timepoint = d35:
##  contrast      estimate   SE   df t.ratio p.value
##  PNS - Control     7.46 3.53 43.7   2.112  0.0405
##
## Sex = M, Timepoint = d35:
##  contrast      estimate   SE   df t.ratio p.value
##  PNS - Control     1.95 4.66 46.9   0.418  0.6776
##
## Degrees-of-freedom method: kenward-roger
```

```
#Is there a difference in males or females within treatment group at each time point?
contrast(Muc2_emcatcat_sex, "revpairwise", by=c("Treatment", "Timepoint"), adjust="BH")
```

```
## Treatment = Control, Timepoint = d7:
##  contrast estimate   SE   df t.ratio p.value
##  M - F       0.328 4.48 43.1   0.073  0.9419
##
## Treatment = PNS, Timepoint = d7:
##  contrast estimate   SE   df t.ratio p.value
##  M - F       0.688 2.66 44.1   0.259  0.7971
##
## Treatment = Control, Timepoint = d21:
##  contrast estimate   SE   df t.ratio p.value
##  M - F       0.271 3.01 44.1   0.090  0.9286
##
## Treatment = PNS, Timepoint = d21:
##  contrast estimate   SE   df t.ratio p.value
##  M - F      -5.477 2.33 43.7  -2.351  0.0233
##
## Treatment = Control, Timepoint = d35:
##  contrast estimate   SE   df t.ratio p.value
##  M - F      -3.316 3.34 42.0  -0.993  0.3262
##
## Treatment = PNS, Timepoint = d35:
##  contrast estimate   SE   df t.ratio p.value
##  M - F      -8.830 4.44 43.1  -1.987  0.0533
##
## Degrees-of-freedom method: kenward-roger
```

Ileum Tissue *Il10* Expression Sex Differences

```
# Does ileum IL10 expression differ by sex
#Include random intercept for litter
IL10_sex_effects <- lmer(IL10 ~ Timepoint * Treatment * Sex + (1 | Litter), data = ileum_dat)
IL10_anova_table_for_sex <- anova(IL10_sex_effects, ddf = "Kenward-Roger")
print(IL10_anova_table_for_sex)
```

```
## Type III Analysis of Variance Table with Kenward-Roger's method
##                         Sum Sq Mean Sq NumDF  DenDF F value    Pr(>F)
## Timepoint               3742.6 1871.30     2 43.007 11.2994 0.0001138 ***
## Treatment               1069.8 1069.82     1 10.631  6.4599 0.0280485 *
## Sex                       31.0   31.00     1 44.245  0.1872 0.6673731
## Timepoint:Treatment     2526.0 1263.01     2 43.007  7.6264 0.0014629 **
## Timepoint:Sex             10.3    5.17     2 44.756  0.0312 0.9692910
## Treatment:Sex              0.0    0.02     1 44.245  0.0001 0.9914083
## Timepoint:Treatment:Sex    2.7    1.34     2 44.756  0.0081 0.9919463
## ---
## Signif. codes:  0 '***' 0.001 '**' 0.01 '*' 0.05 '.' 0.1 ' ' 1
```

```
IL10_emcatcat_sex <- emmeans(IL10_sex_effects, ~ Timepoint * Treatment * Sex)
#Is there a difference in males or females between treatment group at each time point?
contrast(IL10_emcatcat_sex, "revpairwise", by=c("Sex", "Timepoint"), adjust="BH")
```

```
## Sex = F, Timepoint = d7:
##  contrast      estimate    SE   df t.ratio p.value
##  PNS - Control   -0.386  9.02 39.5  -0.043  0.9660
##
## Sex = M, Timepoint = d7:
##  contrast      estimate    SE   df t.ratio p.value
##  PNS - Control   -1.172 14.70 45.9  -0.080  0.9368
##
## Sex = F, Timepoint = d21:
##  contrast      estimate    SE   df t.ratio p.value
##  PNS - Control    0.886  8.26 34.1   0.107  0.9152
##
## Sex = M, Timepoint = d21:
##  contrast      estimate    SE   df t.ratio p.value
##  PNS - Control    2.215  8.71 33.9   0.254  0.8008
##
## Sex = F, Timepoint = d35:
##  contrast      estimate    SE   df t.ratio p.value
##  PNS - Control   36.400 11.20 44.9   3.243  0.0022
##
## Sex = M, Timepoint = d35:
##  contrast      estimate    SE   df t.ratio p.value
##  PNS - Control   35.579 11.20 44.8   3.171  0.0027
##
## Degrees-of-freedom method: kenward-roger
```

```
#Is there a difference in males or females within treatment group at each time point?
contrast(IL10_emcatcat_sex, "revpairwise", by=c("Treatment", "Timepoint"), adjust="BH")
```

```
## Treatment = Control, Timepoint = d7:
##  contrast estimate    SE   df t.ratio p.value
##  M - F       0.991 14.70 44.8   0.067  0.9466
##
## Treatment = PNS, Timepoint = d7:
##  contrast estimate    SE   df t.ratio p.value
##  M - F       0.206  8.75 46.0   0.024  0.9813
##
## Treatment = Control, Timepoint = d21:
##  contrast estimate    SE   df t.ratio p.value
##  M - F       0.927  9.21 36.2   0.101  0.9204
##
## Treatment = PNS, Timepoint = d21:
##  contrast estimate    SE   df t.ratio p.value
##  M - F       2.256  7.83 45.5   0.288  0.7746
##
## Treatment = Control, Timepoint = d35:
##  contrast estimate    SE   df t.ratio p.value
##  M - F       3.781 11.00 43.7   0.343  0.7333
##
## Treatment = PNS, Timepoint = d35:
##  contrast estimate    SE   df t.ratio p.value
##  M - F       2.959 10.80 41.8   0.273  0.7861
##
## Degrees-of-freedom method: kenward-roger
```

Ileum Tissue *Tgfb1* Expression Sex Differences

```
# Does ileum Tgfb1 expression differ by sex
#Include random intercept for litter
Tgfb1_sex_effects <- lmer(Tgfb1 ~ Timepoint * Treatment * Sex + (1 | Litter), data = ileum_dat)
Tgfb1_anova_table_for_sex <- anova(Tgfb1_sex_effects, ddf = "Kenward-Roger")
print(Tgfb1_anova_table_for_sex)
```

```
## Type III Analysis of Variance Table with Kenward-Roger's method
##                         Sum Sq Mean Sq NumDF  DenDF F value    Pr(>F)
## Timepoint              14.6666  7.3333     2 40.718 29.3818 1.263e-08 ***
## Treatment               0.0153  0.0153     1  9.762  0.0614   0.80946
## Sex                     0.3914  0.3914     1 41.152  1.5682   0.21754
## Timepoint:Treatment     0.9483  0.4742     2 40.718  1.8998   0.16263
## Timepoint:Sex           1.2240  0.6120     2 42.848  2.4516   0.09816 .
## Treatment:Sex           0.0747  0.0747     1 41.152  0.2994   0.58722
## Timepoint:Treatment:Sex  0.0374  0.0187     2 42.848  0.0750   0.92788
## ---
## Signif. codes:  0 '***' 0.001 '**' 0.01 '*' 0.05 '.' 0.1 ' ' 1
```

```
Tgfb1_emcatcat_sex <- emmeans(Tgfb1_sex_effects, ~ Timepoint * Treatment * Sex)
#Is there a difference in males or females between treatment group at each time point?
contrast(Tgfb1_emcatcat_sex, "revpairwise", by=c("Sex", "Timepoint"), adjust="BH")
```

```
## Sex = F, Timepoint = d7:
##  contrast      estimate    SE   df t.ratio p.value
##  PNS - Control  -0.4079 0.473 23.9  -0.862  0.3972
##
## Sex = M, Timepoint = d7:
##  contrast      estimate    SE   df t.ratio p.value
##  PNS - Control  -0.7514 0.658 43.6  -1.142  0.2596
##
## Sex = F, Timepoint = d21:
##  contrast      estimate    SE   df t.ratio p.value
##  PNS - Control   0.1166 0.458 20.8   0.254  0.8016
##
## Sex = M, Timepoint = d21:
##  contrast      estimate    SE   df t.ratio p.value
##  PNS - Control   0.1088 0.471 22.0   0.231  0.8193
##
## Sex = F, Timepoint = d35:
##  contrast      estimate    SE   df t.ratio p.value
##  PNS - Control   0.2983 0.535 32.5   0.558  0.5808
##
## Sex = M, Timepoint = d35:
##  contrast      estimate    SE   df t.ratio p.value
##  PNS - Control   0.0967 0.541 33.2   0.179  0.8593
##
## Degrees-of-freedom method: kenward-roger
```

```
#Is there a difference in males or females within treatment group at each time point?
contrast(Tgfb1_emcatcat_sex, "revpairwise", by=c("Treatment", "Timepoint"), adjust="BH")
```

```
## Treatment = Control, Timepoint = d7:
##  contrast estimate    SE   df t.ratio p.value
##  M - F      0.9567 0.585 41.3   1.637  0.1093
##
## Treatment = PNS, Timepoint = d7:
##  contrast estimate    SE   df t.ratio p.value
##  M - F      0.6132 0.347 41.6   1.768  0.0843
##
## Treatment = Control, Timepoint = d21:
##  contrast estimate    SE   df t.ratio p.value
##  M - F      0.0781 0.421 47.6   0.185  0.8537
##
## Treatment = PNS, Timepoint = d21:
##  contrast estimate    SE   df t.ratio p.value
##  M - F      0.0703 0.304 41.4   0.231  0.8181
##
## Treatment = Control, Timepoint = d35:
##  contrast estimate    SE   df t.ratio p.value
##  M - F     -0.1256 0.434 40.6  -0.290  0.7736
##
## Treatment = PNS, Timepoint = d35:
##  contrast estimate    SE   df t.ratio p.value
##  M - F     -0.3272 0.419 40.2  -0.780  0.4399
##
## Degrees-of-freedom method: kenward-roger
```

Ileum Tissue *Foxp3* Expression Sex Differences

```
# Does ileum Foxp3 expression differ by sex
#Include random intercept for litter
Foxp3_sex_effects <- lmer(Foxp3 ~ Timepoint * Treatment * Sex + (1 | Litter), data = ileum_dat)
Foxp3_anova_table_for_sex <- anova(Foxp3_sex_effects, ddf = "Kenward-Roger")
print(Foxp3_anova_table_for_sex)
```

```
## Type III Analysis of Variance Table with Kenward-Roger's method
##                         Sum Sq Mean Sq NumDF  DenDF F value    Pr(>F)
## Timepoint              11092.9  5546.5     2 38.912  7.2931 0.0020422 **
## Treatment                184.5   184.5     1  9.980  0.2425 0.6330267
## Sex                     1485.2  1485.2     1 39.209  1.9529 0.1701317
## Timepoint:Treatment    15221.1  7610.6     2 38.912 10.0072 0.0003115 ***
## Timepoint:Sex           2154.3  1077.1     2 41.477  1.4160 0.2541709
## Treatment:Sex             44.2    44.2     1 39.209  0.0581 0.8107800
## Timepoint:Treatment:Sex  1646.0   823.0     2 41.477  1.0819 0.3483220
## ---
## Signif. codes:  0 '***' 0.001 '**' 0.01 '*' 0.05 '.' 0.1 ' ' 1
```

```
Foxp3_emcatcat_sex <- emmeans(Foxp3_sex_effects, ~ Timepoint * Treatment * Sex)
#Is there a difference in males or females between treatment group at each time point?
contrast(Foxp3_emcatcat_sex, "revpairwise", by=c("Sex", "Timepoint"), adjust="BH")
```

```
## Sex = F, Timepoint = d7:
##  contrast      estimate   SE   df t.ratio p.value
##  PNS - Control     9.79 24.6 26.4   0.398  0.6939
##
## Sex = M, Timepoint = d7:
##  contrast      estimate   SE   df t.ratio p.value
##  PNS - Control    -8.30 35.3 44.1  -0.235  0.8150
##
## Sex = F, Timepoint = d21:
##  contrast      estimate   SE   df t.ratio p.value
##  PNS - Control   -44.90 23.7 22.6  -1.892  0.0714
##
## Sex = M, Timepoint = d21:
##  contrast      estimate   SE   df t.ratio p.value
##  PNS - Control   -74.01 25.8 27.6  -2.869  0.0078
##
## Sex = F, Timepoint = d35:
##  contrast      estimate   SE   df t.ratio p.value
##  PNS - Control    15.05 28.3 35.3   0.533  0.5977
##
## Sex = M, Timepoint = d35:
##  contrast      estimate   SE   df t.ratio p.value
##  PNS - Control    48.75 29.0 37.0   1.683  0.1007
##
## Degrees-of-freedom method: kenward-roger
```

```
#Is there a difference in males or females within treatment group at each time point?
contrast(Foxp3_emcatcat_sex, "revpairwise", by=c("Treatment", "Timepoint"), adjust="BH")
```

```
## Treatment = Control, Timepoint = d7:
##  contrast estimate   SE   df t.ratio p.value
##  M - F       26.76 32.2 39.8   0.830  0.4115
##
## Treatment = PNS, Timepoint = d7:
##  contrast estimate   SE   df t.ratio p.value
##  M - F        8.67 19.1 40.1   0.454  0.6525
##
## Treatment = Control, Timepoint = d21:
##  contrast estimate   SE   df t.ratio p.value
##  M - F       43.79 24.5 45.8   1.786  0.0807
##
## Treatment = PNS, Timepoint = d21:
##  contrast estimate   SE   df t.ratio p.value
##  M - F       14.67 16.7 39.9   0.877  0.3859
##
## Treatment = Control, Timepoint = d35:
##  contrast estimate   SE   df t.ratio p.value
##  M - F      -24.66 24.3 38.7  -1.016  0.3162
##
## Treatment = PNS, Timepoint = d35:
##  contrast estimate   SE   df t.ratio p.value
##  M - F        9.04 23.1 38.4   0.391  0.6981
##
## Degrees-of-freedom method: kenward-roger
```

Ileum Tissue *Il18* Expression Sex Differences

```
# Does ileum IL18 expression differ by sex
#Include random intercept for litter
IL18_sex_effects <- lmer(IL18 ~ Timepoint * Treatment * Sex + (1 | Litter), data = ileum_dat)
IL18_anova_table_for_sex <- anova(IL18_sex_effects, ddf = "Kenward-Roger")
print(IL18_anova_table_for_sex)
```

```
## Type III Analysis of Variance Table with Kenward-Roger's method
##                        Sum Sq Mean Sq NumDF  DenDF F value    Pr(>F)
## Timepoint              43.256 21.6279     2 40.749 14.3517 1.914e-05 ***
## Treatment               4.423  4.4229     1 10.241  2.9350 0.1167373
## Sex                     4.583  4.5826     1 40.217  3.0410 0.0888273 .
## Timepoint:Treatment    29.486 14.7430     2 40.749  9.7830 0.0003389 ***
## Timepoint:Sex           6.705  3.3526     2 43.036  2.2229 0.1205813
## Treatment:Sex           1.162  1.1620     1 40.217  0.7711 0.3850984
## Timepoint:Treatment:Sex  8.616  4.3082     2 43.036  2.8565 0.0684138 .
## ---
## Signif. codes:  0 '***' 0.001 '**' 0.01 '*' 0.05 '.' 0.1 ' ' 1
```

```
IL18_emcatcat_sex <- emmeans(IL18_sex_effects, ~ Timepoint * Treatment * Sex)
#Is there a difference in males or females between treatment group at each time point?
contrast(IL18_emcatcat_sex, "revpairwise", by=c("Sex", "Timepoint"), adjust="BH")
```

```
## Sex = F, Timepoint = d7:
##  contrast      estimate    SE   df t.ratio p.value
##  PNS - Control   -0.993 0.932 35.1  -1.065  0.2940
##
## Sex = M, Timepoint = d7:
##  contrast      estimate    SE   df t.ratio p.value
##  PNS - Control   -1.793 1.450 45.0  -1.233  0.2238
##
## Sex = F, Timepoint = d21:
##  contrast      estimate    SE   df t.ratio p.value
##  PNS - Control    1.147 0.871 29.7   1.317  0.1979
##
## Sex = M, Timepoint = d21:
##  contrast      estimate    SE   df t.ratio p.value
##  PNS - Control    0.554 0.923 30.0   0.601  0.5527
##
## Sex = F, Timepoint = d35:
##  contrast      estimate    SE   df t.ratio p.value
##  PNS - Control    1.684 1.130 42.6   1.489  0.1438
##
## Sex = M, Timepoint = d35:
##  contrast      estimate    SE   df t.ratio p.value
##  PNS - Control    5.236 1.150 43.1   4.541  <.0001
##
## Degrees-of-freedom method: kenward-roger
```

```
#Is there a difference in males or females within treatment group at each time point?
contrast(IL18_emcatcat_sex, "revpairwise", by=c("Treatment", "Timepoint"), adjust="BH")
```

```
## Treatment = Control, Timepoint = d7:
##  contrast estimate    SE   df t.ratio p.value
##  M - F      0.9577 1.420 41.4   0.674  0.5040
##
## Treatment = PNS, Timepoint = d7:
##  contrast estimate    SE   df t.ratio p.value
##  M - F      0.1582 0.851 43.4   0.186  0.8534
##
## Treatment = Control, Timepoint = d21:
##  contrast estimate    SE   df t.ratio p.value
##  M - F      0.0809 0.952 40.7   0.085  0.9328
##
## Treatment = PNS, Timepoint = d21:
##  contrast estimate    SE   df t.ratio p.value
##  M - F     -0.5123 0.768 43.1  -0.667  0.5081
##
## Treatment = Control, Timepoint = d35:
##  contrast estimate    SE   df t.ratio p.value
##  M - F      0.0258 1.070 39.5   0.024  0.9810
##
## Treatment = PNS, Timepoint = d35:
##  contrast estimate    SE   df t.ratio p.value
##  M - F      3.5776 1.040 38.8   3.448  0.0014
##
## Degrees-of-freedom method: kenward-roger
```

Ileum Tissue *Il4* Expression Sex Differences

```
# Does ileum IL4 expression differ by sex
#Include random intercept for litter
IL4_sex_effects <- lmer(IL4 ~ Timepoint * Treatment * Sex + (1 | Litter), data = ileum_dat)
IL4_anova_table_for_sex <- anova(IL4_sex_effects, ddf = "Kenward-Roger")
print(IL4_anova_table_for_sex)
```

```
## Type III Analysis of Variance Table with Kenward-Roger's method
##                         Sum Sq Mean Sq NumDF  DenDF F value  Pr(>F)
## Timepoint              11105.6  5552.8     2 41.950  3.3055 0.04644 *
## Treatment                777.4   777.4     1 10.452  0.4628 0.51111
## Sex                     1287.2  1287.2     1 41.639  0.7663 0.38639
## Timepoint:Treatment     4993.8  2496.9     2 41.950  1.4864 0.23786
## Timepoint:Sex          10121.4  5060.7     2 43.591  3.0091 0.05968 .
## Treatment:Sex           3834.0  3834.0     1 41.639  2.2825 0.13840
## Timepoint:Treatment:Sex   163.9    82.0     2 43.591  0.0487 0.95249
## ---
## Signif. codes:  0 '***' 0.001 '**' 0.01 '*' 0.05 '.' 0.1 ' ' 1
```

```
IL4_emcatcat_sex <- emmeans(IL4_sex_effects, ~ Timepoint * Treatment * Sex)
#Is there a difference in males or females between treatment group at each time point?
contrast(IL4_emcatcat_sex, "revpairwise", by=c("Sex", "Timepoint"), adjust="BH")
```

```
## Sex = F, Timepoint = d7:
##  contrast      estimate   SE   df t.ratio p.value
##  PNS - Control  -15.644 29.1 38.4  -0.537  0.5944
##
## Sex = M, Timepoint = d7:
##  contrast      estimate   SE   df t.ratio p.value
##  PNS - Control  -67.187 47.2 45.0  -1.425  0.1611
##
## Sex = F, Timepoint = d21:
##  contrast      estimate   SE   df t.ratio p.value
##  PNS - Control    2.829 26.8 32.7   0.106  0.9166
##
## Sex = M, Timepoint = d21:
##  contrast      estimate   SE   df t.ratio p.value
##  PNS - Control  -27.509 28.2 32.4  -0.974  0.3373
##
## Sex = F, Timepoint = d35:
##  contrast      estimate   SE   df t.ratio p.value
##  PNS - Control   41.584 36.1 43.9   1.152  0.2555
##
## Sex = M, Timepoint = d35:
##  contrast      estimate   SE   df t.ratio p.value
##  PNS - Control    0.401 36.9 44.1   0.011  0.9914
##
## Degrees-of-freedom method: kenward-roger
```

```
#Is there a difference in males or females between within group at each time point?
contrast(IL4_emcatcat_sex, "revpairwise", by=c("Treatment", "Timepoint"), adjust="BH")
```

```
## Treatment = Control, Timepoint = d7:
##  contrast estimate   SE   df t.ratio p.value
##  M - F       85.17 47.0 43.3   1.811  0.0771
##
## Treatment = PNS, Timepoint = d7:
##  contrast estimate   SE   df t.ratio p.value
##  M - F       33.63 28.0 44.7   1.202  0.2357
##
## Treatment = Control, Timepoint = d21:
##  contrast estimate   SE   df t.ratio p.value
##  M - F       23.11 29.8 35.9   0.774  0.4438
##
## Treatment = PNS, Timepoint = d21:
##  contrast estimate   SE   df t.ratio p.value
##  M - F       -7.23 24.8 43.6  -0.292  0.7720
##
## Treatment = Control, Timepoint = d35:
##  contrast estimate   SE   df t.ratio p.value
##  M - F      -11.10 35.7 41.2  -0.311  0.7577
##
## Treatment = PNS, Timepoint = d35:
##  contrast estimate   SE   df t.ratio p.value
##  M - F      -52.28 34.6 40.4  -1.513  0.1381
##
## Degrees-of-freedom method: kenward-roger
```

Ileum Tissue *Il5* Expression Sex Differences

```
# Does ileum IL5 expression differ by sex
#Include random intercept for litter
IL5_sex_effects <- lmer(IL5 ~ Timepoint * Treatment * Sex + (1 | Litter), data = ileum_dat)
IL5_anova_table_for_sex <- anova(IL5_sex_effects, ddf = "Kenward-Roger")
print(IL5_anova_table_for_sex)
```

```
## Type III Analysis of Variance Table with Kenward-Roger's method
##                          Sum Sq  Mean Sq NumDF  DenDF F value Pr(>F)
## Timepoint              0.131467 0.065734     2 37.540  1.2480 0.2987
## Treatment              0.050580 0.050580     1  9.555  0.9603 0.3513
## Sex                    0.040349 0.040349     1 38.018  0.7661 0.3869
## Timepoint:Treatment    0.247933 0.123966     2 37.540  2.3536 0.1089
## Timepoint:Sex          0.000101 0.000050     2 39.150  0.0010 0.9990
## Treatment:Sex          0.025796 0.025796     1 38.018  0.4898 0.4883
## Timepoint:Treatment:Sex 0.029341 0.014671     2 39.150  0.2785 0.7584
```

```
IL5_emcatcat_sex <- emmeans(IL5_sex_effects, ~ Timepoint * Treatment * Sex)
#Is there a difference in males or females between treatment group at each time point?
contrast(IL5_emcatcat_sex, "revpairwise", by=c("Sex", "Timepoint"), adjust="BH")
```

```
## Sex = F, Timepoint = d7:
##  contrast      estimate    SE   df t.ratio p.value
##  PNS - Control   0.3385 0.229 21.2   1.477  0.1544
##
## Sex = M, Timepoint = d7:
##  contrast      estimate    SE   df t.ratio p.value
##  PNS - Control   0.2025 0.314 40.0   0.645  0.5226
##
## Sex = F, Timepoint = d21:
##  contrast      estimate    SE   df t.ratio p.value
##  PNS - Control   0.3003 0.223 18.8   1.348  0.1936
##
## Sex = M, Timepoint = d21:
##  contrast      estimate    SE   df t.ratio p.value
##  PNS - Control   0.3359 0.241 23.3   1.393  0.1767
##
## Sex = F, Timepoint = d35:
##  contrast      estimate    SE   df t.ratio p.value
##  PNS - Control   0.0628 0.257 28.7   0.245  0.8085
##
## Sex = M, Timepoint = d35:
##  contrast      estimate    SE   df t.ratio p.value
##  PNS - Control  -0.1717 0.259 29.4  -0.662  0.5134
##
## Degrees-of-freedom method: kenward-roger
```

```
#Is there a difference in males or females within treatment group at each time point?
contrast(IL5_emcatcat_sex, "revpairwise", by=c("Treatment", "Timepoint"), adjust="BH")
```

```
## Treatment = Control, Timepoint = d7:
##  contrast estimate    SE   df t.ratio p.value
##  M - F    -0.00374 0.269 37.8  -0.014  0.9890
##
## Treatment = PNS, Timepoint = d7:
##  contrast estimate    SE   df t.ratio p.value
##  M - F    -0.13973 0.169 38.9  -0.824  0.4147
##
## Treatment = Control, Timepoint = d21:
##  contrast estimate    SE   df t.ratio p.value
##  M - F    -0.09036 0.209 43.5  -0.432  0.6678
##
## Treatment = PNS, Timepoint = d21:
##  contrast estimate    SE   df t.ratio p.value
##  M - F    -0.05472 0.141 37.6  -0.388  0.7002
##
## Treatment = Control, Timepoint = d35:
##  contrast estimate    SE   df t.ratio p.value
##  M - F     0.05214 0.200 37.4   0.260  0.7960
##
## Treatment = PNS, Timepoint = d35:
##  contrast estimate    SE   df t.ratio p.value
##  M - F    -0.18228 0.193 36.9  -0.945  0.3507
##
## Degrees-of-freedom method: kenward-roger
```

Ileum Tissue *Il13* Expression Sex Differences

```
# Does ileum IL13 expression differ by sex
#Include random intercept for litter
IL13_sex_effects <- lmer(IL13 ~ Timepoint * Treatment * Sex + (1 | Litter), data = ileum_dat)
IL13_anova_table_for_sex <- anova(IL13_sex_effects, ddf = "Kenward-Roger")
print(IL13_anova_table_for_sex)
```

```
## Type III Analysis of Variance Table with Kenward-Roger's method
##                         Sum Sq Mean Sq NumDF  DenDF F value Pr(>F)
## Timepoint              0.06002 0.03001     2 40.785  0.2405 0.7874
## Treatment              0.73222 0.73222     1  9.923  5.8676 0.0361 *
## Sex                    0.11371 0.11371     1 42.548  0.9112 0.3452
## Timepoint:Treatment    0.39652 0.19826     2 40.785  1.5887 0.2166
## Timepoint:Sex          0.09337 0.04668     2 42.545  0.3736 0.6905
## Treatment:Sex          0.00111 0.00111     1 42.548  0.0089 0.9252
## Timepoint:Treatment:Sex 0.17525 0.08762     2 42.545  0.7012 0.5016
## ---
## Signif. codes:  0 '***' 0.001 '**' 0.01 '*' 0.05 '.' 0.1 ' ' 1
```

```
IL13_emcatcat_sex <- emmeans(IL13_sex_effects, ~ Timepoint * Treatment * Sex)
#Is there a difference in males or females between treatment group at each time point?
contrast(IL13_emcatcat_sex, "revpairwise", by=c("Sex", "Timepoint"), adjust="BH")
```

```
## Sex = F, Timepoint = d7:
##  contrast      estimate    SE   df t.ratio p.value
##  PNS - Control    0.729 0.246 38.0   2.965  0.0052
##
## Sex = M, Timepoint = d7:
##  contrast      estimate    SE   df t.ratio p.value
##  PNS - Control    0.371 0.407 43.8   0.911  0.3675
##
## Sex = F, Timepoint = d21:
##  contrast      estimate    SE   df t.ratio p.value
##  PNS - Control    0.367 0.231 35.5   1.588  0.1211
##
## Sex = M, Timepoint = d21:
##  contrast      estimate    SE   df t.ratio p.value
##  PNS - Control    0.395 0.237 32.4   1.667  0.1052
##
## Sex = F, Timepoint = d35:
##  contrast      estimate    SE   df t.ratio p.value
##  PNS - Control   -0.178 0.301 42.7  -0.590  0.5584
##
## Sex = M, Timepoint = d35:
##  contrast      estimate    SE   df t.ratio p.value
##  PNS - Control    0.219 0.313 43.2   0.700  0.4875
##
## Degrees-of-freedom method: kenward-roger
```

```
#Is there a difference in males or females within treatment group at each time point?
contrast(IL13_emcatcat_sex, "revpairwise", by=c("Treatment", "Timepoint"), adjust="BH")
```

```
## Treatment = Control, Timepoint = d7:
##  contrast estimate    SE   df t.ratio p.value
##  M - F     -0.0841 0.404 43.1  -0.208  0.8362
##
## Treatment = PNS, Timepoint = d7:
##  contrast estimate    SE   df t.ratio p.value
##  M - F     -0.4418 0.250 43.8  -1.765  0.0846
##
## Treatment = Control, Timepoint = d21:
##  contrast estimate    SE   df t.ratio p.value
##  M - F     -0.1045 0.257 35.8  -0.407  0.6865
##
## Treatment = PNS, Timepoint = d21:
##  contrast estimate    SE   df t.ratio p.value
##  M - F     -0.0766 0.213 42.8  -0.360  0.7206
##
## Treatment = Control, Timepoint = d35:
##  contrast estimate    SE   df t.ratio p.value
##  M - F     -0.1847 0.308 41.2  -0.601  0.5514
##
## Treatment = PNS, Timepoint = d35:
##  contrast estimate    SE   df t.ratio p.value
##  M - F      0.2123 0.294 40.9   0.721  0.4749
##
## Degrees-of-freedom method: kenward-roger
```

Ileum Tissue *Il17a* Expression Sex Differences

```
# Does ileum IL17 expression differ by sex
#Include random intercept for litter
IL17_sex_effects <- lmer(IL17 ~ Timepoint * Treatment * Sex + (1 | Litter), data = ileum_dat)
```

```
## fixed-effect model matrix is rank deficient so dropping 1 column / coefficient
```

```
IL17_anova_table_for_sex <- anova(IL17_sex_effects, ddf = "Kenward-Roger")
```

```
## Missing cells for: Timepointd35:TreatmentControl:SexF.
## Interpret type III hypotheses with care.
```

```
print(IL17_anova_table_for_sex)
```

```
## Type III Analysis of Variance Table with Kenward-Roger's method
##                         Sum Sq Mean Sq NumDF  DenDF F value  Pr(>F)
## Timepoint               498.99 249.493     2 34.625  3.7572 0.03336 *
## Treatment               211.18 211.182     1 12.630  3.1809 0.09853 .
## Sex                      32.61  32.606     1 34.850  0.4911 0.48808
## Timepoint:Treatment     266.45 133.223     2 34.322  2.0064 0.14994
## Timepoint:Sex            19.58   9.791     2 34.810  0.1470 0.86386
## Treatment:Sex            16.73  16.733     1 34.227  0.2520 0.61886
## Timepoint:Treatment:Sex   7.85   7.851     1 36.214  0.1183 0.73292
## ---
## Signif. codes:  0 '***' 0.001 '**' 0.01 '*' 0.05 '.' 0.1 ' ' 1
```

```
IL17_emcatcat_sex <- emmeans(IL17_sex_effects, ~ Timepoint * Treatment * Sex)
#Is there a difference in males or females between treatment group at each time point?
contrast(IL17_emcatcat_sex, "revpairwise", by=c("Sex", "Timepoint"), adjust="BH")
```

```
## Sex = F, Timepoint = d7:
##  contrast      estimate   SE   df t.ratio p.value
##  PNS - Control   0.0863 6.05 31.0   0.014  0.9887
##
## Sex = M, Timepoint = d7:
##  contrast      estimate   SE   df t.ratio p.value
##  PNS - Control   0.8865 9.70 37.7   0.091  0.9277
##
## Sex = F, Timepoint = d21:
##  contrast      estimate   SE   df t.ratio p.value
##  PNS - Control  -0.2969 6.37 29.5  -0.047  0.9631
##
## Sex = M, Timepoint = d21:
##  contrast      estimate   SE   df t.ratio p.value
##  PNS - Control   5.6016 6.06 25.9   0.924  0.3640
##
## Sex = F, Timepoint = d35:
##  contrast      estimate   SE   df t.ratio p.value
##  PNS - Control   nonEst   NA   NA      NA      NA
##
## Sex = M, Timepoint = d35:
##  contrast      estimate   SE   df t.ratio p.value
##  PNS - Control  23.6514 8.96 37.9   2.639  0.0120
##
## Degrees-of-freedom method: kenward-roger
```

```
#Is there a difference in males or females within treatment group at each time point?
contrast(IL17_emcatcat_sex, "revpairwise", by=c("Treatment", "Timepoint"), adjust="BH")
```

```
## Treatment = Control, Timepoint = d7:
##  contrast estimate   SE   df t.ratio p.value
##  M - F       -0.46 9.60 37.2  -0.048  0.9620
##
## Treatment = PNS, Timepoint = d7:
##  contrast estimate   SE   df t.ratio p.value
##  M - F        0.34 5.63 36.1   0.060  0.9521
##
## Treatment = Control, Timepoint = d21:
##  contrast estimate   SE   df t.ratio p.value
##  M - F        0.87 6.63 30.2   0.131  0.8965
##
## Treatment = PNS, Timepoint = d21:
##  contrast estimate   SE   df t.ratio p.value
##  M - F        6.77 5.58 36.0   1.213  0.2331
##
## Treatment = Control, Timepoint = d35:
##  contrast estimate   SE   df t.ratio p.value
##  M - F      nonEst   NA   NA      NA      NA
##
## Treatment = PNS, Timepoint = d35:
##  contrast estimate   SE   df t.ratio p.value
##  M - F        4.67 6.84 32.8   0.683  0.4994
##
## Degrees-of-freedom method: kenward-roger
```

Supplementary File 3. Offspring Colon Gene Expression Analysis

```
library(tidyverse)
library(lme4)
library(lmerTest)
library(emmeans)
```

Colon Tissue Gene Expression–Figure 3

Read in Colon Gene Expression Data

```
colon_dat <- read_csv("CT_expression.csv")
```

```
## Rows: 82 Columns: 21
## ── Column specification ────────────────────────────────────────────────────────
## Delimiter: ","
## chr  (5): SampleID, Treatment, Timepoint, Sex, Litter
## dbl (16): Ahr, Foxp3, Ifng, Ifnb, IL1b, IL4, IL5, IL6, IL10, IL13, IL18, Isg...
##
## ℹ Use `spec()` to retrieve the full column specification for this data.
## ℹ Specify the column types or set `show_col_types = FALSE` to quiet this message.
```

```
summary(colon_dat)
```

```
##    SampleID          Treatment          Timepoint             Sex
##  Length:82          Length:82          Length:82          Length:82
##  Class :character   Class :character   Class :character   Class :character
##  Mode  :character   Mode  :character   Mode  :character   Mode  :character
##
##
##
##
##     Litter               Ahr            Foxp3             Ifng
##  Length:82          Min.   :0.155   Min.   : 0.101   Min.   :0.188
##  Class :character   1st Qu.:0.955   1st Qu.: 1.886   1st Qu.:0.518
##  Mode  :character   Median :2.036   Median :10.800   Median :0.791
##                     Mean   :2.313   Mean   :14.212   Mean   :1.257
##                     3rd Qu.:3.445   3rd Qu.:23.262   3rd Qu.:1.600
##                     Max.   :6.377   Max.   :59.378   Max.   :7.763
##                     NA's   :3       NA's   :5       NA's   :4
##       Ifnb             IL1b             IL4              IL5
##  Min.   :0.0490   Min.   : 0.218   Min.   : 0.056   Min.   :0.0660
##  1st Qu.:0.2557   1st Qu.: 1.169   1st Qu.: 1.133   1st Qu.:0.2067
##  Median :0.5000   Median : 1.877   Median : 2.608   Median :0.2895
##  Mean   :0.7700   Mean   : 2.587   Mean   : 6.459   Mean   :0.6769
##  3rd Qu.:1.0035   3rd Qu.: 3.125   3rd Qu.: 7.578   3rd Qu.:0.7218
##  Max.   :3.7680   Max.   :12.585   Max.   :50.398   Max.   :7.2200
##  NA's   :6        NA's   :3        NA's   :9        NA's   :4
##       IL6              IL10             IL13             IL18
##  Min.   :0.1080   Min.   : 0.138   Min.   :0.0560   Min.   : 0.295
##  1st Qu.:0.3900   1st Qu.: 1.081   1st Qu.:0.1650   1st Qu.: 1.112
##  Median :0.5430   Median : 3.046   Median :0.3840   Median : 1.748
##  Mean   :0.6529   Mean   : 4.209   Mean   :0.5294   Mean   : 2.856
##  3rd Qu.:0.7420   3rd Qu.: 6.467   3rd Qu.:0.7900   3rd Qu.: 3.854
##  Max.   :1.9200   Max.   :16.507   Max.   :1.7500   Max.   :12.110
##  NA's   :5        NA's   :3        NA's   :5        NA's   :1
##      Isg15             Muc2            Tgfb1             Tnf
##  Min.   :0.0630   Min.   : 0.209   Min.   :0.2020   Min.   :0.239
##  1st Qu.:0.3795   1st Qu.: 3.296   1st Qu.:0.4407   1st Qu.:0.763
##  Median :0.5630   Median :14.980   Median :0.7280   Median :1.127
##  Mean   :0.7042   Mean   :17.917   Mean   :0.7932   Mean   :1.338
##  3rd Qu.:0.8130   3rd Qu.:27.232   3rd Qu.:1.0822   3rd Qu.:1.697
##  Max.   :2.9210   Max.   :69.743   Max.   :1.8540   Max.   :4.170
##  NA's   :2        NA's   :4                         NA's   :5
##       IL17
##  Min.   :0.0100
##  1st Qu.:0.1705
##  Median :0.4200
##  Mean   :0.5909
##  3rd Qu.:0.9005
##  Max.   :2.7350
##  NA's   :15
```

```
# prep data
colon_dat$Timepoint <- factor(colon_dat$Timepoint, levels = c("d0", "d7", "d21", "d35"))
colon_dat$Treatment <- factor(colon_dat$Treatment, levels = c("Control", "PNS"))
colon_dat$Litter <- factor(colon_dat$Litter, levels = c("F5", "O4", "R4", "R5", "K6", "B4", "F4", "L4", "K5", "P5", "M6"))
colon_dat$Sex <- factor(colon_dat$Sex, levels = c("F", "M"))
```

Colon Tissue *Ifng* Expression

```
# Fit linear mixed-effects model
# Include random intercept for litter
Ifng_model <- lmer(Ifng ~ Timepoint * Treatment + (1 | Litter), data = colon_dat)
summary(Ifng_model)
```

```
## Linear mixed model fit by REML. t-tests use Satterthwaite's method [
## lmerModLmerTest]
## Formula: Ifng ~ Timepoint * Treatment + (1 | Litter)
##    Data: colon_dat
##
## REML criterion at convergence: 233.4
##
## Scaled residuals:
##     Min      1Q  Median      3Q     Max
## -1.3114 -0.5665 -0.1753  0.4839  4.5695
##
## Random effects:
##  Groups   Name        Variance Std.Dev.
##  Litter   (Intercept) 0.4163   0.6452
##  Residual             1.0780   1.0382
## Number of obs: 78, groups:  Litter, 11
##
## Fixed effects:
##                            Estimate Std. Error       df t value Pr(>|t|)
## (Intercept)                 1.24097    0.43964 21.07798   2.823   0.0102 *
## Timepointd7                 0.08832    0.51952 61.83842   0.170   0.8656
## Timepointd21               -0.11701    0.45704 60.71791  -0.256   0.7988
## Timepointd35                0.67011    0.52316 62.59148   1.281   0.2050
## TreatmentPNS                0.78582    0.60274 21.97003   1.304   0.2058
## Timepointd7:TreatmentPNS  -0.91151    0.69484 61.78081  -1.312   0.1944
## Timepointd21:TreatmentPNS -1.08720    0.62963 61.32658  -1.727   0.0893 .
## Timepointd35:TreatmentPNS -1.41290    0.70786 62.61342  -1.996   0.0503 .
## ---
## Signif. codes:  0 '***' 0.001 '**' 0.01 '*' 0.05 '.' 0.1 ' ' 1
##
## Correlation of Fixed Effects:
##             (Intr) Tmpnt7 Tmpn21 Tmpn35 TrtPNS T7:TPN T21:TP
## Timepointd7 -0.475
## Timepontd21 -0.547  0.445
## Timepontd35 -0.480  0.424  0.450
## TreatmntPNS -0.729  0.346  0.399  0.350
## Tmpnt7:TPNS  0.355 -0.748 -0.333 -0.317 -0.503
## Tmpn21:TPNS  0.397 -0.323 -0.726 -0.327 -0.557  0.480
## Tmpn35:TPNS  0.354 -0.314 -0.332 -0.739 -0.488  0.438  0.465
```

```
# Create a type III ANOVA table
Ifng_anova_table <- anova(Ifng_model, ddf = "Kenward-Roger")
print(Ifng_anova_table)
```

```
## Type III Analysis of Variance Table with Kenward-Roger's method
##                     Sum Sq Mean Sq NumDF  DenDF F value Pr(>F)
## Timepoint           5.8792 1.95975     3 63.008  1.8180 0.1530
## Treatment           0.0226 0.02263     1  8.937  0.0210 0.8880
## Timepoint:Treatment 5.1745 1.72485     3 63.008  1.6001 0.1983
```

```
#Because no interaction, test for main effects of treatment at individual time points
Ifng_emcatcat <- emmeans(Ifng_model, ~ Treatment | Timepoint)
#Is there a difference between treatment groups at each time point?
contrast(Ifng_emcatcat, "revpairwise", adjust="BH")
```

```
## Timepoint = d0:
##  contrast      estimate    SE   df t.ratio p.value
##  PNS - Control    0.786 0.604 24.1   1.302  0.2052
##
## Timepoint = d7:
##  contrast      estimate    SE   df t.ratio p.value
##  PNS - Control   -0.126 0.654 30.2  -0.192  0.8488
##
## Timepoint = d21:
##  contrast      estimate    SE   df t.ratio p.value
##  PNS - Control   -0.301 0.581 21.2  -0.519  0.6094
##
## Timepoint = d35:
##  contrast      estimate    SE   df t.ratio p.value
##  PNS - Control   -0.627 0.673 31.6  -0.932  0.3584
##
## Degrees-of-freedom method: kenward-roger
```

Colon Tissue *Ifnb* Expression

```
# Fit linear mixed-effects model
# Include random intercept for litter
Ifnb_model <- lmer(Ifnb ~ Timepoint * Treatment + (1 | Litter), data = colon_dat)
summary(Ifnb_model)
```

```
## Linear mixed model fit by REML. t-tests use Satterthwaite's method [
## lmerModLmerTest]
## Formula: Ifnb ~ Timepoint * Treatment + (1 | Litter)
##    Data: colon_dat
##
## REML criterion at convergence: 160.5
##
## Scaled residuals:
##     Min      1Q  Median      3Q     Max
## -1.6097 -0.5453 -0.2770  0.3879  3.1439
##
## Random effects:
##  Groups   Name        Variance Std.Dev.
##  Litter   (Intercept) 0.1257   0.3545
##  Residual             0.4136   0.6431
## Number of obs: 76, groups:  Litter, 11
##
## Fixed effects:
##                             Estimate Std. Error       df t value Pr(>|t|)
## (Intercept)                  1.23089    0.26950 26.24268   4.567 0.000103 ***
## Timepointd7                 -0.66845    0.32600 59.17709  -2.050 0.044758 *
## Timepointd21                -0.60940    0.30216 61.60014  -2.017 0.048076 *
## Timepointd35                 0.21900    0.32819 60.04939   0.667 0.507133
## TreatmentPNS                -0.10064    0.36348 26.06246  -0.277 0.784061
## Timepointd7:TreatmentPNS     0.10677    0.43332 59.62589   0.246 0.806209
## Timepointd21:TreatmentPNS   -0.01424    0.40381 61.11676  -0.035 0.971978
## Timepointd35:TreatmentPNS   -0.70595    0.44116 60.54888  -1.600 0.114755
## ---
## Signif. codes:  0 '***' 0.001 '**' 0.01 '*' 0.05 '.' 0.1 ' ' 1
##
## Correlation of Fixed Effects:
##              (Intr) Tmpnt7 Tmpn21 Tmpn35 TrtPNS T7:TPN T21:TP
## Timepointd7 -0.518
## Timepontd21 -0.592  0.452
## Timepontd35 -0.523  0.440  0.457
## TreatmntPNS -0.741  0.384  0.439  0.388
## Tmpnt7:TPNS  0.390 -0.752 -0.340 -0.331 -0.537
## Tmpn21:TPNS  0.443 -0.338 -0.748 -0.342 -0.593  0.482
## Tmpn35:TPNS  0.389 -0.327 -0.340 -0.744 -0.523  0.446  0.469
```

```
# Create a type III ANOVA table
Ifnb_anova_table <- anova(Ifnb_model, ddf = "Kenward-Roger")
print(Ifnb_anova_table)
```

```
## Type III Analysis of Variance Table with Kenward-Roger's method
##                     Sum Sq Mean Sq NumDF  DenDF F value  Pr(>F)
## Timepoint           5.6619 1.88729     3 61.717  4.5628 0.00596 **
## Treatment           0.3782 0.37816     1  8.828  0.9143 0.36443
## Timepoint:Treatment 1.6074 0.53581     3 61.717  1.2954 0.28401
## ---
## Signif. codes:  0 '***' 0.001 '**' 0.01 '*' 0.05 '.' 0.1 ' ' 1
```

```
#Because no interaction, test for main effects of treatment at individual time points
Ifnb_emcatcat <- emmeans(Ifnb_model, ~ Treatment | Timepoint)
#Is there a difference between treatment groups at each time point?
contrast(Ifnb_emcatcat, "revpairwise", adjust="BH")
```

```
## Timepoint = d0:
##  contrast      estimate    SE   df t.ratio p.value
##  PNS - Control -0.10064 0.364 27.8  -0.276  0.7844
##
## Timepoint = d7:
##  contrast      estimate    SE   df t.ratio p.value
##  PNS - Control  0.00614 0.390 32.9   0.016  0.9875
##
## Timepoint = d21:
##  contrast      estimate    SE   df t.ratio p.value
##  PNS - Control -0.11488 0.349 24.2  -0.329  0.7447
##
## Timepoint = d35:
##  contrast      estimate    SE   df t.ratio p.value
##  PNS - Control -0.80659 0.402 34.0  -2.008  0.0526
##
## Degrees-of-freedom method: kenward-roger
```

Colon Tissue *Isg15* Expression

```
# Fit linear mixed-effects model
# Include random intercept for litter
Isg15_model <- lmer(Isg15 ~ Timepoint * Treatment + (1 | Litter), data = colon_dat)
```

```
## boundary (singular) fit: see help('isSingular')
```

```
summary(Isg15_model)
```

```
## Linear mixed model fit by REML. t-tests use Satterthwaite's method [
## lmerModLmerTest]
## Formula: Isg15 ~ Timepoint * Treatment + (1 | Litter)
##    Data: colon_dat
##
## REML criterion at convergence: 113.3
##
## Scaled residuals:
##     Min      1Q  Median      3Q     Max
## -2.2204 -0.4583  0.0432  0.3861  3.2089
##
## Random effects:
##  Groups   Name        Variance Std.Dev.
##  Litter   (Intercept) 0.000    0.000
##  Residual             0.219    0.468
## Number of obs: 80, groups:  Litter, 11
##
## Fixed effects:
##                            Estimate Std. Error      df t value Pr(>|t|)
## (Intercept)                  1.1587     0.1480 72.0000   7.829  3.2e-11 ***
## Timepointd7                 -0.7057     0.2306 72.0000  -3.060  0.00311 **
## Timepointd21                -0.6079     0.2045 72.0000  -2.973  0.00401 **
## Timepointd35                -0.6518     0.2220 72.0000  -2.936  0.00446 **
## TreatmentPNS                 0.2605     0.2045 72.0000   1.274  0.20683
## Timepointd7:TreatmentPNS    -0.4161     0.3050 72.0000  -1.364  0.17671
## Timepointd21:TreatmentPNS  -0.2048     0.2803 72.0000  -0.731  0.46731
## Timepointd35:TreatmentPNS  -0.2457     0.3058 72.0000  -0.803  0.42442
## ---
## Signif. codes:  0 '***' 0.001 '**' 0.01 '*' 0.05 '.' 0.1 ' ' 1
##
## Correlation of Fixed Effects:
##             (Intr) Tmpnt7 Tmpn21 Tmpn35 TrtPNS T7:TPN T21:TP
## Timepointd7 -0.642
## Timepontd21 -0.724  0.464
## Timepontd35 -0.667  0.428  0.482
## TreatmntPNS -0.724  0.464  0.524  0.482
## Tmpnt7:TPNS  0.485 -0.756 -0.351 -0.324 -0.670
## Tmpn21:TPNS  0.528 -0.339 -0.729 -0.352 -0.729  0.489
## Tmpn35:TPNS  0.484 -0.311 -0.350 -0.726 -0.669  0.448  0.488
## optimizer (nloptwrap) convergence code: 0 (OK)
## boundary (singular) fit: see help('isSingular')
```

```
# Create a type III ANOVA table
Isg15_anova_table <- anova(Isg15_model, ddf = "Kenward-Roger")
print(Isg15_anova_table)
```

```
## Type III Analysis of Variance Table with Kenward-Roger's method
##                     Sum Sq Mean Sq NumDF  DenDF F value    Pr(>F)
## Timepoint           9.8545  3.2848     3 68.037 14.9948 1.361e-07 ***
## Treatment           0.0364  0.0364     1  8.319  0.1661    0.6939
## Timepoint:Treatment 0.4110  0.1370     3 68.037  0.6254    0.6011
## ---
## Signif. codes:  0 '***' 0.001 '**' 0.01 '*' 0.05 '.' 0.1 ' ' 1
```

```
#Because no interaction, test for main effects of treatment at individual time points
Isg15_emcatcat <- emmeans(Isg15_model, ~ Treatment | Timepoint)
#Is there a difference between treatment groups at each time point?
contrast(Isg15_emcatcat, "revpairwise", adjust="BH")
```

```
## Timepoint = d0:
##  contrast      estimate    SE   df t.ratio p.value
##  PNS - Control   0.2605 0.206 55.8   1.263  0.2118
##
## Timepoint = d7:
##  contrast      estimate    SE   df t.ratio p.value
##  PNS - Control  -0.1556 0.228 61.0  -0.682  0.4979
##
## Timepoint = d21:
##  contrast      estimate    SE   df t.ratio p.value
##  PNS - Control   0.0556 0.192 53.7   0.290  0.7732
##
## Timepoint = d35:
##  contrast      estimate    SE   df t.ratio p.value
##  PNS - Control   0.0148 0.231 55.6   0.064  0.9493
##
## Degrees-of-freedom method: kenward-roger
```

Colon Tissue *Il1b* Expression

```
# Fit linear mixed-effects model
# Include random intercept for litter
IL1b_model <- lmer(IL1b ~ Timepoint * Treatment + (1 | Litter), data = colon_dat)
summary(IL1b_model)
```

```
## Linear mixed model fit by REML. t-tests use Satterthwaite's method [
## lmerModLmerTest]
## Formula: IL1b ~ Timepoint * Treatment + (1 | Litter)
##    Data: colon_dat
##
## REML criterion at convergence: 318.9
##
## Scaled residuals:
##      Min       1Q   Median       3Q      Max
## -2.16913 -0.54033 -0.07422  0.38070  3.04564
##
## Random effects:
##  Groups   Name        Variance Std.Dev.
##  Litter   (Intercept) 1.172    1.083
##  Residual             3.482    1.866
## Number of obs: 79, groups:  Litter, 11
##
## Fixed effects:
##                           Estimate Std. Error      df t value Pr(>|t|)
## (Intercept)                 1.0884     0.7967 26.8198   1.366   0.1832
## Timepointd7                 2.2658     0.9459 62.4837   2.395   0.0196 *
## Timepointd21                3.8155     0.8533 63.9497   4.471 3.25e-05 ***
## Timepointd35                1.8578     0.9159 62.9860   2.028   0.0467 *
## TreatmentPNS                0.4019     1.0607 25.6040   0.379   0.7079
## Timepointd7:TreatmentPNS   -1.6726     1.2306 62.6140  -1.359   0.1790
## Timepointd21:TreatmentPNS  -2.6797     1.1409 63.5906  -2.349   0.0220 *
## Timepointd35:TreatmentPNS  -1.5997     1.2652 63.6510  -1.264   0.2107
## ---
## Signif. codes:  0 '***' 0.001 '**' 0.01 '*' 0.05 '.' 0.1 ' ' 1
##
## Correlation of Fixed Effects:
##             (Intr) Tmpnt7 Tmpn21 Tmpn35 TrtPNS T7:TPN T21:TP
## Timepointd7 -0.509
## Timepontd21 -0.595  0.468
## Timepontd35 -0.533  0.459  0.491
## TreatmntPNS -0.751  0.382  0.447  0.401
## Tmpnt7:TPNS  0.391 -0.769 -0.360 -0.353 -0.522
## Tmpn21:TPNS  0.445 -0.350 -0.748 -0.367 -0.580  0.485
## Tmpn35:TPNS  0.386 -0.332 -0.356 -0.724 -0.510  0.445  0.468
```

```
# Create a type III ANOVA table
IL1b_anova_table <- anova(IL1b_model, ddf = "Kenward-Roger")
print(IL1b_anova_table)
```

```
## Type III Analysis of Variance Table with Kenward-Roger's method
##                     Sum Sq Mean Sq NumDF  DenDF F value    Pr(>F)
## Timepoint           66.145 22.0482     3 64.179  6.3325 0.0007892 ***
## Treatment            6.557  6.5575     1  8.974  1.8834 0.2032694
## Timepoint:Treatment 19.359  6.4528     3 64.179  1.8533 0.1464237
## ---
## Signif. codes:  0 '***' 0.001 '**' 0.01 '*' 0.05 '.' 0.1 ' ' 1
```

```
#Because no interaction, test for main effects of treatment at individual time points
IL1b_emcatcat <- emmeans(IL1b_model, ~ Treatment | Timepoint)
#Is there a difference between treatment groups at each time point?
contrast(IL1b_emcatcat, "revpairwise", adjust="BH")
```

```
## Timepoint = d0:
##  contrast      estimate   SE   df t.ratio p.value
##  PNS - Control    0.402 1.06 26.5   0.378  0.7083
##
## Timepoint = d7:
##  contrast      estimate   SE   df t.ratio p.value
##  PNS - Control   -1.271 1.13 31.4  -1.121  0.2709
##
## Timepoint = d21:
##  contrast      estimate   SE   df t.ratio p.value
##  PNS - Control   -2.278 1.01 22.9  -2.249  0.0344
##
## Timepoint = d35:
##  contrast      estimate   SE   df t.ratio p.value
##  PNS - Control   -1.198 1.17 33.5  -1.023  0.3137
##
## Degrees-of-freedom method: kenward-roger
```

Colon Tissue *Il6* Expression

```
# Fit linear mixed-effects model
# Include random intercept for litter
IL6_model <- lmer(IL6 ~ Timepoint * Treatment + (1 | Litter), data = colon_dat)
```

```
## boundary (singular) fit: see help('isSingular')
```

```
summary(IL6_model)
```

```
## Linear mixed model fit by REML. t-tests use Satterthwaite's method [
## lmerModLmerTest]
## Formula: IL6 ~ Timepoint * Treatment + (1 | Litter)
##    Data: colon_dat
##
## REML criterion at convergence: 79
##
## Scaled residuals:
##      Min       1Q   Median       3Q      Max
## -1.68917 -0.73479 -0.08065  0.37854  2.70101
##
## Random effects:
##  Groups   Name        Variance Std.Dev.
##  Litter   (Intercept) 0.0000   0.0000
##  Residual             0.1419   0.3768
## Number of obs: 77, groups:  Litter, 11
##
## Fixed effects:
##                             Estimate Std. Error      df t value Pr(>|t|)
## (Intercept)                   1.1314     0.1191 69.0000   9.496 3.76e-14 ***
## Timepointd7                  -0.4646     0.1946 69.0000  -2.388 0.019690 *
## Timepointd21                 -0.6641     0.1685 69.0000  -3.941 0.000192 ***
## Timepointd35                 -0.3434     0.1787 69.0000  -1.922 0.058794 .
## TreatmentPNS                 -0.4026     0.1646 69.0000  -2.446 0.017017 *
## Timepointd7:TreatmentPNS      0.2309     0.2523 69.0000   0.915 0.363237
## Timepointd21:TreatmentPNS     0.4247     0.2285 69.0000   1.859 0.067362 .
## Timepointd35:TreatmentPNS     0.1342     0.2502 69.0000   0.536 0.593362
## ---
## Signif. codes:  0 '***' 0.001 '**' 0.01 '*' 0.05 '.' 0.1 ' ' 1
##
## Correlation of Fixed Effects:
##             (Intr) Tmpnt7 Tmpn21 Tmpn35 TrtPNS T7:TPN T21:TP
## Timepointd7 -0.612
## Timepontd21 -0.707  0.433
## Timepontd35 -0.667  0.408  0.471
## TreatmntPNS -0.724  0.443  0.512  0.482
## Tmpnt7:TPNS  0.472 -0.771 -0.334 -0.315 -0.652
## Tmpn21:TPNS  0.521 -0.319 -0.737 -0.348 -0.720  0.470
## Tmpn35:TPNS  0.476 -0.292 -0.337 -0.714 -0.658  0.429  0.474
## optimizer (nloptwrap) convergence code: 0 (OK)
## boundary (singular) fit: see help('isSingular')
```

```
# Create a type III ANOVA table
IL6_anova_table <- anova(IL6_model, ddf = "Kenward-Roger")
print(IL6_anova_table)
```

```
## Type III Analysis of Variance Table with Kenward-Roger's method
##                      Sum Sq Mean Sq NumDF  DenDF F value   Pr(>F)
## Timepoint           2.33593 0.77864     3 65.461  5.4851 0.002007 **
## Treatment           0.74615 0.74615     1  8.259  5.2566 0.050058 .
## Timepoint:Treatment 0.50492 0.16831     3 65.461  1.1856 0.322090
## ---
## Signif. codes:  0 '***' 0.001 '**' 0.01 '*' 0.05 '.' 0.1 ' ' 1
```

```
#Because no interaction, test for main effects of treatment at individual time points
IL6_emcatcat <- emmeans(IL6_model, ~ Treatment | Timepoint)
#Is there a difference between treatment groups at each time point?
contrast(IL6_emcatcat, "revpairwise", adjust="BH")
```

```
## Timepoint = d0:
##  contrast      estimate    SE   df t.ratio p.value
##  PNS - Control  -0.4026 0.166 53.0  -2.425  0.0187
##
## Timepoint = d7:
##  contrast      estimate    SE   df t.ratio p.value
##  PNS - Control  -0.1717 0.193 60.3  -0.888  0.3779
##
## Timepoint = d21:
##  contrast      estimate    SE   df t.ratio p.value
##  PNS - Control   0.0221 0.159 50.4   0.139  0.8904
##
## Timepoint = d35:
##  contrast      estimate    SE   df t.ratio p.value
##  PNS - Control  -0.2684 0.192 53.4  -1.397  0.1682
##
## Degrees-of-freedom method: kenward-roger
```

Colon Tissue *Tnfa* Expression

```
# Fit linear mixed-effects model
# Include random intercept for litter
Tnf_model <- lmer(Tnf ~ Timepoint * Treatment + (1 | Litter), data = colon_dat)
summary(Tnf_model)
```

```
## Linear mixed model fit by REML. t-tests use Satterthwaite's method [
## lmerModLmerTest]
## Formula: Tnf ~ Timepoint * Treatment + (1 | Litter)
##    Data: colon_dat
##
## REML criterion at convergence: 139.6
##
## Scaled residuals:
##     Min      1Q  Median      3Q     Max
## -2.2244 -0.5621 -0.1802  0.4223  2.6573
##
## Random effects:
##  Groups   Name        Variance Std.Dev.
##  Litter   (Intercept) 0.1864   0.4318
##  Residual             0.2738   0.5233
## Number of obs: 77, groups:  Litter, 11
##
## Fixed effects:
##                           Estimate Std. Error       df t value Pr(>|t|)
## (Intercept)                0.98595    0.26249 18.72949   3.756 0.001365 **
## Timepointd7                0.98864    0.27109 61.59600   3.647 0.000548 ***
## Timepointd21               0.33673    0.24276 60.19729   1.387 0.170518
## Timepointd35              -0.08036    0.27518 62.34776  -0.292 0.771250
## TreatmentPNS               0.29072    0.35453 18.57157   0.820 0.422596
## Timepointd7:TreatmentPNS   0.12566    0.35342 61.16822   0.356 0.723406
## Timepointd21:TreatmentPNS -0.45553    0.32492 60.10946  -1.402 0.166069
## Timepointd35:TreatmentPNS -0.26790    0.36528 61.73032  -0.733 0.466086
## ---
## Signif. codes:  0 '***' 0.001 '**' 0.01 '*' 0.05 '.' 0.1 ' ' 1
##
## Correlation of Fixed Effects:
##             (Intr) Tmpnt7 Tmpn21 Tmpn35 TrtPNS T7:TPN T21:TP
## Timepointd7 -0.444
## Timepontd21 -0.489  0.452
## Timepontd35 -0.452  0.466  0.461
## TreatmntPNS -0.740  0.329  0.362  0.335
## Tmpnt7:TPNS  0.341 -0.767 -0.347 -0.358 -0.460
## Tmpn21:TPNS  0.365 -0.338 -0.747 -0.345 -0.490  0.482
## Tmpn35:TPNS  0.341 -0.351 -0.348 -0.753 -0.443  0.459  0.468
```

```
# Create a type III ANOVA table
Tnf_anova_table <- anova(Tnf_model, ddf = "Kenward-Roger")
print(Tnf_anova_table)
```

```
## Type III Analysis of Variance Table with Kenward-Roger's method
##                     Sum Sq Mean Sq NumDF  DenDF F value    Pr(>F)
## Timepoint          15.2838  5.0946     3 61.606 18.6072 1.043e-08 ***
## Treatment           0.0650  0.0650     1  8.947  0.2373    0.6379
## Timepoint:Treatment  0.9723  0.3241     3 61.606  1.1837    0.3233
## ---
## Signif. codes:  0 '***' 0.001 '**' 0.01 '*' 0.05 '.' 0.1 ' ' 1
```

```
#Because no interaction, test for main effects of treatment at individual time points
Tnf_emcatcat <- emmeans(Tnf_model, ~ Treatment | Timepoint)
#Is there a difference between treatment groups at each time point?
contrast(Tnf_emcatcat, "revpairwise", adjust="BH")
```

```
## Timepoint = d0:
##  contrast      estimate    SE   df t.ratio p.value
##  PNS - Control   0.2907 0.355 19.2   0.819  0.4227
##
## Timepoint = d7:
##  contrast      estimate    SE   df t.ratio p.value
##  PNS - Control   0.4164 0.368 21.7   1.130  0.2708
##
## Timepoint = d21:
##  contrast      estimate    SE   df t.ratio p.value
##  PNS - Control  -0.1648 0.344 17.2  -0.479  0.6382
##
## Timepoint = d35:
##  contrast      estimate    SE   df t.ratio p.value
##  PNS - Control   0.0228 0.381 23.8   0.060  0.9528
##
## Degrees-of-freedom method: kenward-roger
```

Colon Tissue *Ahr* Expression

```
# Fit linear mixed-effects model
# Include random intercept for litter
Ahr_model <- lmer(Ahr ~ Timepoint * Treatment + (1 | Litter), data = colon_dat)
summary(Ahr_model)
```

```
## Linear mixed model fit by REML. t-tests use Satterthwaite's method [
## lmerModLmerTest]
## Formula: Ahr ~ Timepoint * Treatment + (1 | Litter)
##    Data: colon_dat
##
## REML criterion at convergence: 244.1
##
## Scaled residuals:
##      Min       1Q   Median       3Q      Max
## -1.72367 -0.64587 -0.09239  0.57757  2.72891
##
## Random effects:
##  Groups   Name        Variance Std.Dev.
##  Litter   (Intercept) 0.332    0.5762
##  Residual             1.236    1.1116
## Number of obs: 79, groups:  Litter, 11
##
## Fixed effects:
##                              Estimate Std. Error      df t value Pr(>|t|)
## (Intercept)                    1.0582     0.4561 27.4583   2.320 0.028006 *
## Timepointd7                    3.9054     0.5632 61.4857   6.934  2.9e-09 ***
## Timepointd21                   1.4590     0.5076 63.3055   2.874 0.005508 **
## Timepointd35                   1.7985     0.5452 62.1205   3.299 0.001610 **
## TreatmentPNS                   0.2068     0.6136 27.2897   0.337 0.738646
## Timepointd7:TreatmentPNS      -2.5720     0.7374 61.3023  -3.488 0.000907 ***
## Timepointd21:TreatmentPNS     -0.7808     0.6827 62.2277  -1.144 0.257129
## Timepointd35:TreatmentPNS     -1.0942     0.7488 63.3001  -1.461 0.148871
## ---
## Signif. codes:  0 '***' 0.001 '**' 0.01 '*' 0.05 '.' 0.1 ' ' 1
##
## Correlation of Fixed Effects:
##             (Intr) Tmpnt7 Tmpn21 Tmpn35 TrtPNS T7:TPN T21:TP
## Timepointd7 -0.530
## Timepontd21 -0.617  0.471
## Timepontd35 -0.555  0.458  0.493
## TreatmntPNS -0.743  0.394  0.459  0.413
## Tmpnt7:TPNS  0.405 -0.764 -0.359 -0.350 -0.551
## Tmpn21:TPNS  0.459 -0.350 -0.743 -0.367 -0.611  0.492
## Tmpn35:TPNS  0.404 -0.334 -0.359 -0.728 -0.548  0.458  0.486
```

```
# Create a type III ANOVA table
Ahr_anova_table <- anova(Ahr_model, ddf = "Kenward-Roger")
print(Ahr_anova_table)
```

```
## Type III Analysis of Variance Table with Kenward-Roger's method
##                     Sum Sq Mean Sq NumDF  DenDF F value    Pr(>F)
## Timepoint           62.469 20.8231     3 64.396 16.8507 3.457e-08 ***
## Treatment            5.309  5.3092     1  8.909  4.2965  0.068369 .
## Timepoint:Treatment 15.512  5.1705     3 64.396  4.1841  0.009074 **
## ---
## Signif. codes:  0 '***' 0.001 '**' 0.01 '*' 0.05 '.' 0.1 ' ' 1
```

```
# Because we have an interaction
Ahr_emcatcat <- emmeans(Ahr_model, ~ Timepoint * Treatment)

#Is there a difference between treatment groups at each time point?
contrast(Ahr_emcatcat, "revpairwise", by="Timepoint", adjust="BH")
```

```
## Timepoint = d0:
##  contrast      estimate    SE   df t.ratio p.value
##  PNS - Control    0.207 0.615 30.6   0.336  0.7389
##
## Timepoint = d7:
##  contrast      estimate    SE   df t.ratio p.value
##  PNS - Control   -2.365 0.652 34.9  -3.630  0.0009
##
## Timepoint = d21:
##  contrast      estimate    SE   df t.ratio p.value
##  PNS - Control   -0.574 0.576 25.3  -0.997  0.3281
##
## Timepoint = d35:
##  contrast      estimate    SE   df t.ratio p.value
##  PNS - Control   -0.887 0.663 34.9  -1.339  0.1892
##
## Degrees-of-freedom method: kenward-roger
```

```
#Is there a difference between time points within each treatment?
contrast(Ahr_emcatcat, "revpairwise", by="Treatment", adjust="BH")
```

```
## Treatment = Control:
##  contrast  estimate    SE   df t.ratio p.value
##  d7 - d0     3.9054 0.564 63.0   6.923  <.0001
##  d21 - d0    1.4590 0.510 64.5   2.861  0.0068
##  d21 - d7   -2.4464 0.558 66.6  -4.388  0.0001
##  d35 - d0    1.7985 0.547 63.5   3.290  0.0025
##  d35 - d7   -2.1069 0.577 62.5  -3.649  0.0011
##  d35 - d21   0.3395 0.535 66.6   0.634  0.5282
##
## Treatment = PNS:
##  contrast  estimate    SE   df t.ratio p.value
##  d7 - d0     1.3334 0.476 62.6   2.799  0.0409
##  d21 - d0    0.6781 0.457 62.4   1.484  0.2668
##  d21 - d7   -0.6553 0.457 62.4  -1.434  0.2668
##  d35 - d0    0.7043 0.517 65.6   1.362  0.2668
##  d35 - d7   -0.6292 0.520 66.0  -1.210  0.2765
##  d35 - d21   0.0262 0.502 66.1   0.052  0.9586
##
## Degrees-of-freedom method: kenward-roger
## P value adjustment: BH method for 6 tests
```

Colon Tissue *Muc2* Expression

```
##Muc2
# Fit linear mixed-effects model
Muc2_model <- lmer(Muc2 ~ Timepoint * Treatment + (1 | Litter), data = colon_dat) # include random intercept for litter
summary(Muc2_model)
```

```
## Linear mixed model fit by REML. t-tests use Satterthwaite's method [
## lmerModLmerTest]
## Formula: Muc2 ~ Timepoint * Treatment + (1 | Litter)
##    Data: colon_dat
##
## REML criterion at convergence: 545.7
##
## Scaled residuals:
##     Min      1Q  Median      3Q     Max
## -2.3772 -0.4213  0.0438  0.2397  3.5400
##
## Random effects:
##  Groups   Name        Variance Std.Dev.
##  Litter   (Intercept) 21.65    4.653
##  Residual             97.79    9.889
## Number of obs: 78, groups:  Litter, 11
##
## Fixed effects:
##                            Estimate Std. Error      df t value Pr(>|t|)
## (Intercept)                  1.6869     3.9111 32.8107   0.431    0.669
## Timepointd7                  4.3242     5.2900 63.5841   0.817    0.417
## Timepointd21                24.9847     4.5525 61.2041   5.488 8.24e-07 ***
## Timepointd35                37.8358     4.8859 64.0781   7.744 9.05e-11 ***
## TreatmentPNS                 0.5861     5.2908 32.5014   0.111    0.912
## Timepointd7:TreatmentPNS     1.1146     6.7947 63.1691   0.164    0.870
## Timepointd21:TreatmentPNS    0.6809     6.0774 61.7719   0.112    0.911
## Timepointd35:TreatmentPNS   -8.2571     6.6514 63.7671  -1.241    0.219
## ---
## Signif. codes:  0 '***' 0.001 '**' 0.01 '*' 0.05 '.' 0.1 ' ' 1
##
## Correlation of Fixed Effects:
##             (Intr) Tmpnt7 Tmpn21 Tmpn35 TrtPNS T7:TPN T21:TP
## Timepointd7 -0.529
## Timepontd21 -0.609  0.442
## Timepontd35 -0.578  0.447  0.478
## TreatmntPNS -0.739  0.391  0.450  0.427
## Tmpnt7:TPNS  0.412 -0.779 -0.344 -0.348 -0.559
## Tmpn21:TPNS  0.456 -0.331 -0.749 -0.358 -0.621  0.481
## Tmpn35:TPNS  0.424 -0.328 -0.351 -0.735 -0.566  0.451  0.482
```

```
# Create a type III ANOVA table
Muc2_anova_table <- anova(Muc2_model, ddf = "Kenward-Roger")
print(Muc2_anova_table)
```

```
## Type III Analysis of Variance Table with Kenward-Roger's method
##                     Sum Sq Mean Sq NumDF  DenDF F value   Pr(>F)
## Timepoint          14081.0  4693.7     3 63.724 47.9966 2.41e-16 ***
## Treatment              7.7     7.7     1  9.013  0.0786   0.7855
## Timepoint:Treatment   242.8    80.9     3 63.724  0.8278   0.4835
## ---
## Signif. codes:  0 '***' 0.001 '**' 0.01 '*' 0.05 '.' 0.1 ' ' 1
```

```
#Because no interaction, test for main effects of treatment at individual time points
Muc2_emcatcat <- emmeans(Muc2_model, ~ Treatment | Timepoint)
#Is there a difference between treatment groups at each time point?
contrast(Muc2_emcatcat, "revpairwise", adjust="BH")
```

```
## Timepoint = d0:
##  contrast      estimate   SE   df t.ratio p.value
##  PNS - Control    0.586 5.30 33.1   0.111  0.9126
##
## Timepoint = d7:
##  contrast      estimate   SE   df t.ratio p.value
##  PNS - Control    1.701 5.85 41.1   0.291  0.7727
##
## Timepoint = d21:
##  contrast      estimate   SE   df t.ratio p.value
##  PNS - Control    1.267 5.01 27.8   0.253  0.8021
##
## Timepoint = d35:
##  contrast      estimate   SE   df t.ratio p.value
##  PNS - Control   -7.671 5.74 37.2  -1.337  0.1893
##
## Degrees-of-freedom method: kenward-roger
```

Supplementary Gene Expression

Colon Tissue *Il10* Expression

```
# Fit linear mixed-effects model
# Include random intercept for litter
IL10_model <- lmer(IL10 ~ Timepoint * Treatment + (1 | Litter), data = colon_dat)
summary(IL10_model)
```

```
## Linear mixed model fit by REML. t-tests use Satterthwaite's method [
## lmerModLmerTest]
## Formula: IL10 ~ Timepoint * Treatment + (1 | Litter)
##    Data: colon_dat
##
## REML criterion at convergence: 353.5
##
## Scaled residuals:
##      Min       1Q   Median       3Q      Max
## -1.96565 -0.73017  0.03673  0.45998  2.21395
##
## Random effects:
##  Groups   Name        Variance Std.Dev.
##  Litter   (Intercept) 3.939    1.985
##  Residual             5.238    2.289
## Number of obs: 79, groups:  Litter, 11
##
## Fixed effects:
##                           Estimate Std. Error      df t value Pr(>|t|)
## (Intercept)                 1.7114     1.1506 16.1622   1.487   0.1562
## Timepointd7                 1.9501     1.1486 62.5217   1.698   0.0945 .
## Timepointd21                5.2382     1.0086 61.7844   5.193 2.45e-06 ***
## Timepointd35                6.1438     1.1119 62.8632   5.526 6.71e-07 ***
## TreatmentPNS               -0.7121     1.5719 16.6977  -0.453   0.6564
## Timepointd7:TreatmentPNS   -0.9741     1.5168 62.3086  -0.642   0.5231
## Timepointd21:TreatmentPNS  -1.0140     1.3828 61.7676  -0.733   0.4662
## Timepointd35:TreatmentPNS  -1.3082     1.5629 62.9559  -0.837   0.4058
## ---
## Signif. codes:  0 '***' 0.001 '**' 0.01 '*' 0.05 '.' 0.1 ' ' 1
##
## Correlation of Fixed Effects:
##             (Intr) Tmpnt7 Tmpn21 Tmpn35 TrtPNS T7:TPN T21:TP
## Timepointd7 -0.399
## Timepontd21 -0.462  0.442
## Timepontd35 -0.420  0.447  0.465
## TreatmntPNS -0.732  0.292  0.338  0.307
## Tmpnt7:TPNS  0.302 -0.757 -0.334 -0.339 -0.429
## Tmpn21:TPNS  0.337 -0.322 -0.729 -0.339 -0.472  0.480
## Tmpn35:TPNS  0.299 -0.318 -0.331 -0.711 -0.415  0.442  0.460
```

```
# Create a type III ANOVA table
IL10_anova_table <- anova(IL10_model, ddf = "Kenward-Roger")
print(IL10_anova_table)
```

```
## Type III Analysis of Variance Table with Kenward-Roger's method
##                     Sum Sq Mean Sq NumDF  DenDF F value    Pr(>F)
## Timepoint           388.34 129.446     3 63.190 24.7151 1.065e-10 ***
## Treatment             7.12   7.123     1  9.014  1.3600    0.2735
## Timepoint:Treatment   4.63   1.542     3 63.190  0.2944    0.8293
## ---
## Signif. codes:  0 '***' 0.001 '**' 0.01 '*' 0.05 '.' 0.1 ' ' 1
```

```
#Because no interaction, test for main effects of treatment at individual time points
IL10_emcatcat <- emmeans(IL10_model, ~ Treatment | Timepoint)
#Is there a difference between treatment groups at each time point?
contrast(IL10_emcatcat, "revpairwise", adjust="BH")
```

```
## Timepoint = d0:
##  contrast      estimate   SE   df t.ratio p.value
##  PNS - Control   -0.712 1.57 17.8  -0.453  0.6562
##
## Timepoint = d7:
##  contrast      estimate   SE   df t.ratio p.value
##  PNS - Control   -1.686 1.65 21.0  -1.020  0.3192
##
## Timepoint = d21:
##  contrast      estimate   SE   df t.ratio p.value
##  PNS - Control   -1.726 1.53 16.1  -1.130  0.2751
##
## Timepoint = d35:
##  contrast      estimate   SE   df t.ratio p.value
##  PNS - Control   -2.020 1.70 22.8  -1.189  0.2467
##
## Degrees-of-freedom method: kenward-roger
```

Colon Tissue *Tgfb1* Expression

```
# Fit linear mixed-effects model
# Include random intercept for litter
Tgfb1_model <- lmer(Tgfb1 ~ Timepoint * Treatment + (1 | Litter), data = colon_dat)
summary(Tgfb1_model)
```

```
## Linear mixed model fit by REML. t-tests use Satterthwaite's method [
## lmerModLmerTest]
## Formula: Tgfb1 ~ Timepoint * Treatment + (1 | Litter)
##    Data: colon_dat
##
## REML criterion at convergence: 65.8
##
## Scaled residuals:
##      Min       1Q   Median       3Q      Max
## -2.51934 -0.60784 -0.02894  0.56153  2.04230
##
## Random effects:
##  Groups   Name        Variance Std.Dev.
##  Litter   (Intercept) 0.01243  0.1115
##  Residual             0.10294  0.3208
## Number of obs: 82, groups:  Litter, 11
##
## Fixed effects:
##                              Estimate Std. Error        df t value Pr(>|t|)
## (Intercept)                  1.112616   0.113643 36.308985   9.790 9.96e-12 ***
## Timepointd7                 -0.098138   0.159500 66.943369  -0.615   0.5405
## Timepointd21                -0.346119   0.140837 65.267017  -2.458   0.0167 *
## Timepointd35                -0.659482   0.153955 67.870239  -4.284 5.93e-05 ***
## TreatmentPNS                -0.001453   0.153803 36.411646  -0.009   0.9925
## Timepointd7:TreatmentPNS   -0.319187   0.208817 66.410767  -1.529   0.1311
## Timepointd21:TreatmentPNS -0.083265   0.189468 65.142425  -0.439   0.6618
## Timepointd35:TreatmentPNS -0.066571   0.210580 68.256980  -0.316   0.7529
## ---
## Signif. codes:  0 '***' 0.001 '**' 0.01 '*' 0.05 '.' 0.1 ' ' 1
##
## Correlation of Fixed Effects:
##             (Intr) Tmpnt7 Tmpn21 Tmpn35 TrtPNS T7:TPN T21:TP
## Timepointd7 -0.570
## Timepontd21 -0.651  0.454
## Timepontd35 -0.596  0.437  0.474
## TreatmntPNS -0.739  0.421  0.481  0.441
## Tmpnt7:TPNS  0.435 -0.764 -0.346 -0.334 -0.593
## Tmpn21:TPNS  0.484 -0.337 -0.743 -0.353 -0.655  0.478
## Tmpn35:TPNS  0.436 -0.319 -0.347 -0.731 -0.588  0.439  0.472
```

```
# Create a type III ANOVA table
Tgfb1_anova_table <- anova(Tgfb1_model, ddf = "Kenward-Roger")
print(Tgfb1_anova_table)
```

```
## Type III Analysis of Variance Table with Kenward-Roger's method
##                     Sum Sq Mean Sq NumDF  DenDF F value    Pr(>F)
## Timepoint           4.6156 1.53852     3 68.233 14.9445 1.409e-07 ***
## Treatment           0.1450 0.14499     1  8.892  1.4085    0.2661
## Timepoint:Treatment 0.2589 0.08629     3 68.233  0.8382    0.4776
## ---
## Signif. codes:  0 '***' 0.001 '**' 0.01 '*' 0.05 '.' 0.1 ' ' 1
```

```
#Because no interaction, test for main effects of treatment at individual time points
Tgfb1_emcatcat <- emmeans(Tgfb1_model, ~ Treatment | Timepoint)
#Is there a difference between treatment groups at each time point?
contrast(Tgfb1_emcatcat, "revpairwise", adjust="BH")
```

```
## Timepoint = d0:
##  contrast      estimate    SE   df t.ratio p.value
##  PNS - Control -0.00145 0.154 39.5  -0.009  0.9925
##
## Timepoint = d7:
##  contrast      estimate    SE   df t.ratio p.value
##  PNS - Control -0.32064 0.172 48.1  -1.868  0.0679
##
## Timepoint = d21:
##  contrast      estimate    SE   df t.ratio p.value
##  PNS - Control -0.08472 0.146 35.0  -0.579  0.5664
##
## Timepoint = d35:
##  contrast      estimate    SE   df t.ratio p.value
##  PNS - Control -0.06802 0.175 46.4  -0.390  0.6987
##
## Degrees-of-freedom method: kenward-roger
```

Colon Tissue *Foxp3* Expression

```
# Fit linear mixed-effects model
Foxp3_model <- lmer(Foxp3 ~ Timepoint * Treatment + (1 | Litter), data = colon_dat) # include random intercept for litter
summary(Foxp3_model)
```

```
## Linear mixed model fit by REML. t-tests use Satterthwaite's method [
## lmerModLmerTest]
## Formula: Foxp3 ~ Timepoint * Treatment + (1 | Litter)
##    Data: colon_dat
##
## REML criterion at convergence: 546.1
##
## Scaled residuals:
##     Min      1Q  Median      3Q     Max
## -1.5641 -0.6014 -0.1334  0.2559  3.7506
##
## Random effects:
##  Groups   Name        Variance Std.Dev.
##  Litter   (Intercept)  29.73    5.452
##  Residual             108.08   10.396
## Number of obs: 77, groups:  Litter, 11
##
## Fixed effects:
##                           Estimate Std. Error       df t value Pr(>|t|)
## (Intercept)                 0.4180     4.2828  28.2390   0.098 0.922942
## Timepointd7                29.5282     5.5162  60.5153   5.353 1.41e-06 ***
## Timepointd21               15.9299     4.8811  62.9126   3.264 0.001781 **
## Timepointd35               22.0252     5.3027  61.2365   4.154 0.000103 ***
## TreatmentPNS                0.6682     5.7748  28.0803   0.116 0.908708
## Timepointd7:TreatmentPNS  -11.6711     7.1094  60.6100  -1.642 0.105842
## Timepointd21:TreatmentPNS   2.4583     6.4508  61.7857   0.381 0.704444
## Timepointd35:TreatmentPNS -16.3042     7.1507  62.0138  -2.280 0.026053 *
## ---
## Signif. codes:  0 '***' 0.001 '**' 0.01 '*' 0.05 '.' 0.1 ' ' 1
##
## Correlation of Fixed Effects:
##             (Intr) Tmpnt7 Tmpn21 Tmpn35 TrtPNS T7:TPN T21:TP
## Timepointd7 -0.503
## Timepontd21 -0.601  0.430
## Timepontd35 -0.533  0.416  0.464
## TreatmntPNS -0.742  0.373  0.446  0.395
## Tmpnt7:TPNS  0.390 -0.776 -0.334 -0.323 -0.536
## Tmpn21:TPNS  0.455 -0.325 -0.757 -0.351 -0.607  0.473
## Tmpn35:TPNS  0.395 -0.309 -0.344 -0.742 -0.536  0.435  0.476
```

```
# Create a type III ANOVA table
Foxp3_anova_table <- anova(Foxp3_model, ddf = "Kenward-Roger")
print(Foxp3_anova_table)
```

```
## Type III Analysis of Variance Table with Kenward-Roger's method
##                     Sum Sq Mean Sq NumDF  DenDF F value    Pr(>F)
## Timepoint           5443.1 1814.36     3 62.821 16.7861 4.054e-08 ***
## Treatment            204.5  204.54     1  9.029  1.8924   0.20209
## Timepoint:Treatment 1044.8  348.27     3 62.821  3.2221   0.02849 *
## ---
## Signif. codes:  0 '***' 0.001 '**' 0.01 '*' 0.05 '.' 0.1 ' ' 1
```

```
# Because we have an interaction
Foxp3_emcatcat <- emmeans(Foxp3_model, ~ Timepoint * Treatment)

#Is there a difference between treatment groups at each time point?
contrast(Foxp3_emcatcat, "revpairwise", by="Timepoint", adjust="BH")
```

```
## Timepoint = d0:
##  contrast      estimate   SE   df t.ratio p.value
##  PNS - Control    0.668 5.79 29.6   0.115  0.9089
##
## Timepoint = d7:
##  contrast      estimate   SE   df t.ratio p.value
##  PNS - Control  -11.003 6.34 36.9  -1.736  0.0909
##
## Timepoint = d21:
##  contrast      estimate   SE   df t.ratio p.value
##  PNS - Control    3.127 5.47 25.0   0.572  0.5724
##
## Timepoint = d35:
##  contrast      estimate   SE   df t.ratio p.value
##  PNS - Control  -15.636 6.39 36.2  -2.449  0.0193
##
## Degrees-of-freedom method: kenward-roger
```

```
#Is there a difference between time points within each treatment?
contrast(Foxp3_emcatcat, "revpairwise", by="Treatment", adjust="BH")
```

```
## Treatment = Control:
##  contrast  estimate   SE   df t.ratio p.value
##  d7 - d0     29.528 5.53 61.2   5.342  <.0001
##  d21 - d0    15.930 4.91 63.4   3.243  0.0038
##  d21 - d7   -13.598 5.64 65.6  -2.412  0.0280
##  d35 - d0    22.025 5.32 61.8   4.139  0.0003
##  d35 - d7    -7.503 5.87 61.6  -1.279  0.2469
##  d35 - d21    6.095 5.33 64.9   1.143  0.2570
##
## Treatment = PNS:
##  contrast  estimate   SE   df t.ratio p.value
##  d7 - d0     17.857 4.50 61.4   3.969  0.0006
##  d21 - d0    18.388 4.23 60.9   4.352  0.0003
##  d21 - d7     0.531 4.20 60.4   0.126  0.8999
##  d35 - d0     5.721 4.83 63.4   1.184  0.2891
##  d35 - d7   -12.136 4.86 63.6  -2.500  0.0225
##  d35 - d21  -12.667 4.62 63.7  -2.745  0.0157
##
## Degrees-of-freedom method: kenward-roger
## P value adjustment: BH method for 6 tests
```

Colon Tissue *Il18* Expression

```
# Fit linear mixed-effects model
# Include random intercept for litter
IL18_model <- lmer(IL18 ~ Timepoint * Treatment + (1 | Litter), data = colon_dat)
summary(IL18_model)
```

```
## Linear mixed model fit by REML. t-tests use Satterthwaite's method [
## lmerModLmerTest]
## Formula: IL18 ~ Timepoint * Treatment + (1 | Litter)
##    Data: colon_dat
##
## REML criterion at convergence: 337.2
##
## Scaled residuals:
##     Min      1Q  Median      3Q     Max
## -1.8285 -0.6083  0.0943  0.4266  3.1900
##
## Random effects:
##  Groups   Name        Variance Std.Dev.
##  Litter   (Intercept) 1.282    1.132
##  Residual             3.989    1.997
## Number of obs: 81, groups:  Litter, 11
##
## Fixed effects:
##                          Estimate Std. Error       df t value Pr(>|t|)
## (Intercept)               1.21942    0.81423 25.19161   1.498 0.146651
## Timepointd7               0.48693    0.99833 65.62828   0.488 0.627357
## Timepointd21              3.32880    0.87879 64.45459   3.788 0.000336 ***
## Timepointd35              3.32432    0.96525 66.20592   3.444 0.000999 ***
## TreatmentPNS             -0.16987    1.10184 25.19034  -0.154 0.878705
## Timepointd7:TreatmentPNS   0.07712    1.30557 65.19148   0.059 0.953074
## Timepointd21:TreatmentPNS -0.11383    1.19101 64.34141  -0.096 0.924156
## Timepointd35:TreatmentPNS -1.09191    1.32103 66.32298  -0.827 0.411452
## ---
## Signif. codes:  0 '***' 0.001 '**' 0.01 '*' 0.05 '.' 0.1 ' ' 1
##
## Correlation of Fixed Effects:
##             (Intr) Tmpnt7 Tmpn21 Tmpn35 TrtPNS T7:TPN T21:TP
## Timepointd7 -0.494
## Timepontd21 -0.568  0.447
## Timepontd35 -0.518  0.443  0.469
## TreatmntPNS -0.739  0.365  0.420  0.383
## Tmpnt7:TPNS  0.377 -0.765 -0.342 -0.339 -0.515
## Tmpn21:TPNS  0.419 -0.330 -0.738 -0.346 -0.567  0.471
## Tmpn35:TPNS  0.379 -0.324 -0.343 -0.731 -0.510  0.440  0.462
```

```
# Create a type III ANOVA table
IL18_anova_table <- anova(IL18_model, ddf = "Kenward-Roger")
print(IL18_anova_table)
```

```
## Type III Analysis of Variance Table with Kenward-Roger's method
##                      Sum Sq Mean Sq NumDF  DenDF F value    Pr(>F)    
## Timepoint           162.277  54.092     3 66.051 13.5616 5.418e-07 ***
## Treatment             1.192   1.192     1  8.976  0.2988    0.5980    
## Timepoint:Treatment   3.689   1.230     3 66.051  0.3083    0.8193    
## ---
## Signif. codes:  0 '***' 0.001 '**' 0.01 '*' 0.05 '.' 0.1 ' ' 1
```

```
#Because no interaction, test for main effects of treatment at individual time points
IL18_emcatcat <- emmeans(IL18_model, ~ Treatment | Timepoint)
#Is there a difference between treatment groups at each time point?
contrast(IL18_emcatcat, "revpairwise", adjust="BH")
```

```
## Timepoint = d0:
##  contrast      estimate   SE   df t.ratio p.value
##  PNS - Control  -0.1699 1.10 26.1  -0.154  0.8788
## 
## Timepoint = d7:
##  contrast      estimate   SE   df t.ratio p.value
##  PNS - Control  -0.0927 1.20 33.0  -0.077  0.9389
## 
## Timepoint = d21:
##  contrast      estimate   SE   df t.ratio p.value
##  PNS - Control  -0.2837 1.07 23.8  -0.265  0.7933
## 
## Timepoint = d35:
##  contrast      estimate   SE   df t.ratio p.value
##  PNS - Control  -1.2618 1.22 33.3  -1.033  0.3090
## 
## Degrees-of-freedom method: kenward-roger
```

Colon Tissue *Il4* Expression

```
# Fit linear mixed-effects model
# Include random intercept for litter
IL4_model <- lmer(IL4 ~ Timepoint * Treatment + (1 | Litter), data = colon_dat)
summary(IL4_model)
```

```
## Linear mixed model fit by REML. t-tests use Satterthwaite's method [
## lmerModLmerTest]
## Formula: IL4 ~ Timepoint * Treatment + (1 | Litter)
##    Data: colon_dat
## 
## REML criterion at convergence: 472.6
## 
## Scaled residuals: 
##     Min      1Q  Median      3Q     Max 
## -2.5161 -0.4325 -0.1828  0.3703  3.7403 
## 
## Random effects:
##  Groups   Name        Variance Std.Dev.
##  Litter   (Intercept)  2.912   1.707   
##  Residual             62.004   7.874   
## Number of obs: 73, groups:  Litter, 11
## 
## Fixed effects:
##                           Estimate Std. Error      df t value Pr(>|t|)    
## (Intercept)                  1.148      3.081  56.007   0.373  0.71088    
## Timepointd7                  5.227      4.221  59.073   1.239  0.22042    
## Timepointd21                 2.126      3.894  59.526   0.546  0.58720    
## Timepointd35                18.825      4.089  59.367   4.604 2.23e-05 ***
## TreatmentPNS                 3.786      3.954  53.208   0.958  0.34257    
## Timepointd7:TreatmentPNS    -6.602      5.397  58.726  -1.223  0.22611    
## Timepointd21:TreatmentPNS   -1.440      5.149  59.390  -0.280  0.78066    
## Timepointd35:TreatmentPNS  -14.752      5.515  61.430  -2.675  0.00957 ** 
## ---
## Signif. codes:  0 '***' 0.001 '**' 0.01 '*' 0.05 '.' 0.1 ' ' 1
## 
## Correlation of Fixed Effects:
##             (Intr) Tmpnt7 Tmpn21 Tmpn35 TrtPNS T7:TPN T21:TP
## Timepointd7 -0.681                                          
## Timepontd21 -0.743  0.535                                   
## Timepontd35 -0.705  0.518  0.554                            
## TreatmntPNS -0.779  0.531  0.579  0.549                     
## Tmpnt7:TPNS  0.533 -0.782 -0.418 -0.405 -0.680              
## Tmpn21:TPNS  0.562 -0.404 -0.756 -0.419 -0.716  0.520       
## Tmpn35:TPNS  0.522 -0.384 -0.411 -0.741 -0.667  0.490  0.507
```

```
# Create a type III ANOVA table
IL4_anova_table <- anova(IL4_model, ddf = "Kenward-Roger")
print(IL4_anova_table)
```

```
## Type III Analysis of Variance Table with Kenward-Roger's method
##                      Sum Sq Mean Sq NumDF  DenDF F value    Pr(>F)
## Timepoint           1279.92  426.64     3 60.619  6.8792 0.0004605 ***
## Treatment             48.45   48.45     1  8.519  0.7814 0.4009776
## Timepoint:Treatment  530.26  176.75     3 60.619  2.8500 0.0447262 *
## ---
## Signif. codes:  0 '***' 0.001 '**' 0.01 '*' 0.05 '.' 0.1 ' ' 1
```

```
# Because we have an interaction
IL4_emcatcat <- emmeans(IL4_model, ~ Timepoint * Treatment)

#Is there a difference between treatment groups at each time point?
contrast(IL4_emcatcat, "revpairwise", by="Timepoint", adjust="BH")
```

```
## Timepoint = d0:
##  contrast      estimate   SE   df t.ratio p.value
##  PNS - Control     3.79 3.97 51.9   0.954  0.3445
##
## Timepoint = d7:
##  contrast      estimate   SE   df t.ratio p.value
##  PNS - Control    -2.82 4.00 49.1  -0.705  0.4844
##
## Timepoint = d21:
##  contrast      estimate   SE   df t.ratio p.value
##  PNS - Control     2.35 3.63 43.2   0.647  0.5212
##
## Timepoint = d35:
##  contrast      estimate   SE   df t.ratio p.value
##  PNS - Control   -10.97 4.19 46.8  -2.619  0.0118
##
## Degrees-of-freedom method: kenward-roger
```

```
#Is there a difference between time points within each treatment?
contrast(IL4_emcatcat, "revpairwise", by="Treatment", adjust="BH")
```

```
## Treatment = Control:
##  contrast  estimate   SE   df t.ratio p.value
##  d7 - d0      5.227 4.24 58.4   1.234  0.3332
##  d21 - d0     2.126 3.91 58.9   0.543  0.5890
##  d21 - d7    -3.102 3.98 63.5  -0.779  0.5266
##  d35 - d0    18.825 4.11 58.7   4.583  0.0001
##  d35 - d7    13.598 4.09 57.0   3.327  0.0031
##  d35 - d21   16.700 3.82 63.1   4.369  0.0001
##
## Treatment = PNS:
##  contrast  estimate   SE   df t.ratio p.value
##  d7 - d0     -1.374 3.37 57.4  -0.407  0.8222
##  d21 - d0     0.685 3.39 58.5   0.202  0.8404
##  d21 - d7     2.060 3.37 57.4   0.611  0.8157
##  d35 - d0     4.073 3.77 63.2   1.081  0.7606
##  d35 - d7     5.448 3.79 63.9   1.439  0.7606
##  d35 - d21    3.388 3.84 64.9   0.883  0.7606
##
## Degrees-of-freedom method: kenward-roger
## P value adjustment: BH method for 6 tests
```

Colon Tissue *Il5* Expression

```
# Fit linear mixed-effects model
# Include random intercept for Litter
IL5_model <- lmer(IL5 ~ Timepoint * Treatment + (1 | Litter), data = colon_dat)
summary(IL5_model)
```

```
## Linear mixed model fit by REML. t-tests use Satterthwaite's method [
## lmerModLmerTest]
## Formula: IL5 ~ Timepoint * Treatment + (1 | Litter)
##    Data: colon_dat
##
## REML criterion at convergence: 214.6
##
## Scaled residuals:
##     Min      1Q  Median      3Q     Max
## -1.6782 -0.1684 -0.0201  0.2237  4.1483
##
## Random effects:
##  Groups   Name        Variance Std.Dev.
##  Litter   (Intercept) 0.3742   0.6117
##  Residual             0.8094   0.8997
## Number of obs: 78, groups:  Litter, 11
##
## Fixed effects:
##                             Estimate Std. Error      df t value Pr(>|t|)
## (Intercept)                   0.9379     0.4096 23.2610   2.290   0.0314 *
## Timepointd7                  -0.5080     0.4650 63.4636  -1.093   0.2787
## Timepointd21                 -0.5067     0.4169 61.5272  -1.215   0.2289
## Timepointd35                 -0.7754     0.4502 63.8299  -1.722   0.0899 .
## TreatmentPNS                  1.3234     0.5465 22.2197   2.421   0.0241 *
## Timepointd7:TreatmentPNS     -1.4750     0.6069 62.7858  -2.430   0.0180 *
## Timepointd21:TreatmentPNS    -1.4341     0.5484 61.4504  -2.615   0.0112 *
## Timepointd35:TreatmentPNS    -0.8705     0.6180 63.6209  -1.408   0.1639
## ---
## Signif. codes:  0 '***' 0.001 '**' 0.01 '*' 0.05 '.' 0.1 ' ' 1
##
## Correlation of Fixed Effects:
##             (Intr) Tmpnt7 Tmpn21 Tmpn35 TrtPNS T7:TPN T21:TP
## Timepointd7 -0.487
## Timepontd21 -0.544  0.479
## Timepontd35 -0.509  0.479  0.500
## TreatmntPNS -0.749  0.365  0.408  0.382
## Tmpnt7:TPNS  0.373 -0.766 -0.367 -0.367 -0.489
## Tmpn21:TPNS  0.414 -0.364 -0.760 -0.380 -0.539  0.488
## Tmpn35:TPNS  0.371 -0.349 -0.364 -0.728 -0.479  0.450  0.476
```

```
# Create a type III ANOVA table
IL5_anova_table <- anova(IL5_model, ddf = "Kenward-Roger")
print(IL5_anova_table)
```

```
## Type III Analysis of Variance Table with Kenward-Roger's method
##                     Sum Sq Mean Sq NumDF  DenDF F value    Pr(>F)
## Timepoint           21.6848  7.2283     3 62.663  8.9301 5.192e-05 ***
## Treatment            0.6309  0.6309     1  9.002  0.7794   0.40028
## Timepoint:Treatment  6.9514  2.3171     3 62.663  2.8627   0.04376 *
## ---
## Signif. codes:  0 '***' 0.001 '**' 0.01 '*' 0.05 '.' 0.1 ' ' 1
```

```
# Because we have an interaction
IL5_emcatcat <- emmeans(IL5_model, ~ Timepoint * Treatment)

#Is there a difference between treatment groups at each time point?
contrast(IL5_emcatcat, "revpairwise", by="Timepoint", adjust="BH")
```

```
## Timepoint = d0:
##  contrast      estimate    SE   df t.ratio p.value
##  PNS - Control    1.323 0.547 22.5   2.419  0.0241
##
## Timepoint = d7:
##  contrast      estimate    SE   df t.ratio p.value
##  PNS - Control   -0.152 0.587 27.6  -0.258  0.7982
##
## Timepoint = d21:
##  contrast      estimate    SE   df t.ratio p.value
##  PNS - Control   -0.111 0.526 19.9  -0.210  0.8354
##
## Timepoint = d35:
##  contrast      estimate    SE   df t.ratio p.value
##  PNS - Control    0.453 0.600 28.7   0.754  0.4567
##
## Degrees-of-freedom method: kenward-roger
```

```
#Is there a difference between time points within each treatment?
contrast(IL5_emcatcat, "revpairwise", by="Treatment", adjust="BH")
```

```
## Treatment = Control:
##  contrast  estimate    SE   df t.ratio p.value
##  d7 - d0   -0.50802 0.467 63.6  -1.087  0.5620
##  d21 - d0  -0.50671 0.418 61.7  -1.214  0.5620
##  d21 - d7   0.00131 0.454 63.0   0.003  0.9977
##  d35 - d0  -0.77536 0.453 63.9  -1.713  0.5497
##  d35 - d7  -0.26734 0.467 61.3  -0.572  0.6834
##  d35 - d21 -0.26865 0.436 63.0  -0.616  0.6834
##
## Treatment = PNS:
##  contrast  estimate    SE   df t.ratio p.value
##  d7 - d0   -1.98300 0.391 61.9  -5.075  <.0001
##  d21 - d0  -1.94081 0.357 61.5  -5.442  <.0001
##  d21 - d7   0.04220 0.374 61.3   0.113  0.9105
##  d35 - d0  -1.64584 0.426 63.5  -3.867  0.0005
##  d35 - d7   0.33717 0.443 63.3   0.761  0.5778
##  d35 - d21  0.29497 0.417 63.9   0.708  0.5778
##
## Degrees-of-freedom method: kenward-roger
## P value adjustment: BH method for 6 tests
```

Colon Tissue *Il13* Expression

```
# Fit linear mixed-effects model
# Include random intercept for litter
IL13_model <- lmer(IL13 ~ Timepoint * Treatment + (1 | Litter), data = colon_dat)
summary(IL13_model)
```

```
## Linear mixed model fit by REML. t-tests use Satterthwaite's method [
## lmerModLmerTest]
## Formula: IL13 ~ Timepoint * Treatment + (1 | Litter)
##    Data: colon_dat
##
## REML criterion at convergence: 55.6
##
## Scaled residuals:
##     Min      1Q  Median      3Q     Max
## -1.7511 -0.5599 -0.1638  0.3296  2.5933
##
## Random effects:
##  Groups   Name        Variance Std.Dev.
##  Litter   (Intercept) 0.009157 0.09569
##  Residual             0.094711 0.30775
## Number of obs: 77, groups:  Litter, 11
##
## Fixed effects:
##                           Estimate Std. Error       df t value Pr(>|t|)
## (Intercept)                1.00628    0.11198 42.75537   8.986 2.10e-11 ***
## Timepointd7               -0.21702    0.15686 64.57800  -1.384    0.171
## Timepointd21              -0.71968    0.14205 61.88586  -5.066 3.92e-06 ***
## Timepointd35              -0.90247    0.15154 65.16975  -5.955 1.15e-07 ***
## TreatmentPNS              -0.10302    0.15101 42.55978  -0.682    0.499
## Timepointd7:TreatmentPNS  -0.01921    0.20742 64.10499  -0.093    0.926
## Timepointd21:TreatmentPNS  0.21668    0.19072 62.68949   1.136    0.260
## Timepointd35:TreatmentPNS  0.13898    0.20617 64.75731   0.674    0.503
## ---
## Signif. codes:  0 '***' 0.001 '**' 0.01 '*' 0.05 '.' 0.1 ' ' 1
##
## Correlation of Fixed Effects:
##             (Intr) Tmpnt7 Tmpn21 Tmpn35 TrtPNS T7:TPN T21:TP
## Timepointd7 -0.608
## Timepontd21 -0.673  0.480
## Timepontd35 -0.634  0.465  0.499
## TreatmntPNS -0.742  0.451  0.499  0.470
## Tmpnt7:TPNS  0.460 -0.756 -0.363 -0.352 -0.622
## Tmpn21:TPNS  0.501 -0.357 -0.745 -0.372 -0.676  0.495
## Tmpn35:TPNS  0.466 -0.342 -0.367 -0.735 -0.622  0.462  0.494
```

```
# Create a type III ANOVA table
IL13_anova_table <- anova(IL13_model, ddf = "Kenward-Roger")
print(IL13_anova_table)
```

```
## Type III Analysis of Variance Table with Kenward-Roger's method
##                     Sum Sq Mean Sq NumDF  DenDF F value    Pr(>F)
## Timepoint           7.6205 2.54017     3 63.507 26.8192 2.517e-11 ***
## Treatment           0.0039 0.00388     1  8.518  0.0410    0.8443
## Timepoint:Treatment 0.1873 0.06245     3 63.507  0.6593    0.5801
## ---
## Signif. codes:  0 '***' 0.001 '**' 0.01 '*' 0.05 '.' 0.1 ' ' 1
```

```
#Because no interaction, test for main effects of treatment at individual time points
IL13_emcatcat <- emmeans(IL13_model, ~ Treatment | Timepoint)
#Is there a difference between treatment groups at each time point?
contrast(IL13_emcatcat, "revpairwise", adjust="BH")
```

```
## Timepoint = d0:
##  contrast      estimate    SE   df t.ratio p.value
##  PNS - Control   -0.103 0.152 42.4  -0.679  0.5011
##
## Timepoint = d7:
##  contrast      estimate    SE   df t.ratio p.value
##  PNS - Control   -0.122 0.165 48.2  -0.741  0.4621
##
## Timepoint = d21:
##  contrast      estimate    SE   df t.ratio p.value
##  PNS - Control    0.114 0.142 38.2   0.798  0.4299
##
## Timepoint = d35:
##  contrast      estimate    SE   df t.ratio p.value
##  PNS - Control    0.036 0.165 44.5   0.218  0.8285
##
## Degrees-of-freedom method: kenward-roger
```

Colon Tissue *Il17a* Expression

```
# Fit linear mixed-effects model
# Include random intercept for litter
IL17_model <- lmer(IL17 ~ Timepoint * Treatment + (1 | Litter), data = colon_dat)
summary(IL17_model)
```

```
## Linear mixed model fit by REML. t-tests use Satterthwaite's method [
## lmerModLmerTest]
## Formula: IL17 ~ Timepoint * Treatment + (1 | Litter)
##    Data: colon_dat
##
## REML criterion at convergence: 99.5
##
## Scaled residuals:
##     Min      1Q  Median      3Q     Max
## -1.4227 -0.6152 -0.1564  0.4513  2.9144
##
## Random effects:
##  Groups   Name        Variance Std.Dev.
##  Litter   (Intercept) 0.1280   0.3578
##  Residual             0.1894   0.4352
## Number of obs: 67, groups:  Litter, 11
##
## Fixed effects:
##                            Estimate Std. Error       df t value Pr(>|t|)
## (Intercept)                 0.75143    0.36198 52.07416   2.076   0.0429 *
## Timepointd7                 0.15605    0.37403 54.11216   0.417   0.6782
## Timepointd21               -0.07882    0.35266 52.25196  -0.223   0.8240
## Timepointd35                0.09803    0.38047 54.93394   0.258   0.7976
## TreatmentPNS               -0.40997    0.41759 42.62333  -0.982   0.3318
## Timepointd7:TreatmentPNS  -0.20569    0.42625 53.30237  -0.483   0.6314
## Timepointd21:TreatmentPNS  0.20003    0.40235 51.84455   0.497   0.6212
## Timepointd35:TreatmentPNS  0.39652    0.43511 54.03644   0.911   0.3662
## ---
## Signif. codes:  0 '***' 0.001 '**' 0.01 '*' 0.05 '.' 0.1 ' ' 1
##
## Correlation of Fixed Effects:
##             (Intr) Tmpnt7 Tmpn21 Tmpn35 TrtPNS T7:TPN T21:TP
## Timepointd7 -0.797
## Timepontd21 -0.823  0.807
## Timepontd35 -0.801  0.806  0.814
## TreatmntPNS -0.867  0.691  0.714  0.694
## Tmpnt7:TPNS  0.699 -0.877 -0.708 -0.707 -0.731
## Tmpn21:TPNS  0.721 -0.708 -0.877 -0.714 -0.758  0.753
## Tmpn35:TPNS  0.700 -0.704 -0.712 -0.874 -0.726  0.732  0.746
```

```
# Create a type III ANOVA table
IL17_anova_table <- anova(IL17_model, ddf = "Kenward-Roger")
print(IL17_anova_table)
```

```
## Type III Analysis of Variance Table with Kenward-Roger's method
##                      Sum Sq Mean Sq NumDF  DenDF F value Pr(>F)
## Timepoint           0.74731 0.24910     3 52.678  1.3153 0.2792
## Treatment           0.29559 0.29559     1  9.876  1.5608 0.2403
## Timepoint:Treatment 0.75034 0.25011     3 52.678  1.3206 0.2775
```

```
#Because no interaction, test for main effects of treatment at individual time points
IL17_emcatcat <- emmeans(IL17_model, ~ Treatment | Timepoint)
#Is there a difference between treatment groups at each time point?
contrast(IL17_emcatcat, "revpairwise", adjust="BH")
```

```
## Timepoint = d0:
##  contrast      estimate    SE   df t.ratio p.value
##  PNS - Control  -0.4100 0.420 43.8  -0.976  0.3343
##
## Timepoint = d7:
##  contrast      estimate    SE   df t.ratio p.value
##  PNS - Control  -0.6157 0.310 21.2  -1.986  0.0601
##
## Timepoint = d21:
##  contrast      estimate    SE   df t.ratio p.value
##  PNS - Control  -0.2099 0.286 16.3  -0.735  0.4730
##
## Timepoint = d35:
##  contrast      estimate    SE   df t.ratio p.value
##  PNS - Control  -0.0135 0.318 22.4  -0.042  0.9666
##
## Degrees-of-freedom method: kenward-roger
```

Sex Differences in Colon Tissue Gene Expression

Colon Tissue *Ifng* Expression Sex Differences

```
# Does colon Ifny expression differ by sex
#Include random intercept for litter
Ifng_sex_effects <- lmer(Ifng ~ Timepoint * Treatment * Sex + (1 | Litter), data = colon_dat)
Ifng_anova_table_for_sex <- anova(Ifng_sex_effects, ddf = "Kenward-Roger")
print(Ifng_anova_table_for_sex)
```

```
## Type III Analysis of Variance Table with Kenward-Roger's method
##                          Sum Sq Mean Sq NumDF  DenDF F value Pr(>F)
## Timepoint               2.12903 1.06452     2 39.946  1.6157 0.2115
## Treatment               1.33896 1.33896     1 10.135  2.0322 0.1841
## Sex                     0.36047 0.36047     1 41.694  0.5471 0.4637
## Timepoint:Treatment     1.97098 0.98549     2 39.946  1.4957 0.2364
## Timepoint:Sex           0.90874 0.45437     2 43.534  0.6892 0.5074
## Treatment:Sex           1.07117 1.07117     1 41.694  1.6258 0.2093
## Timepoint:Treatment:Sex 0.19493 0.09746     2 43.534  0.1478 0.8630
```

```
Ifng_emcatcat_sex <- emmeans(Ifng_sex_effects, ~ Timepoint * Treatment * Sex)
#Is there a difference in males or females between treatment group at each time point?
contrast(Ifng_emcatcat_sex, "revpairwise", by=c("Sex", "Timepoint"), adjust="BH")
```

```
## Sex = F, Timepoint = d7:
##  contrast      estimate    SE   df t.ratio p.value
##  PNS - Control   -0.639 0.671 37.8  -0.952  0.3473
##
## Sex = M, Timepoint = d7:
##  contrast      estimate    SE   df t.ratio p.value
##  PNS - Control    0.507 0.959 45.0   0.529  0.5994
##
## Sex = F, Timepoint = d21:
##  contrast      estimate    SE   df t.ratio p.value
##  PNS - Control   -0.508 0.573 29.4  -0.887  0.3821
##
## Sex = M, Timepoint = d21:
##  contrast      estimate    SE   df t.ratio p.value
##  PNS - Control   -0.141 0.602 30.4  -0.234  0.8164
##
## Sex = F, Timepoint = d35:
##  contrast      estimate    SE   df t.ratio p.value
##  PNS - Control   -1.518 0.734 42.1  -2.068  0.0448
##
## Sex = M, Timepoint = d35:
##  contrast      estimate    SE   df t.ratio p.value
##  PNS - Control   -0.901 0.765 42.9  -1.177  0.2455
##
## Degrees-of-freedom method: kenward-roger
```

```
#Is there a difference in males or females within treatment group at each time point?
contrast(Ifng_emcatcat_sex, "revpairwise", by=c("Treatment", "Timepoint"), adjust="BH")
```

```
## Treatment = Control, Timepoint = d7:
##  contrast estimate    SE   df t.ratio p.value
##  M - F      -0.570 0.940 41.7  -0.607  0.5474
##
## Treatment = PNS, Timepoint = d7:
##  contrast estimate    SE   df t.ratio p.value
##  M - F       0.576 0.628 43.8   0.917  0.3639
##
## Treatment = Control, Timepoint = d21:
##  contrast estimate    SE   df t.ratio p.value
##  M - F      -0.142 0.627 40.4  -0.226  0.8225
##
## Treatment = PNS, Timepoint = d21:
##  contrast estimate    SE   df t.ratio p.value
##  M - F       0.226 0.494 41.3   0.457  0.6500
##
## Treatment = Control, Timepoint = d35:
##  contrast estimate    SE   df t.ratio p.value
##  M - F      -0.971 0.737 42.4  -1.318  0.1946
##
## Treatment = PNS, Timepoint = d35:
##  contrast estimate    SE   df t.ratio p.value
##  M - F      -0.354 0.679 39.6  -0.522  0.6049
##
## Degrees-of-freedom method: kenward-roger
```

Colon Tissue *Ifnb* Expression Sex Differences

```
# Does colon Ifnb expression differ by sex
#Include random intercept for litter
Ifnb_sex_effects <- lmer(Ifnb ~ Timepoint * Treatment * Sex + (1 | Litter), data = colon_dat)
Ifnb_anova_table_for_sex <- anova(Ifnb_sex_effects, ddf = "Kenward-Roger")
print(Ifnb_anova_table_for_sex)
```

```
## Type III Analysis of Variance Table with Kenward-Roger's method
##                          Sum Sq Mean Sq NumDF  DenDF F value  Pr(>F)
## Timepoint               2.21943 1.10972     2 39.352  3.1298 0.05478 .
## Treatment               0.70918 0.70918     1 10.000  2.0002 0.18765
## Sex                     0.34781 0.34781     1 41.088  0.9810 0.32775
## Timepoint:Treatment     2.27962 1.13981     2 39.352  3.2147 0.05091 .
## Timepoint:Sex           0.35634 0.17817     2 42.725  0.5021 0.60877
## Treatment:Sex           0.74249 0.74249     1 41.088  2.0942 0.15545
## Timepoint:Treatment:Sex 0.03096 0.01548     2 42.725  0.0436 0.95735
## ---
## Signif. codes:  0 '***' 0.001 '**' 0.01 '*' 0.05 '.' 0.1 ' ' 1
```

```
Ifnb_emcatcat_sex <- emmeans(Ifnb_sex_effects, ~ Timepoint * Treatment * Sex)
#Is there a difference in males or females between treatment group at each time point?
contrast(Ifnb_emcatcat_sex, "revpairwise", by=c("Sex", "Timepoint"), adjust="BH")
```

```
## Sex = F, Timepoint = d7:
##  contrast      estimate    SE   df t.ratio p.value
##  PNS - Control   -0.313 0.488 37.2  -0.641  0.5253
##
## Sex = M, Timepoint = d7:
##  contrast      estimate    SE   df t.ratio p.value
##  PNS - Control    0.448 0.701 44.0   0.639  0.5264
##
## Sex = F, Timepoint = d21:
##  contrast      estimate    SE   df t.ratio p.value
##  PNS - Control   -0.321 0.429 29.5  -0.747  0.4610
##
## Sex = M, Timepoint = d21:
##  contrast      estimate    SE   df t.ratio p.value
##  PNS - Control    0.127 0.436 30.1   0.292  0.7723
##
## Sex = F, Timepoint = d35:
##  contrast      estimate    SE   df t.ratio p.value
##  PNS - Control   -1.392 0.535 41.5  -2.603  0.0128
##
## Sex = M, Timepoint = d35:
##  contrast      estimate    SE   df t.ratio p.value
##  PNS - Control   -0.822 0.558 42.1  -1.472  0.1483
##
## Degrees-of-freedom method: kenward-roger
```

```
#Is there a difference in males or females within treatment group at each time point?
contrast(Ifnb_emcatcat_sex, "revpairwise", by=c("Treatment", "Timepoint"), adjust="BH")
```

```
## Treatment = Control, Timepoint = d7:
##  contrast estimate    SE   df t.ratio p.value
##  M - F      -0.396 0.690 41.3  -0.573  0.5697
##
## Treatment = PNS, Timepoint = d7:
##  contrast estimate    SE   df t.ratio p.value
##  M - F       0.365 0.460 43.1   0.794  0.4318
##
## Treatment = Control, Timepoint = d21:
##  contrast estimate    SE   df t.ratio p.value
##  M - F      -0.321 0.471 37.5  -0.681  0.5003
##
## Treatment = PNS, Timepoint = d21:
##  contrast estimate    SE   df t.ratio p.value
##  M - F       0.127 0.362 40.6   0.352  0.7266
##
## Treatment = Control, Timepoint = d35:
##  contrast estimate    SE   df t.ratio p.value
##  M - F      -0.782 0.541 42.0  -1.444  0.1561
##
## Treatment = PNS, Timepoint = d35:
##  contrast estimate    SE   df t.ratio p.value
##  M - F      -0.212 0.498 38.8  -0.425  0.6729
##
## Degrees-of-freedom method: kenward-roger
```

Colon Tissue *Isg15* Expression Sex Differences

```
# Does colon Isg15 expression differ by sex
#Include random intercept for litter
Isg15_sex_effects <- lmer(Isg15 ~ Timepoint * Treatment * Sex + (1 | Litter), data = colon_dat)
Isg15_anova_table_for_sex <- anova(Isg15_sex_effects, ddf = "Kenward-Roger")
print(Isg15_anova_table_for_sex)
```

```
## Type III Analysis of Variance Table with Kenward-Roger's method
##                          Sum Sq  Mean Sq NumDF  DenDF F value  Pr(>F)
## Timepoint               0.54345 0.271723     2 40.593  4.2689 0.02077 *
## Treatment               0.00199 0.001987     1 10.065  0.0312 0.86325
## Sex                     0.00046 0.000457     1 41.523  0.0072 0.93291
## Timepoint:Treatment     0.01177 0.005884     2 40.593  0.0924 0.91189
## Timepoint:Sex           0.08089 0.040447     2 43.233  0.6352 0.53468
## Treatment:Sex           0.01320 0.013205     1 41.523  0.2075 0.65114
## Timepoint:Treatment:Sex 0.11333 0.056666     2 43.233  0.8900 0.41805
## ---
## Signif. codes:  0 '***' 0.001 '**' 0.01 '*' 0.05 '.' 0.1 ' ' 1
```

```
Isg15_emcatcat_sex <- emmeans(Isg15_sex_effects, ~ Timepoint * Treatment * Sex)
#Is there a difference in males or females between treatment group at each time point?
contrast(Isg15_emcatcat_sex, "revpairwise", by=c("Sex", "Timepoint"), adjust="BH")
```

```
## Sex = F, Timepoint = d7:
##  contrast      estimate    SE   df t.ratio p.value
##  PNS - Control  -0.0719 0.212 30.3  -0.339  0.7367
##
## Sex = M, Timepoint = d7:
##  contrast      estimate    SE   df t.ratio p.value
##  PNS - Control  -0.0466 0.313 46.2  -0.149  0.8822
##
## Sex = F, Timepoint = d21:
##  contrast      estimate    SE   df t.ratio p.value
##  PNS - Control   0.2077 0.202 25.6   1.027  0.3139
##
## Sex = M, Timepoint = d21:
##  contrast      estimate    SE   df t.ratio p.value
##  PNS - Control  -0.1690 0.211 27.3  -0.800  0.4304
##
## Sex = F, Timepoint = d35:
##  contrast      estimate    SE   df t.ratio p.value
##  PNS - Control  -0.0966 0.246 39.4  -0.392  0.6969
##
## Sex = M, Timepoint = d35:
##  contrast      estimate    SE   df t.ratio p.value
##  PNS - Control   0.0211 0.250 40.0   0.085  0.9330
##
## Degrees-of-freedom method: kenward-roger
```

```
#Is there a difference in males or females within treatment group at each time point?
contrast(Isg15_emcatcat_sex, "revpairwise", by=c("Treatment", "Timepoint"), adjust="BH")
```

```
## Treatment = Control, Timepoint = d7:
##  contrast estimate    SE   df t.ratio p.value
##  M - F    -0.07128 0.294 41.5  -0.243  0.8096
##
## Treatment = PNS, Timepoint = d7:
##  contrast estimate    SE   df t.ratio p.value
##  M - F    -0.04599 0.175 42.4  -0.262  0.7944
##
## Treatment = Control, Timepoint = d21:
##  contrast estimate    SE   df t.ratio p.value
##  M - F     0.32623 0.206 46.8   1.587  0.1193
##
## Treatment = PNS, Timepoint = d21:
##  contrast estimate    SE   df t.ratio p.value
##  M - F    -0.05044 0.157 42.0  -0.321  0.7498
##
## Treatment = Control, Timepoint = d35:
##  contrast estimate    SE   df t.ratio p.value
##  M - F    -0.11641 0.218 40.6  -0.533  0.5970
##
## Treatment = PNS, Timepoint = d35:
##  contrast estimate    SE   df t.ratio p.value
##  M - F     0.00133 0.212 40.1   0.006  0.9950
##
## Degrees-of-freedom method: kenward-roger
```

Colon Tissue *Il1b* Expression Sex Differences

```
# Does colon Il1b expression differ by sex
#Include random intercept for litter
IL1b_sex_effects <- lmer(IL1b ~ Timepoint * Treatment * Sex + (1 | Litter), data = colon_dat)
IL1b_anova_table_for_sex <- anova(IL1b_sex_effects, ddf = "Kenward-Roger")
print(IL1b_anova_table_for_sex)
```

```
## Type III Analysis of Variance Table with Kenward-Roger's method
##                         Sum Sq Mean Sq NumDF  DenDF F value Pr(>F)
## Timepoint              18.6593  9.3296     2 40.567  1.9344 0.1576
## Treatment               8.0025  8.0025     1 10.238  1.6592 0.2261
## Sex                     0.7613  0.7613     1 41.913  0.1578 0.6932
## Timepoint:Treatment     2.5102  1.2551     2 40.567  0.2602 0.7722
## Timepoint:Sex           0.3529  0.1765     2 43.093  0.0366 0.9641
## Treatment:Sex           0.7662  0.7662     1 41.913  0.1589 0.6922
## Timepoint:Treatment:Sex  0.3113  0.1557     2 43.093  0.0323 0.9683
```

```
IL1b_emcatcat_sex <- emmeans(IL1b_sex_effects, ~ Timepoint * Treatment * Sex)
#Is there a difference in males or females between treatment group at each time point?
contrast(IL1b_emcatcat_sex, "revpairwise", by=c("Sex", "Timepoint"), adjust="BH")
```

```
## Sex = F, Timepoint = d7:
##  contrast      estimate   SE   df t.ratio p.value
##  PNS - Control   -1.425 1.74 33.4  -0.819  0.4183
##
## Sex = M, Timepoint = d7:
##  contrast      estimate   SE   df t.ratio p.value
##  PNS - Control   -0.473 2.65 45.8  -0.178  0.8592
##
## Sex = F, Timepoint = d21:
##  contrast      estimate   SE   df t.ratio p.value
##  PNS - Control   -2.518 1.74 30.9  -1.444  0.1589
##
## Sex = M, Timepoint = d21:
##  contrast      estimate   SE   df t.ratio p.value
##  PNS - Control   -1.726 1.70 28.7  -1.015  0.3186
##
## Sex = F, Timepoint = d35:
##  contrast      estimate   SE   df t.ratio p.value
##  PNS - Control   -1.320 2.08 42.2  -0.634  0.5296
##
## Sex = M, Timepoint = d35:
##  contrast      estimate   SE   df t.ratio p.value
##  PNS - Control   -1.261 2.10 41.7  -0.602  0.5507
##
## Degrees-of-freedom method: kenward-roger
```

```
#Is there a difference in males or females within treatment group at each time point?
contrast(IL1b_emcatcat_sex, "revpairwise", by=c("Treatment", "Timepoint"), adjust="BH")
```

```
## Treatment = Control, Timepoint = d7:
##  contrast estimate   SE   df t.ratio p.value
##  M - F     -0.7261 2.55 41.4  -0.285  0.7773
##
## Treatment = PNS, Timepoint = d7:
##  contrast estimate   SE   df t.ratio p.value
##  M - F      0.2265 1.52 42.4   0.149  0.8819
##
## Treatment = Control, Timepoint = d21:
##  contrast estimate   SE   df t.ratio p.value
##  M - F     -0.9472 1.74 44.6  -0.543  0.5896
##
## Treatment = PNS, Timepoint = d21:
##  contrast estimate   SE   df t.ratio p.value
##  M - F     -0.1554 1.45 42.4  -0.107  0.9149
##
## Treatment = Control, Timepoint = d35:
##  contrast estimate   SE   df t.ratio p.value
##  M - F     -0.1272 1.90 40.4  -0.067  0.9469
##
## Treatment = PNS, Timepoint = d35:
##  contrast estimate   SE   df t.ratio p.value
##  M - F     -0.0682 1.91 41.0  -0.036  0.9717
##
## Degrees-of-freedom method: kenward-roger
```

Colon Tissue *Il6* Expression Sex Differences

```
# Does colon IL6 expression differ by sex
#Include random intercept for litter
IL6_sex_effects <- lmer(IL6 ~ Timepoint * Treatment * Sex + (1 | Litter), data = colon_dat)
IL6_anova_table_for_sex <- anova(IL6_sex_effects, ddf = "Kenward-Roger")
print(IL6_anova_table_for_sex)
```

```
## Type III Analysis of Variance Table with Kenward-Roger's method
##                          Sum Sq  Mean Sq NumDF  DenDF F value Pr(>F)
## Timepoint              0.169754 0.084877     2 41.317  0.8015 0.4555
## Treatment              0.192480 0.192480     1 10.021  1.8176 0.2073
## Sex                    0.024200 0.024200     1 41.774  0.2285 0.6351
## Timepoint:Treatment    0.246006 0.123003     2 41.317  1.1615 0.3230
## Timepoint:Sex          0.227170 0.113585     2 42.347  1.0708 0.3518
## Treatment:Sex          0.004126 0.004126     1 41.774  0.0390 0.8445
## Timepoint:Treatment:Sex 0.095903 0.047951     2 42.347  0.4521 0.6393
```

```
IL6_emcatcat_sex <- emmeans(IL6_sex_effects, ~ Timepoint * Treatment * Sex)
#Is there a difference in males or females between treatment group at each time point?
contrast(IL6_emcatcat_sex, "revpairwise", by=c("Sex", "Timepoint"), adjust="BH")
```

```
## Sex = F, Timepoint = d7:
##  contrast      estimate    SE   df t.ratio p.value
##  PNS - Control  -0.2574 0.234 39.4  -1.099  0.2786
##
## Sex = M, Timepoint = d7:
##  contrast      estimate    SE   df t.ratio p.value
##  PNS - Control   0.0573 0.371 43.9   0.154  0.8781
##
## Sex = F, Timepoint = d21:
##  contrast      estimate    SE   df t.ratio p.value
##  PNS - Control   0.0931 0.214 32.8   0.435  0.6665
##
## Sex = M, Timepoint = d21:
##  contrast      estimate    SE   df t.ratio p.value
##  PNS - Control  -0.1127 0.218 32.3  -0.516  0.6092
##
## Sex = F, Timepoint = d35:
##  contrast      estimate    SE   df t.ratio p.value
##  PNS - Control  -0.3888 0.283 42.9  -1.375  0.1762
##
## Sex = M, Timepoint = d35:
##  contrast      estimate    SE   df t.ratio p.value
##  PNS - Control  -0.3691 0.283 42.9  -1.306  0.1984
##
## Degrees-of-freedom method: kenward-roger
```

```
#Is there a difference in males or females within treatment group at each time point?
contrast(IL6_emcatcat_sex, "revpairwise", by=c("Treatment", "Timepoint"), adjust="BH")
```

```
## Treatment = Control, Timepoint = d7:
##  contrast estimate    SE   df t.ratio p.value
##  M - F     -0.2553 0.376 42.8  -0.680  0.5004
##
## Treatment = PNS, Timepoint = d7:
##  contrast estimate    SE   df t.ratio p.value
##  M - F      0.0594 0.221 44.0   0.268  0.7898
##
## Treatment = Control, Timepoint = d21:
##  contrast estimate    SE   df t.ratio p.value
##  M - F      0.2480 0.237 32.7   1.045  0.3035
##
## Treatment = PNS, Timepoint = d21:
##  contrast estimate    SE   df t.ratio p.value
##  M - F      0.0422 0.196 42.9   0.215  0.8305
##
## Treatment = Control, Timepoint = d35:
##  contrast estimate    SE   df t.ratio p.value
##  M - F     -0.2126 0.279 42.3  -0.762  0.4501
##
## Treatment = PNS, Timepoint = d35:
##  contrast estimate    SE   df t.ratio p.value
##  M - F     -0.1930 0.274 39.9  -0.704  0.4856
##
## Degrees-of-freedom method: kenward-roger
```

Colon Tissue *Tnfa* Expression Sex Differences

```
# Does colon Tnf expression differ by sex
#Include random intercept for litter
Tnf_sex_effects <- lmer(Tnf ~ Timepoint * Treatment * Sex + (1 | Litter), data = colon_dat)
Tnf_anova_table_for_sex <- anova(Tnf_sex_effects, ddf = "Kenward-Roger")
print(Tnf_anova_table_for_sex)
```

```
## Type III Analysis of Variance Table with Kenward-Roger's method
##                         Sum Sq Mean Sq NumDF  DenDF F value    Pr(>F)
## Timepoint               8.5622  4.2811     2 40.132 13.4678 3.348e-05 ***
## Treatment               0.0336  0.0336     1 10.892  0.1056    0.7514
## Sex                     0.0609  0.0609     1 40.156  0.1915    0.6640
## Timepoint:Treatment     0.5263  0.2631     2 40.132  0.8278    0.4443
## Timepoint:Sex           0.0795  0.0397     2 42.038  0.1249    0.8829
## Treatment:Sex           0.0353  0.0353     1 40.156  0.1111    0.7407
## Timepoint:Treatment:Sex 0.0108  0.0054     2 42.038  0.0170    0.9832
## ---
## Signif. codes:  0 '***' 0.001 '**' 0.01 '*' 0.05 '.' 0.1 ' ' 1
```

```
Tnf_emcatcat_sex <- emmeans(Tnf_sex_effects, ~ Timepoint * Treatment * Sex)
#Is there a difference in males or females between treatment group at each time point?
contrast(Tnf_emcatcat_sex, "revpairwise", by=c("Sex", "Timepoint"), adjust="BH")
```

```
## Sex = F, Timepoint = d7:
##  contrast      estimate    SE   df t.ratio p.value
##  PNS - Control   0.4930 0.457 31.4   1.078  0.2891
##
## Sex = M, Timepoint = d7:
##  contrast      estimate    SE   df t.ratio p.value
##  PNS - Control   0.3279 0.693 44.6   0.474  0.6382
##
## Sex = F, Timepoint = d21:
##  contrast      estimate    SE   df t.ratio p.value
##  PNS - Control  -0.1599 0.436 26.2  -0.367  0.7165
##
## Sex = M, Timepoint = d21:
##  contrast      estimate    SE   df t.ratio p.value
##  PNS - Control  -0.1957 0.489 31.7  -0.401  0.6915
##
## Sex = F, Timepoint = d35:
##  contrast      estimate    SE   df t.ratio p.value
##  PNS - Control   0.1800 0.699 44.2   0.257  0.7981
##
## Sex = M, Timepoint = d35:
##  contrast      estimate    SE   df t.ratio p.value
##  PNS - Control  -0.0353 0.545 40.2  -0.065  0.9487
##
## Degrees-of-freedom method: kenward-roger
```

```
#Is there a difference in males or females within treatment group at each time point?
contrast(Tnf_emcatcat_sex, "revpairwise", by=c("Treatment", "Timepoint"), adjust="BH")
```

```
## Treatment = Control, Timepoint = d7:
##  contrast estimate    SE   df t.ratio p.value
##  M - F      0.0286 0.663 40.9   0.043  0.9658
##
## Treatment = PNS, Timepoint = d7:
##  contrast estimate    SE   df t.ratio p.value
##  M - F     -0.1365 0.389 40.6  -0.351  0.7277
##
## Treatment = Control, Timepoint = d21:
##  contrast estimate    SE   df t.ratio p.value
##  M - F      0.1304 0.492 44.4   0.265  0.7922
##
## Treatment = PNS, Timepoint = d21:
##  contrast estimate    SE   df t.ratio p.value
##  M - F      0.0946 0.344 39.6   0.275  0.7850
##
## Treatment = Control, Timepoint = d35:
##  contrast estimate    SE   df t.ratio p.value
##  M - F      0.3224 0.659 40.4   0.489  0.6276
##
## Treatment = PNS, Timepoint = d35:
##  contrast estimate    SE   df t.ratio p.value
##  M - F      0.1071 0.472 38.2   0.227  0.8219
##
## Degrees-of-freedom method: kenward-roger
```

Colon Tissue *Ahr* Expression Sex Differences

```
# Does colon Ahr expression differ by sex
#Include random intercept for litter
Ahr_sex_effects <- lmer(Ahr ~ Timepoint * Treatment * Sex + (1 | Litter), data = colon_dat)
Ahr_anova_table_for_sex <- anova(Ahr_sex_effects, ddf = "Kenward-Roger")
print(Ahr_anova_table_for_sex)
```

```
## Type III Analysis of Variance Table with Kenward-Roger's method
##                         Sum Sq Mean Sq NumDF  DenDF F value  Pr(>F)
## Timepoint               9.1521  4.5760     2 41.000  3.6188 0.03570 *
## Treatment               6.6776  6.6776     1 10.207  5.2807 0.04391 *
## Sex                     1.4382  1.4382     1 42.027  1.1373 0.29230
## Timepoint:Treatment     2.8951  1.4475     2 41.000  1.1447 0.32828
## Timepoint:Sex           4.4349  2.2174     2 43.782  1.7529 0.18521
## Treatment:Sex           0.0545  0.0545     1 42.027  0.0431 0.83647
## Timepoint:Treatment:Sex 7.0878  3.5439     2 43.782  2.8015 0.07163 .
## ---
## Signif. codes:  0 '***' 0.001 '**' 0.01 '*' 0.05 '.' 0.1 ' ' 1
```

```
Ahr_emcatcat_sex <- emmeans(Ahr_sex_effects, ~ Timepoint * Treatment * Sex)
#Is there a difference in males or females between treatment group at each time point?
contrast(Ahr_emcatcat_sex, "revpairwise", by=c("Sex", "Timepoint"), adjust="BH")
```

```
## Sex = F, Timepoint = d7:
##  contrast      estimate    SE   df t.ratio p.value
##  PNS - Control   -3.086 0.911 32.6  -3.388  0.0019
##
## Sex = M, Timepoint = d7:
##  contrast      estimate    SE   df t.ratio p.value
##  PNS - Control   -0.521 1.370 46.6  -0.380  0.7056
##
## Sex = F, Timepoint = d21:
##  contrast      estimate    SE   df t.ratio p.value
##  PNS - Control    0.370 0.864 27.4   0.429  0.6716
##
## Sex = M, Timepoint = d21:
##  contrast      estimate    SE   df t.ratio p.value
##  PNS - Control   -1.717 0.905 29.0  -1.898  0.0677
##
## Sex = F, Timepoint = d35:
##  contrast      estimate    SE   df t.ratio p.value
##  PNS - Control   -1.220 1.070 41.3  -1.142  0.2601
##
## Sex = M, Timepoint = d35:
##  contrast      estimate    SE   df t.ratio p.value
##  PNS - Control   -2.172 1.080 41.9  -2.003  0.0517
##
## Degrees-of-freedom method: kenward-roger
```

```
#Is there a difference in males or females within treatment group at each time point?
contrast(Ahr_emcatcat_sex, "revpairwise", by=c("Treatment", "Timepoint"), adjust="BH")
```

```
## Treatment = Control, Timepoint = d7:
##  contrast estimate    SE   df t.ratio p.value
##  M - F      -1.760 1.310 42.1  -1.347  0.1853
##
## Treatment = PNS, Timepoint = d7:
##  contrast estimate    SE   df t.ratio p.value
##  M - F       0.805 0.780 43.1   1.032  0.3078
##
## Treatment = Control, Timepoint = d21:
##  contrast estimate    SE   df t.ratio p.value
##  M - F       1.482 0.902 46.1   1.643  0.1072
##
## Treatment = PNS, Timepoint = d21:
##  contrast estimate    SE   df t.ratio p.value
##  M - F      -0.605 0.699 42.7  -0.866  0.3915
##
## Treatment = Control, Timepoint = d35:
##  contrast estimate    SE   df t.ratio p.value
##  M - F      -0.701 0.973 41.0  -0.721  0.4749
##
## Treatment = PNS, Timepoint = d35:
##  contrast estimate    SE   df t.ratio p.value
##  M - F      -1.653 0.944 40.5  -1.751  0.0875
##
## Degrees-of-freedom method: kenward-roger
```

Colon Tissue *Muc2* Expression Sex Differences

```
# Does colon Muc2 expression differ by sex
#Include random intercept for litter
Muc2_sex_effects <- lmer(Muc2 ~ Timepoint * Treatment * Sex + (1 | Litter), data = colon_dat)
Muc2_anova_table_for_sex <- anova(Muc2_sex_effects, ddf = "Kenward-Roger")
print(Muc2_anova_table_for_sex)
```

```
## Type III Analysis of Variance Table with Kenward-Roger's method
##                         Sum Sq Mean Sq NumDF  DenDF F value    Pr(>F)
## Timepoint               3979.2 1989.61     2 40.708 15.0328 1.292e-05 ***
## Treatment                  0.2    0.15     1 10.638  0.0011    0.9737
## Sex                        0.6    0.63     1 41.066  0.0047    0.9454
## Timepoint:Treatment      114.0   57.00     2 40.708  0.4306    0.6530
## Timepoint:Sex             92.9   46.47     2 44.002  0.3509    0.7060
## Treatment:Sex             80.8   80.77     1 41.066  0.6103    0.4392
## Timepoint:Treatment:Sex  115.6   57.78     2 44.002  0.4363    0.6492
## ---
## Signif. codes:  0 '***' 0.001 '**' 0.01 '*' 0.05 '.' 0.1 ' ' 1
```

```
Muc2_emcatcat_sex <- emmeans(Muc2_sex_effects, ~ Timepoint * Treatment * Sex)
#Is there a difference in males or females between treatment group at each time point?
contrast(Muc2_emcatcat_sex, "revpairwise", by=c("Sex", "Timepoint"), adjust="BH")
```

```
## Sex = F, Timepoint = d7:
##  contrast      estimate    SE   df t.ratio p.value
##  PNS - Control     3.71  9.00 37.6   0.412  0.6824
##
## Sex = M, Timepoint = d7:
##  contrast      estimate    SE   df t.ratio p.value
##  PNS - Control     2.25 13.60 46.0   0.165  0.8694
##
## Sex = F, Timepoint = d21:
##  contrast      estimate    SE   df t.ratio p.value
##  PNS - Control     1.98  8.20 30.3   0.242  0.8107
##
## Sex = M, Timepoint = d21:
##  contrast      estimate    SE   df t.ratio p.value
##  PNS - Control     1.54  9.19 35.0   0.168  0.8676
##
## Sex = F, Timepoint = d35:
##  contrast      estimate    SE   df t.ratio p.value
##  PNS - Control     2.81 10.50 43.0   0.269  0.7893
##
## Sex = M, Timepoint = d35:
##  contrast      estimate    SE   df t.ratio p.value
##  PNS - Control   -13.40 10.60 43.6  -1.264  0.2131
##
## Degrees-of-freedom method: kenward-roger
```

```
#Is there a difference in males or females within treatment group at each time point?
contrast(Muc2_emcatcat_sex, "revpairwise", by=c("Treatment", "Timepoint"), adjust="BH")
```

```
## Treatment = Control, Timepoint = d7:
##  contrast estimate    SE   df t.ratio p.value
##  M - F       -1.49 13.40 42.1  -0.111  0.9125
##
## Treatment = PNS, Timepoint = d7:
##  contrast estimate    SE   df t.ratio p.value
##  M - F       -2.94  7.90 43.3  -0.373  0.7113
##
## Treatment = Control, Timepoint = d21:
##  contrast estimate    SE   df t.ratio p.value
##  M - F       -1.67  9.58 44.2  -0.174  0.8624
##
## Treatment = PNS, Timepoint = d21:
##  contrast estimate    SE   df t.ratio p.value
##  M - F       -2.11  6.91 42.7  -0.305  0.7617
##
## Treatment = Control, Timepoint = d35:
##  contrast estimate    SE   df t.ratio p.value
##  M - F       13.01  9.97 41.7   1.306  0.1988
##
## Treatment = PNS, Timepoint = d35:
##  contrast estimate    SE   df t.ratio p.value
##  M - F       -3.21  9.62 40.2  -0.333  0.7407
##
## Degrees-of-freedom method: kenward-roger
```

Colon Tissue *Il10* Expression Sex Differences

```
# Does colon IL10 expression differ by sex
#Include random intercept for litter
IL10_sex_effects <- lmer(IL10 ~ Timepoint * Treatment * Sex + (1 | Litter), data = colon_dat)
IL10_anova_table_for_sex <- anova(IL10_sex_effects, ddf = "Kenward-Roger")
print(IL10_anova_table_for_sex)
```

```
## Type III Analysis of Variance Table with Kenward-Roger's method
##                         Sum Sq Mean Sq NumDF  DenDF F value   Pr(>F)
## Timepoint               82.872  41.436     2 38.603  7.1002 0.002367 **
## Treatment                8.277   8.277     1  9.662  1.4184 0.262107
## Sex                      2.741   2.741     1 39.175  0.4697 0.497139
## Timepoint:Treatment      2.649   1.324     2 38.603  0.2269 0.798020
## Timepoint:Sex            8.079   4.040     2 40.567  0.6921 0.506336
## Treatment:Sex            8.699   8.699     1 39.175  1.4907 0.229411
## Timepoint:Treatment:Sex  0.655   0.327     2 40.567  0.0561 0.945533
## ---
## Signif. codes:  0 '***' 0.001 '**' 0.01 '*' 0.05 '.' 0.1 ' ' 1
```

```
IL10_emcatcat_sex <- emmeans(IL10_sex_effects, ~ Timepoint * Treatment * Sex)
#Is there a difference in males or females between treatment group at each time point?
contrast(IL10_emcatcat_sex, "revpairwise", by=c("Sex", "Timepoint"), adjust="BH")
```

```
## Sex = F, Timepoint = d7:
##  contrast      estimate   SE   df t.ratio p.value
##  PNS - Control   -0.914 2.32 23.0  -0.394  0.6975
##
## Sex = M, Timepoint = d7:
##  contrast      estimate   SE   df t.ratio p.value
##  PNS - Control   -2.387 3.20 41.8  -0.745  0.4604
##
## Sex = F, Timepoint = d21:
##  contrast      estimate   SE   df t.ratio p.value
##  PNS - Control   -0.440 2.25 20.1  -0.196  0.8469
##
## Sex = M, Timepoint = d21:
##  contrast      estimate   SE   df t.ratio p.value
##  PNS - Control   -3.222 2.33 21.8  -1.384  0.1804
##
## Sex = F, Timepoint = d35:
##  contrast      estimate   SE   df t.ratio p.value
##  PNS - Control   -2.033 2.64 31.9  -0.770  0.4472
##
## Sex = M, Timepoint = d35:
##  contrast      estimate   SE   df t.ratio p.value
##  PNS - Control   -3.818 2.65 31.8  -1.442  0.1589
##
## Degrees-of-freedom method: kenward-roger
```

```
#Is there a difference in males or females within treatment group at each time point?
contrast(IL10_emcatcat_sex, "revpairwise", by=c("Treatment", "Timepoint"), adjust="BH")
```

```
## Treatment = Control, Timepoint = d7:
##  contrast estimate   SE   df t.ratio p.value
##  M - F       0.914 2.83 39.1   0.323  0.7482
##
## Treatment = PNS, Timepoint = d7:
##  contrast estimate   SE   df t.ratio p.value
##  M - F      -0.559 1.69 39.7  -0.330  0.7431
##
## Treatment = Control, Timepoint = d21:
##  contrast estimate   SE   df t.ratio p.value
##  M - F       1.452 2.04 45.4   0.711  0.4809
##
## Treatment = PNS, Timepoint = d21:
##  contrast estimate   SE   df t.ratio p.value
##  M - F      -1.330 1.52 39.6  -0.874  0.3873
##
## Treatment = Control, Timepoint = d35:
##  contrast estimate   SE   df t.ratio p.value
##  M - F      -1.041 2.10 38.5  -0.497  0.6224
##
## Treatment = PNS, Timepoint = d35:
##  contrast estimate   SE   df t.ratio p.value
##  M - F      -2.827 2.05 38.0  -1.376  0.1768
##
## Degrees-of-freedom method: kenward-roger
```

Colon Tissue *Tgfb1* Expression Sex Differences

```
# Does colon Tgfb1 expression differ by sex
#Include random intercept for litter
Tgfb1_sex_effects <- lmer(Tgfb1 ~ Timepoint * Treatment * Sex + (1 | Litter), data = colon_dat)
Tgfb1_anova_table_for_sex <- anova(Tgfb1_sex_effects, ddf = "Kenward-Roger")
print(Tgfb1_anova_table_for_sex)
```

```
## Type III Analysis of Variance Table with Kenward-Roger's method
##                          Sum Sq Mean Sq NumDF  DenDF F value    Pr(>F)
## Timepoint               1.57452 0.78726     2 40.574 19.6028 1.104e-06 ***
## Treatment               0.05041 0.05041     1  9.701  1.2552    0.2895
## Sex                     0.00354 0.00354     1 40.985  0.0882    0.7680
## Timepoint:Treatment     0.04755 0.02378     2 40.574  0.5921    0.5579
## Timepoint:Sex           0.03276 0.01638     2 42.585  0.4078    0.6677
## Treatment:Sex           0.01317 0.01317     1 40.985  0.3279    0.5700
## Timepoint:Treatment:Sex 0.01634 0.00817     2 42.585  0.2034    0.8168
## ---
## Signif. codes:  0 '***' 0.001 '**' 0.01 '*' 0.05 '.' 0.1 ' ' 1
```

```
Tgfb1_emcatcat_sex <- emmeans(Tgfb1_sex_effects, ~ Timepoint * Treatment * Sex)
#Is there a difference in males or females between treatment group at each time point?
contrast(Tgfb1_emcatcat_sex, "revpairwise", by=c("Sex", "Timepoint"), adjust="BH")
```

```
## Sex = F, Timepoint = d7:
##  contrast      estimate    SE   df t.ratio p.value
##  PNS - Control  -0.2240 0.195 22.7  -1.149  0.2626
##
## Sex = M, Timepoint = d7:
##  contrast      estimate    SE   df t.ratio p.value
##  PNS - Control  -0.3324 0.268 42.5  -1.242  0.2211
##
## Sex = F, Timepoint = d21:
##  contrast      estimate    SE   df t.ratio p.value
##  PNS - Control  -0.0326 0.189 19.9  -0.172  0.8651
##
## Sex = M, Timepoint = d21:
##  contrast      estimate    SE   df t.ratio p.value
##  PNS - Control  -0.1926 0.194 21.1  -0.992  0.3324
##
## Sex = F, Timepoint = d35:
##  contrast      estimate    SE   df t.ratio p.value
##  PNS - Control  -0.1384 0.219 30.9  -0.631  0.5325
##
## Sex = M, Timepoint = d35:
##  contrast      estimate    SE   df t.ratio p.value
##  PNS - Control  -0.1022 0.222 31.6  -0.461  0.6481
##
## Degrees-of-freedom method: kenward-roger
```

```
#Is there a difference in males or females within treatment group at each time point?
contrast(Tgfb1_emcatcat_sex, "revpairwise", by=c("Treatment", "Timepoint"), adjust="BH")
```

```
## Treatment = Control, Timepoint = d7:
##  contrast estimate    SE   df t.ratio p.value
##  M - F      0.0854 0.235 41.1   0.364  0.7179
##
## Treatment = PNS, Timepoint = d7:
##  contrast estimate    SE   df t.ratio p.value
##  M - F     -0.0231 0.139 41.3  -0.166  0.8693
##
## Treatment = Control, Timepoint = d21:
##  contrast estimate    SE   df t.ratio p.value
##  M - F      0.1652 0.170 47.4   0.973  0.3356
##
## Treatment = PNS, Timepoint = d21:
##  contrast estimate    SE   df t.ratio p.value
##  M - F      0.0051 0.122 41.2   0.042  0.9669
##
## Treatment = Control, Timepoint = d35:
##  contrast estimate    SE   df t.ratio p.value
##  M - F     -0.0742 0.174 40.5  -0.426  0.6723
##
## Treatment = PNS, Timepoint = d35:
##  contrast estimate    SE   df t.ratio p.value
##  M - F     -0.0380 0.168 40.0  -0.226  0.8227
##
## Degrees-of-freedom method: kenward-roger
```

Colon Tissue *Foxp3* Expression Sex Differences

```
# Does colon Foxp3 expression differ by sex
#Include random intercept for litter
Foxp3_sex_effects <- lmer(Foxp3 ~ Timepoint * Treatment * Sex + (1 | Litter), data = colon_dat)
Foxp3_anova_table_for_sex <- anova(Foxp3_sex_effects, ddf = "Kenward-Roger")
print(Foxp3_anova_table_for_sex)
```

```
## Type III Analysis of Variance Table with Kenward-Roger's method
##                         Sum Sq Mean Sq NumDF  DenDF F value  Pr(>F)
## Timepoint               603.23  301.62     2 38.898  2.4819 0.09673 .
## Treatment               290.93  290.93     1 10.120  2.3940 0.15248
## Sex                       3.50    3.50     1 39.978  0.0288 0.86616
## Timepoint:Treatment     645.24  322.62     2 38.898  2.6547 0.08304 .
## Timepoint:Sex           207.76  103.88     2 42.173  0.8544 0.43277
## Treatment:Sex           163.21  163.21     1 39.978  1.3430 0.25339
## Timepoint:Treatment:Sex 538.42  269.21     2 42.173  2.2143 0.12178
## ---
## Signif. codes:  0 '***' 0.001 '**' 0.01 '*' 0.05 '.' 0.1 ' ' 1
```

```
Foxp3_emcatcat_sex <- emmeans(Foxp3_sex_effects, ~ Timepoint * Treatment * Sex)
#Is there a difference in males or females between treatment group at each time point?
contrast(Foxp3_emcatcat_sex, "revpairwise", by=c("Sex", "Timepoint"), adjust="BH")
```

```
## Sex = F, Timepoint = d7:
##  contrast      estimate    SE   df t.ratio p.value
##  PNS - Control   -14.67  9.24 32.5  -1.587  0.1223
##
## Sex = M, Timepoint = d7:
##  contrast      estimate    SE   df t.ratio p.value
##  PNS - Control    -3.11 13.50 44.7  -0.230  0.8188
##
## Sex = F, Timepoint = d21:
##  contrast      estimate    SE   df t.ratio p.value
##  PNS - Control    15.08  8.57 26.0   1.760  0.0902
##
## Sex = M, Timepoint = d21:
##  contrast      estimate    SE   df t.ratio p.value
##  PNS - Control   -14.84  9.51 30.9  -1.561  0.1287
##
## Sex = F, Timepoint = d35:
##  contrast      estimate    SE   df t.ratio p.value
##  PNS - Control   -15.31 10.60 39.1  -1.444  0.1567
##
## Sex = M, Timepoint = d35:
##  contrast      estimate    SE   df t.ratio p.value
##  PNS - Control   -23.50 11.00 40.7  -2.141  0.0383
##
## Degrees-of-freedom method: kenward-roger
```

```
#Is there a difference in males or females within treatment group at each time point?
contrast(Foxp3_emcatcat_sex, "revpairwise", by=c("Treatment", "Timepoint"), adjust="BH")
```

```
## Treatment = Control, Timepoint = d7:
##  contrast estimate    SE   df t.ratio p.value
##  M - F     -7.0232 13.00 40.0  -0.541  0.5915
##
## Treatment = PNS, Timepoint = d7:
##  contrast estimate    SE   df t.ratio p.value
##  M - F      4.5329  7.61 40.5   0.595  0.5549
##
## Treatment = Control, Timepoint = d21:
##  contrast estimate    SE   df t.ratio p.value
##  M - F     22.1793  9.63 44.6   2.302  0.0261
##
## Treatment = PNS, Timepoint = d21:
##  contrast estimate    SE   df t.ratio p.value
##  M - F     -7.7424  6.66 40.1  -1.162  0.2522
##
## Treatment = Control, Timepoint = d35:
##  contrast estimate    SE   df t.ratio p.value
##  M - F      0.0658 10.20 41.4   0.006  0.9949
##
## Treatment = PNS, Timepoint = d35:
##  contrast estimate    SE   df t.ratio p.value
##  M - F     -8.1251  9.24 38.1  -0.880  0.3846
##
## Degrees-of-freedom method: kenward-roger
```

Colon Tissue *Il18* Expression Sex Differences

```
# Does colon IL18 expression differ by sex
#Include random intercept for litter
IL18_sex_effects <- lmer(IL18 ~ Timepoint * Treatment * Sex + (1 | Litter), data = colon_dat)
IL18_anova_table_for_sex <- anova(IL18_sex_effects, ddf = "Kenward-Roger")
print(IL18_anova_table_for_sex)
```

```
## Type III Analysis of Variance Table with Kenward-Roger's method
##                        Sum Sq Mean Sq NumDF  DenDF F value   Pr(>F)
## Timepoint              53.077 26.5384     2 40.479  5.8223 0.005993 **
## Treatment               0.514  0.5139     1 10.023  0.1127 0.743972
## Sex                     0.024  0.0235     1 41.380  0.0052 0.943093
## Timepoint:Treatment     2.155  1.0774     2 40.479  0.2364 0.790565
## Timepoint:Sex           1.759  0.8793     2 43.068  0.1928 0.825314
## Treatment:Sex           7.568  7.5677     1 41.380  1.6603 0.204723
## Timepoint:Treatment:Sex  1.650  0.8250     2 43.068  0.1809 0.835116
## ---
## Signif. codes:  0 '***' 0.001 '**' 0.01 '*' 0.05 '.' 0.1 ' ' 1
```

```
IL18_emcatcat_sex <- emmeans(IL18_sex_effects, ~ Timepoint * Treatment * Sex)
#Is there a difference in males or females between treatment group at each time point?
contrast(IL18_emcatcat_sex, "revpairwise", by=c("Sex", "Timepoint"), adjust="BH")
```

```
## Sex = F, Timepoint = d7:
##  contrast      estimate   SE   df t.ratio p.value
##  PNS - Control    0.700 1.81 29.6   0.386  0.7025
##
## Sex = M, Timepoint = d7:
##  contrast      estimate   SE   df t.ratio p.value
##  PNS - Control   -0.547 2.66 46.0  -0.205  0.8381
##
## Sex = F, Timepoint = d21:
##  contrast      estimate   SE   df t.ratio p.value
##  PNS - Control    1.300 1.73 25.1   0.750  0.4603
##
## Sex = M, Timepoint = d21:
##  contrast      estimate   SE   df t.ratio p.value
##  PNS - Control   -1.751 1.81 26.8  -0.968  0.3417
##
## Sex = F, Timepoint = d35:
##  contrast      estimate   SE   df t.ratio p.value
##  PNS - Control   -0.479 2.10 38.8  -0.228  0.8210
##
## Sex = M, Timepoint = d35:
##  contrast      estimate   SE   df t.ratio p.value
##  PNS - Control   -1.778 2.13 39.4  -0.834  0.4091
##
## Degrees-of-freedom method: kenward-roger
```

```
#Is there a difference in males or females within treatment group at each time point?
contrast(IL18_emcatcat_sex, "revpairwise", by=c("Treatment", "Timepoint"), adjust="BH")
```

```
## Treatment = Control, Timepoint = d7:
##  contrast estimate   SE   df t.ratio p.value
##  M - F      0.5731 2.49 41.4   0.230  0.8190
##
## Treatment = PNS, Timepoint = d7:
##  contrast estimate   SE   df t.ratio p.value
##  M - F     -0.6734 1.48 42.1  -0.454  0.6525
##
## Treatment = Control, Timepoint = d21:
##  contrast estimate   SE   df t.ratio p.value
##  M - F      1.1165 1.75 46.9   0.639  0.5260
##
## Treatment = PNS, Timepoint = d21:
##  contrast estimate   SE   df t.ratio p.value
##  M - F     -1.9346 1.33 41.9  -1.454  0.1535
##
## Treatment = Control, Timepoint = d35:
##  contrast estimate   SE   df t.ratio p.value
##  M - F      1.2648 1.85 40.4   0.684  0.4979
##
## Treatment = PNS, Timepoint = d35:
##  contrast estimate   SE   df t.ratio p.value
##  M - F     -0.0345 1.79 40.0  -0.019  0.9848
##
## Degrees-of-freedom method: kenward-roger
```

Colon Tissue *Il4* Expression Sex Differences

```
# Does colon IL4 expression differ by sex
#Include random intercept for litter
IL4_sex_effects <- lmer(IL4 ~ Timepoint * Treatment * Sex + (1 | Litter), data = colon_dat)
IL4_anova_table_for_sex <- anova(IL4_sex_effects, ddf = "Kenward-Roger")
print(IL4_anova_table_for_sex)
```

```
## Type III Analysis of Variance Table with Kenward-Roger's method
##                         Sum Sq Mean Sq NumDF  DenDF F value  Pr(>F)
## Timepoint               563.58 281.788     2 41.905  3.3564 0.04446 *
## Treatment               113.51 113.507     1  9.863  1.3523 0.27224
## Sex                      15.55  15.554     1 41.829  0.1853 0.66906
## Timepoint:Treatment     343.64 171.820     2 41.905  2.0465 0.14190
## Timepoint:Sex            15.04   7.519     2 39.585  0.0893 0.91474
## Treatment:Sex            28.60  28.599     1 41.829  0.3407 0.56255
## Timepoint:Treatment:Sex  96.06  48.028     2 39.585  0.5705 0.56983
## ---
## Signif. codes:  0 '***' 0.001 '**' 0.01 '*' 0.05 '.' 0.1 ' ' 1
```

```
IL4_emcatcat_sex <- emmeans(IL4_sex_effects, ~ Timepoint * Treatment * Sex)
#Is there a difference in males or females between treatment group at each time point?
contrast(IL4_emcatcat_sex, "revpairwise", by=c("Sex", "Timepoint"), adjust="BH")
```

```
## Sex = F, Timepoint = d7:
##  contrast      estimate    SE   df t.ratio p.value
##  PNS - Control   -2.758  6.19 36.3  -0.446  0.6584
##
## Sex = M, Timepoint = d7:
##  contrast      estimate    SE   df t.ratio p.value
##  PNS - Control    0.172 10.30 42.5   0.017  0.9868
##
## Sex = F, Timepoint = d21:
##  contrast      estimate    SE   df t.ratio p.value
##  PNS - Control    2.601  6.95 32.0   0.374  0.7105
##
## Sex = M, Timepoint = d21:
##  contrast      estimate    SE   df t.ratio p.value
##  PNS - Control    1.912  5.95 31.0   0.322  0.7499
##
## Sex = F, Timepoint = d35:
##  contrast      estimate    SE   df t.ratio p.value
##  PNS - Control   -5.476  7.83 41.7  -0.699  0.4884
##
## Sex = M, Timepoint = d35:
##  contrast      estimate    SE   df t.ratio p.value
##  PNS - Control  -18.507  7.79 41.5  -2.374  0.0223
##
## Degrees-of-freedom method: kenward-roger
```

```
#Is there a difference in males or females between within group at each time point?
contrast(IL4_emcatcat_sex, "revpairwise", by=c("Treatment", "Timepoint"), adjust="BH")
```

```
## Treatment = Control, Timepoint = d7:
##  contrast estimate    SE   df t.ratio p.value
##  M - F     -3.8556 10.50 42.8  -0.369  0.7141
##
## Treatment = PNS, Timepoint = d7:
##  contrast estimate    SE   df t.ratio p.value
##  M - F     -0.9249  6.13 42.1  -0.151  0.8807
##
## Treatment = Control, Timepoint = d21:
##  contrast estimate    SE   df t.ratio p.value
##  M - F      0.7839  6.47 28.8   0.121  0.9044
##
## Treatment = PNS, Timepoint = d21:
##  contrast estimate    SE   df t.ratio p.value
##  M - F      0.0948  6.72 41.0   0.014  0.9888
##
## Treatment = Control, Timepoint = d35:
##  contrast estimate    SE   df t.ratio p.value
##  M - F      4.4879  7.85 42.2   0.572  0.5703
##
## Treatment = PNS, Timepoint = d35:
##  contrast estimate    SE   df t.ratio p.value
##  M - F     -8.5424  7.70 40.1  -1.109  0.2740
##
## Degrees-of-freedom method: kenward-roger
```

Colon Tissue *Il5* Expression Sex Differences

```
# Does colon IL5 expression differ by sex
#Include random intercept for litter
IL5_sex_effects <- lmer(IL5 ~ Timepoint * Treatment * Sex + (1 | Litter), data = colon_dat)
IL5_anova_table_for_sex <- anova(IL5_sex_effects, ddf = "Kenward-Roger")
print(IL5_anova_table_for_sex)
```

```
## Type III Analysis of Variance Table with Kenward-Roger's method
##                           Sum Sq  Mean Sq NumDF  DenDF F value  Pr(>F)
## Timepoint               0.211124 0.105562     2 37.147  4.8127 0.01385 *
## Treatment               0.008229 0.008229     1  9.443  0.3752 0.55466
## Sex                     0.007480 0.007480     1 37.859  0.3410 0.56270
## Timepoint:Treatment     0.020950 0.010475     2 37.147  0.4776 0.62405
## Timepoint:Sex           0.018589 0.009295     2 38.942  0.4237 0.65760
## Treatment:Sex           0.046749 0.046749     1 37.859  2.1313 0.15256
## Timepoint:Treatment:Sex 0.046124 0.023062     2 38.942  1.0513 0.35919
## ---
## Signif. codes:  0 '***' 0.001 '**' 0.01 '*' 0.05 '.' 0.1 ' ' 1
```

```
IL5_emcatcat_sex <- emmeans(IL5_sex_effects, ~ Timepoint * Treatment * Sex)
#Is there a difference in males or females between treatment group at each time point?
contrast(IL5_emcatcat_sex, "revpairwise", by=c("Sex", "Timepoint"), adjust="BH")
```

```
## Sex = F, Timepoint = d7:
##  contrast      estimate    SE   df t.ratio p.value
##  PNS - Control -0.22976 0.156 19.4  -1.475  0.1563
##
## Sex = M, Timepoint = d7:
##  contrast      estimate    SE   df t.ratio p.value
##  PNS - Control -0.04990 0.209 37.8  -0.239  0.8123
##
## Sex = F, Timepoint = d21:
##  contrast      estimate    SE   df t.ratio p.value
##  PNS - Control -0.23363 0.152 17.9  -1.533  0.1428
##
## Sex = M, Timepoint = d21:
##  contrast      estimate    SE   df t.ratio p.value
##  PNS - Control  0.07037 0.155 18.4   0.454  0.6554
##
## Sex = F, Timepoint = d35:
##  contrast      estimate    SE   df t.ratio p.value
##  PNS - Control  0.00585 0.175 27.0   0.033  0.9736
##
## Sex = M, Timepoint = d35:
##  contrast      estimate    SE   df t.ratio p.value
##  PNS - Control -0.02892 0.175 26.8  -0.166  0.8697
##
## Degrees-of-freedom method: kenward-roger
```

```
#Is there a difference in males or females within treatment group at each time point?
contrast(IL5_emcatcat_sex, "revpairwise", by=c("Treatment", "Timepoint"), adjust="BH")
```

```
## Treatment = Control, Timepoint = d7:
##  contrast  estimate     SE   df t.ratio p.value
##  M - F    -0.180653 0.1740 37.6  -1.040  0.3050
##
## Treatment = PNS, Timepoint = d7:
##  contrast  estimate     SE   df t.ratio p.value
##  M - F    -0.000787 0.1110 39.0  -0.007  0.9944
##
## Treatment = Control, Timepoint = d21:
##  contrast  estimate     SE   df t.ratio p.value
##  M - F    -0.119237 0.1270 42.9  -0.939  0.3530
##
## Treatment = PNS, Timepoint = d21:
##  contrast  estimate     SE   df t.ratio p.value
##  M - F     0.184764 0.0919 38.4   2.010  0.0515
##
## Treatment = Control, Timepoint = d35:
##  contrast  estimate     SE   df t.ratio p.value
##  M - F    -0.014480 0.1290 37.1  -0.112  0.9111
##
## Treatment = PNS, Timepoint = d35:
##  contrast  estimate     SE   df t.ratio p.value
##  M - F    -0.049251 0.1310 37.4  -0.377  0.7084
##
## Degrees-of-freedom method: kenward-roger
```

Colon Tissue *Il13* Expression Sex Differences

```
# Does colon IL13 expression differ by sex
#Include random intercept for litter
IL13_sex_effects <- lmer(IL13 ~ Timepoint * Treatment * Sex + (1 | Litter), data = colon_dat)
```

```
## boundary (singular) fit: see help('isSingular')
```

```
IL13_anova_table_for_sex <- anova(IL13_sex_effects, ddf = "Kenward-Roger")
print(IL13_anova_table_for_sex)
```

```
## Type III Analysis of Variance Table with Kenward-Roger's method
##                          Sum Sq Mean Sq NumDF  DenDF F value    Pr(>F)
## Timepoint               1.42203 0.71102     2 42.821  9.4107 0.0004096 ***
## Treatment               0.02639 0.02639     1  9.949  0.3493 0.5677041
## Sex                     0.04367 0.04367     1 44.766  0.5780 0.4511000
## Timepoint:Treatment     0.01114 0.00557     2 42.821  0.0737 0.9290467
## Timepoint:Sex           0.08760 0.04380     2 42.793  0.5788 0.5649089
## Treatment:Sex           0.16096 0.16096     1 44.766  2.1304 0.1513879
## Timepoint:Treatment:Sex 0.07421 0.03710     2 42.793  0.4903 0.6158418
## ---
## Signif. codes:  0 '***' 0.001 '**' 0.01 '*' 0.05 '.' 0.1 ' ' 1
```

```
IL13_emcatcat_sex <- emmeans(IL13_sex_effects, ~ Timepoint * Treatment * Sex)
#Is there a difference in males or females between treatment group at each time point?
contrast(IL13_emcatcat_sex, "revpairwise", by=c("Sex", "Timepoint"), adjust="BH")
```

```
## Sex = F, Timepoint = d7:
##  contrast      estimate    SE   df t.ratio p.value
##  PNS - Control  -0.1997 0.204 37.6  -0.977  0.3349
##
## Sex = M, Timepoint = d7:
##  contrast      estimate    SE   df t.ratio p.value
##  PNS - Control   0.2910 0.307 44.4   0.949  0.3476
##
## Sex = F, Timepoint = d21:
##  contrast      estimate    SE   df t.ratio p.value
##  PNS - Control  -0.0552 0.171 37.5  -0.322  0.7494
##
## Sex = M, Timepoint = d21:
##  contrast      estimate    SE   df t.ratio p.value
##  PNS - Control   0.2444 0.175 33.7   1.398  0.1714
##
## Sex = F, Timepoint = d35:
##  contrast      estimate    SE   df t.ratio p.value
##  PNS - Control   0.0135 0.228 43.5   0.059  0.9530
##
## Sex = M, Timepoint = d35:
##  contrast      estimate    SE   df t.ratio p.value
##  PNS - Control   0.0273 0.232 43.7   0.118  0.9068
##
## Degrees-of-freedom method: kenward-roger
```

```
#Is there a difference in males or females within treatment group at each time point?
contrast(IL13_emcatcat_sex, "revpairwise", by=c("Treatment", "Timepoint"), adjust="BH")
```

```
## Treatment = Control, Timepoint = d7:
##  contrast estimate    SE   df t.ratio p.value
##  M - F     -0.4597 0.311 45.0  -1.478  0.1464
##
## Treatment = PNS, Timepoint = d7:
##  contrast estimate    SE   df t.ratio p.value
##  M - F      0.0310 0.206 40.8   0.150  0.8812
##
## Treatment = Control, Timepoint = d21:
##  contrast estimate    SE   df t.ratio p.value
##  M - F     -0.1189 0.191 32.3  -0.624  0.5372
##
## Treatment = PNS, Timepoint = d21:
##  contrast estimate    SE   df t.ratio p.value
##  M - F      0.1806 0.164 44.9   1.102  0.2764
##
## Treatment = Control, Timepoint = d35:
##  contrast estimate    SE   df t.ratio p.value
##  M - F     -0.0328 0.234 44.5  -0.140  0.8890
##
## Treatment = PNS, Timepoint = d35:
##  contrast estimate    SE   df t.ratio p.value
##  M - F     -0.0190 0.228 43.6  -0.083  0.9340
##
## Degrees-of-freedom method: kenward-roger
```

Colon Tissue *Il17a* Expression Sex Differences

```
# Does colon IL17 expression differ by sex
#Include random intercept for litter
IL17_sex_effects <- lmer(IL17 ~ Timepoint * Treatment * Sex + (1 | Litter), data = colon_dat)
IL17_anova_table_for_sex <- anova(IL17_sex_effects, ddf = "Kenward-Roger")
print(IL17_anova_table_for_sex)
```

```
## Type III Analysis of Variance Table with Kenward-Roger's method
##                          Sum Sq  Mean Sq NumDF  DenDF F value Pr(>F)
## Timepoint               0.18499 0.092497     2 37.216  0.5313 0.5922
## Treatment               0.25608 0.256080     1  9.821  1.4710 0.2536
## Sex                     0.07774 0.077744     1 37.929  0.4466 0.5080
## Timepoint:Treatment     0.18048 0.090242     2 37.216  0.5184 0.5997
## Timepoint:Sex           0.51429 0.257145     2 39.373  1.4768 0.2407
## Treatment:Sex           0.28152 0.281523     1 37.929  1.6171 0.2112
## Timepoint:Treatment:Sex 0.24928 0.124640     2 39.373  0.7158 0.4950
```

```
IL17_emcatcat_sex <- emmeans(IL17_sex_effects, ~ Timepoint * Treatment * Sex)
#Is there a difference in males or females between treatment group at each time point?
contrast(IL17_emcatcat_sex, "revpairwise", by=c("Sex", "Timepoint"), adjust="BH")
```

```
## Sex = F, Timepoint = d7:
##  contrast      estimate    SE   df t.ratio p.value
##  PNS - Control  -0.5533 0.394 28.7  -1.405  0.1707
##
## Sex = M, Timepoint = d7:
##  contrast      estimate    SE   df t.ratio p.value
##  PNS - Control  -0.4518 0.535 42.4  -0.844  0.4034
##
## Sex = F, Timepoint = d21:
##  contrast      estimate    SE   df t.ratio p.value
##  PNS - Control   0.0339 0.379 24.5   0.090  0.9293
##
## Sex = M, Timepoint = d21:
##  contrast      estimate    SE   df t.ratio p.value
##  PNS - Control  -0.7909 0.395 26.6  -2.003  0.0555
##
## Sex = F, Timepoint = d35:
##  contrast      estimate    SE   df t.ratio p.value
##  PNS - Control   0.0729 0.431 33.9   0.169  0.8669
##
## Sex = M, Timepoint = d35:
##  contrast      estimate    SE   df t.ratio p.value
##  PNS - Control  -0.3330 0.446 35.6  -0.746  0.4605
##
## Degrees-of-freedom method: kenward-roger
```

```
#Is there a difference in males or females within treatment group at each time point?
contrast(IL17_emcatcat_sex, "revpairwise", by=c("Treatment", "Timepoint"), adjust="BH")
```

```
## Treatment = Control, Timepoint = d7:
##  contrast estimate    SE   df t.ratio p.value
##  M - F     -0.1682 0.488 37.7  -0.344  0.7324
##
## Treatment = PNS, Timepoint = d7:
##  contrast estimate    SE   df t.ratio p.value
##  M - F     -0.0668 0.311 37.5  -0.215  0.8312
##
## Treatment = Control, Timepoint = d21:
##  contrast estimate    SE   df t.ratio p.value
##  M - F      0.8650 0.374 43.7   2.311  0.0256
##
## Treatment = PNS, Timepoint = d21:
##  contrast estimate    SE   df t.ratio p.value
##  M - F      0.0402 0.276 38.0   0.146  0.8851
##
## Treatment = Control, Timepoint = d35:
##  contrast estimate    SE   df t.ratio p.value
##  M - F      0.1645 0.387 38.4   0.426  0.6728
##
## Treatment = PNS, Timepoint = d35:
##  contrast estimate    SE   df t.ratio p.value
##  M - F     -0.2413 0.352 36.6  -0.686  0.4970
##
## Degrees-of-freedom method: kenward-roger
```

PNS and Offspring Immune Dysregulation: Alpha Diversity

```
library(tidyverse)
library(lme4)
library(lmerTest)
library(emmeans)
```

Additional Alpha Diversity Statistical Analysis–Controlling for Litter

Read in Ileum Alpha Diversity Data

```
#read in ileum alpha diversity
ic_alpha <- read_csv("IC_alphadiversity.csv")
```

```
## Rows: 57 Columns: 8
## ── Column specification ────────────────────────────────────────────────────────
## Delimiter: ","
## chr (6): id, Version, Timepoint, Treatment, Litter, Sex
## dbl (2): Shannon, Faith
##
## ℹ Use `spec()` to retrieve the full column specification for this data.
## ℹ Specify the column types or set `show_col_types = FALSE` to quiet this message.
```

```
# prep data
ic_alpha$Timepoint <- factor(ic_alpha$Timepoint, levels = c("d7", "d21", "d35"))
ic_alpha$Treatment <- factor(ic_alpha$Treatment, levels = c("Control", "PNS"))
ic_alpha$Litter <- factor(ic_alpha$Litter, levels = c("F5", "O4", "R4", "R5", "K6", "B4", "F4", "L4", "K5", "P5", "M6"))
```

Ileum Shannon Alpha Diversity

```
# Fit linear mixed-effects model
# Include random intercept for litter
ic_shannon_model <- lmer(Shannon ~ Timepoint * Treatment + (1 | Litter), data = ic_alpha)
summary(ic_shannon_model)
```

```
## Linear mixed model fit by REML. t-tests use Satterthwaite's method [
## lmerModLmerTest]
## Formula: Shannon ~ Timepoint * Treatment + (1 | Litter)
##    Data: ic_alpha
##
## REML criterion at convergence: 123.5
##
## Scaled residuals:
##     Min      1Q  Median      3Q     Max
## -1.7529 -0.5467 -0.1394  0.4444  2.3655
##
## Random effects:
##  Groups   Name        Variance Std.Dev.
##  Litter   (Intercept) 0.07147  0.2673
##  Residual             0.45993  0.6782
## Number of obs: 57, groups:  Litter, 11
##
## Fixed effects:
##                            Estimate Std. Error      df t value Pr(>|t|)
## (Intercept)                  1.5157     0.2884 33.8100   5.256 8.12e-06 ***
## Timepointd21                 2.3902     0.3427 48.2907   6.975 7.80e-09 ***
## Timepointd35                 1.7937     0.3625 42.7931   4.948 1.21e-05 ***
## TreatmentPNS                -0.4006     0.3703 31.9708  -1.082 0.287439
## Timepointd21:TreatmentPNS   0.1593     0.4387 46.5527   0.363 0.718131
## Timepointd35:TreatmentPNS   1.8067     0.4873 45.7292   3.707 0.000564 ***
## ---
## Signif. codes:  0 '***' 0.001 '**' 0.01 '*' 0.05 '.' 0.1 ' ' 1
##
## Correlation of Fixed Effects:
##             (Intr) Tmpn21 Tmpn35 TrtPNS T21:TP
## Timepontd21 -0.705
## Timepontd35 -0.628  0.529
## TreatmntPNS -0.779  0.549  0.489
## Tmpn21:TPNS  0.551 -0.781 -0.413 -0.687
## Tmpn35:TPNS  0.468 -0.393 -0.744 -0.602  0.506
```

```
# Create a type III ANOVA table
ic_shannon_anova_table <- anova(ic_shannon_model, ddf = "Kenward-Roger")
print(ic_shannon_anova_table)
```

```
## Type III Analysis of Variance Table with Kenward-Roger's method
##                     Sum Sq Mean Sq NumDF  DenDF F value    Pr(>F)
## Timepoint           75.050  37.525     2 46.564 81.5823 6.064e-16 ***
## Treatment            0.472   0.472     1  8.776  1.0267 0.3380477
## Timepoint:Treatment  7.529   3.764     2 46.564  8.1838 0.0009001 ***
## ---
## Signif. codes:  0 '***' 0.001 '**' 0.01 '*' 0.05 '.' 0.1 ' ' 1
```

Ileum FaithPD Alpha Diversity

```
# Fit linear mixed-effects model
# Include random intercept for litter
ic_faith_model <- lmer(Faith ~ Timepoint * Treatment + (1 | Litter), data = ic_alpha)
summary(ic_faith_model)
```

```
## Linear mixed model fit by REML. t-tests use Satterthwaite's method [
## lmerModLmerTest]
## Formula: Faith ~ Timepoint * Treatment + (1 | Litter)
##    Data: ic_alpha
##
## REML criterion at convergence: 366
##
## Scaled residuals:
##      Min       1Q   Median       3Q      Max
## -1.78639 -0.63480 -0.03189  0.52597  2.15510
##
## Random effects:
##  Groups   Name        Variance Std.Dev.
##  Litter   (Intercept) 17.60    4.195
##  Residual             49.43    7.031
## Number of obs: 57, groups:  Litter, 11
##
## Fixed effects:
##                             Estimate Std. Error      df t value Pr(>|t|)
## (Intercept)                   4.7802     3.3369 28.3222   1.433    0.163
## Timepointd21                 26.7357     3.6099 47.4708   7.406 1.87e-09 ***
## Timepointd35                 18.9236     3.7581 43.0308   5.035 9.00e-06 ***
## TreatmentPNS                 -0.1374     4.3132 26.0158  -0.032    0.975
## Timepointd21:TreatmentPNS   -0.7106     4.5950 46.0116  -0.155    0.878
## Timepointd35:TreatmentPNS   25.8244     5.0882 44.9767   5.075 7.16e-06 ***
## ---
## Signif. codes:  0 '***' 0.001 '**' 0.01 '*' 0.05 '.' 0.1 ' ' 1
##
## Correlation of Fixed Effects:
##             (Intr) Tmpn21 Tmpn35 TrtPNS T21:TP
## Timepontd21 -0.644
## Timepontd35 -0.563  0.521
## TreatmntPNS -0.774  0.498  0.436
## Tmpn21:TPNS  0.506 -0.786 -0.409 -0.620
## Tmpn35:TPNS  0.416 -0.384 -0.739 -0.534  0.499
```

```
# Create a type III ANOVA table
ic_faith_anova_table <- anova(ic_faith_model, ddf = "Kenward-Roger")
print(ic_faith_anova_table)
```

```
## Type III Analysis of Variance Table with Kenward-Roger's method
##                     Sum Sq Mean Sq NumDF  DenDF F value    Pr(>F)
## Timepoint           9439.7  4719.8     2 45.456 95.4743 < 2.2e-16 ***
## Treatment            319.2   319.2     1  8.974  6.4563   0.03173 *
## Timepoint:Treatment 1711.7   855.9     2 45.456 17.3127 2.572e-06 ***
## ---
## Signif. codes:  0 '***' 0.001 '**' 0.01 '*' 0.05 '.' 0.1 ' ' 1
```

Read in Colon Alpha Diversity Data

```
#read in colon alpha diversity
cc_alpha <- read_csv("CC_alphadiversity.csv")
```

```
## Rows: 52 Columns: 8
## ── Column specification ────────────────────────────────────────────────────────
## Delimiter: ","
## chr (6): id, Version, Timepoint, Treatment, Litter, Sex
## dbl (2): Shannon, Faith
##
## ℹ Use `spec()` to retrieve the full column specification for this data.
## ℹ Specify the column types or set `show_col_types = FALSE` to quiet this message.
```

```
# prep data
cc_alpha$Timepoint <- factor(cc_alpha$Timepoint, levels = c("d7", "d21", "d35"))
cc_alpha$Treatment <- factor(cc_alpha$Treatment, levels = c("Control", "PNS"))
cc_alpha$Litter <- factor(cc_alpha$Litter, levels = c("F5", "O4", "R4", "R5", "K6", "B4", "F4", "L4", "K5", "P5", "M6"))
```

Colon Shannon Alpha Diversity

```
# Fit linear mixed-effects model
# Include random intercept for litter
cc_shannon_model <- lmer(Shannon ~ Timepoint * Treatment + (1 | Litter), data = cc_alpha)
summary(cc_shannon_model)
```

```
## Linear mixed model fit by REML. t-tests use Satterthwaite's method [
## lmerModLmerTest]
## Formula: Shannon ~ Timepoint * Treatment + (1 | Litter)
##    Data: cc_alpha
##
## REML criterion at convergence: 78.3
##
## Scaled residuals:
##     Min      1Q  Median      3Q     Max
## -1.8873 -0.5557 -0.1195  0.6369  1.7273
##
## Random effects:
##  Groups   Name        Variance Std.Dev.
##  Litter   (Intercept) 0.1051   0.3242
##  Residual             0.1926   0.4389
## Number of obs: 52, groups:  Litter, 11
##
## Fixed effects:
##                            Estimate Std. Error      df t value Pr(>|t|)
## (Intercept)                  1.4254     0.2367 24.3459   6.023 3.04e-06 ***
## Timepointd21                 4.0075     0.2384 41.0415  16.813  < 2e-16 ***
## Timepointd35                 3.1085     0.2454 37.5034  12.666 4.06e-15 ***
## TreatmentPNS                -0.3521     0.3244 24.4524  -1.085   0.2884
## Timepointd21:TreatmentPNS   0.6228     0.3207 41.2240   1.942   0.0590 .
## Timepointd35:TreatmentPNS  -0.6108     0.3350 37.4557  -1.823   0.0763 .
## ---
## Signif. codes:  0 '***' 0.001 '**' 0.01 '*' 0.05 '.' 0.1 ' ' 1
##
## Correlation of Fixed Effects:
##             (Intr) Tmpn21 Tmpn35 TrtPNS T21:TP
## Timepontd21 -0.636
## Timepontd35 -0.560  0.549
## TreatmntPNS -0.730  0.464  0.408
## Tmpn21:TPNS  0.472 -0.743 -0.408 -0.651
## Tmpn35:TPNS  0.410 -0.402 -0.733 -0.556  0.558
```

```
# Create a type III ANOVA table
cc_shannon_anova_table <- anova(cc_shannon_model, ddf = "Kenward-Roger")
print(cc_shannon_anova_table)
```

```
## Type III Analysis of Variance Table with Kenward-Roger's method
##                      Sum Sq Mean Sq NumDF  DenDF  F value    Pr(>F)
## Timepoint           137.238  68.619     2 40.122 356.1248 < 2.2e-16 ***
## Treatment             0.405   0.405     1  8.954   2.1005  0.181355
## Timepoint:Treatment   2.991   1.496     2 40.122   7.7626  0.001413 **
## ---
## Signif. codes:  0 '***' 0.001 '**' 0.01 '*' 0.05 '.' 0.1 ' ' 1
```

Colon FaithPD Alpha Diversity

```
# Fit linear mixed-effects model
# Include random intercept for litter
cc_faith_model <- lmer(Faith ~ Timepoint * Treatment + (1 | Litter), data = cc_alpha)
summary(cc_faith_model)
```

```
## Linear mixed model fit by REML. t-tests use Satterthwaite's method [
## lmerModLmerTest]
## Formula: Faith ~ Timepoint * Treatment + (1 | Litter)
##    Data: cc_alpha
##
## REML criterion at convergence: 304.4
##
## Scaled residuals:
##     Min      1Q  Median      3Q     Max
## -2.3461 -0.5614 -0.0861  0.5754  2.2440
##
## Random effects:
##  Groups   Name        Variance Std.Dev.
##  Litter   (Intercept)  1.194   1.093
##  Residual             32.231   5.677
## Number of obs: 52, groups:  Litter, 11
##
## Fixed effects:
##                            Estimate Std. Error       df t value Pr(>|t|)
## (Intercept)                  4.6651     2.3859  37.2684   1.955 0.058086 .
## Timepointd21                36.4721     2.9557  43.8500  12.340 7.37e-16 ***
## Timepointd35                39.8111     3.1616  38.2909  12.592 3.46e-15 ***
## TreatmentPNS                 0.1413     3.2562  35.9649   0.043 0.965631
## Timepointd21:TreatmentPNS   9.8265     3.9704  43.9937   2.475 0.017251 *
## Timepointd35:TreatmentPNS -16.9311     4.3179  38.2374  -3.921 0.000354 ***
## ---
## Signif. codes:  0 '***' 0.001 '**' 0.01 '*' 0.05 '.' 0.1 ' ' 1
##
## Correlation of Fixed Effects:
##             (Intr) Tmpn21 Tmpn35 TrtPNS T21:TP
## Timepontd21 -0.776
## Timepontd35 -0.714  0.575
## TreatmntPNS -0.733  0.569  0.523
## Tmpn21:TPNS  0.578 -0.744 -0.428 -0.788
## Tmpn35:TPNS  0.523 -0.421 -0.732 -0.712  0.583
```

```
# Create a type III ANOVA table
cc_faith_anova_table <- anova(cc_faith_model, ddf = "Kenward-Roger")
print(cc_faith_anova_table)
```

```
## Type III Analysis of Variance Table with Kenward-Roger's method
##                      Sum Sq Mean Sq NumDF  DenDF  F value    Pr(>F)
## Timepoint           13870.3  6935.1     2 42.416 215.0269 < 2.2e-16 ***
## Treatment              48.1    48.1     1  7.914   1.4917    0.2571
## Timepoint:Treatment  1545.9   772.9     2 42.416  23.9656 1.085e-07 ***
## ---
## Signif. codes:  0 '***' 0.001 '**' 0.01 '*' 0.05 '.' 0.1 ' ' 1
```

PNS and Offspring Immune Dysregulation: Beta Diversity

```
library(qiime2R)
library(permute)
library(vegan)
```

Additional Beta Diversity Statistical Analysis–Controlling for Litter

Ileum Beta Diversity QIIME2 Unweighted UniFrac Distances

```
#Read in ileum Unweighted UniFrac QIIME2 distance matrix artifact file
ileum_uwu_dm <- read_qza("ileum_unweighted_unifrac_dist_matrix.qza")

#Convert from list to distance object
ileum_uwu_dmo <- as.dist(ileum_uwu_dm$data)

#Read in associated metadata
ileum_meta <- read_q2metadata("meta_ileum_rarefied.tsv")

#Define permuation design to account for litter
ileum_perm <- how(blocks = ileum_meta$Litter, nperm = 999)
#Run Adonis2 while controlling for permutations at the level of treatment assignment (litter)
ileum_unweighted_unifrac_adonis2 <- adonis2(ileum_uwu_dmo ~ Treatment * Timepoint, data = ileum_meta, by = "terms", permutat
ions = ileum_perm)
print(ileum_unweighted_unifrac_adonis2)
```

```
## Permutation test for adonis under reduced model
## Terms added sequentially (first to last)
## Blocks:  ileum_meta$Litter
## Permutation: free
## Number of permutations: 999
##
## adonis2(formula = ileum_uwu_dmo ~ Treatment * Timepoint, data = ileum_meta, permutations = ileum_perm, by = "terms")
##                     Df SumOfSqs      R2      F Pr(>F)
## Treatment            1   0.3413 0.04599 2.0247  0.001 ***
## Timepoint            1   0.5692 0.07670 3.3768  0.006 **
## Treatment:Timepoint  1   0.6109 0.08232 3.6244  0.001 ***
## Residual            35   5.8998 0.79499
## Total               38   7.4213 1.00000
## ---
## Signif. codes:  0 '***' 0.001 '**' 0.01 '*' 0.05 '.' 0.1 ' ' 1
```

Colon Beta Diversity QIIME2 Unweighted UniFrac Distances

```
#Read in colon Unweighted UniFrac QIIME2 distance matrix artifact file
colon_uwu_dm <- read_qza("colon_unweighted_unifrac_dist_matrix.qza")

#Convert from list to distance object
colon_uwu_dmo <- as.dist(colon_uwu_dm$data)

#Read in associated metadata
colon_meta <- read_q2metadata("meta_colon_rarefied.tsv")

#Define permuation design to account for litter
colon_perm <- how(blocks = colon_meta$Litter, nperm = 999)
#Run Adonis2 while controlling for permutations at the level of treatment assignment (litter)
colon_unweighted_unifrac_adonis2 <- adonis2(colon_uwu_dmo ~ Treatment * Timepoint, data = colon_meta, by = "terms", permutat
ions = colon_perm)
print(colon_unweighted_unifrac_adonis2)
```

```
## Permutation test for adonis under reduced model
## Terms added sequentially (first to last)
## Blocks:  colon_meta$Litter
## Permutation: free
## Number of permutations: 999
##
## adonis2(formula = colon_uwu_dmo ~ Treatment * Timepoint, data = colon_meta, permutations = colon_perm, by = "terms")
##                     Df SumOfSqs      R2      F Pr(>F)
## Treatment            1   1.4602 0.11404 7.0675  0.046 *
## Timepoint            2   1.1481 0.08966 2.7784  0.031 *
## Treatment:Timepoint  2   0.6924 0.05407 1.6756  0.147
## Residual            46   9.5041 0.74223
## Total               51  12.8048 1.00000
## ---
## Signif. codes:  0 '***' 0.001 '**' 0.01 '*' 0.05 '.' 0.1 ' ' 1
```

Supplementary File 5. C57BL/6 WT vs MyD88 KO Offspring Gene Expression Analysis

```
library(tidyverse)
library(lme4)
library(lmerTest)
library(emmeans)
```

MyD88 KO vs C57BL6 WT Lung Tissue Gene Expression–Figure 8a

Read in Lung Gene Expression Data

```
myd88_lung_dat <- read_csv("Myd88_d7_Lg_expression.csv")
```

```
## Rows: 36 Columns: 21
## ── Column specification ────────────────────────────────────────────────────────
## Delimiter: ","
## chr  (4): SampleID, Treatment, Strain, Litter
## dbl (17): Ifnb, Ifng, Isg15, Muc5ac, Muc5b, Il1b, Il4, Il5, Il6, Il10, Il13,...
##
## ℹ Use `spec()` to retrieve the full column specification for this data.
## ℹ Specify the column types or set `show_col_types = FALSE` to quiet this message.
```

```
summary(myd88_lung_dat) # To make sure that your data looks correct (i.e., R is interpreting it the way you want)
```

```
##    SampleID          Treatment            Strain             Litter
##  Length:36          Length:36          Length:36          Length:36
##  Class :character   Class :character   Class :character   Class :character
##  Mode  :character   Mode  :character   Mode  :character   Mode  :character
##
##
##
##
##       Ifnb             Ifng            Isg15            Muc5ac
##  Min.   : 0.450   Min.   : 0.790   Min.   :0.4400   Min.   :0.080
##  1st Qu.: 1.480   1st Qu.: 1.288   1st Qu.:0.6150   1st Qu.:0.350
##  Median : 2.790   Median : 1.790   Median :0.8200   Median :0.590
##  Mean   : 6.931   Mean   : 2.739   Mean   :0.9037   Mean   :0.856
##  3rd Qu.: 6.673   3rd Qu.: 2.685   3rd Qu.:1.1550   3rd Qu.:0.940
##  Max.   :37.000   Max.   :11.340   Max.   :1.8400   Max.   :4.210
##                                    NA's   :1        NA's   :1
##      Muc5b            Il1b             Il4              Il5
##  Min.   :0.050   Min.   : 0.690   Min.   :0.000   Min.   :0.500
##  1st Qu.:0.240   1st Qu.: 1.255   1st Qu.:0.255   1st Qu.:1.405
##  Median :0.480   Median : 1.550   Median :0.640   Median :2.560
##  Mean   :1.233   Mean   : 2.501   Mean   :0.822   Mean   :3.093
##  3rd Qu.:1.595   3rd Qu.: 2.435   3rd Qu.:1.395   3rd Qu.:4.455
##  Max.   :6.030   Max.   :12.490   Max.   :2.170   Max.   :8.430
##  NA's   :1       NA's   :1        NA's   :1       NA's   :1
##       Il6             Il10             Il13            Il17a
##  Min.   :0.830   Min.   :0.3100   Min.   : 0.560   Min.   :0.170
##  1st Qu.:1.045   1st Qu.:0.6225   1st Qu.: 1.200   1st Qu.:0.755
##  Median :1.330   Median :0.9050   Median : 2.340   Median :0.940
##  Mean   :1.949   Mean   :0.8685   Mean   : 5.031   Mean   :1.063
##  3rd Qu.:2.245   3rd Qu.:1.0650   3rd Qu.: 7.505   3rd Qu.:1.380
##  Max.   :5.590   Max.   :1.4300   Max.   :15.790   Max.   :2.280
##  NA's   :1       NA's   :2        NA's   :1        NA's   :1
##       Il18            Tgfb1             Tnf              Ahr
##  Min.   :0.3800   Min.   :0.8100   Min.   :0.560   Min.   :0.7900
##  1st Qu.:0.6500   1st Qu.:0.9875   1st Qu.:0.775   1st Qu.:0.9675
##  Median :0.8300   Median :1.1300   Median :0.930   Median :1.2750
##  Mean   :0.8843   Mean   :1.1933   Mean   :1.123   Mean   :1.8467
##  3rd Qu.:1.1100   3rd Qu.:1.3850   3rd Qu.:1.255   3rd Qu.:2.4325
##  Max.   :1.5000   Max.   :1.8100   Max.   :2.690   Max.   :5.2500
##  NA's   :1                         NA's   :1
##      FoxP3
##  Min.   :0.3000
##  1st Qu.:0.5500
##  Median :0.9700
##  Mean   :0.8942
##  3rd Qu.:1.1400
##  Max.   :1.6100
##  NA's   :3
```

```
# prep data
myd88_lung_dat$Strain <- factor(myd88_lung_dat$Strain, levels = c("MyD88_KO", "WT"))
myd88_lung_dat$Treatment <- factor(myd88_lung_dat$Treatment, levels = c("Control", "PNS"))
myd88_lung_dat$Litter <- factor(myd88_lung_dat$Litter, levels = c("P4", "Q5", "L4", "O6", "O4", "R4", "R5", "K6", "B4", "F
4", "L4_wt", "K5", "P5", "M6"))

myd88_lung_dat$Treatment <- relevel(myd88_lung_dat$Treatment, ref = "PNS")
myd88_lung_dat$Strain  <- relevel(myd88_lung_dat$Strain,  ref = "WT")
```

Lung Tissue *Ifng* Expression

```
##IFNg
# Fit linear mixed-effects model
# Include random intercept for litter
Ifng_model_Lg <- lmer(Ifng ~ Strain * Treatment + (1 | Litter), data = myd88_lung_dat)
summary(Ifng_model_Lg)
```

```
## Linear mixed model fit by REML. t-tests use Satterthwaite's method [
## lmerModLmerTest]
## Formula: Ifng ~ Strain * Treatment + (1 | Litter)
##    Data: myd88_lung_dat
##
## REML criterion at convergence: 135.4
##
## Scaled residuals:
##      Min       1Q   Median       3Q      Max
## -1.77515 -0.27345  0.01826  0.23271  3.01941
##
## Random effects:
##  Groups   Name        Variance Std.Dev.
##  Litter   (Intercept) 1.812    1.346
##  Residual             2.261    1.504
## Number of obs: 36, groups:  Litter, 14
##
## Fixed effects:
##                                  Estimate Std. Error      df t value Pr(>|t|)
## (Intercept)                        5.3001     0.7171  8.6873   7.391    5e-05
## StrainMyD88_KO                    -3.8641     1.2630  5.0445  -3.059   0.0278
## TreatmentControl                  -3.2208     1.1423  8.9119  -2.820   0.0203
## StrainMyD88_KO:TreatmentControl   2.8646     1.9382  6.3087   1.478   0.1875
##
## (Intercept)                      ***
## StrainMyD88_KO                   *
## TreatmentControl                 *
## StrainMyD88_KO:TreatmentControl
## ---
## Signif. codes:  0 '***' 0.001 '**' 0.01 '*' 0.05 '.' 0.1 ' ' 1
##
## Correlation of Fixed Effects:
##             (Intr) StMD88_KO TrtmnC
## StrnMD88_KO -0.568
## TrtmntCntrl -0.628  0.356
## SMD88_KO:TC  0.370 -0.652    -0.589
```

```
# Create a type III ANOVA table
Ifng_anova_table_Lg <- anova(Ifng_model_Lg, ddf = "Kenward-Roger")
print(Ifng_anova_table_Lg)
```

```
## Type III Analysis of Variance Table with Kenward-Roger's method
##                  Sum Sq Mean Sq NumDF  DenDF F value  Pr(>F)
## Strain          14.1941 14.1941     1 8.1322  6.2767 0.03618 *
## Treatment        7.6775  7.6775     1 8.1322  3.3951 0.10204
## Strain:Treatment  4.9240  4.9240     1 8.1322  2.1774 0.17768
## ---
## Signif. codes:  0 '***' 0.001 '**' 0.01 '*' 0.05 '.' 0.1 ' ' 1
```

```
Ifng_emcatcat_Lg <- emmeans(Ifng_model_Lg, ~ Treatment * Strain)

#Is there a difference relative to PNS WT offspring?
contrast(Ifng_emcatcat_Lg, method = "trt.vs.ctrl", ref = "PNS WT", adjust="BH")
```

```
##  contrast                  estimate   SE    df t.ratio p.value
##  Control WT - PNS WT          -3.22 1.15 11.16  -2.811  0.0198
##  PNS MyD88_KO - PNS WT        -3.86 1.26  6.60  -3.057  0.0198
##  Control MyD88_KO - PNS WT    -4.22 1.38  9.27  -3.068  0.0198
##
## Degrees-of-freedom method: kenward-roger
## P value adjustment: BH method for 3 tests
```

```
#Is there a difference relative to control WT?
contrast(Ifng_emcatcat_Lg, method = "trt.vs.ctrl", ref = "Control WT", adjust="BH")
```

```
##  contrast                      estimate   SE    df t.ratio p.value
##  PNS WT - Control WT              3.221 1.15 11.16   2.811  0.0502
##  PNS MyD88_KO - Control WT       -0.643 1.37  7.17  -0.470  0.6526
##  Control MyD88_KO - Control WT   -1.000 1.47  9.59  -0.678  0.6526
##
## Degrees-of-freedom method: kenward-roger
## P value adjustment: BH method for 3 tests
```

```
#Was there a difference between control and PNS in MyD88 KO mice?
Ifng_emcatcat_Lg2 <- emmeans(Ifng_model_Lg, ~ Treatment | Strain)
contrast(Ifng_emcatcat_Lg2, method = "pairwise", adjust="BH")
```

```
## Strain = WT:
##  contrast      estimate   SE    df t.ratio p.value
##  PNS - Control    3.221 1.15 11.16   2.811  0.0167
##
## Strain = MyD88_KO:
##  contrast      estimate   SE    df t.ratio p.value
##  PNS - Control    0.356 1.57  6.95   0.227  0.8267
##
## Degrees-of-freedom method: kenward-roger
```

Lung Tissue *Ifnb* Expression

```
##Ifnb
# Fit linear mixed-effects model
# Include random intercept for litter
Ifnb_model_Lg <- lmer(Ifnb ~ Strain * Treatment + (1 | Litter), data = myd88_lung_dat)
summary(Ifnb_model_Lg)
```

```
## Linear mixed model fit by REML. t-tests use Satterthwaite's method [
## lmerModLmerTest]
## Formula: Ifnb ~ Strain * Treatment + (1 | Litter)
##    Data: myd88_lung_dat
##
## REML criterion at convergence: 209.4
##
## Scaled residuals:
##      Min       1Q   Median       3Q      Max
## -2.19112 -0.21756 -0.01459  0.17663  3.00046
##
## Random effects:
##  Groups   Name        Variance Std.Dev.
##  Litter   (Intercept) 43.56    6.600
##  Residual             17.71    4.209
## Number of obs: 36, groups:  Litter, 14
##
## Fixed effects:
##                                 Estimate Std. Error      df t value Pr(>|t|)
## (Intercept)                       15.996      2.992   9.515   5.347 0.000385
## StrainMyD88_KO                   -14.249      5.666   7.603  -2.515 0.037547
## TreatmentControl                 -10.935      4.748   9.648  -2.303 0.044922
## StrainMyD88_KO:TreatmentControl   10.356      8.435   8.301   1.228 0.253186
##
## (Intercept)                     ***
## StrainMyD88_KO                  *
## TreatmentControl                *
## StrainMyD88_KO:TreatmentControl
## ---
## Signif. codes:  0 '***' 0.001 '**' 0.01 '*' 0.05 '.' 0.1 ' ' 1
##
## Correlation of Fixed Effects:
##             (Intr) StMD88_KO TrtmnC
## StrnMD88_KO -0.528
## TrtmntCntrl -0.630  0.333
## SMD88_KO:TC  0.355 -0.672    -0.563
```

```
# Create a type III ANOVA table
Ifnb_anova_table_Lg <- anova(Ifnb_model_Lg, ddf = "Kenward-Roger")
print(Ifnb_anova_table_Lg)
```

```
## Type III Analysis of Variance Table with Kenward-Roger's method
##                  Sum Sq Mean Sq NumDF  DenDF F value  Pr(>F)
## Strain           81.883  81.883     1 9.0505  4.6226 0.05987 .
## Treatment        32.986  32.986     1 9.0505  1.8622 0.20533
## Strain:Treatment 26.684  26.684     1 9.0505  1.5064 0.25066
## ---
## Signif. codes:  0 '***' 0.001 '**' 0.01 '*' 0.05 '.' 0.1 ' ' 1
```

```
Ifnb_emcatcat_Lg <- emmeans(Ifnb_model_Lg, ~ Treatment * Strain)

#Is there a difference relative to PNS WT offspring?
contrast(Ifnb_emcatcat_Lg, method = "trt.vs.ctrl", ref = "PNS WT", adjust="BH")
```

```
##  contrast                  estimate   SE    df t.ratio p.value
##  Control WT - PNS WT          -10.9 4.75 10.47  -2.301  0.0431
##  PNS MyD88_KO - PNS WT        -14.2 5.67  8.31  -2.514  0.0431
##  Control MyD88_KO - PNS WT    -14.8 5.87  9.56  -2.527  0.0431
##
## Degrees-of-freedom method: kenward-roger
## P value adjustment: BH method for 3 tests
```

```
#Is there a difference relative to control WT?
contrast(Ifnb_emcatcat_Lg, method = "trt.vs.ctrl", ref = "Control WT", adjust="BH")
```

```
##  contrast                       estimate   SE    df t.ratio p.value
##  PNS WT - Control WT              10.94 4.75 10.47   2.301  0.1293
##  PNS MyD88_KO - Control WT        -3.31 6.06  8.60  -0.546  0.5987
##  Control MyD88_KO - Control WT    -3.89 6.25  9.72  -0.623  0.5987
##
## Degrees-of-freedom method: kenward-roger
## P value adjustment: BH method for 3 tests
```

```
#Was there a difference between control and PNS in MyD88 KO mice?
Ifnb_emcatcat_Lg2 <- emmeans(Ifnb_model_Lg, ~ Treatment | Strain)
contrast(Ifnb_emcatcat_Lg2, method = "pairwise", adjust="BH")
```

```
## Strain = WT:
##  contrast      estimate   SE    df t.ratio p.value
##  PNS - Control   10.935 4.75 10.47   2.301  0.0431
##
## Strain = MyD88_KO:
##  contrast      estimate   SE    df t.ratio p.value
##  PNS - Control    0.579 6.97  8.48   0.083  0.9357
##
## Degrees-of-freedom method: kenward-roger
```

Lung Tissue *Isg15* Expression

```
##Isg15
# Fit linear mixed-effects model
# Include random intercept for litter
Isg15_model_Lg <- lmer(Isg15 ~ Strain * Treatment + (1 | Litter), data = myd88_lung_dat)
summary(Isg15_model_Lg)
```

```
## Linear mixed model fit by REML. t-tests use Satterthwaite's method [
## lmerModLmerTest]
## Formula: Isg15 ~ Strain * Treatment + (1 | Litter)
##    Data: myd88_lung_dat
##
## REML criterion at convergence: 10.1
##
## Scaled residuals:
##      Min       1Q   Median       3Q      Max
## -1.42189 -0.47205 -0.01185  0.21946  2.56303
##
## Random effects:
##  Groups   Name        Variance Std.Dev.
##  Litter   (Intercept) 0.04706  0.2169
##  Residual             0.04181  0.2045
## Number of obs: 35, groups:  Litter, 14
##
## Fixed effects:
##                                 Estimate Std. Error      df t value Pr(>|t|)
## (Intercept)                       0.8125     0.1086 11.7220   7.484 8.48e-06
## StrainMyD88_KO                    0.2958     0.1970  8.1076   1.502    0.171
## TreatmentControl                 -0.2002     0.1727 11.9500  -1.159    0.269
## StrainMyD88_KO:TreatmentControl   0.1238     0.2983  9.4117   0.415    0.688
##
## (Intercept)                     ***
## StrainMyD88_KO
## TreatmentControl
## StrainMyD88_KO:TreatmentControl
## ---
## Signif. codes:  0 '***' 0.001 '**' 0.01 '*' 0.05 '.' 0.1 ' ' 1
##
## Correlation of Fixed Effects:
##             (Intr) StMD88_KO TrtmnC
## StrnMD88_KO -0.551
## TrtmntCntrl -0.629  0.346
## SMD88_KO:TC  0.364 -0.660    -0.579
```

```
# Create a type III ANOVA table
Isg15_anova_table_Lg <- anova(Isg15_model_Lg, ddf = "Kenward-Roger")
print(Isg15_anova_table_Lg)
```

```
## Type III Analysis of Variance Table with Kenward-Roger's method
##                    Sum Sq  Mean Sq NumDF  DenDF F value  Pr(>F)
## Strain           0.239848 0.239848     1 8.4437  5.7366 0.04194 *
## Treatment        0.035872 0.035872     1 8.4437  0.8580 0.38002
## Strain:Treatment 0.007180 0.007180     1 8.4437  0.1717 0.68891
## ---
## Signif. codes:  0 '***' 0.001 '**' 0.01 '*' 0.05 '.' 0.1 ' ' 1
```

```
Isg15_emcatcat_Lg <- emmeans(Isg15_model_Lg, ~ Treatment * Strain)

#Is there a difference relative to PNS WT offspring?
contrast(Isg15_emcatcat_Lg, method = "trt.vs.ctrl", ref = "PNS WT", adjust="BH")
```

```
##  contrast                  estimate    SE    df t.ratio p.value
##  Control WT - PNS WT         -0.200 0.173 10.85  -1.156  0.3223
##  PNS MyD88_KO - PNS WT        0.296 0.197  7.23   1.501  0.3223
##  Control MyD88_KO - PNS WT    0.219 0.210  9.31   1.045  0.3223
##
## Degrees-of-freedom method: kenward-roger
## P value adjustment: BH method for 3 tests
```

```
#Is there a difference relative to control WT?
contrast(Isg15_emcatcat_Lg, method = "trt.vs.ctrl", ref = "Control WT", adjust="BH")
```

```
##  contrast                      estimate    SE    df t.ratio p.value
##  PNS WT - Control WT              0.200 0.173 10.85   1.156  0.2724
##  PNS MyD88_KO - Control WT        0.496 0.213  7.70   2.334  0.1386
##  Control MyD88_KO - Control WT    0.420 0.224  9.57   1.870  0.1386
##
## Degrees-of-freedom method: kenward-roger
## P value adjustment: BH method for 3 tests
```

```
#Was there a difference between control and PNS in MyD88 KO mice?
Isg15_emcatcat_Lg2 <- emmeans(Isg15_model_Lg, ~ Treatment | Strain)
contrast(Isg15_emcatcat_Lg2, method = "pairwise", adjust="BH")
```

```
## Strain = WT:
##  contrast      estimate    SE    df t.ratio p.value
##  PNS - Control   0.2002 0.173 10.85   1.156  0.2724
##
## Strain = MyD88_KO:
##  contrast      estimate    SE    df t.ratio p.value
##  PNS - Control   0.0764 0.243  7.49   0.314  0.7620
##
## Degrees-of-freedom method: kenward-roger
```

Lung Tissue *Il1b* Expression

```
##Il1b
# Fit linear mixed-effects model
# Include random intercept for litter
Il1b_model_Lg <- lmer(Il1b ~ Strain * Treatment + (1 | Litter), data = myd88_lung_dat)
summary(Il1b_model_Lg)
```

```
## Linear mixed model fit by REML. t-tests use Satterthwaite's method [
## lmerModLmerTest]
## Formula: Il1b ~ Strain * Treatment + (1 | Litter)
##    Data: myd88_lung_dat
##
## REML criterion at convergence: 108.5
##
## Scaled residuals:
##      Min       1Q   Median       3Q      Max
## -2.72614 -0.28153 -0.03258  0.28399  2.59267
##
## Random effects:
##  Groups   Name        Variance Std.Dev.
##  Litter   (Intercept) 8.1308   2.8515
##  Residual             0.4735   0.6881
## Number of obs: 35, groups:  Litter, 14
##
## Fixed effects:
##                                 Estimate Std. Error     df t value Pr(>|t|)
## (Intercept)                        4.893      1.186 10.073   4.124  0.00203 **
## StrainMyD88_KO                    -3.233      2.347  9.647  -1.377  0.19954
## TreatmentControl                  -3.452      1.875 10.045  -1.841  0.09523 .
## StrainMyD88_KO:TreatmentControl    2.884      3.432  9.767   0.840  0.42087
## ---
## Signif. codes:  0 '***' 0.001 '**' 0.01 '*' 0.05 '.' 0.1 ' ' 1
##
## Correlation of Fixed Effects:
##             (Intr) StMD88_KO TrtmnC
## StrnMD88_KO -0.505
## TrtmntCntrl -0.633  0.320
## SMD88_KO:TC  0.346 -0.684    -0.546
```

```
# Create a type III ANOVA table
Il1b_anova_table_Lg <- anova(Il1b_model_Lg, ddf = "Kenward-Roger")
print(Il1b_anova_table_Lg)
```

```
## Type III Analysis of Variance Table with Kenward-Roger's method
##                   Sum Sq Mean Sq NumDF  DenDF F value Pr(>F)
## Strain           0.51567 0.51567     1 9.7924  1.0890 0.3218
## Treatment        0.64960 0.64960     1 9.7924  1.3719 0.2692
## Strain:Treatment 0.33429 0.33429     1 9.7924  0.7060 0.4208
```

```
Il1b_emcatcat_Lg <- emmeans(Il1b_model_Lg, ~ Treatment * Strain)

#Is there a difference relative to PNS WT offspring?
contrast(Il1b_emcatcat_Lg, method = "trt.vs.ctrl", ref = "PNS WT", adjust="BH")
```

```
##  contrast                  estimate   SE    df t.ratio p.value
##  Control WT - PNS WT          -3.45 1.87 10.07  -1.841  0.1995
##  PNS MyD88_KO - PNS WT        -3.23 2.35  9.67  -1.377  0.1995
##  Control MyD88_KO - PNS WT    -3.80 2.36  9.89  -1.610  0.1995
##
## Degrees-of-freedom method: kenward-roger
## P value adjustment: BH method for 3 tests
```

```
#Is there a difference relative to control WT?
contrast(Il1b_emcatcat_Lg, method = "trt.vs.ctrl", ref = "Control WT", adjust="BH")
```

```
##  contrast                      estimate   SE   df t.ratio p.value
##  PNS WT - Control WT              3.452 1.87 10.1   1.841  0.2855
##  PNS MyD88_KO - Control WT        0.219 2.49  9.7   0.088  0.9317
##  Control MyD88_KO - Control WT   -0.349 2.50  9.9  -0.139  0.9317
##
## Degrees-of-freedom method: kenward-roger
## P value adjustment: BH method for 3 tests
```

```
#Was there a difference between control and PNS in MyD88 KO mice?
Il1b_emcatcat_Lg2 <- emmeans(Il1b_model_Lg, ~ Treatment | Strain)
contrast(Il1b_emcatcat_Lg2, method = "pairwise", adjust="BH")
```

```
## Strain = WT:
##  contrast      estimate   SE    df t.ratio p.value
##  PNS - Control    3.452 1.87 10.07   1.841  0.0952
##
## Strain = MyD88_KO:
##  contrast      estimate   SE    df t.ratio p.value
##  PNS - Control    0.568 2.88  9.68   0.198  0.8474
##
## Degrees-of-freedom method: kenward-roger
```

Lung Tissue *Il6* Expression

```
##Il6
# Fit linear mixed-effects model
# Include random intercept for litter
Il6_model_Lg <- lmer(Il6 ~ Strain * Treatment + (1 | Litter), data = myd88_lung_dat)
summary(Il6_model_Lg)
```

```
## Linear mixed model fit by REML. t-tests use Satterthwaite's method [
## lmerModLmerTest]
## Formula: Il6 ~ Strain * Treatment + (1 | Litter)
##    Data: myd88_lung_dat
##
## REML criterion at convergence: 88.9
##
## Scaled residuals:
##      Min       1Q   Median       3Q      Max
## -1.84791 -0.17258 -0.01394  0.17765  2.23302
##
## Random effects:
##  Groups   Name        Variance Std.Dev.
##  Litter   (Intercept) 0.2719   0.5214
##  Residual             0.6374   0.7983
## Number of obs: 35, groups:  Litter, 14
##
## Fixed effects:
##                                  Estimate Std. Error      df t value Pr(>|t|)
## (Intercept)                        3.5746     0.3233  7.3771  11.056 7.45e-06
## StrainMyD88_KO                    -2.4504     0.5418  3.3312  -4.522  0.01610
## TreatmentControl                  -2.1997     0.5161  7.6198  -4.262  0.00308
## StrainMyD88_KO:TreatmentControl   2.1183     0.8494  4.6278   2.494  0.05883
##
## (Intercept)                     ***
## StrainMyD88_KO                  *
## TreatmentControl                **
## StrainMyD88_KO:TreatmentControl .
## ---
## Signif. codes:  0 '***' 0.001 '**' 0.01 '*' 0.05 '.' 0.1 ' ' 1
##
## Correlation of Fixed Effects:
##             (Intr) StMD88_KO TrtmnC
## StrnMD88_KO -0.597
## TrtmntCntrl -0.626  0.374
## SMD88_KO:TC  0.381 -0.638    -0.608
```

```
# Create a type III ANOVA table
Il6_anova_table_Lg <- anova(Il6_model_Lg, ddf = "Kenward-Roger")
print(Il6_anova_table_Lg)
```

```
## Type III Analysis of Variance Table with Kenward-Roger's method
##                  Sum Sq Mean Sq NumDF  DenDF F value  Pr(>F)
## Strain           6.8018  6.8018     1 7.7074 10.6721 0.01201 *
## Treatment        4.5708  4.5708     1 7.7074  7.1716 0.02896 *
## Strain:Treatment 3.9417  3.9417     1 7.7074  6.1846 0.03878 *
## ---
## Signif. codes:  0 '***' 0.001 '**' 0.01 '*' 0.05 '.' 0.1 ' ' 1
```

```
Il6_emcatcat_Lg <- emmeans(Il6_model_Lg, ~ Treatment * Strain)

#Is there a difference relative to PNS WT offspring?
contrast(Il6_emcatcat_Lg, method = "trt.vs.ctrl", ref = "PNS WT", adjust="BH")
```

```
##  contrast                  estimate    SE    df t.ratio p.value
##  Control WT - PNS WT          -2.20 0.518 11.80  -4.244  0.0035
##  PNS MyD88_KO - PNS WT        -2.45 0.542  5.74  -4.517  0.0045
##  Control MyD88_KO - PNS WT    -2.53 0.611  9.19  -4.145  0.0036
##
## Degrees-of-freedom method: kenward-roger
## P value adjustment: BH method for 3 tests
```

```
#Is there a difference relative to control WT?
contrast(Il6_emcatcat_Lg, method = "trt.vs.ctrl", ref = "Control WT", adjust="BH")
```

```
##  contrast                      estimate    SE    df t.ratio p.value
##  PNS WT - Control WT              2.200 0.518 11.80   4.244  0.0035
##  PNS MyD88_KO - Control WT       -0.251 0.594  6.47  -0.422  0.6864
##  Control MyD88_KO - Control WT   -0.332 0.657  9.62  -0.506  0.6864
##
## Degrees-of-freedom method: kenward-roger
## P value adjustment: BH method for 3 tests
```

```
#Was there a difference between control and PNS in MyD88 KO mice?
Il6_emcatcat_Lg2 <- emmeans(Il6_model_Lg, ~ Treatment | Strain)
contrast(Il6_emcatcat_Lg2, method = "pairwise", adjust="BH")
```

```
## Strain = WT:
##  contrast      estimate    SE    df t.ratio p.value
##  PNS - Control   2.1997 0.518 11.80   4.244  0.0012
##
## Strain = MyD88_KO:
##  contrast      estimate    SE    df t.ratio p.value
##  PNS - Control   0.0814 0.676  6.15   0.120  0.9080
##
## Degrees-of-freedom method: kenward-roger
```

Lung Tissue *Tnfa* Expression

```
##Tnf
# Fit linear mixed-effects model
# Include random intercept for litter
Tnf_model_Lg <- lmer(Tnf ~ Strain * Treatment + (1 | Litter), data = myd88_lung_dat)
summary(Tnf_model_Lg)
```

```
## Linear mixed model fit by REML. t-tests use Satterthwaite's method [
## lmerModLmerTest]
## Formula: Tnf ~ Strain * Treatment + (1 | Litter)
##    Data: myd88_lung_dat
##
## REML criterion at convergence: 38.2
##
## Scaled residuals:
##     Min      1Q  Median      3Q     Max
## -2.0140 -0.5093 -0.1122  0.5630  2.6810
##
## Random effects:
##  Groups   Name        Variance Std.Dev.
##  Litter   (Intercept) 0.16648  0.4080
##  Residual             0.09277  0.3046
## Number of obs: 35, groups:  Litter, 14
##
## Fixed effects:
##                                 Estimate Std. Error      df t value Pr(>|t|)
## (Intercept)                       1.5089     0.1912  9.6151   7.890 1.68e-05
## StrainMyD88_KO                   -0.5806     0.3574  7.2822  -1.624   0.1466
## TreatmentControl                 -0.6659     0.3038  9.7784  -2.192   0.0537
## StrainMyD88_KO:TreatmentControl   0.7741     0.5347  8.1156   1.448   0.1852
##
## (Intercept)                     ***
## StrainMyD88_KO
## TreatmentControl                .
## StrainMyD88_KO:TreatmentControl
## ---
## Signif. codes:  0 '***' 0.001 '**' 0.01 '*' 0.05 '.' 0.1 ' ' 1
##
## Correlation of Fixed Effects:
##             (Intr) StMD88_KO TrtmnC
## StrnMD88_KO -0.535
## TrtmntCntrl -0.630  0.337
## SMD88_KO:TC  0.358 -0.668    -0.568
```

```
# Create a type III ANOVA table
Tnf_anova_table_Lg <- anova(Tnf_model_Lg, ddf = "Kenward-Roger")
print(Tnf_anova_table_Lg)
```

```
## Type III Analysis of Variance Table with Kenward-Roger's method
##                    Sum Sq Mean Sq NumDF  DenDF F value Pr(>F)
## Strain           0.04857 0.04857     1 8.8378  0.5236 0.4880
## Treatment        0.10081 0.10081     1 8.8378  1.0867 0.3249
## Strain:Treatment 0.19418 0.19418     1 8.8378  2.0932 0.1825
```

```
Tnf_emcatcat_Lg <- emmeans(Tnf_model_Lg, ~ Treatment * Strain)

#Is there a difference relative to PNS WT offspring?
contrast(Tnf_emcatcat_Lg, method = "trt.vs.ctrl", ref = "PNS WT", adjust="BH")
```

```
##  contrast                  estimate    SE    df t.ratio p.value
##  Control WT - PNS WT         -0.666 0.304 10.58  -2.189  0.1560
##  PNS MyD88_KO - PNS WT       -0.581 0.358  7.96  -1.624  0.2149
##  Control MyD88_KO - PNS WT   -0.472 0.373  9.45  -1.266  0.2357
##
## Degrees-of-freedom method: kenward-roger
## P value adjustment: BH method for 3 tests
```

```
#Is there a difference relative to control WT?
contrast(Tnf_emcatcat_Lg, method = "trt.vs.ctrl", ref = "Control WT", adjust="BH")
```

```
##  contrast                      estimate    SE    df t.ratio p.value
##  PNS WT - Control WT             0.6659 0.304 10.58   2.189  0.1560
##  PNS MyD88_KO - Control WT       0.0853 0.384  8.31   0.222  0.8294
##  Control MyD88_KO - Control WT   0.1935 0.398  9.65   0.486  0.8294
##
## Degrees-of-freedom method: kenward-roger
## P value adjustment: BH method for 3 tests
```

```
#Was there a difference between control and PNS in MyD88 KO mice?
Tnf_emcatcat_Lg2 <- emmeans(Tnf_model_Lg, ~ Treatment | Strain)
contrast(Tnf_emcatcat_Lg2, method = "pairwise", adjust="BH")
```

```
## Strain = WT:
##  contrast      estimate    SE    df t.ratio p.value
##  PNS - Control    0.666 0.304 10.58   2.189  0.0520
##
## Strain = MyD88_KO:
##  contrast      estimate    SE    df t.ratio p.value
##  PNS - Control   -0.108 0.440  8.14  -0.246  0.8120
##
## Degrees-of-freedom method: kenward-roger
```

Lung Tissue *Ahr* Expression

```
##Ahr
# Fit linear mixed-effects model
# Include random intercept for litter
Ahr_model_Lg <- lmer(Ahr ~ Strain * Treatment + (1 | Litter), data = myd88_lung_dat)
summary(Ahr_model_Lg)
```

```
## Linear mixed model fit by REML. t-tests use Satterthwaite's method [
## lmerModLmerTest]
## Formula: Ahr ~ Strain * Treatment + (1 | Litter)
##    Data: myd88_lung_dat
##
## REML criterion at convergence: 74.8
##
## Scaled residuals:
##      Min       1Q   Median       3Q      Max
## -1.76075 -0.29361 -0.04612  0.16424  2.69267
##
## Random effects:
##  Groups   Name        Variance Std.Dev.
##  Litter   (Intercept) 0.4361   0.6603
##  Residual             0.3002   0.5479
## Number of obs: 36, groups:  Litter, 14
##
## Fixed effects:
##                                 Estimate Std. Error      df t value Pr(>|t|)
## (Intercept)                       3.0330     0.3180 10.2764   9.537 1.99e-06
## StrainMyD88_KO                   -2.1057     0.5851  7.3588  -3.599  0.00805
## TreatmentControl                 -0.6645     0.5055 10.4665  -1.315  0.21673
## StrainMyD88_KO:TreatmentControl   0.7353     0.8814  8.4163   0.834  0.42716
##
## (Intercept)                     ***
## StrainMyD88_KO                  **
## TreatmentControl
## StrainMyD88_KO:TreatmentControl
## ---
## Signif. codes:  0 '***' 0.001 '**' 0.01 '*' 0.05 '.' 0.1 ' ' 1
##
## Correlation of Fixed Effects:
##             (Intr) StMD88_KO TrtmnC
## StrnMD88_KO -0.543
## TrtmntCntrl -0.629  0.342
## SMD88_KO:TC  0.361 -0.664    -0.574
```

```
# Create a type III ANOVA table
Ahr_anova_table_Lg <- anova(Ahr_model_Lg, ddf = "Kenward-Roger")
print(Ahr_anova_table_Lg)
```

```
## Type III Analysis of Variance Table with Kenward-Roger's method
##                  Sum Sq Mean Sq NumDF  DenDF F value   Pr(>F)
## Strain           4.6619  4.6619     1 8.6392 15.5301 0.003686 **
## Treatment        0.1360  0.1360     1 8.6392  0.4531 0.518477
## Strain:Treatment 0.2086  0.2086     1 8.6392  0.6949 0.426948
## ---
## Signif. codes:  0 '***' 0.001 '**' 0.01 '*' 0.05 '.' 0.1 ' ' 1
```

```
Ahr_emcatcat_Lg <- emmeans(Ahr_model_Lg, ~ Treatment * Strain)

#Is there a difference relative to PNS WT offspring?
contrast(Ahr_emcatcat_Lg, method = "trt.vs.ctrl", ref = "PNS WT", adjust="BH")
```

```
##  contrast                  estimate    SE    df t.ratio p.value
##  Control WT - PNS WT         -0.665 0.506 10.72  -1.312  0.2168
##  PNS MyD88_KO - PNS WT       -2.106 0.585  7.56  -3.597  0.0132
##  Control MyD88_KO - PNS WT   -2.035 0.618  9.41  -3.293  0.0132
##
## Degrees-of-freedom method: kenward-roger
## P value adjustment: BH method for 3 tests
```

```
#Is there a difference relative to control WT?
contrast(Ahr_emcatcat_Lg, method = "trt.vs.ctrl", ref = "Control WT", adjust="BH")
```

```
##  contrast                      estimate    SE    df t.ratio p.value
##  PNS WT - Control WT              0.665 0.506 10.72   1.312  0.2168
##  PNS MyD88_KO - Control WT       -1.441 0.630  7.98  -2.289  0.0984
##  Control MyD88_KO - Control WT   -1.370 0.660  9.63  -2.077  0.0984
##
## Degrees-of-freedom method: kenward-roger
## P value adjustment: BH method for 3 tests
```

```
#Was there a difference between control and PNS in MyD88 KO mice?
Ahr_emcatcat_Lg2 <- emmeans(Ahr_model_Lg, ~ Treatment | Strain)
contrast(Ahr_emcatcat_Lg2, method = "pairwise", adjust="BH")
```

```
## Strain = WT:
##  contrast      estimate    SE    df t.ratio p.value
##  PNS - Control   0.6645 0.506 10.72   1.312  0.2168
##
## Strain = MyD88_KO:
##  contrast      estimate    SE    df t.ratio p.value
##  PNS - Control  -0.0708 0.722  7.81  -0.098  0.9244
##
## Degrees-of-freedom method: kenward-roger
```

Lung Tissue *Muc5ac* Expression

```
##Muc5ac
# Fit linear mixed-effects model
# Include random intercept for litter
Muc5ac_model_Lg <- lmer(Muc5ac ~ Strain * Treatment + (1 | Litter), data = myd88_lung_dat)
summary(Muc5ac_model_Lg)
```

```
## Linear mixed model fit by REML. t-tests use Satterthwaite's method [
## lmerModLmerTest]
## Formula: Muc5ac ~ Strain * Treatment + (1 | Litter)
##    Data: myd88_lung_dat
##
## REML criterion at convergence: 83.3
##
## Scaled residuals:
##      Min       1Q   Median       3Q      Max
## -1.96103 -0.39781 -0.00704  0.26566  2.61492
##
## Random effects:
##  Groups   Name        Variance Std.Dev.
##  Litter   (Intercept) 0.6740   0.8210
##  Residual             0.4044   0.6359
## Number of obs: 35, groups:  Litter, 14
##
## Fixed effects:
##                                   Estimate Std. Error      df t value Pr(>|t|)
## (Intercept)                         1.5941     0.3883  8.1784   4.106  0.00325 **
## StrainMyD88_KO                     -1.0782     0.7221  5.9670  -1.493  0.18626
## TreatmentControl                   -1.2780     0.6169  8.3332  -2.072  0.07065 .
## StrainMyD88_KO:TreatmentControl   1.9099     1.0825  6.7499   1.764  0.12261
## ---
## Signif. codes:  0 '***' 0.001 '**' 0.01 '*' 0.05 '.' 0.1 ' ' 1
##
## Correlation of Fixed Effects:
##             (Intr) StMD88_KO TrtmnC
## StrnMD88_KO -0.538
## TrtmntCntrl -0.629  0.338
## SMD88_KO:TC  0.359 -0.667    -0.570
```

```
# Create a type III ANOVA table
Muc5ac_anova_table_Lg <- anova(Muc5ac_model_Lg, ddf = "Kenward-Roger")
print(Muc5ac_anova_table_Lg)
```

```
## Type III Analysis of Variance Table with Kenward-Roger's method
##                   Sum Sq Mean Sq NumDF  DenDF F value Pr(>F)
## Strain           0.02096 0.02096     1 8.7733  0.0518 0.8251
## Treatment        0.14388 0.14388     1 8.7733  0.3558 0.5659
## Strain:Treatment 1.25718 1.25718     1 8.7733  3.1087 0.1126
```

```
Muc5ac_emcatcat_Lg <- emmeans(Muc5ac_model_Lg, ~ Treatment * Strain)

#Is there a difference relative to PNS WT offspring?
contrast(Muc5ac_emcatcat_Lg, method = "trt.vs.ctrl", ref = "PNS WT", adjust="BH")
```

```
##  contrast                  estimate    SE    df t.ratio p.value
##  Control WT - PNS WT         -1.278 0.618 10.62  -2.068  0.1915
##  PNS MyD88_KO - PNS WT       -1.078 0.722  7.83  -1.493  0.2620
##  Control MyD88_KO - PNS WT   -0.446 0.756  9.43  -0.590  0.5690
##
## Degrees-of-freedom method: kenward-roger
## P value adjustment: BH method for 3 tests
```

```
#Is there a difference relative to control WT?
contrast(Muc5ac_emcatcat_Lg, method = "trt.vs.ctrl", ref = "Control WT", adjust="BH")
```

```
##  contrast                      estimate    SE    df t.ratio p.value
##  PNS WT - Control WT              1.278 0.618 10.62   2.068  0.1915
##  PNS MyD88_KO - Control WT        0.200 0.775  8.20   0.258  0.8030
##  Control MyD88_KO - Control WT    0.832 0.807  9.64   1.030  0.4921
##
## Degrees-of-freedom method: kenward-roger
## P value adjustment: BH method for 3 tests
```

```
#Was there a difference between control and PNS in MyD88 KO mice?
Muc5ac_emcatcat_Lg2 <- emmeans(Muc5ac_model_Lg, ~ Treatment | Strain)
contrast(Muc5ac_emcatcat_Lg2, method = "pairwise", adjust="BH")
```

```
## Strain = WT:
##  contrast      estimate    SE    df t.ratio p.value
##  PNS - Control    1.278 0.618 10.62   2.068  0.0638
##
## Strain = MyD88_KO:
##  contrast      estimate    SE    df t.ratio p.value
##  PNS - Control   -0.632 0.890  8.03  -0.710  0.4977
##
## Degrees-of-freedom method: kenward-roger
```

Lung Tissue *Muc5b* Expression

```
##Muc5b
# Fit linear mixed-effects model
# Include random intercept for litter
Muc5b_model_Lg <- lmer(Muc5b ~ Strain * Treatment + (1 | Litter), data = myd88_lung_dat)
summary(Muc5b_model_Lg)
```

```
## Linear mixed model fit by REML. t-tests use Satterthwaite's method [
## lmerModLmerTest]
## Formula: Muc5b ~ Strain * Treatment + (1 | Litter)
##    Data: myd88_lung_dat
##
## REML criterion at convergence: 113.1
##
## Scaled residuals:
##      Min       1Q   Median       3Q      Max
## -2.02258 -0.20437 -0.06257  0.25794  2.53330
##
## Random effects:
##  Groups   Name        Variance Std.Dev.
##  Litter   (Intercept) 0.3777   0.6146
##  Residual             1.4894   1.2204
## Number of obs: 35, groups:  Litter, 14
##
## Fixed effects:
##                                 Estimate Std. Error      df t value Pr(>|t|)
## (Intercept)                       2.7803     0.4477  6.6200   6.211 0.000549
## StrainMyD88_KO                   -2.4411     0.7165  2.3667  -3.407 0.060107
## TreatmentControl                 -2.3707     0.7156  6.8663  -3.313 0.013256
## StrainMyD88_KO:TreatmentControl   3.3040     1.1474  3.6413   2.880 0.050383
##
## (Intercept)                     ***
## StrainMyD88_KO                  .
## TreatmentControl                *
## StrainMyD88_KO:TreatmentControl .
## ---
## Signif. codes:  0 '***' 0.001 '**' 0.01 '*' 0.05 '.' 0.1 ' ' 1
##
## Correlation of Fixed Effects:
##             (Intr) StMD88_KO TrtmnC
## StrnMD88_KO -0.625
## TrtmntCntrl -0.626  0.391
## SMD88_KO:TC  0.390 -0.624    -0.624
```

```
# Create a type III ANOVA table
Muc5b_anova_table_Lg <- anova(Muc5b_model_Lg, ddf = "Kenward-Roger")
print(Muc5b_anova_table_Lg)
```

```
## Type III Analysis of Variance Table with Kenward-Roger's method
##                  Sum Sq Mean Sq NumDF DenDF F value  Pr(>F)
## Strain           2.7967  2.7967     1 7.517  1.8778 0.21011
## Treatment        2.3197  2.3197     1 7.517  1.5575 0.24951
## Strain:Treatment 12.2579 12.2579     1 7.517  8.2302 0.02227 *
## ---
## Signif. codes:  0 '***' 0.001 '**' 0.01 '*' 0.05 '.' 0.1 ' ' 1
```

```
Muc5b_emcatcat_Lg <- emmeans(Muc5b_model_Lg, ~ Treatment * Strain)

#Is there a difference relative to PNS WT offspring?
contrast(Muc5b_emcatcat_Lg, method = "trt.vs.ctrl", ref = "PNS WT", adjust="BH")
```

```
##  contrast                  estimate    SE    df t.ratio p.value
##  Control WT - PNS WT          -2.37 0.719 12.59  -3.297  0.0180
##  PNS MyD88_KO - PNS WT        -2.44 0.718  5.14  -3.402  0.0276
##  Control MyD88_KO - PNS WT    -1.51 0.836  9.35  -1.804  0.1034
##
## Degrees-of-freedom method: kenward-roger
## P value adjustment: BH method for 3 tests
```

```
#Is there a difference relative to control WT?
contrast(Muc5b_emcatcat_Lg, method = "trt.vs.ctrl", ref = "Control WT", adjust="BH")
```

```
##  contrast                      estimate    SE    df t.ratio p.value
##  PNS WT - Control WT             2.3707 0.719 12.59   3.297  0.0180
##  PNS MyD88_KO - Control WT      -0.0704 0.793  6.00  -0.089  0.9321
##  Control MyD88_KO - Control WT   0.8629 0.901  9.87   0.958  0.5415
##
## Degrees-of-freedom method: kenward-roger
## P value adjustment: BH method for 3 tests
```

```
#Was there a difference between control and PNS in MyD88 KO mice?
Muc5b_emcatcat_Lg2 <- emmeans(Muc5b_model_Lg, ~ Treatment | Strain)
contrast(Muc5b_emcatcat_Lg2, method = "pairwise", adjust="BH")
```

```
## Strain = WT:
##  contrast      estimate    SE    df t.ratio p.value
##  PNS - Control    2.371 0.719 12.59   3.297  0.0060
##
## Strain = MyD88_KO:
##  contrast      estimate    SE    df t.ratio p.value
##  PNS - Control   -0.933 0.900  5.63  -1.037  0.3420
##
## Degrees-of-freedom method: kenward-roger
```

MyD88 KO vs C57BL6 WT Ileum Tissue Gene Expression–Figure 8b

Read in Ileum Gene Expression Data

```
myd88_ileum_dat <- read_csv("Myd88_d7_IT_expression.csv")
```

```
## Rows: 36 Columns: 20
## ── Column specification ────────────────────────────────────────────────────────
## Delimiter: ","
## chr  (4): SampleID, Treatment, Strain, Litter
## dbl (16): Ifnb, Ifng, Isg15, Muc2, Il1b, Il4, Il5, Il6, Il10, Il13, Il17a, I...
##
## ℹ Use `spec()` to retrieve the full column specification for this data.
## ℹ Specify the column types or set `show_col_types = FALSE` to quiet this message.
```

```
summary(myd88_ileum_dat) # To make sure that your data looks correct (i.e., R is interpreting it the way you want)
```

```
##    SampleID          Treatment            Strain             Litter
##  Length:36          Length:36          Length:36          Length:36
##  Class :character   Class :character   Class :character   Class :character
##  Mode  :character   Mode  :character   Mode  :character   Mode  :character
##
##
##
##
##       Ifnb             Ifng            Isg15            Muc2
##  Min.   : 0.380   Min.   : 0.650   Min.   :0.060   Min.   :0.0900
##  1st Qu.: 0.870   1st Qu.: 0.890   1st Qu.:0.615   1st Qu.:0.4775
##  Median : 2.370   Median : 1.850   Median :1.000   Median :0.9000
##  Mean   : 4.146   Mean   : 3.459   Mean   :1.412   Mean   :0.9933
##  3rd Qu.: 5.320   3rd Qu.: 4.420   3rd Qu.:2.020   3rd Qu.:1.1975
##  Max.   :16.250   Max.   :21.370   Max.   :5.810   Max.   :3.0000
##  NA's   :3        NA's   :3        NA's   :1
##       Il1b             Il4              Il5             Il6
##  Min.   :0.3800   Min.   :0.0100   Min.   :0.380   Min.   : 0.530
##  1st Qu.:0.8325   1st Qu.:0.2600   1st Qu.:0.975   1st Qu.: 0.950
##  Median :1.1150   Median :0.5300   Median :1.390   Median : 1.600
##  Mean   :1.9241   Mean   :0.5969   Mean   :1.430   Mean   : 2.635
##  3rd Qu.:2.2125   3rd Qu.:0.8850   3rd Qu.:1.798   3rd Qu.: 2.850
##  Max.   :7.6800   Max.   :1.8200   Max.   :2.800   Max.   :12.680
##  NA's   :2        NA's   :1        NA's   :2       NA's   :3
##       Il10             Il13           Il17a            Il18
##  Min.   :0.1900   Min.   :0.070   Min.   :0.120   Min.   :0.560
##  1st Qu.:0.5750   1st Qu.:0.300   1st Qu.:0.525   1st Qu.:0.900
##  Median :0.8600   Median :0.710   Median :0.870   Median :1.030
##  Mean   :0.9046   Mean   :0.712   Mean   :1.070   Mean   :1.666
##  3rd Qu.:1.1950   3rd Qu.:1.010   3rd Qu.:1.290   3rd Qu.:1.975
##  Max.   :2.3800   Max.   :1.890   Max.   :3.250   Max.   :5.570
##  NA's   :1        NA's   :1       NA's   :1       NA's   :1
##      Tgfb1            Tnf             Ahr             FoxP3
##  Min.   :0.770   Min.   :0.770   Min.   : 0.800   Min.   :0.210
##  1st Qu.:0.940   1st Qu.:0.990   1st Qu.: 0.920   1st Qu.:0.745
##  Median :1.340   Median :1.530   Median : 2.800   Median :1.240
##  Mean   :1.528   Mean   :2.522   Mean   : 4.761   Mean   :1.813
##  3rd Qu.:2.110   3rd Qu.:2.995   3rd Qu.: 6.385   3rd Qu.:2.175
##  Max.   :2.610   Max.   :8.730   Max.   :17.310   Max.   :6.320
##  NA's   :1       NA's   :1       NA's   :2        NA's   :1
```

```
# prep data
myd88_ileum_dat$Strain <- factor(myd88_ileum_dat$Strain, levels = c("MyD88_KO", "WT"))
myd88_ileum_dat$Treatment <- factor(myd88_ileum_dat$Treatment, levels = c("Control", "PNS"))
myd88_ileum_dat$Litter <- factor(myd88_ileum_dat$Litter, levels = c("P4", "Q5", "L4", "O6", "O4", "R4", "R5", "K6", "B4", "F
4", "L4_wt", "K5", "P5", "M6"))

myd88_ileum_dat$Treatment <- relevel(myd88_ileum_dat$Treatment, ref = "PNS")
myd88_ileum_dat$Strain  <- relevel(myd88_ileum_dat$Strain,  ref = "WT")
```

Ileum Tissue *Ifng* Expression

```
##IFNg
# Fit linear mixed-effects model
# Include random intercept for litter
Ifng_model_IT <- lmer(Ifng ~ Strain * Treatment + (1 | Litter), data = myd88_ileum_dat)
summary(Ifng_model_IT)
```

```
## Linear mixed model fit by REML. t-tests use Satterthwaite's method [
## lmerModLmerTest]
## Formula: Ifng ~ Strain * Treatment + (1 | Litter)
##    Data: myd88_ileum_dat
##
## REML criterion at convergence: 157.8
##
## Scaled residuals:
##      Min       1Q   Median       3Q      Max
## -2.09906 -0.22169 -0.02680  0.08431  3.13793
##
## Random effects:
##  Groups   Name        Variance Std.Dev.
##  Litter   (Intercept) 12.089   3.477
##  Residual              5.937   2.437
## Number of obs: 33, groups:  Litter, 14
##
## Fixed effects:
##                                 Estimate Std. Error     df t value Pr(>|t|)
## (Intercept)                        8.570      1.629  9.645   5.259 0.000415 ***
## StrainMyD88_KO                    -7.314      3.032  7.188  -2.412 0.045726 *
## TreatmentControl                  -6.492      2.599  9.987  -2.497 0.031622 *
## StrainMyD88_KO:TreatmentControl    6.270      4.535  8.011   1.382 0.204144
## ---
## Signif. codes:  0 '***' 0.001 '**' 0.01 '*' 0.05 '.' 0.1 ' ' 1
##
## Correlation of Fixed Effects:
##             (Intr) StMD88_KO TrtmnC
## StrnMD88_KO -0.537
## TrtmntCntrl -0.627  0.337
## SMD88_KO:TC  0.359 -0.669    -0.573
```

```
# Create a type III ANOVA table
Ifng_anova_table_IT <- anova(Ifng_model_IT, ddf = "Kenward-Roger")
print(Ifng_anova_table_IT)
```

```
## Type III Analysis of Variance Table with Kenward-Roger's method
##                  Sum Sq Mean Sq NumDF DenDF F value  Pr(>F)
## Strain           20.132  20.132     1 8.677  3.3911 0.09991 .
## Treatment        12.987  12.987     1 8.677  2.1876 0.17449
## Strain:Treatment 11.328  11.328     1 8.677  1.9081 0.20170
## ---
## Signif. codes:  0 '***' 0.001 '**' 0.01 '*' 0.05 '.' 0.1 ' ' 1
```

```
Ifng_emcatcat_IT <- emmeans(Ifng_model_IT, ~ Treatment * Strain)

#Is there a difference relative to PNS WT offspring?
contrast(Ifng_emcatcat_IT, method = "trt.vs.ctrl", ref = "PNS WT", adjust="BH")
```

```
##  contrast                  estimate   SE    df t.ratio p.value
##  Control WT - PNS WT          -6.49 2.60 10.73  -2.492  0.0432
##  PNS MyD88_KO - PNS WT        -7.31 3.03  7.81  -2.411  0.0432
##  Control MyD88_KO - PNS WT    -7.54 3.15  9.14  -2.390  0.0432
##
## Degrees-of-freedom method: kenward-roger
## P value adjustment: BH method for 3 tests
```

```
#Is there a difference relative to control WT?
contrast(Ifng_emcatcat_IT, method = "trt.vs.ctrl", ref = "Control WT", adjust="BH")
```

```
##  contrast                      estimate   SE    df t.ratio p.value
##  PNS WT - Control WT              6.492 2.60 10.73   2.492  0.0912
##  PNS MyD88_KO - Control WT       -0.823 3.26  8.27  -0.252  0.8072
##  Control MyD88_KO - Control WT   -1.044 3.38  9.47  -0.309  0.8072
##
## Degrees-of-freedom method: kenward-roger
## P value adjustment: BH method for 3 tests
```

```
#Was there a difference between control and PNS in MyD88 KO mice?
Ifng_emcatcat_IT2 <- emmeans(Ifng_model_IT, ~ Treatment | Strain)
contrast(Ifng_emcatcat_IT2, method = "pairwise", adjust="BH")
```

```
## Strain = WT:
##  contrast      estimate   SE    df t.ratio p.value
##  PNS - Control    6.492 2.60 10.73   2.492  0.0304
##
## Strain = MyD88_KO:
##  contrast      estimate   SE    df t.ratio p.value
##  PNS - Control    0.222 3.72  7.85   0.060  0.9539
##
## Degrees-of-freedom method: kenward-roger
```

Ileum Tissue *Ifnb* Expression

```
##Ifnb
# Fit linear mixed-effects model
# Include random intercept for litter
Ifnb_model_IT <- lmer(Ifnb ~ Strain * Treatment + (1 | Litter), data = myd88_ileum_dat)
```

```
## boundary (singular) fit: see help('isSingular')
```

```
summary(Ifnb_model_IT)
```

```
## Linear mixed model fit by REML. t-tests use Satterthwaite's method [
## lmerModLmerTest]
## Formula: Ifnb ~ Strain * Treatment + (1 | Litter)
##    Data: myd88_ileum_dat
##
## REML criterion at convergence: 160.3
##
## Scaled residuals:
##      Min       1Q   Median       3Q      Max
## -2.00157 -0.29071 -0.19164  0.04203  2.66946
##
## Random effects:
##  Groups   Name        Variance Std.Dev.
##  Litter   (Intercept)  0.00    0.000
##  Residual             11.09    3.331
## Number of obs: 33, groups:  Litter, 14
##
## Fixed effects:
##                                  Estimate Std. Error     df t value Pr(>|t|)
## (Intercept)                         9.227      1.053 29.000   8.760 1.22e-09 ***
## StrainMyD88_KO                     -7.579      1.426 29.000  -5.314 1.06e-05 ***
## TreatmentControl                   -6.207      1.720 29.000  -3.609  0.00114 **
## StrainMyD88_KO:TreatmentControl    5.889      2.470 29.000   2.384  0.02390 *
## ---
## Signif. codes:  0 '***' 0.001 '**' 0.01 '*' 0.05 '.' 0.1 ' ' 1
##
## Correlation of Fixed Effects:
##             (Intr) StMD88_KO TrtmnC
## StrnMD88_KO -0.739
## TrtmntCntrl -0.612  0.452
## SMD88_KO:TC  0.426 -0.577    -0.696
## optimizer (nloptwrap) convergence code: 0 (OK)
## boundary (singular) fit: see help('isSingular')
```

```
# Create a type III ANOVA table
Ifnb_anova_table_IT <- anova(Ifnb_model_IT, ddf = "Kenward-Roger")
print(Ifnb_anova_table_IT)
```

```
## Type III Analysis of Variance Table with Kenward-Roger's method
##                   Sum Sq Mean Sq NumDF  DenDF F value   Pr(>F)
## Strain           153.835 153.835     1 8.6843 13.8655 0.005051 **
## Treatment         76.248  76.248     1 8.6843  6.8724 0.028559 *
## Strain:Treatment  62.095  62.095     1 8.6843  5.5967 0.043170 *
## ---
## Signif. codes:  0 '***' 0.001 '**' 0.01 '*' 0.05 '.' 0.1 ' ' 1
```

```
Ifnb_emcatcat_IT <- emmeans(Ifnb_model_IT, ~ Treatment * Strain)

#Is there a difference relative to PNS WT offspring?
contrast(Ifnb_emcatcat_IT, method = "trt.vs.ctrl", ref = "PNS WT", adjust="BH")
```

```
##  contrast                  estimate   SE    df t.ratio p.value
##  Control WT - PNS WT          -6.21 1.73 18.13  -3.579  0.0032
##  PNS MyD88_KO - PNS WT        -7.58 1.43  4.53  -5.295  0.0043
##  Control MyD88_KO - PNS WT    -7.90 1.84 11.27  -4.290  0.0032
##
## Degrees-of-freedom method: kenward-roger
## P value adjustment: BH method for 3 tests
```

```
#Is there a difference relative to control WT?
contrast(Ifnb_emcatcat_IT, method = "trt.vs.ctrl", ref = "Control WT", adjust="BH")
```

```
##  contrast                      estimate   SE   df t.ratio p.value
##  PNS WT - Control WT               6.21 1.73 18.1   3.579  0.0064
##  PNS MyD88_KO - Control WT        -1.37 1.68  6.5  -0.819  0.4420
##  Control MyD88_KO - Control WT    -1.69 2.04 12.7  -0.830  0.4420
##
## Degrees-of-freedom method: kenward-roger
## P value adjustment: BH method for 3 tests
```

```
#Was there a difference between control and PNS in MyD88 KO mice?
Ifnb_emcatcat_IT2 <- emmeans(Ifnb_model_IT, ~ Treatment | Strain)
contrast(Ifnb_emcatcat_IT2, method = "pairwise", adjust="BH")
```

```
## Strain = WT:
##  contrast      estimate   SE    df t.ratio p.value
##  PNS - Control    6.207 1.73 18.13   3.579  0.0021
##
## Strain = MyD88_KO:
##  contrast      estimate   SE    df t.ratio p.value
##  PNS - Control    0.318 1.79  4.82   0.178  0.8657
##
## Degrees-of-freedom method: kenward-roger
```

Ileum Tissue *Isg15* Expression

```
##Isg15
# Fit linear mixed-effects model
# Include random intercept for litter
Isg15_model_IT <- lmer(Isg15 ~ Strain * Treatment + (1 | Litter), data = myd88_ileum_dat)
summary(Isg15_model_IT)
```

```
## Linear mixed model fit by REML. t-tests use Satterthwaite's method [
## lmerModLmerTest]
## Formula: Isg15 ~ Strain * Treatment + (1 | Litter)
##    Data: myd88_ileum_dat
##
## REML criterion at convergence: 79.1
##
## Scaled residuals:
##     Min      1Q  Median      3Q     Max
## -1.8851 -0.3323 -0.1225  0.2951  2.1786
##
## Random effects:
##  Groups   Name        Variance Std.Dev.
##  Litter   (Intercept) 2.2012   1.484
##  Residual             0.2153   0.464
## Number of obs: 35, groups:  Litter, 14
##
## Fixed effects:
##                                  Estimate Std. Error      df t value Pr(>|t|)
## (Intercept)                        1.4078     0.6250 10.1028   2.253   0.0477 *
## StrainMyD88_KO                    -0.4826     1.2280  9.4113  -0.393   0.7031
## TreatmentControl                   1.1530     0.9871 10.0583   1.168   0.2697
## StrainMyD88_KO:TreatmentControl  -1.0761     1.7992  9.6066  -0.598   0.5636
## ---
## Signif. codes:  0 '***' 0.001 '**' 0.01 '*' 0.05 '.' 0.1 ' ' 1
##
## Correlation of Fixed Effects:
##             (Intr) StMD88_KO TrtmnC
## StrnMD88_KO -0.509
## TrtmntCntrl -0.633  0.322
## SMD88_KO:TC  0.347 -0.683    -0.549
```

```
# Create a type III ANOVA table
Isg15_anova_table_IT <- anova(Isg15_model_IT, ddf = "Kenward-Roger")
print(Isg15_anova_table_IT)
```

```
## Type III Analysis of Variance Table with Kenward-Roger's method
##                    Sum Sq  Mean Sq NumDF  DenDF F value Pr(>F)
## Strain           0.277078 0.277078     1 9.6629  1.2871 0.2839
## Treatment        0.100575 0.100575     1 9.6629  0.4672 0.5103
## Strain:Treatment 0.077006 0.077006     1 9.6629  0.3577 0.5635
```

```
Isg15_emcatcat_IT <- emmeans(Isg15_model_IT, ~ Treatment * Strain)

#Is there a difference relative to PNS WT offspring?
contrast(Isg15_emcatcat_IT, method = "trt.vs.ctrl", ref = "PNS WT", adjust="BH")
```

```
##  contrast                  estimate    SE    df t.ratio p.value
##  Control WT - PNS WT          1.153 0.987 10.12   1.168  0.7503
##  PNS MyD88_KO - PNS WT       -0.483 1.230  9.47  -0.393  0.7503
##  Control MyD88_KO - PNS WT   -0.406 1.240  9.83  -0.327  0.7503
##
## Degrees-of-freedom method: kenward-roger
## P value adjustment: BH method for 3 tests
```

```
#Is there a difference relative to control WT?
contrast(Isg15_emcatcat_IT, method = "trt.vs.ctrl", ref = "Control WT", adjust="BH")
```

```
##  contrast                      estimate    SE    df t.ratio p.value
##  PNS WT - Control WT              -1.15 0.987 10.12  -1.168  0.2696
##  PNS MyD88_KO - Control WT        -1.64 1.300  9.52  -1.254  0.2696
##  Control MyD88_KO - Control WT    -1.56 1.320  9.84  -1.185  0.2696
##
## Degrees-of-freedom method: kenward-roger
## P value adjustment: BH method for 3 tests
```

```
#Was there a difference between control and PNS in MyD88 KO mice?
Isg15_emcatcat_IT2 <- emmeans(Isg15_model_IT, ~ Treatment | Strain)
contrast(Isg15_emcatcat_IT2, method = "pairwise", adjust="BH")
```

```
## Strain = WT:
##  contrast      estimate    SE    df t.ratio p.value
##  PNS - Control  -1.1530 0.987 10.12  -1.168  0.2696
##
## Strain = MyD88_KO:
##  contrast      estimate    SE    df t.ratio p.value
##  PNS - Control  -0.0769 1.500  9.48  -0.051  0.9603
##
## Degrees-of-freedom method: kenward-roger
```

Ileum Tissue *Il1b* Expression

```
##Il1b
# Fit linear mixed-effects model
# Include random intercept for litter
Il1b_model_IT <- lmer(Il1b ~ Strain * Treatment + (1 | Litter), data = myd88_ileum_dat)
summary(Il1b_model_IT)
```

```
## Linear mixed model fit by REML. t-tests use Satterthwaite's method [
## lmerModLmerTest]
## Formula: Il1b ~ Strain * Treatment + (1 | Litter)
##    Data: myd88_ileum_dat
##
## REML criterion at convergence: 91.5
##
## Scaled residuals:
##      Min       1Q   Median       3Q      Max
## -1.58360 -0.30210 -0.01676  0.27578  2.00908
##
## Random effects:
##  Groups   Name        Variance Std.Dev.
##  Litter   (Intercept) 3.5606   1.8870
##  Residual             0.3384   0.5817
## Number of obs: 34, groups:  Litter, 14
##
## Fixed effects:
##                                   Estimate Std. Error      df t value Pr(>|t|)
## (Intercept)                         3.6170     0.7942  9.8030   4.554  0.00111 **
## StrainMyD88_KO                     -2.8437     1.5621  9.1616  -1.820  0.10144
## TreatmentControl                   -1.2589     1.2544  9.7603  -1.004  0.33978
## StrainMyD88_KO:TreatmentControl   1.5075     2.2877  9.3373   0.659  0.52584
## ---
## Signif. codes:  0 '***' 0.001 '**' 0.01 '*' 0.05 '.' 0.1 ' ' 1
##
## Correlation of Fixed Effects:
##             (Intr) StMD88_KO TrtmnC
## StrnMD88_KO -0.508
## TrtmntCntrl -0.633  0.322
## SMD88_KO:TC  0.347 -0.683    -0.548
```

```
# Create a type III ANOVA table
Il1b_anova_table_IT <- anova(Il1b_model_IT, ddf = "Kenward-Roger")
print(Il1b_anova_table_IT)
```

```
## Type III Analysis of Variance Table with Kenward-Roger's method
##                   Sum Sq Mean Sq NumDF  DenDF F value  Pr(>F)
## Strain           1.12970 1.12970     1 9.6787  3.3382 0.09864 .
## Treatment        0.06601 0.06601     1 9.6787  0.1951 0.66845
## Strain:Treatment 0.14694 0.14694     1 9.6787  0.4342 0.52530
## ---
## Signif. codes:  0 '***' 0.001 '**' 0.01 '*' 0.05 '.' 0.1 ' ' 1
```

```
Il1b_emcatcat_IT <- emmeans(Il1b_model_IT, ~ Treatment * Strain)

#Is there a difference relative to PNS WT offspring?
contrast(Il1b_emcatcat_IT, method = "trt.vs.ctrl", ref = "PNS WT", adjust="BH")
```

```
##  contrast                  estimate   SE    df t.ratio p.value
##  Control WT - PNS WT          -1.26 1.25 10.11  -1.004  0.3390
##  PNS MyD88_KO - PNS WT        -2.84 1.56  9.50  -1.820  0.1966
##  Control MyD88_KO - PNS WT    -2.60 1.58  9.83  -1.647  0.1966
##
## Degrees-of-freedom method: kenward-roger
## P value adjustment: BH method for 3 tests
```

```
#Is there a difference relative to control WT?
contrast(Il1b_emcatcat_IT, method = "trt.vs.ctrl", ref = "Control WT", adjust="BH")
```

```
##  contrast                      estimate   SE    df t.ratio p.value
##  PNS WT - Control WT              1.26 1.25 10.11   1.004  0.4429
##  PNS MyD88_KO - Control WT       -1.58 1.66  9.55  -0.955  0.4429
##  Control MyD88_KO - Control WT   -1.34 1.67  9.84  -0.799  0.4429
##
## Degrees-of-freedom method: kenward-roger
## P value adjustment: BH method for 3 tests
```

```
#Was there a difference between control and PNS in MyD88 KO mice?
Il1b_emcatcat_IT2 <- emmeans(Il1b_model_IT, ~ Treatment | Strain)
contrast(Il1b_emcatcat_IT2, method = "pairwise", adjust="BH")
```

```
## Strain = WT:
##  contrast      estimate   SE   df t.ratio p.value
##  PNS - Control    1.259 1.25 10.1   1.004  0.3390
##
## Strain = MyD88_KO:
##  contrast      estimate   SE   df t.ratio p.value
##  PNS - Control   -0.249 1.91  9.5  -0.130  0.8993
##
## Degrees-of-freedom method: kenward-roger
```

Ileum Tissue *Il6* Expression

```
##Il6
# Fit linear mixed-effects model
# Include random intercept for litter
Il6_model_IT <- lmer(Il6 ~ Strain * Treatment + (1 | Litter), data = myd88_ileum_dat)
summary(Il6_model_IT)
```

```
## Linear mixed model fit by REML. t-tests use Satterthwaite's method [
## lmerModLmerTest]
## Formula: Il6 ~ Strain * Treatment + (1 | Litter)
##    Data: myd88_ileum_dat
##
## REML criterion at convergence: 131.4
##
## Scaled residuals:
##      Min       1Q   Median       3Q      Max
## -1.73935 -0.22735 -0.03616  0.09827  2.40614
##
## Random effects:
##  Groups   Name        Variance Std.Dev.
##  Litter   (Intercept) 4.694    2.167
##  Residual             2.421    1.556
## Number of obs: 33, groups:  Litter, 14
##
## Fixed effects:
##                                  Estimate Std. Error     df t value Pr(>|t|)
## (Intercept)                         5.925      1.021  8.728   5.801 0.000291 ***
## StrainMyD88_KO                     -4.745      1.895  6.350  -2.504 0.044154 *
## TreatmentControl                   -3.994      1.630  9.063  -2.451 0.036540 *
## StrainMyD88_KO:TreatmentControl    3.838      2.838  7.139   1.353 0.217486
## ---
## Signif. codes:  0 '***' 0.001 '**' 0.01 '*' 0.05 '.' 0.1 ' ' 1
##
## Correlation of Fixed Effects:
##             (Intr) StMD88_KO TrtmnC
## StrnMD88_KO -0.539
## TrtmntCntrl -0.627  0.338
## SMD88_KO:TC  0.360 -0.668    -0.574
```

```
# Create a type III ANOVA table
Il6_anova_table_IT <- anova(Il6_model_IT, ddf = "Kenward-Roger")
print(Il6_anova_table_IT)
```

```
## Type III Analysis of Variance Table with Kenward-Roger's method
##                  Sum Sq Mean Sq NumDF  DenDF F value  Pr(>F)
## Strain           9.5852  9.5852     1 8.6337  3.9594 0.07917 .
## Treatment        5.1700  5.1700     1 8.6337  2.1356 0.17933
## Strain:Treatment 4.4207  4.4207     1 8.6337  1.8261 0.21093
## ---
## Signif. codes:  0 '***' 0.001 '**' 0.01 '*' 0.05 '.' 0.1 ' ' 1
```

```
Il6_emcatcat_IT <- emmeans(Il6_model_IT, ~ Treatment * Strain)

#Is there a difference relative to PNS WT offspring?
contrast(Il6_emcatcat_IT, method = "trt.vs.ctrl", ref = "PNS WT", adjust="BH")
```

```
##  contrast                  estimate   SE    df t.ratio p.value
##  Control WT - PNS WT          -3.99 1.63 10.76  -2.445  0.0378
##  PNS MyD88_KO - PNS WT        -4.74 1.90  7.74  -2.502  0.0378
##  Control MyD88_KO - PNS WT    -4.90 1.97  9.12  -2.483  0.0378
##
## Degrees-of-freedom method: kenward-roger
## P value adjustment: BH method for 3 tests
```

```
#Is there a difference relative to control WT?
contrast(Il6_emcatcat_IT, method = "trt.vs.ctrl", ref = "Control WT", adjust="BH")
```

```
##  contrast                      estimate   SE    df t.ratio p.value
##  PNS WT - Control WT              3.994 1.63 10.76   2.445  0.0989
##  PNS MyD88_KO - Control WT       -0.750 2.04  8.21  -0.368  0.7225
##  Control MyD88_KO - Control WT   -0.907 2.11  9.45  -0.429  0.7225
##
## Degrees-of-freedom method: kenward-roger
## P value adjustment: BH method for 3 tests
```

```
#Was there a difference between control and PNS in MyD88 KO mice?
Il6_emcatcat_IT2 <- emmeans(Il6_model_IT, ~ Treatment | Strain)
contrast(Il6_emcatcat_IT2, method = "pairwise", adjust="BH")
```

```
## Strain = WT:
##  contrast      estimate   SE    df t.ratio p.value
##  PNS - Control    3.994 1.63 10.76   2.445  0.0330
##
## Strain = MyD88_KO:
##  contrast      estimate   SE    df t.ratio p.value
##  PNS - Control    0.156 2.32  7.78   0.067  0.9481
##
## Degrees-of-freedom method: kenward-roger
```

Ileum Tissue *Tnfa* Expression

```
##Tnf
# Fit linear mixed-effects model
# Include random intercept for litter
Tnf_model_IT <- lmer(Tnf ~ Strain * Treatment + (1 | Litter), data = myd88_ileum_dat)
summary(Tnf_model_IT)
```

```
## Linear mixed model fit by REML. t-tests use Satterthwaite's method [
## lmerModLmerTest]
## Formula: Tnf ~ Strain * Treatment + (1 | Litter)
##    Data: myd88_ileum_dat
##
## REML criterion at convergence: 115.2
##
## Scaled residuals:
##      Min       1Q   Median       3Q      Max
## -1.87775 -0.26777 -0.09551  0.13201  1.98289
##
## Random effects:
##  Groups   Name        Variance Std.Dev.
##  Litter   (Intercept) 1.688    1.299
##  Residual             1.172    1.083
## Number of obs: 35, groups:  Litter, 14
##
## Fixed effects:
##                                 Estimate Std. Error      df t value Pr(>|t|)
## (Intercept)                       4.8291     0.6265 10.0448   7.708 1.59e-05
## StrainMyD88_KO                   -3.7675     1.1551  7.2335  -3.261   0.0132
## TreatmentControl                 -2.4878     0.9959 10.2334  -2.498   0.0311
## StrainMyD88_KO:TreatmentControl   2.4363     1.7378  8.2392   1.402   0.1975
##
## (Intercept)                     ***
## StrainMyD88_KO                  *
## TreatmentControl                *
## StrainMyD88_KO:TreatmentControl
## ---
## Signif. codes:  0 '***' 0.001 '**' 0.01 '*' 0.05 '.' 0.1 ' ' 1
##
## Correlation of Fixed Effects:
##             (Intr) StMD88_KO TrtmnC
## StrnMD88_KO -0.542
## TrtmntCntrl -0.629  0.341
## SMD88_KO:TC  0.361 -0.665    -0.573
```

```
# Create a type III ANOVA table
Tnf_anova_table_IT <- anova(Tnf_model_IT, ddf = "Kenward-Roger")
print(Tnf_anova_table_IT)
```

```
## Type III Analysis of Variance Table with Kenward-Roger's method
##                  Sum Sq Mean Sq NumDF  DenDF F value  Pr(>F)
## Strain          10.0744 10.0744     1 8.6533  8.5938 0.01742 *
## Treatment        2.4988  2.4988     1 8.6533  2.1315 0.17963
## Strain:Treatment  2.3003  2.3003     1 8.6533  1.9623 0.19610
## ---
## Signif. codes:  0 '***' 0.001 '**' 0.01 '*' 0.05 '.' 0.1 ' ' 1
```

```
Tnf_emcatcat_IT <- emmeans(Tnf_model_IT, ~ Treatment * Strain)

#Is there a difference relative to PNS WT offspring?
contrast(Tnf_emcatcat_IT, method = "trt.vs.ctrl", ref = "PNS WT", adjust="BH")
```

```
##  contrast                  estimate    SE    df t.ratio p.value
##  Control WT - PNS WT          -2.49 0.998 10.70  -2.493  0.0304
##  PNS MyD88_KO - PNS WT        -3.77 1.160  7.61  -3.260  0.0185
##  Control MyD88_KO - PNS WT    -3.82 1.220  9.38  -3.138  0.0185
##
## Degrees-of-freedom method: kenward-roger
## P value adjustment: BH method for 3 tests
```

```
#Is there a difference relative to control WT?
contrast(Tnf_emcatcat_IT, method = "trt.vs.ctrl", ref = "Control WT", adjust="BH")
```

```
##  contrast                      estimate    SE    df t.ratio p.value
##  PNS WT - Control WT               2.49 0.998 10.70   2.493  0.0912
##  PNS MyD88_KO - Control WT        -1.28 1.240  8.02  -1.030  0.3331
##  Control MyD88_KO - Control WT    -1.33 1.300  9.61  -1.024  0.3331
##
## Degrees-of-freedom method: kenward-roger
## P value adjustment: BH method for 3 tests
```

```
#Was there a difference between control and PNS in MyD88 KO mice?
Tnf_emcatcat_IT2 <- emmeans(Tnf_model_IT, ~ Treatment | Strain)
contrast(Tnf_emcatcat_IT2, method = "pairwise", adjust="BH")
```

```
## Strain = WT:
##  contrast      estimate    SE    df t.ratio p.value
##  PNS - Control   2.4878 0.998 10.70   2.493  0.0304
##
## Strain = MyD88_KO:
##  contrast      estimate    SE    df t.ratio p.value
##  PNS - Control   0.0515 1.420  7.84   0.036  0.9721
##
## Degrees-of-freedom method: kenward-roger
```

Ileum Tissue *Ahr* Expression

```
##Ahr
# Fit linear mixed-effects model
# Include random intercept for litter
Ahr_model_IT <- lmer(Ahr ~ Strain * Treatment + (1 | Litter), data = myd88_ileum_dat)
summary(Ahr_model_IT)
```

```
## Linear mixed model fit by REML. t-tests use Satterthwaite's method [
## lmerModLmerTest]
## Formula: Ahr ~ Strain * Treatment + (1 | Litter)
##    Data: myd88_ileum_dat
##
## REML criterion at convergence: 145.7
##
## Scaled residuals:
##      Min       1Q   Median       3Q      Max
## -1.93972 -0.25205 -0.00341  0.03145  2.73333
##
## Random effects:
##  Groups   Name        Variance Std.Dev.
##  Litter   (Intercept) 8.094    2.845
##  Residual             3.141    1.772
## Number of obs: 34, groups:  Litter, 14
##
## Fixed effects:
##                                 Estimate Std. Error     df t value Pr(>|t|)
## (Intercept)                        8.037      1.300 10.617   6.183 8.02e-05 ***
## StrainMyD88_KO                    -7.156      2.451  8.426  -2.920   0.0182 *
## TreatmentControl                   1.403      2.048 10.477   0.685   0.5080
## StrainMyD88_KO:TreatmentControl   -1.277      3.634  9.036  -0.351   0.7334
## ---
## Signif. codes:  0 '***' 0.001 '**' 0.01 '*' 0.05 '.' 0.1 ' ' 1
##
## Correlation of Fixed Effects:
##             (Intr) StMD88_KO TrtmnC
## StrnMD88_KO -0.530
## TrtmntCntrl -0.635  0.337
## SMD88_KO:TC  0.358 -0.674    -0.563
```

```
# Create a type III ANOVA table
Ahr_anova_table_IT <- anova(Ahr_model_IT, ddf = "Kenward-Roger")
print(Ahr_anova_table_IT)
```

```
## Type III Analysis of Variance Table with Kenward-Roger's method
##                  Sum Sq Mean Sq NumDF  DenDF F value   Pr(>F)
## Strain           57.750  57.750     1 8.9469 18.3850 0.002054 **
## Treatment         0.556   0.556     1 8.9469  0.1771 0.683798
## Strain:Treatment  0.387   0.387     1 8.9469  0.1233 0.733605
## ---
## Signif. codes:  0 '***' 0.001 '**' 0.01 '*' 0.05 '.' 0.1 ' ' 1
```

```
Ahr_emcatcat_IT <- emmeans(Ahr_model_IT, ~ Treatment * Strain)

#Is there a difference relative to PNS WT offspring?
contrast(Ahr_emcatcat_IT, method = "trt.vs.ctrl", ref = "PNS WT", adjust="BH")
```

```
##  contrast                  estimate   SE    df t.ratio p.value
##  Control WT - PNS WT           1.40 2.05 10.38   0.685  0.5086
##  PNS MyD88_KO - PNS WT        -7.16 2.45  8.34  -2.919  0.0307
##  Control MyD88_KO - PNS WT    -7.03 2.53  9.46  -2.780  0.0307
##
## Degrees-of-freedom method: kenward-roger
## P value adjustment: BH method for 3 tests
```

```
#Is there a difference relative to control WT?
contrast(Ahr_emcatcat_IT, method = "trt.vs.ctrl", ref = "Control WT", adjust="BH")
```

```
##  contrast                      estimate   SE   df t.ratio p.value
##  PNS WT - Control WT              -1.40 2.05 10.4  -0.685  0.5086
##  PNS MyD88_KO - Control WT        -8.56 2.61  8.5  -3.276  0.0167
##  Control MyD88_KO - Control WT    -8.43 2.69  9.5  -3.141  0.0167
##
## Degrees-of-freedom method: kenward-roger
## P value adjustment: BH method for 3 tests
```

```
#Was there a difference between control and PNS in MyD88 KO mice?
Ahr_emcatcat_IT2 <- emmeans(Ahr_model_IT, ~ Treatment | Strain)
contrast(Ahr_emcatcat_IT2, method = "pairwise", adjust="BH")
```

```
## Strain = WT:
##  contrast      estimate   SE    df t.ratio p.value
##  PNS - Control   -1.403 2.05 10.38  -0.685  0.5086
##
## Strain = MyD88_KO:
##  contrast      estimate   SE    df t.ratio p.value
##  PNS - Control   -0.127 3.00  8.37  -0.042  0.9673
##
## Degrees-of-freedom method: kenward-roger
```

Ileum Tissue *Muc2* Expression

```
##Muc2
# Fit linear mixed-effects model
# Include random intercept for litter
Muc2_model_IT <- lmer(Muc2 ~ Strain * Treatment + (1 | Litter), data = myd88_ileum_dat)
summary(Muc2_model_IT)
```

```
## Linear mixed model fit by REML. t-tests use Satterthwaite's method [
## lmerModLmerTest]
## Formula: Muc2 ~ Strain * Treatment + (1 | Litter)
##    Data: myd88_ileum_dat
##
## REML criterion at convergence: 44
##
## Scaled residuals:
##      Min       1Q   Median       3Q      Max
## -1.50094 -0.31622 -0.07359  0.31862  1.95726
##
## Random effects:
##  Groups   Name        Variance Std.Dev.
##  Litter   (Intercept) 0.74782  0.8648
##  Residual             0.06537  0.2557
## Number of obs: 36, groups:  Litter, 14
##
## Fixed effects:
##                                  Estimate Std. Error       df t value Pr(>|t|)
## (Intercept)                       1.00291    0.36189 10.07493   2.771   0.0196 *
## StrainMyD88_KO                    0.07456    0.71409  9.54781   0.104   0.9190
## TreatmentControl                 -0.34976    0.57277 10.11480  -0.611   0.5549
## StrainMyD88_KO:TreatmentControl  0.28135    1.04620  9.74242   0.269   0.7936
## ---
## Signif. codes:  0 '***' 0.001 '**' 0.01 '*' 0.05 '.' 0.1 ' ' 1
##
## Correlation of Fixed Effects:
##             (Intr) StMD88_KO TrtmnC
## StrnMD88_KO -0.507
## TrtmntCntrl -0.632  0.320
## SMD88_KO:TC  0.346 -0.683    -0.547
```

```
# Create a type III ANOVA table
Muc2_anova_table_IT <- anova(Muc2_model_IT, ddf = "Kenward-Roger")
print(Muc2_anova_table_IT)
```

```
## Type III Analysis of Variance Table with Kenward-Roger's method
##                     Sum Sq   Mean Sq NumDF  DenDF F value Pr(>F)
## Strain           0.0110665 0.0110665     1 9.7457  0.1693 0.6896
## Treatment        0.0104425 0.0104425     1 9.7457  0.1598 0.6980
## Strain:Treatment 0.0047272 0.0047272     1 9.7457  0.0723 0.7936
```

```
Muc2_emcatcat_IT <- emmeans(Muc2_model_IT, ~ Treatment * Strain)

#Is there a difference relative to PNS WT offspring?
contrast(Muc2_emcatcat_IT, method = "trt.vs.ctrl", ref = "PNS WT", adjust="BH")
```

```
##  contrast                  estimate    SE    df t.ratio p.value
##  Control WT - PNS WT       -0.34976 0.573 10.12  -0.611  0.9933
##  PNS MyD88_KO - PNS WT      0.07456 0.714  9.55   0.104  0.9933
##  Control MyD88_KO - PNS WT  0.00616 0.720  9.88   0.009  0.9933
##
## Degrees-of-freedom method: kenward-roger
## P value adjustment: BH method for 3 tests
```

```
#Is there a difference relative to control WT?
contrast(Muc2_emcatcat_IT, method = "trt.vs.ctrl", ref = "Control WT", adjust="BH")
```

```
##  contrast                      estimate    SE    df t.ratio p.value
##  PNS WT - Control WT              0.350 0.573 10.12   0.611  0.6516
##  PNS MyD88_KO - Control WT        0.424 0.759  9.63   0.559  0.6516
##  Control MyD88_KO - Control WT    0.356 0.765  9.92   0.465  0.6516
##
## Degrees-of-freedom method: kenward-roger
## P value adjustment: BH method for 3 tests
```

```
#Was there a difference between control and PNS in MyD88 KO mice?
Muc2_emcatcat_IT2 <- emmeans(Muc2_model_IT, ~ Treatment | Strain)
contrast(Muc2_emcatcat_IT2, method = "pairwise", adjust="BH")
```

```
## Strain = WT:
##  contrast      estimate    SE    df t.ratio p.value
##  PNS - Control   0.3498 0.573 10.12   0.611  0.5549
##
## Strain = MyD88_KO:
##  contrast      estimate    SE    df t.ratio p.value
##  PNS - Control   0.0684 0.875  9.59   0.078  0.9393
##
## Degrees-of-freedom method: kenward-roger
```
